# Supplementary material for: Aspergillus fumigatus promotes tumor angiogenesis via SLC7A11 on myeloid-derived suppressor cells
Source: EMBO Rep. 2025 Nov 17;26(24):6266–91. doi: 10.1038/s44319-025-00627-x (PMC12715260; doi:10.1038/s44319-025-00627-x)
Supplement: Supplementary file 1 — Appendix [file 44319_2025_627_MOESM1_ESM.pdf]

Aspergillus fumigatus promotes tumor angiogenesis via SLC7A11 on myeloid-derived suppressor cells

Appendix

Content：

Appendix Table S1 page 2

Appendix Table S2 page 3

Appendix Table S3 page 4

Appendix Table S4 page 5

Appendix Table S1: SLC7A11-Interacting Proteins in Unstimulated MDSCs (Control) Identified by Co-IP/MS

Protein FDR Confidence:	Accession	Sum PEP Score	Coverage [%]	# Peptides	# PSMs	# Unique Peptides	# AAs	MW [kDa]	calc. pI	Score Sequest HT: Sequest HT	Gene Symbol	Abundances (Normalized): F1:
High	Q8VDD5	379.075	51	99	145	87	1960	226.2	5.66	430.9	Myh9	1424196821
High	P60710	87.542	58	22	40	10	375	41.7	5.48	108.09	Actb	1297216539
High	P68134	54.63	32	14	27	2	377	42	5.39	70.66	Acta1	14243835.5
High	Q60605	43.907	58	8	17	8	151	16.9	4.65	50.37	Myl6	492411843.9
High	Q61879	41.828	7	13	18	1	1976	228.9	5.54	41.55	Myh10	2723143.25
High	P02535	39.398	14	11	22	8	570	57.7	5.11	61.24	Krt10	1260812084
High	Q922U2	38.42	18	17	25	7	580	61.7	7.75	59.66	Krt5	924691086.1
High	Q6IFZ6	33.254	12	10	15	4	572	61.3	8.02	43.29	Krt77	480932278.3
High	Q3TTY5	32.882	10	11	17	6	707	70.9	8.06	48.64	Krt2	296845183
High	Q3THE2	29.332	52	7	9	7	172	19.8	4.84	31.88	Myl12b	197359861.3
High	P08730	26.978	14	7	13	1	437	47.7	4.86	39.52	Krt13	108623958.3
High	Q61414	25.972	13	7	12	3	452	49.1	4.86	30.1	Krt15	126118133
High	P20152	24.651	31	13	15	12	466	53.7	5.12	30.42	Vim	61004037.88
High	P01942	24.375	28	3	8	3	142	15.1	8.22	26.91	Hba	160801594
High	P07724	22	20	10	10	10	608	68.6	6.07	29.34	Alb	163495478.9
High	P21107	20.145	31	7	10	7	285	33	4.72	23.11	Tpm3	39024039.75
High	Q9CZX8	16.014	40	7	11	7	145	16.1	10.4	28.45	Rps19	52732846.5
High	Q9QWL7	15.905	14	5	6	1	433	48.1	5.06	17.86	Krt17	2025767.25
High	P04104	15.714	6	5	8	2	637	65.6	8.15	24.89	Krt1	466653012.8
High	Q8VED5	15.681	8	7	10	1	531	57.5	7.69	20.32	Krt79	151347373
High	P50446	15.261	13	9	12	1	553	59.3	7.94	27.68	Krt6a	2281289.25
High	Q61781	15.26	18	6	6	1	484	52.8	5.17	18.87	Krt14	14188518.56
High	P02088	15.229	42	5	6	5	147	15.8	7.65	14.55	Hbb-b1	145828809.9
High	Q6IFX2	14.591	15	6	6	3	452	50.1	5.16	18.83	Krt42	10073436.75
High	Q3UV17	13.366	9	8	9	2	594	62.8	8.43	18.31	Krt76	399763582.6
High	P19001	10.302	11	5	6	1	403	44.5	5.39	12.49	Krt19	
High	Q6ZWV7	9.135	30	3	4	3	123	14.5	11.05	6.53	Rpl35	14421837.63
High	P28293	8.961	23	6	6	6	261	29.1	10.56	15.65	Ctsg	35949659.88
High	Q6NXH9	8.616	8	5	8	1	539	58.9	8.09	21.85	Krt73	194367844.5
High	P62082	8.557	19	3	3	3	194	22.1	10.1	10.04	Rps7	4075976.125
High	P14115	8.541	32	5	5	5	148	16.6	11.12	11.32	Rpl27a	69251967.25
High	P31725	8.427	13	2	2	2	113	13	7.17	7.3	S100a9	9131524
High	P0DP27	8.009	32	3	4	3	149	16.8	4.22	6.25	Calm2	11401552
High	P10852	7.636	11	4	4	4	526	58.3	5.91	7.82	Slc3a2	7709057.375
High	P63038	7.568	12	4	4	4	573	60.9	6.18	8.19	Hspd1	4468773.75
High	Q02257	7.138	4	2	2	2	745	81.7	6.14	7.57	Jup	2287967.25
High	Q64475	6.771	28	3	3	3	126	13.9	10.32	8.44	H2bc3	23179809.63
High	Q9Z204	6.365	4	1	2	1	313	34.4	5.05	6.67	Hnrnpc	29132812.5
High	P47754	6.131	13	3	3	2	286	32.9	5.85	7.85	Capza2	13672541.75
High	P60867	6.011	19	2	2	2	119	13.4	9.94	7.61	Rps20	9099193
High	P28798	5.871	4	2	2	2	589	63.4	6.8	6.37	Grn	11165511.5
High	P62702	5.847	16	5	5	5	263	29.6	10.15	9.48	Rps4x	19029109.75
High	P86048	4.974	11	3	3	3	214	24.5	10.11	4.18	Rpl10l	4099600.625
High	P47753	4.92	10	2	2	1	286	32.9	5.55	4.93	Capza1	2329476
High	P97461	4.383	11	2	2	2	204	22.9	9.72	6.44		7514139.75
High	P62242	4.314	12	2	2	2	208	24.2	10.32	5.18	Rps8	4743480.5
High	E9Q557	4.287	1	3	3	3	2883	332.7	6.8	5.57	Dsp	5660818.5
High	P62908	4.181	9	2	2	2	243	26.7	9.66	5.84	Rps3	5665052.5
High	P01630	4.008	21	2	2	2	113	12.5	8.65	5.96		11568951.5
High	Q9CXW4	3.989	12	3	3	3	178	20.2	9.6	5.66	Rpl11	12736647.56
High	P30681	3.949	12	2	2	2	210	24.1	7.31	5.44	Hmgb2	1125914.5
High	P62806	3.844	30	3	3	3	103	11.4	11.36	6.47	H4c1; H4c11; H4c12; H4c14; H4c16; H4c2;	10047254.75
High	P62984	3.844	23	2	2	2	128	14.7	9.83	5.72	Uba52	8385607
High	P14131	3.707	14	2	2	2	146	16.4	10.21	5.07	Rps16	12653781.5
High	P27005	3.685	27	1	2	1	89	10.3	5.68	5.54	S100a8	8054707.5
High	P47915	3.665	12	2	2	2	160	17.6	11.84	5.37	Rpl29	39761644
High	Q9D154	3.608	6	2	2	2	379	42.5	6.21	5.14	Serpinb1a	6084121.25
High	P32848	3.55	13	1	1	1	110	11.9	5.19	2.5	Pvalb	1342521.25
High	P59999	3.408	11	2	2	2	168	19.7	8.43	4.47	Arpc4	5757423
High	P17897	3.173	14	2	2	1	148	16.8	9.41	3.24	Lyz1	5647912.25
High	P97351	3.172	11	3	3	3	264	29.9	9.73	3.9	Rps3a	14188913
High	P62918	3.152	7	2	2	2	257	28	11.03	5.12	Rpl8	7490234
High	P61255	3.135	16	2	2	2	145	17.2	10.55	2.33	Rpl26	2075264.5
High	Q922Q8	3.109	12	2	2	2	307	34.9	9.52	5.87	Lrrc59	3585898.625
High	P63276	2.941	8	2	2	2	135	15.5	9.85	5.17	Rps17	9494932.5
High	P51881	2.897	6	2	2	2	298	32.9	9.73	2.27	Slc25a5	7853052.5
High	P07356	2.843	4	1	1	1	339	38.7	7.69	3.14	Anxa2	1301076.375
High	P61514	2.809	22	2	2	2	92	10.3	10.43	3.82	Rpl37a	9782476
High	Q99P91	2.746	3	1	1	1	574	63.6	7.61	3.32	Gpnmb	2030807.625
High	P61358	2.538	13	2	2	2	136	15.8	10.56	3.61	Rpl27	9833891
High	P70248	2.508	1	1	1	1	1099	125.9	8.92	0	Myo1f	512570.625
High	Q9CR57	2.486	6	1	1	1	217	23.5	11.02	2.63	Rpl14	
High	P67984	2.398	10	1	1	1	128	14.8	9.19	2.89	Rpl22	5034876
High	O08692	2.342	18	2	2	2	167	19.3	5.31	5.29	Ngp	10950139.75
High	P62264	2.339	7	1	1	1	151	16.3	10.05	2.75	Rps14	2210728.25
High	Q9JJI8	2.246	19	1	1	1	70	8.2	10.1	2.42	Rpl38	1434227.625
High	P27659	2.223	2	2	2	2	403	46.1	10.21	4.67	Rpl3	2781582.375
High	Q99JY9	2.214	7	2	2	2	418	47.3	5.88	5.15	Actr3	2753610
High	Q9WV32	2.193	3	1	1	1	372	41	8.35	2.1	Arpc1b	1371730
High	Q9WTR6	2.148	2	1	1	1	502	55.4	9.23	3.01	Slc7a11	1800947.125
High	Q64467	1.931	4	2	2	2	440	47.6	7.88	3.05	Gapdhs	17028523
High	P56135	1.83	14	1	1	1	88	10.3	9.95	2.97	Atp5mf	5232630.5
High	P01867	1.784	4	1	1	1	404	44.2	6.52	2.71	Ighg2b	4740334
High	P08905	1.767	20	2	2	1	148	16.7	8.81	0	Lyz2	
High	Q8R0X7	1.763	2	1	1	1	568	63.6	9.1	2.64	Sgpl1	1098889.25
High	P47911	1.699	5	1	1	1	296	33.5	10.7	2.09	Rpl6	
High	Q61495	1.675	2	1	1	1	1057	114.5	4.89	2.02	Dsg1a	1286069.75
High	P17182	1.631	3	1	1	1	434	47.1	6.8	2.63	Eno1	1182950.375
High	Q3UND0	1.597	3	1	1	1	358	40.7	4.7	2.09	Skap2	2016221.75
High	Q9QY15	1.596	6	2	2	2	484	54.8	5.99	0	Ddx25	117892886.8
High	P35980	1.591	5	1	1	1	188	21.6	11.78	2.4	Rpl18	1191355.5
High	P51410	1.587	14	2	2	2	192	21.9	9.95	4.67	Rpl9	2437702.5
High	P62830	1.524	6	1	1	1	140	14.9	10.51	0	Rpl23	2089609.875
High	P05064	1.5	4	1	1	1	364	39.3	8.09	0	Aldoa	1379373.125
High	P62267	1.446	8	1	1	1	143	15.8	10.49	2.46	Rps23	1348148.625
High	P62852	1.44	7	1	1	1	125	13.7	10.11	2.52	Rps25	28324924
High	P62962	1.336	13	1	1	1	140	14.9	8.28	2.59	Pfn1	738371.375
High	P01132	1.335	2	1	1	1	1217	133	6.46	0	Egf	
High	P62855	1.327	13	1	1	1	115	13	11	2.91	Rps26	2106526
High	Q6ZQ06	1.312	0	1	1	1	1403	160.8	5.53	2.64	Cep162	2409683.75
High	Q9Z2E3	1.256	2	1	1	1	911	101.2	6.98	0	Ern2	3270879.75
High	Q80UW5	1.211	1	1	1	1	1551	172	6.05	2.6	Cdc42bpg	60875096
High	P63017	1.107	2	1	1	1	646	70.8	5.52	2.4	Hspa8	952216.0625
High	Q99M74	1.1	2	1	1	1	516	57.1	6.25	2.82	Krt82	131121040
High	O35261	1.095	8	1	1	1	457	48.7	5.44	0	E2f3	
High	P14069	1.019	9	1	1	1	89	10	5.48	1.85	S100a6	2805186.25
High	P11247	1.013	2	1	1	1	718	81.1	9.55	0	Mpo	1033972.563
High	Q9CPT0	1.005	3	1	1	1	328	37	7.49	1.88	Bcl2l14	3550211
High	P01837	0.989	7	1	1	1	107	11.9	5.9	2.17	Igkc	4727842.5
High	P63325	0.957	5	1	1	1	165	18.9	10.15	1.88	Rps10	1642456.125
High	P47757	0.954	4	1	1	1	277	31.3	5.74	1.98	Capzb	5727784.5
High	Q924C1	0.936	2	1	1	1	1204	136.9	5.82	2.12	Xpo5	2374897
High	A6BLY7	0.931	2	1	1	1	462	50.3	5.29	2.61	Krt28	72189384
High	Q8VEK3	0.918	1	1	1	1	800	87.9	6.24	0	Hnrnpu	2349465.75
High	Q64523	0.916	7	1	1	1	129	14	10.9	1.93	H2ac20	25765558
High	Q925N2	0.885	6	1	1	1	322	36.1	8.82	0	Sfxn2	36998888
High	P62301	0.833	5	1	1	1	151	17.2	10.54	2	Rps13	8086739.5
High	P10126	0.823	2	1	1	1	462	50.1	9.01	2.46	Eef1a1	6120314.5
High	Q9D7S7	0.798	6	1	1	1	122	14.5	9.45	0	Rpl22l1	1344387
High	Q62261	0.789	1	1	1	1	2363	274.1	5.58	0	Sptbn1	2875077.5
High	Q3V1N1	0.779	1	1	1	1	1048	116.5	7.18	2.47	Mfhas1	6419190
High	Q9D6H2	0.774	5	1	1	1	143	16.3	5.16	0	Ift25	4747180.5
High	Q91VR8	0.759	11	1	1	1	75	8.8	5.45	1.68	Brk1	1821198.125
High	Q6P2L6	0.758	1	1	1	1	1439	160.9	8.27	0	Nsd3	
Medium	Q2EMV9	0.754	1	1	1	1	1817	203.7	6.79	0	Parp14	

Appendix Table S2: SLC7A11-Interacting Proteins in *A. fumigatus*-Stimulated MDSCs Identified by Co-IP/MS

Protein FDR Confidence:	Accession	Sum PEP Score	Coverage [%]	# Peptides	# PSMs	# Unique Peptides	# AAs	MW [kDa]	calc. pI	Score Sequest HT: Sequest HT	Gene Symbol	Abundances (Normalized): F2:
High	Q8VDD5	269.16	45	82	115	82	1960	226.2	5.66	350.62	Myh9	1006760021
High	P60710	59.081	54	18	30	6	375	41.7	5.48	90.86	Actb	1093346922
High	P68134	36.287	32	13	21	1	377	42	5.39	57.13	Acta1	2372989.25
High	P02535	31.83	12	9	16	6	570	57.7	5.11	53.41	Krt10	990425732
High	Q60605	27.459	51	7	11	7	151	16.9	4.65	39.27	Myl6	517598913.7
High	Q922U2	26.379	13	9	16	6	580	61.7	7.75	42.65	Krt5	730853598.3
High	Q3THE2	25.38	47	7	9	7	172	19.8	4.84	30.82	Myl12b	202878151.3
High	Q61414	23.27	13	7	12	3	452	49.1	4.86	32.07	Krt15	262597629.8
High	P07724	23.11	21	10	10	10	608	68.6	6.07	29.26	Alb	179388452.9
High	P21107	20.928	28	7	11	7	285	33	4.72	28.38	Tpm3	38903209.25
High	Q3TTY5	20.774	8	6	11	5	707	70.9	8.06	35.63	Krt2	291230767
High	P01942	18.233	28	3	5	3	142	15.1	8.22	21.97	Hba	149739605
High	Q6IFZ6	16.681	8	5	9	3	572	61.3	8.02	25.34	Krt77	354096427.3
High	P20152	16.279	17	6	7	6	466	53.7	5.12	20.38	Vim	19315149
High	P04104	16.012	5	5	10	3	637	65.6	8.15	29.86	Krt1	719304000.5
High	P08730	15.762	12	5	9	1	437	47.7	4.86	27.47	Krt13	46406984
High	Q61781	14.345	17	7	7	2	484	52.8	5.17	18.83	Krt14	24744419.5
High	Q6IFX2	12.681	14	6	6	2	452	50.1	5.16	18.79	Krt42	25618835
High	P01867	11.909	26	5	6	5	404	44.2	6.52	14.58	Ighg2b	52711781
High	P02088	11.629	31	4	5	4	147	15.8	7.65	16.05	Hbb-b1	131890576.5
High	P50446	11.566	10	5	8	2	553	59.3	7.94	22.58	Krt6a	8221169
High	Q6ZWV7	11.543	30	5	6	5	123	14.5	11.05	13.74	Rpl35	15000590.5
High	Q9CZX8	10.282	34	6	7	6	145	16.1	10.4	18.84	Rps19	68717749.5
High	P28293	8.858	15	5	5	5	261	29.1	10.56	11.09	Ctsg	31630030.25
High	Q9Z2K1	8.731	7	4	4	1	469	51.6	5.2	13.86	Krt16	2715207.25
High	P62908	7.705	25	4	4	4	243	26.7	9.66	10.51	Rps3	8778599.75
High	Q6NXH9	7.436	6	3	6	1	539	58.9	8.09	16.04	Krt73	138463872
High	P0DP27	7.388	11	1	3	1	149	16.8	4.22	7.98	Calm2	9798097.5
High	Q02257	7.379	4	2	2	2	745	81.7	6.14	7.53	Jup	5775342.25
High	Q3UV17	6.167	5	3	4	1	594	62.8	8.43	10.9	Krt76	381259040
High	P28798	6.143	4	2	2	2	589	63.4	6.8	6.54	Grn	7154448.375
High	P60867	4.74	9	1	2	1	119	13.4	9.94	8.55	Rps20	
High	P01630	4.54	21	2	2	2	113	12.5	8.65	6.75		20141335
High	Q8VED5	4.488	4	2	3	1	531	57.5	7.69	8.84	Krt79	136102576
High	P62984	4.352	13	1	2	1	128	14.7	9.83	8.14	Uba52	6558494
High	P14115	4.142	24	3	3	3	148	16.6	11.12	7.75	Rpl27a	54271206
High	P01837	3.983	18	2	2	2	107	11.9	5.9	3.9	Igkc	4374564.25
High	Q64475	3.925	21	2	2	2	126	13.9	10.32	5.98	H2bc3	5045448.875
High	P62242	3.853	12	2	2	2	208	24.2	10.32	4.35	Rps8	4486217.25
High	P01756	3.759	21	1	1	1	117	13	7.11	3.61		5512685.5
High	P47754	3.687	10	2	2	1	286	32.9	5.85	5.19	Capza2	3802424.25
High	P14131	3.337	14	2	2	2	146	16.4	10.21	4.9	Rps16	9784351.75
High	P47753	3.274	10	2	2	1	286	32.9	5.55	2.22	Capza1	10632873.5
High	P62082	3.182	7	1	1	1	194	22.1	10.1	3.34	Rps7	2289882.25
High	Q9Z204	3.14	4	1	2	1	313	34.4	5.05	5.23	Hnrnpc	21968489.5
High	Q9CXW4	3.066	8	1	1	1	178	20.2	9.6	3.75	Rpl11	8803884
High	Q922Q8	3	8	1	1	1	307	34.9	9.52	2.82	Lrrc59	
High	P62702	2.913	7	2	2	2	263	29.6	10.15	5.46	Rps4x	9669965.5
High	P32848	2.91	13	1	1	1	110	11.9	5.19	2.76	Pvalb	1178688.5
High	O08692	2.723	13	1	1	1	167	19.3	5.31	3.71	Ngp	3431246.5
High	P10852	2.707	5	2	2	2	526	58.3	5.91	4.64	Slc3a2	6131962.25
High	P62852	2.638	15	2	2	2	125	13.7	10.11	5.22	Rps25	32554860
High	Q9D154	2.524	3	1	1	1	379	42.5	6.21	2.75	Serpinb1a	3875435.5
High	P62849	2.342	9	1	1	1	133	15.4	10.78	2.93	Rps24	4050637.75
High	P17897	2.337	8	1	1	1	148	16.8	9.41	2.83	Lyz1	3315520
High	P47915	2.308	7	1	2	1	160	17.6	11.84	2.6	Rpl29	42015429
High	P97461	2.293	6	1	1	1	204	22.9	9.72	3.27		4983455.5
High	E9Q557	2.27	1	2	2	2	2883	332.7	6.8	5.17	Dsp	3791893.688
High	P63038	2.207	3	2	2	2	573	60.9	6.18	4.75	Hspd1	3642919.75
High	P27005	2.167	27	1	2	1	89	10.3	5.68	2.12	S100a8	7318233.75
High	P47963	1.888	5	1	1	1	211	24.3	11.55	2.29	Rpl13	1406362.125
High	Q64467	1.826	3	1	1	1	440	47.6	7.88	3.14	Gapdhs	9228514
High	P62918	1.82	4	1	1	1	257	28	11.03	2.84	Rpl8	3987580
High	P62806	1.801	10	1	1	1	103	11.4	11.36	2.33	H4c1; H4c11; H4c12; H4c14; H4c16; H4c2;	1838908.5
High	Q9WTR6	1.795	2	1	1	1	502	55.4	9.23	2.72	Slc7a11	1448450.125
High	Q9CR57	1.786	6	1	1	1	217	23.5	11.02	3.14	Rpl14	1675620.625
High	Q3UND0	1.77	3	1	1	1	358	40.7	4.7	2.46	Skap2	3241254
High	P07356	1.72	4	1	1	1	339	38.7	7.69	2.28	Anxa2	704561.25
High	P62264	1.571	7	1	1	1	151	16.3	10.05	2.57	Rps14	1748175.5
High	Q64523	1.552	7	1	1	1	129	14	10.9	2.25	H2ac20	23908008
High	P48962	1.481	3	1	1	1	298	32.9	9.72	0	Slc25a4	2042478.25
High	P11247	1.404	1	1	1	1	718	81.1	9.55	1.94	Mpo	1722934.875
High	P67984	1.378	10	1	1	1	128	14.8	9.19	2.57	Rpl22	4752834.5
High	P59999	1.354	7	1	1	1	168	19.7	8.43	2.47	Arpc4	2405658
High	P56135	1.337	14	1	1	1	88	10.3	9.95	2.45	Atp5mf	5223939.5
High	Q99P91	1.286	3	1	1	1	574	63.6	7.61	3.24	Gpnmb	1974111.75
High	P63276	1.245	7	1	1	1	135	15.5	9.85	2.68	Rps17	5685126.5
High	P30681	1.186	8	1	1	1	210	24.1	7.31	2.66	Hmgb2	
High	P63158	1.174	7	1	1	1	215	24.9	5.74	0	Hmgb1	
High	Q8R0X7	1.173	2	1	1	1	568	63.6	9.1	2.09	Sgpl1	727706.25
High	Q9WV32	1.162	3	1	1	1	372	41	8.35	2.2	Arpc1b	1380339.5
High	P97351	1.154	3	1	1	1	264	29.9	9.73	1.71	Rps3a	2333632
High	P10126	1.121	5	1	1	1	462	50.1	9.01	2.15	Eef1a1	1394907.625
High	P86048	1.11	4	1	1	1	214	24.5	10.11	0	Rpl10l	
High	P35980	1.078	5	1	1	1	188	21.6	11.78	0	Rpl18	1424777
High	P62830	1.077	6	1	1	1	140	14.9	10.51	0	Rpl23	1465805.25
High	P61358	1.072	6	1	1	1	136	15.8	10.56	1.93	Rpl27	4747578

AppendixTable S3: Histologic score of lung tumors

**Histologic criteria of tumors**		**Score**
**Tumor Cell Morphology**	**•Nuclear: Uniform size/shape, pale chromatin**	0
	**•Cytoplasmic: Abundant eosinophilic staining**	
	**•Mitosis: ≤1**	
	**Nuclear: Mild enlargement (1.5× normal)**	1
	**•Cytoplasmic: Focal vacuolization**	
	**•Mitosis: 2-3**	
	**•Nuclear: Moderate pleomorphism (>2× variation)**	2
	**•Cytoplasmic: Diffuse basophilia**	
	**•Mitosis: 4-5**	
	**• Nuclear: Marked atypia (giant/multinucleated cells)**	3
	**•Cytoplasmic: Scant cytoplasm (naked nuclei)**	
	**•Mitosis: ≥6**	
**Tumor Necrosis**	**<5%**	0
	**1%-15%**	1
	**16%-30%**	2
	**>30%**	3
**Infiltration of inflammatory cells**	**absent:<10**	0
	**mild:10-50**	1
	**moderate:50-100**	2
	**severe:>100**	3
**Invasion**	**absent**	0
	**present**	1

AppendixTable S4: Characteristics of clinical patients with Lung cancer for FISH analysis

**Characteristic**	**Category**	**Number of Patients(n=15)**
**Ages**	≤55	4
	>55	11
**Sex**	Female	6
	male	9
**Primary Tumor Location**	Right lung upper lobe	5
	Right lung middle lobe	2
	Right lung lower lobe	2
	Left lung upper lobe	4
	Left lung lower lobe	2
**Clinical Stage**	I	3
	II	5
	III	3
	IV	4
**Lymph Node Metastasis**	No	10
	Yes	5
**Total**		**15**
