# Supplementary figures and images for: Aspergillus fumigatus promotes tumor angiogenesis via SLC7A11 on myeloid-derived suppressor cells (part 1 of 2)
Source: EMBO Rep. 2025 Nov 17;26(24):6266–91. doi: 10.1038/s44319-025-00627-x (PMC12715260; doi:10.1038/s44319-025-00627-x)

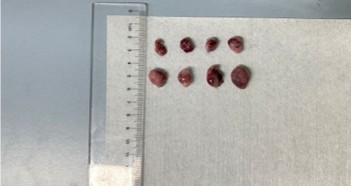

Supplement: Supplementary file 3 — Source data Fig. 1 [file 44319_2025_627_MOESM3_ESM.zip › Figure 1/1C/Tumor.jpg]

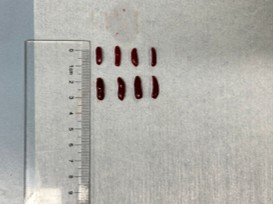

Supplement: Supplementary file 3 — Source data Fig. 1 [file 44319_2025_627_MOESM3_ESM.zip › Figure 1/1E/Spleen.jpg]

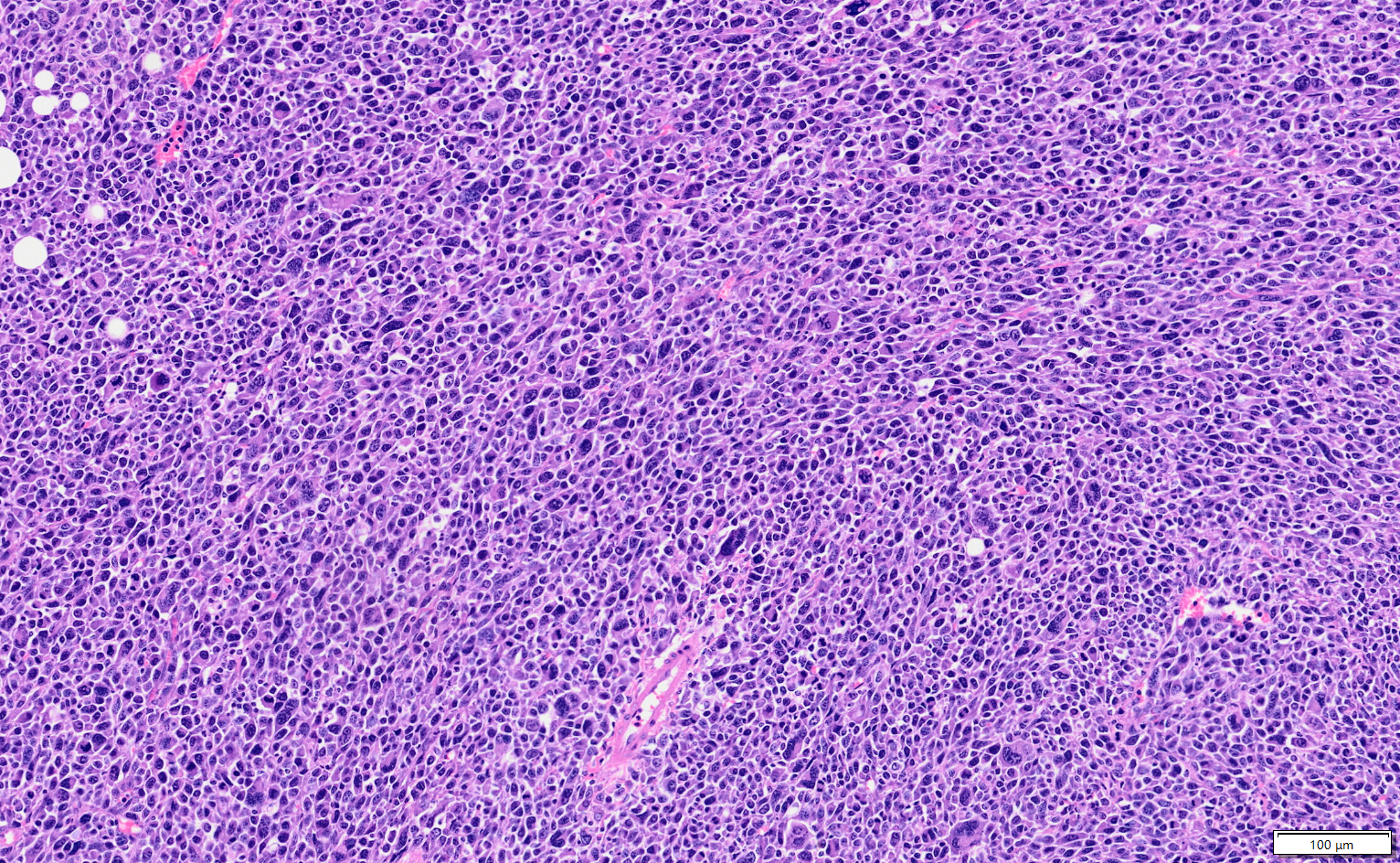

Supplement: Supplementary file 3 — Source data Fig. 1 [file 44319_2025_627_MOESM3_ESM.zip › Figure 1/1G/LLC A.f HE.png]

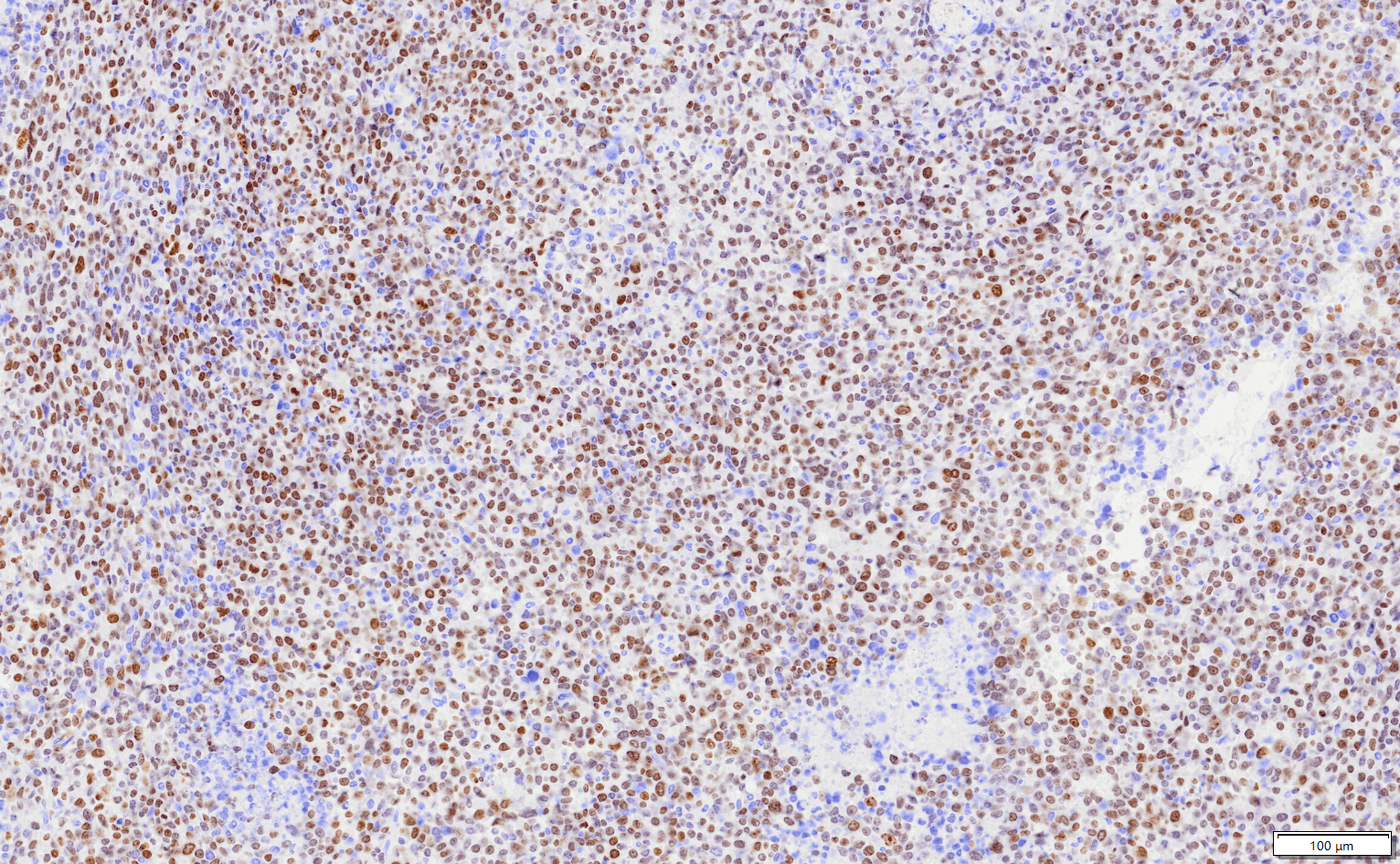

Supplement: Supplementary file 3 — Source data Fig. 1 [file 44319_2025_627_MOESM3_ESM.zip › Figure 1/1G/LLC A.f ki67.png]

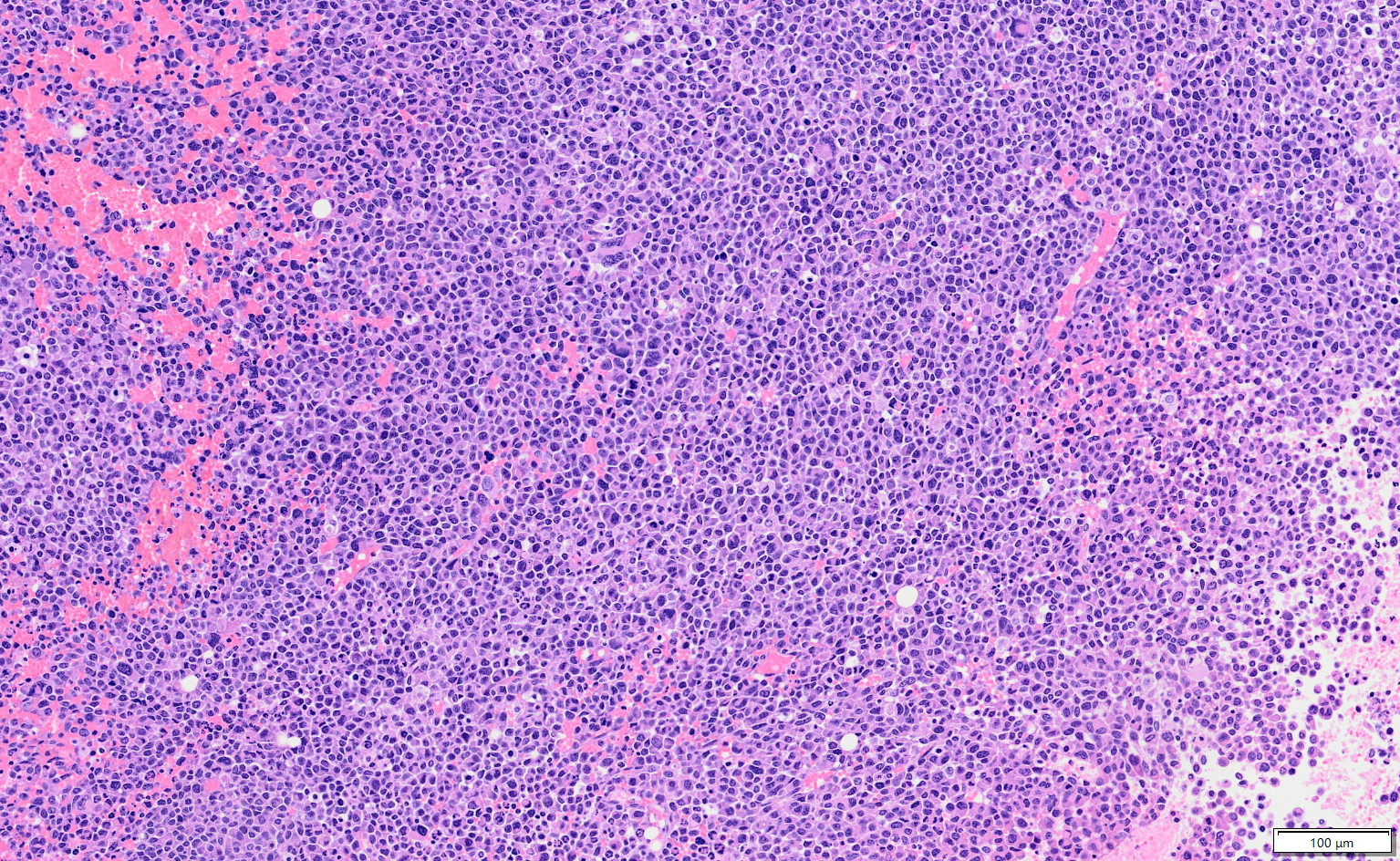

Supplement: Supplementary file 3 — Source data Fig. 1 [file 44319_2025_627_MOESM3_ESM.zip › Figure 1/1G/LLC Ctrl HE.png]

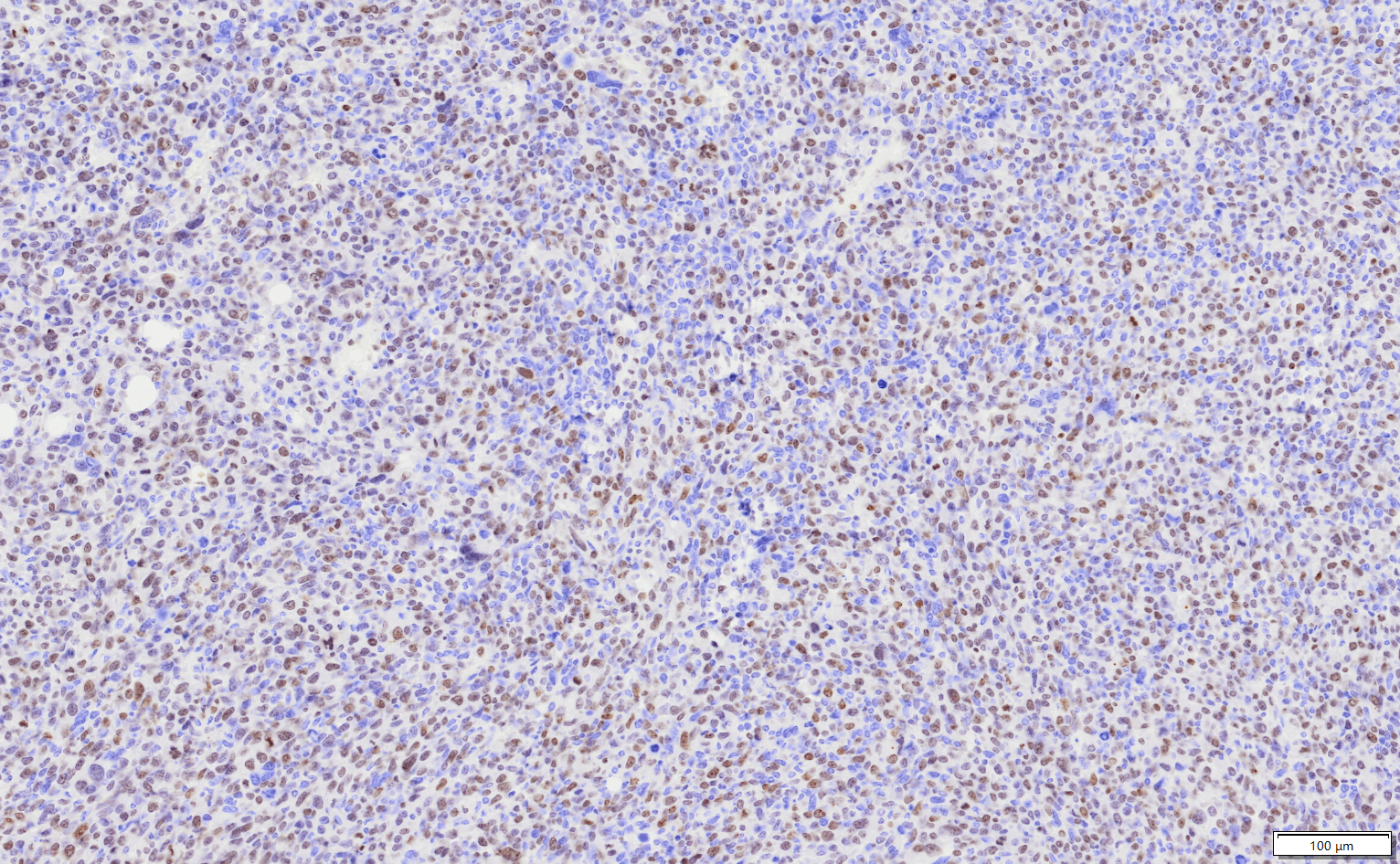

Supplement: Supplementary file 3 — Source data Fig. 1 [file 44319_2025_627_MOESM3_ESM.zip › Figure 1/1G/LLC Ctrl ki67.png]

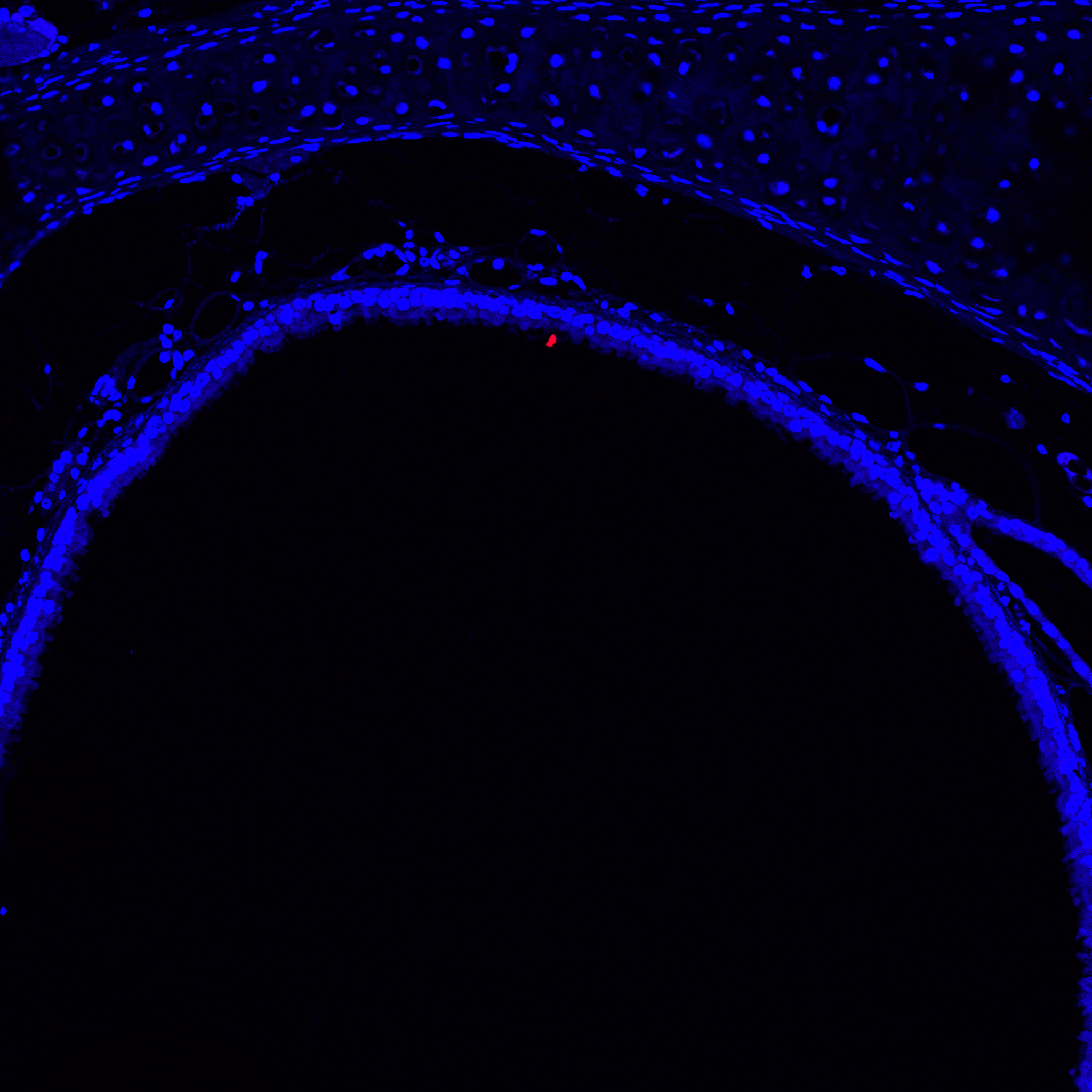

Supplement: Supplementary file 3 — Source data Fig. 1 [file 44319_2025_627_MOESM3_ESM.zip › Figure 1/1H/Bronchi LLC A.f.png]

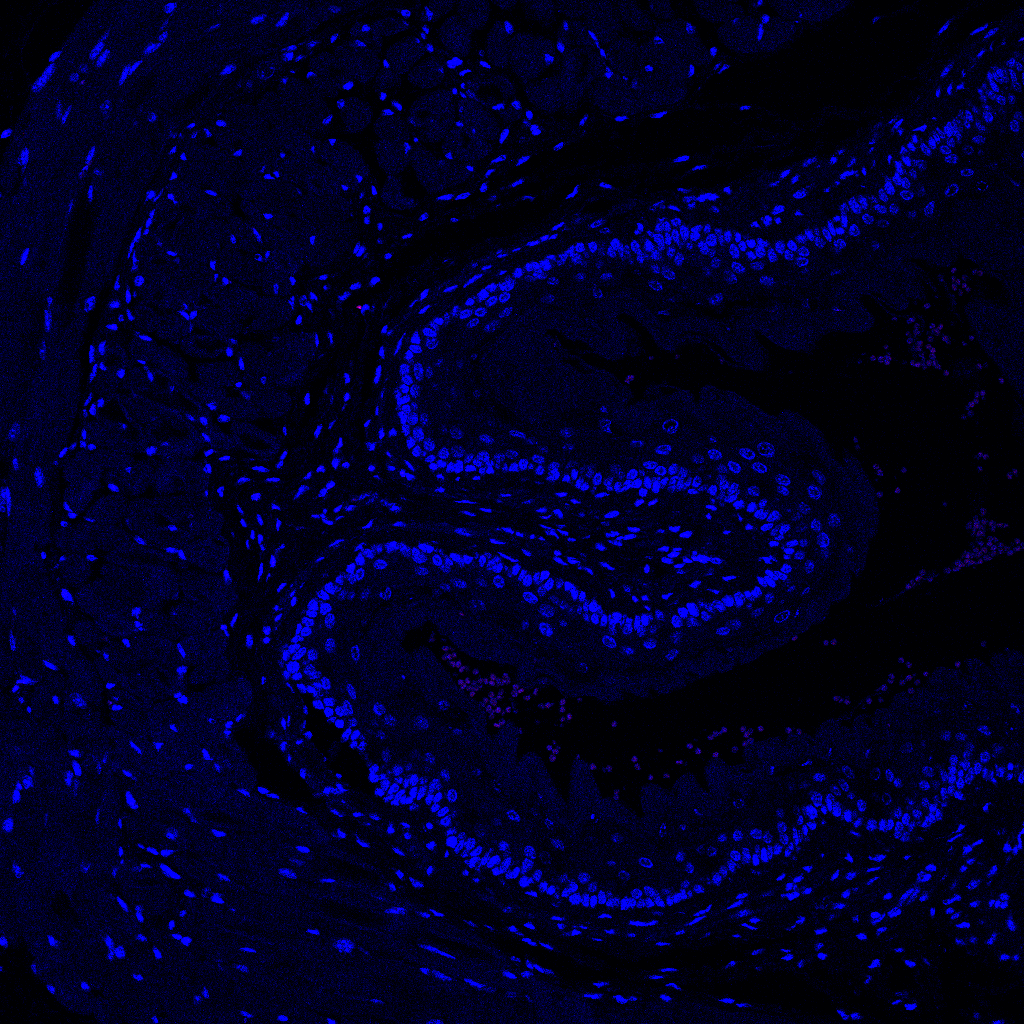

Supplement: Supplementary file 3 — Source data Fig. 1 [file 44319_2025_627_MOESM3_ESM.zip › Figure 1/1H/Bronchi LLC Ctrl.tif]

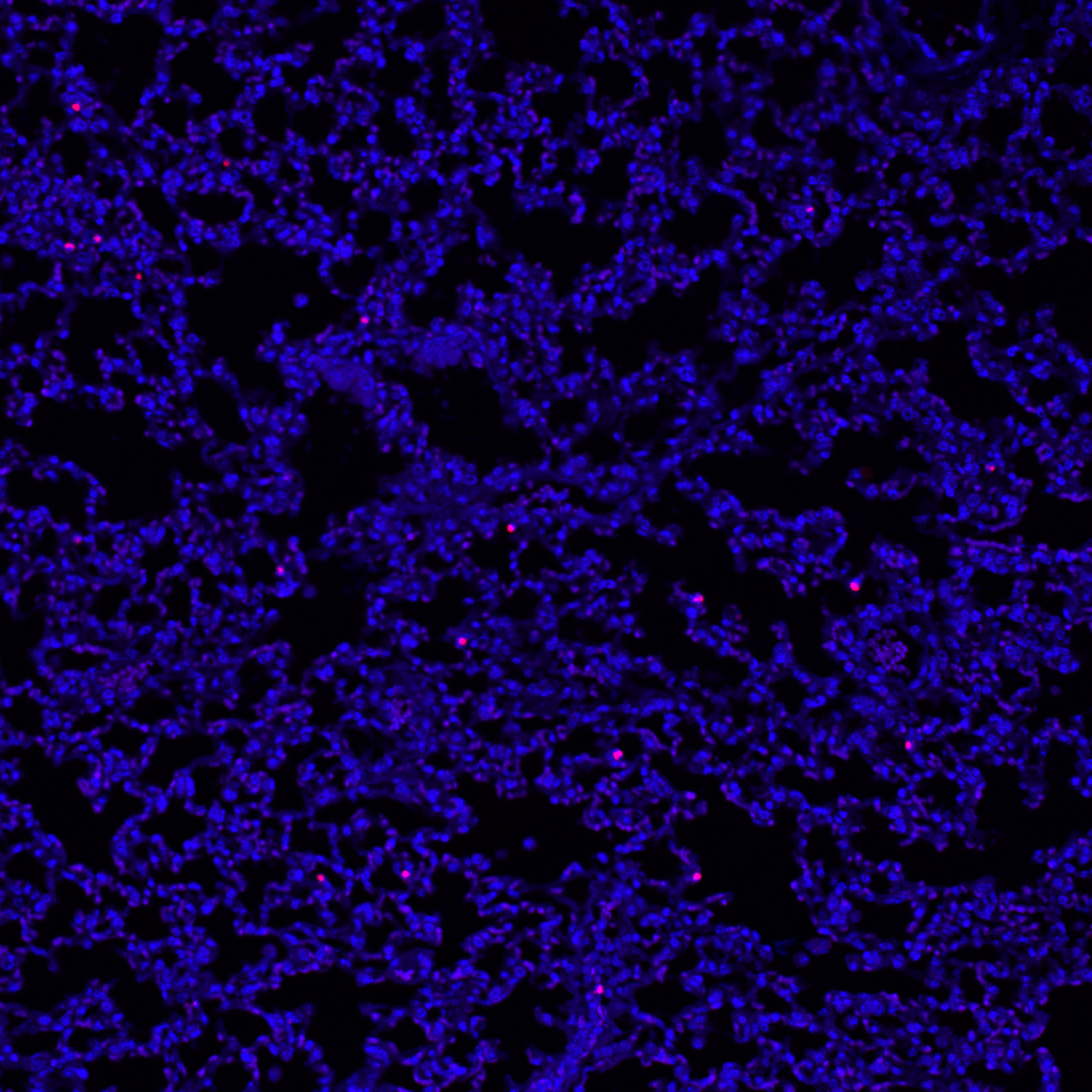

Supplement: Supplementary file 3 — Source data Fig. 1 [file 44319_2025_627_MOESM3_ESM.zip › Figure 1/1H/Lung LLC A.f.png]

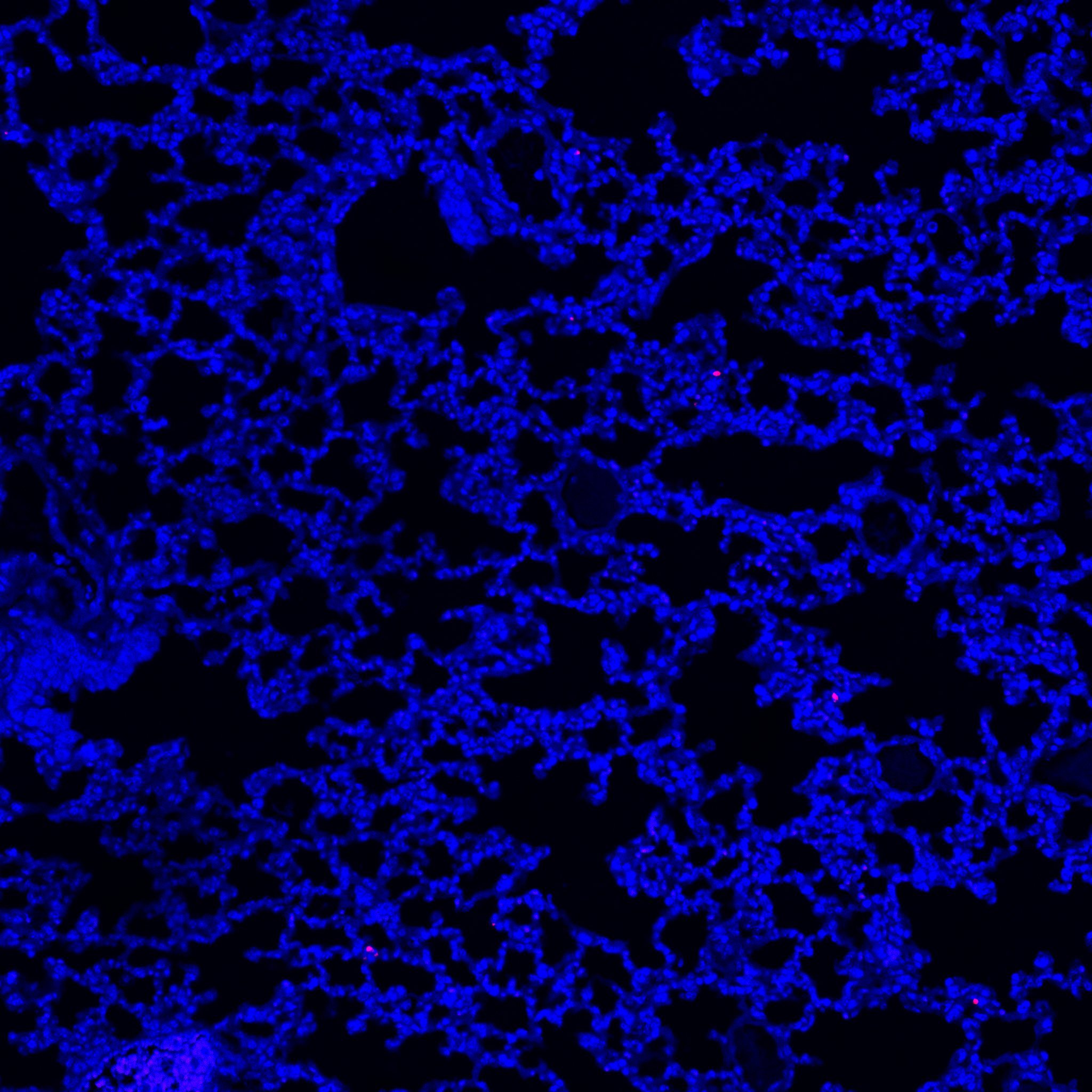

Supplement: Supplementary file 3 — Source data Fig. 1 [file 44319_2025_627_MOESM3_ESM.zip › Figure 1/1H/Lung LLC Ctrl.png]

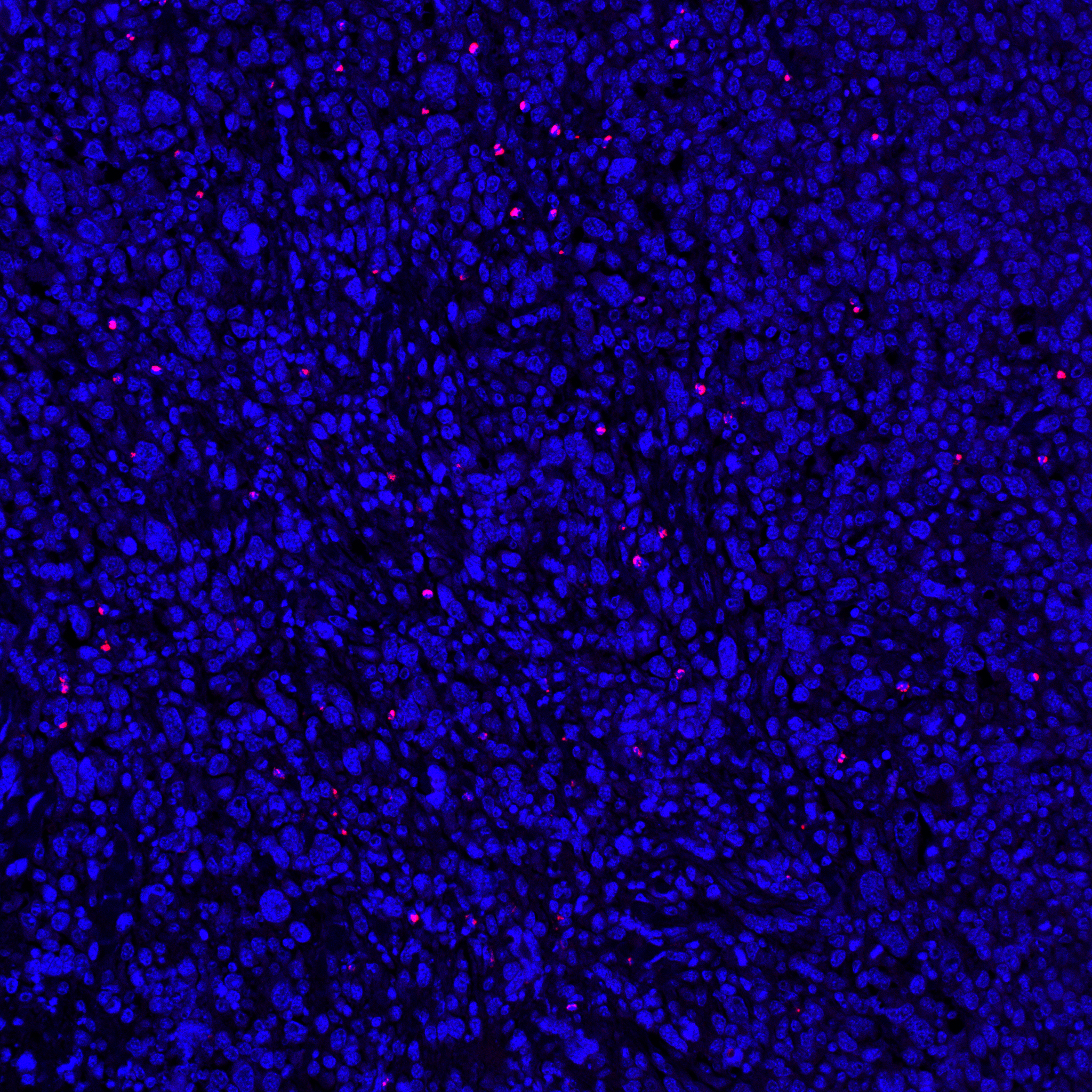

Supplement: Supplementary file 3 — Source data Fig. 1 [file 44319_2025_627_MOESM3_ESM.zip › Figure 1/1H/Tumor LLC A.f.png]

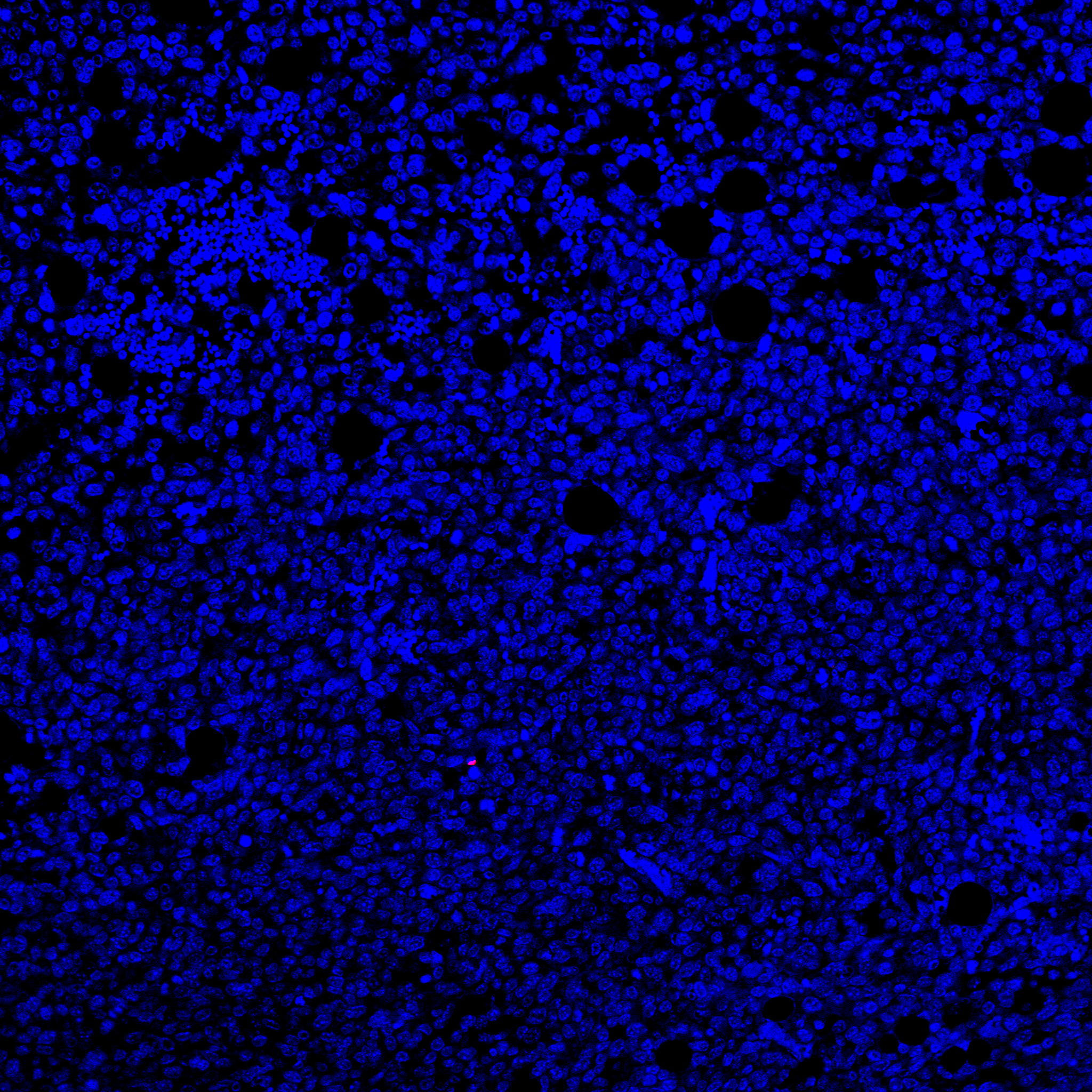

Supplement: Supplementary file 3 — Source data Fig. 1 [file 44319_2025_627_MOESM3_ESM.zip › Figure 1/1H/Tumor LLC Ctrl.png]

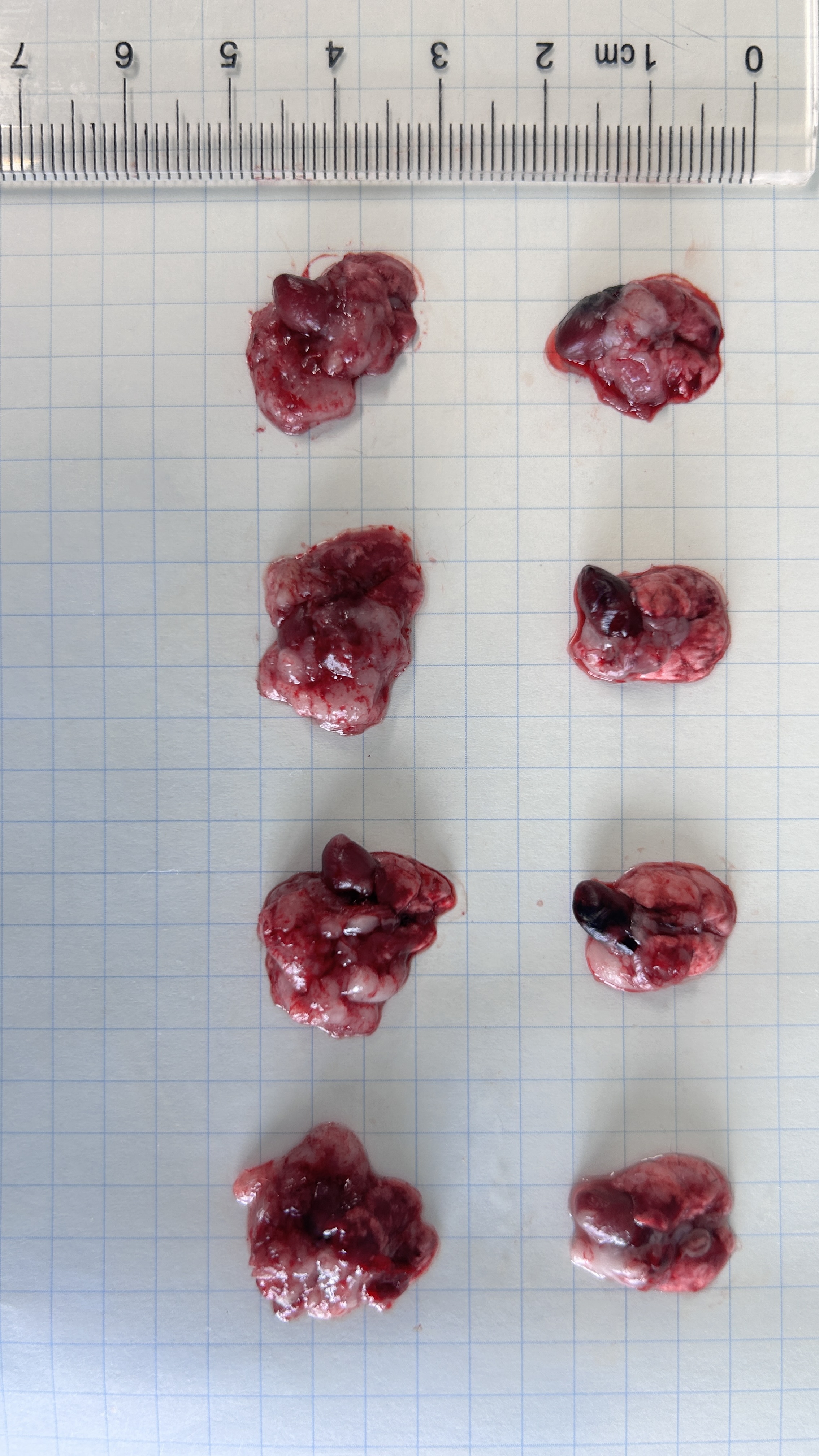

Supplement: Supplementary file 3 — Source data Fig. 1 [file 44319_2025_627_MOESM3_ESM.zip › Figure 1/1J/Tumor.jpg]

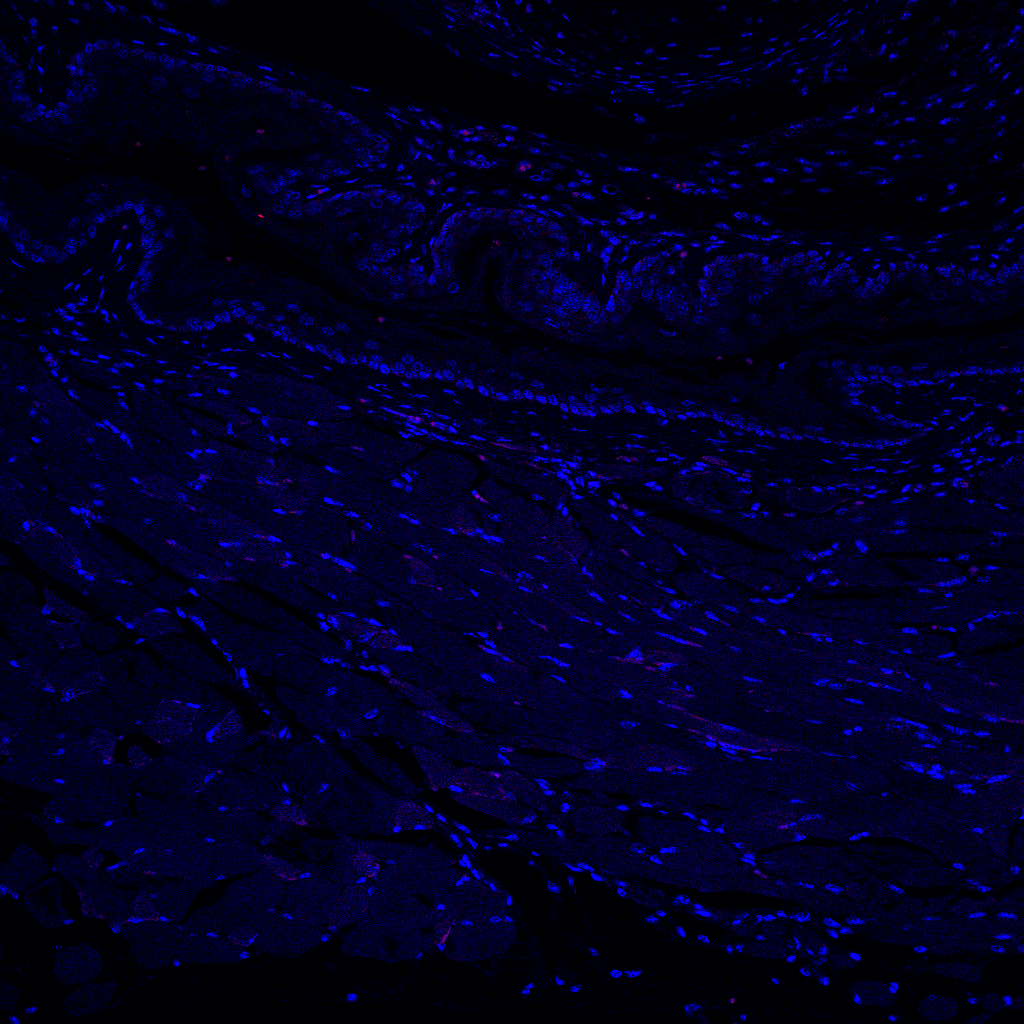

Supplement: Supplementary file 3 — Source data Fig. 1 [file 44319_2025_627_MOESM3_ESM.zip › Figure 1/1O/Bronchi Luad A.f.png]

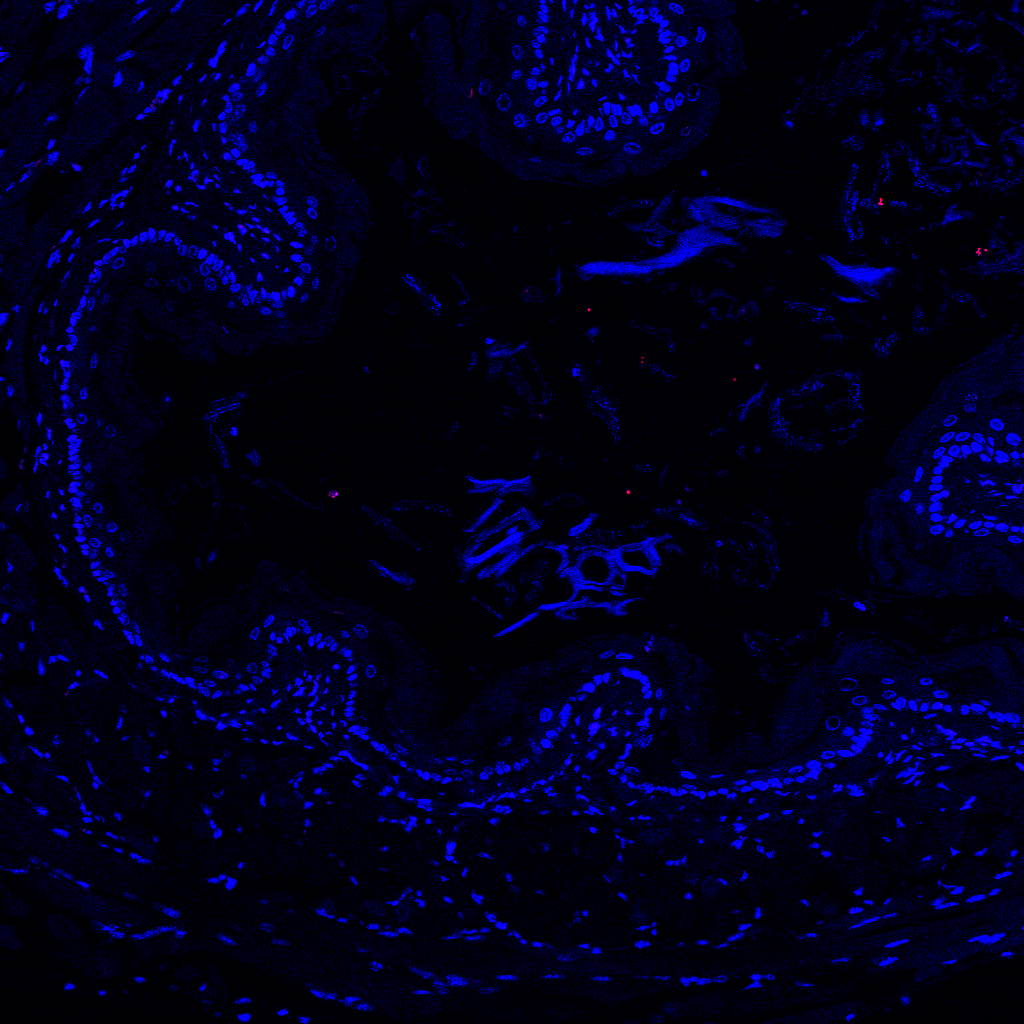

Supplement: Supplementary file 3 — Source data Fig. 1 [file 44319_2025_627_MOESM3_ESM.zip › Figure 1/1O/Bronchi Luad Ctrl.tif]

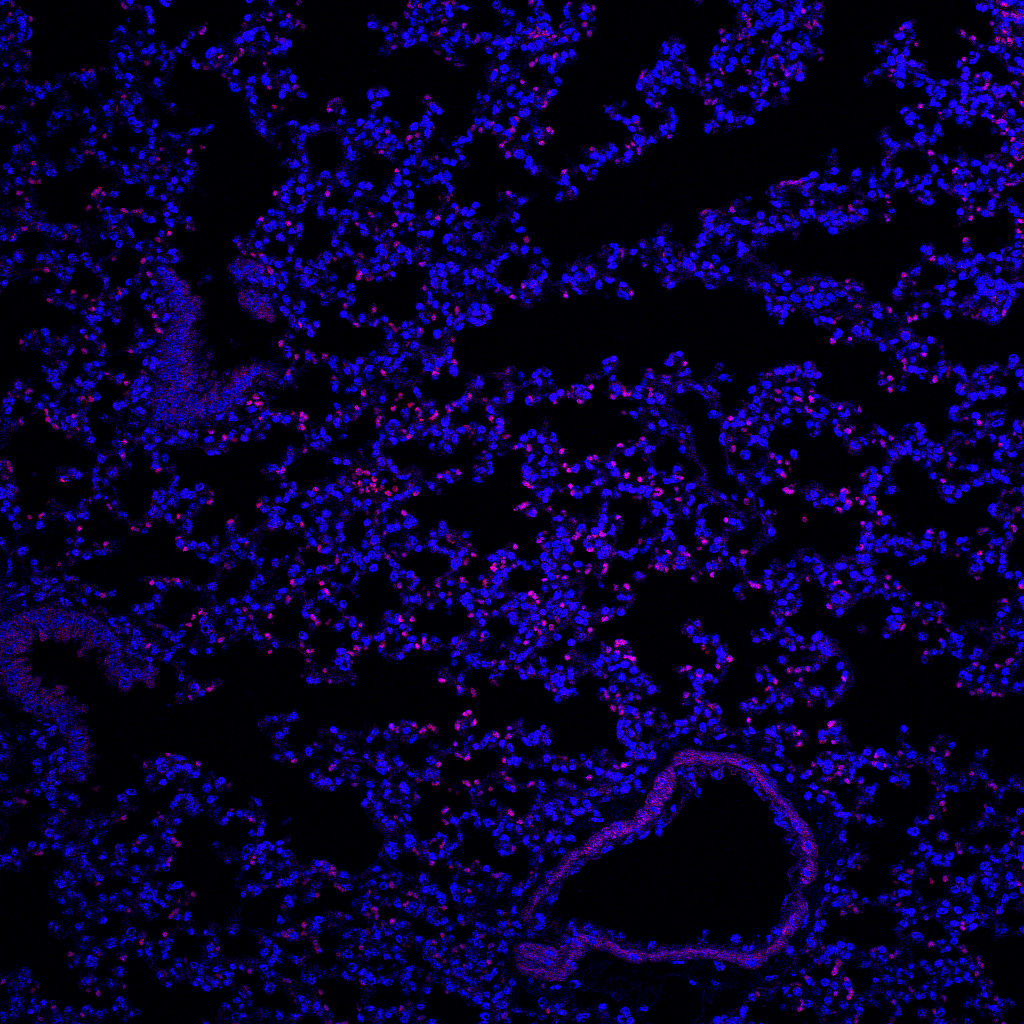

Supplement: Supplementary file 3 — Source data Fig. 1 [file 44319_2025_627_MOESM3_ESM.zip › Figure 1/1O/Lung Luad A.f.tif]

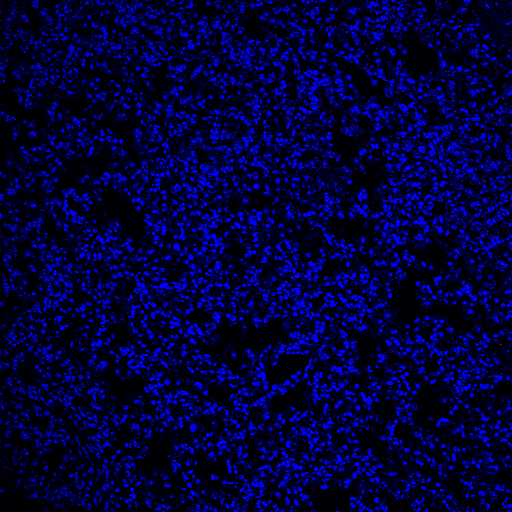

Supplement: Supplementary file 3 — Source data Fig. 1 [file 44319_2025_627_MOESM3_ESM.zip › Figure 1/1O/Lung Luad Ctrl.tif]

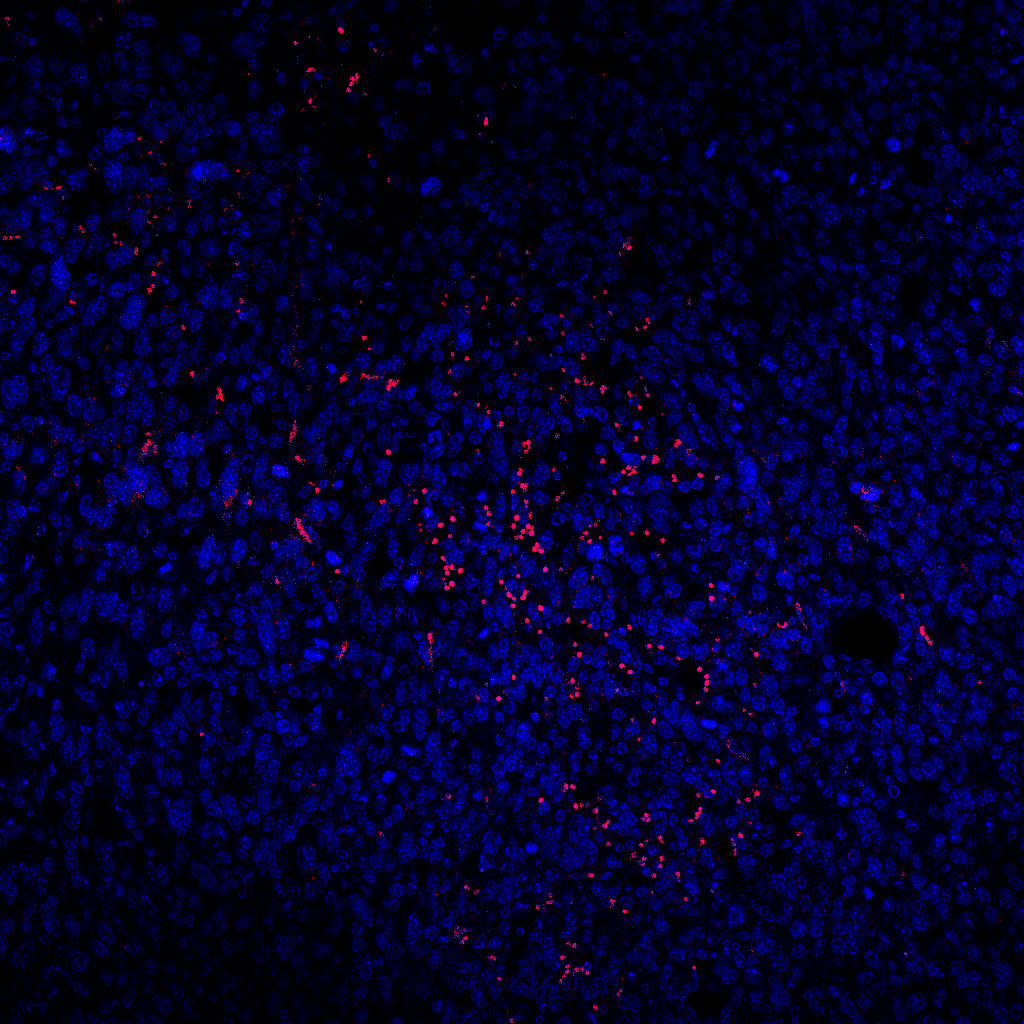

Supplement: Supplementary file 3 — Source data Fig. 1 [file 44319_2025_627_MOESM3_ESM.zip › Figure 1/1O/Tumor Luad A.f.png]

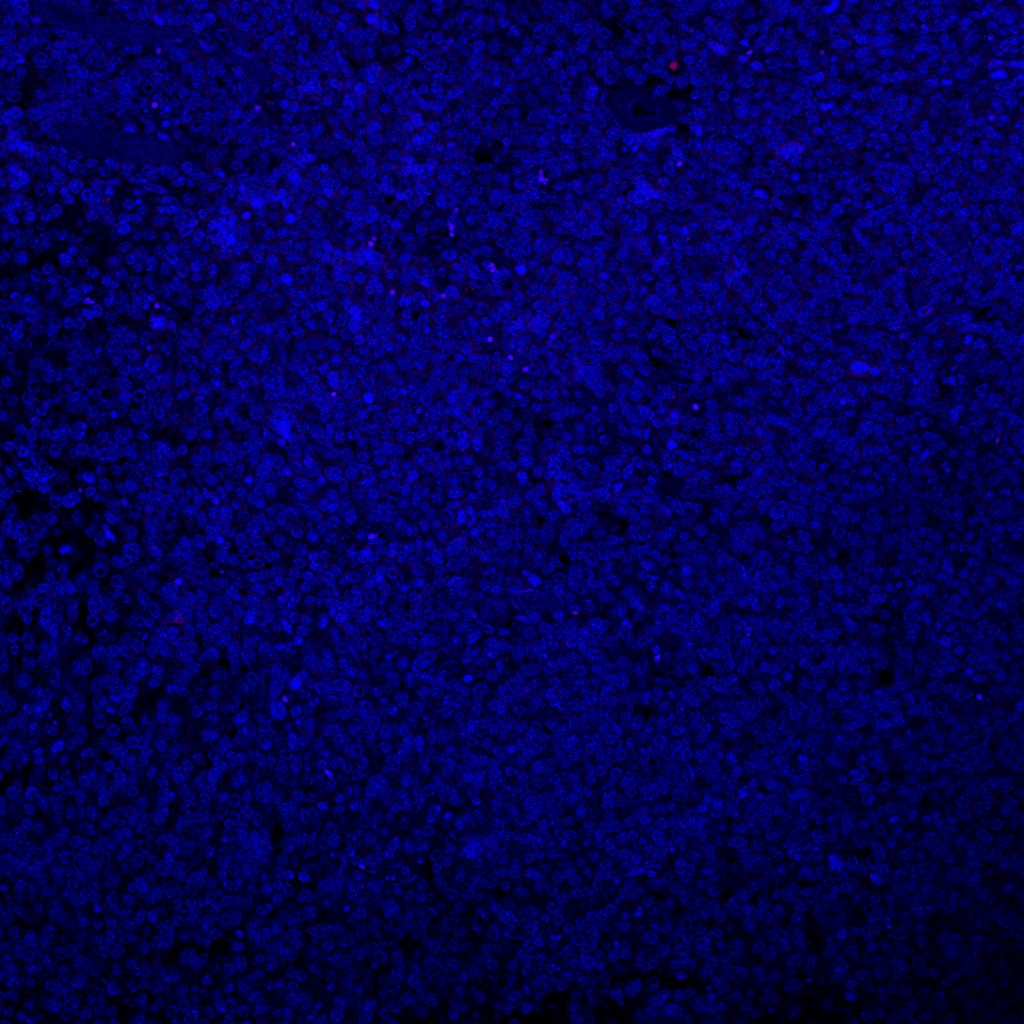

Supplement: Supplementary file 3 — Source data Fig. 1 [file 44319_2025_627_MOESM3_ESM.zip › Figure 1/1O/Tumor Luad Ctrl.tif]

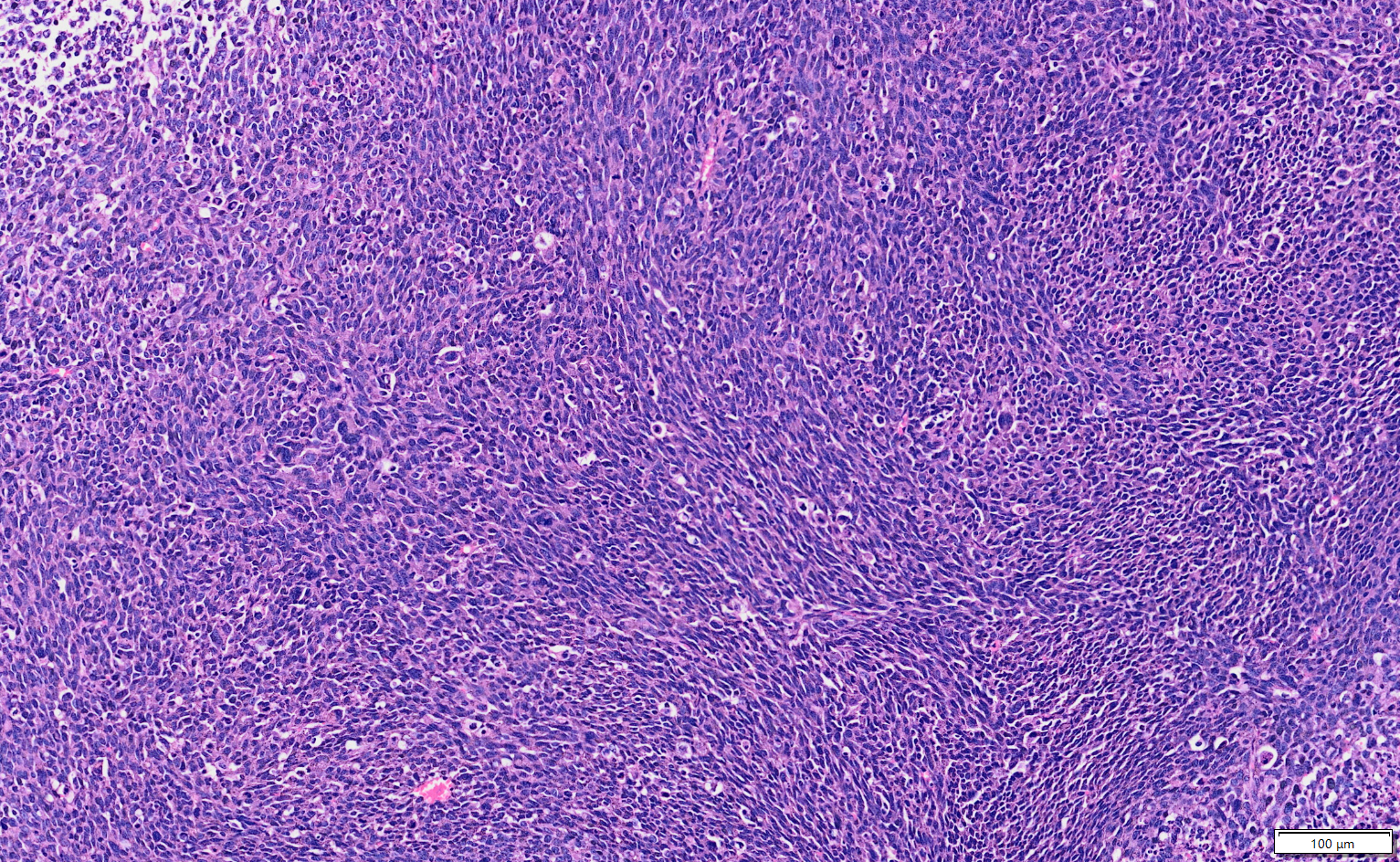

Supplement: Supplementary file 3 — Source data Fig. 1 [file 44319_2025_627_MOESM3_ESM.zip › Figure 1/1N/LUAD A.f HE.png]

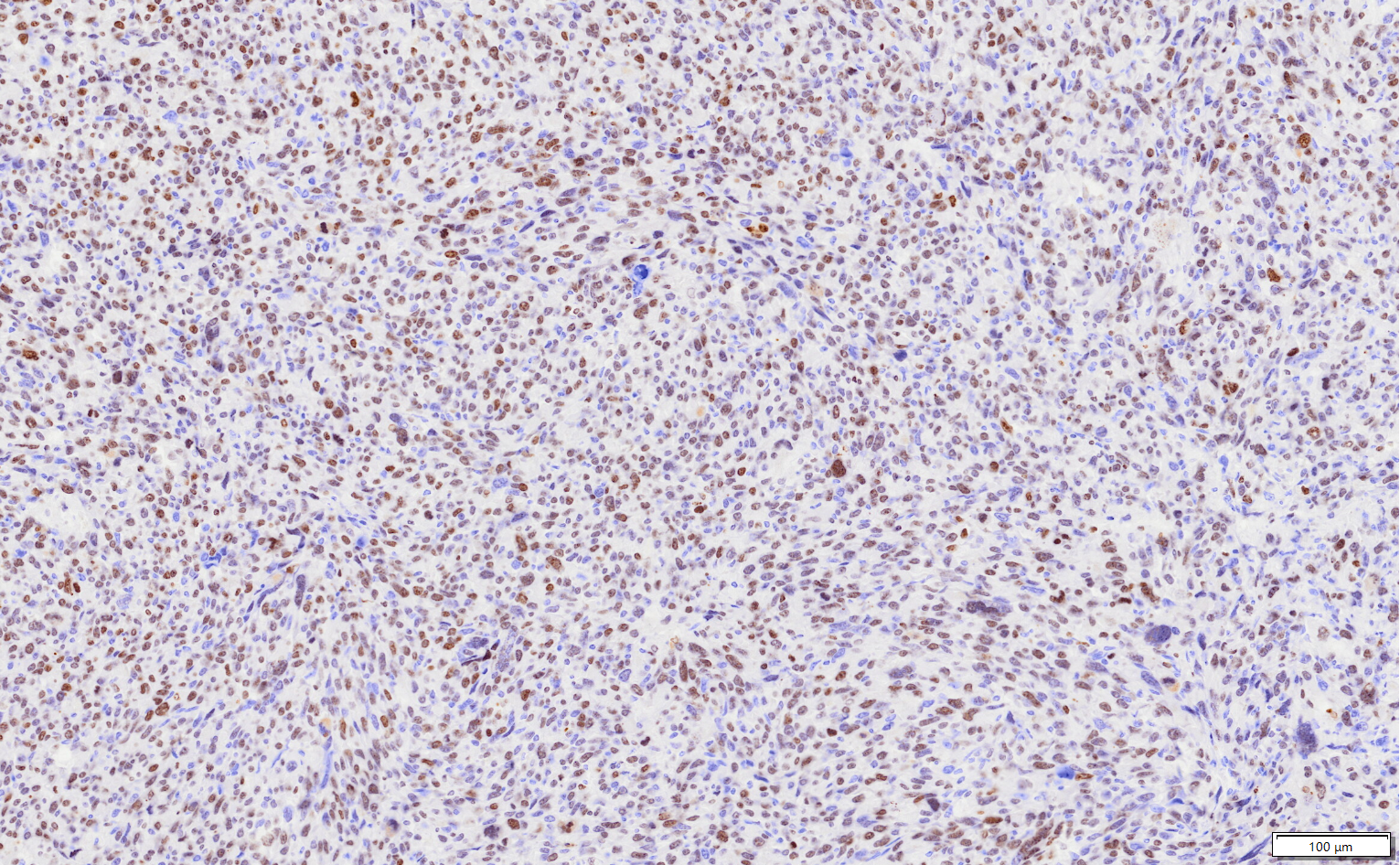

Supplement: Supplementary file 3 — Source data Fig. 1 [file 44319_2025_627_MOESM3_ESM.zip › Figure 1/1N/LUAD A.f Ki67.png]

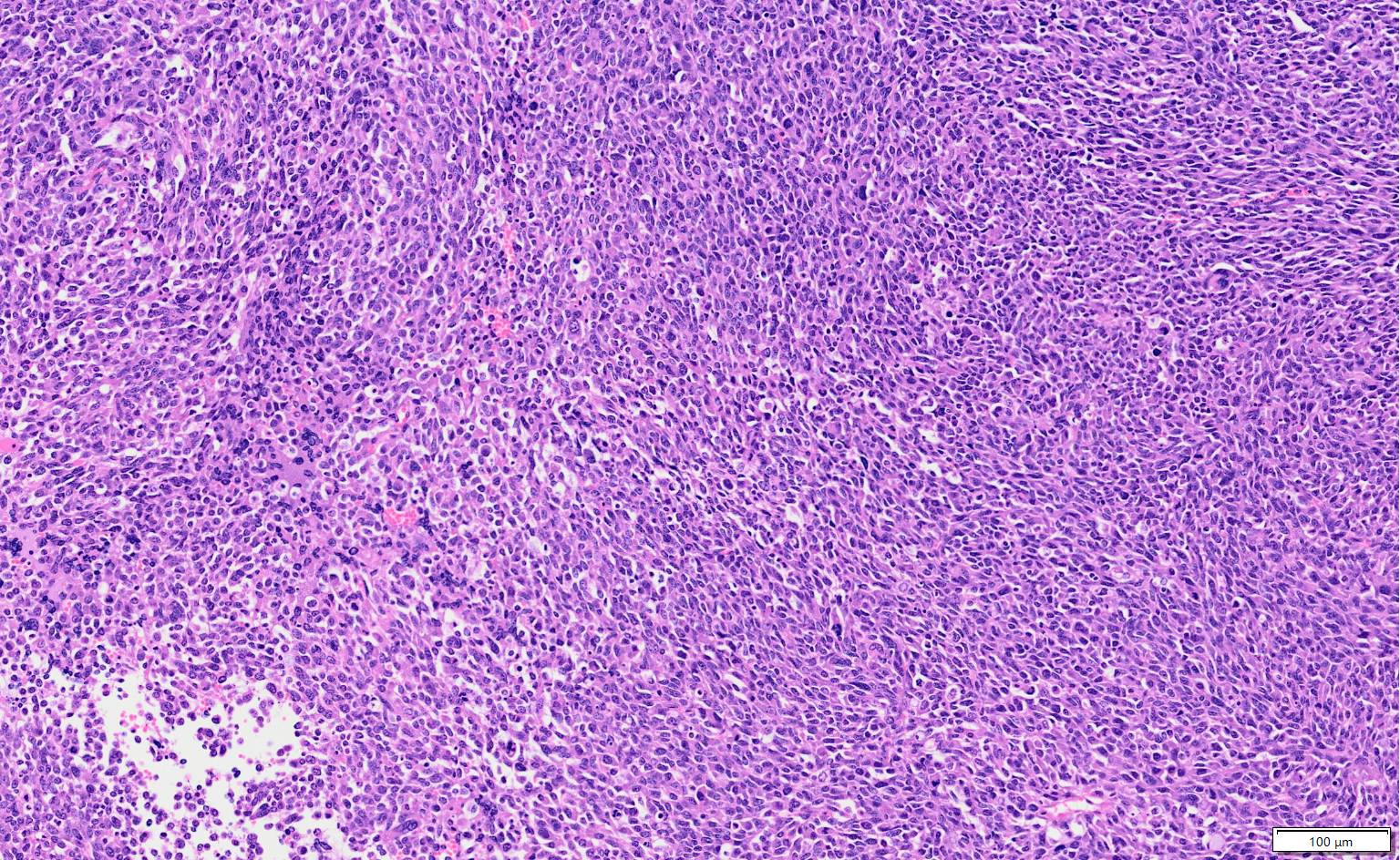

Supplement: Supplementary file 3 — Source data Fig. 1 [file 44319_2025_627_MOESM3_ESM.zip › Figure 1/1N/LUAD Ctrl HE.png]

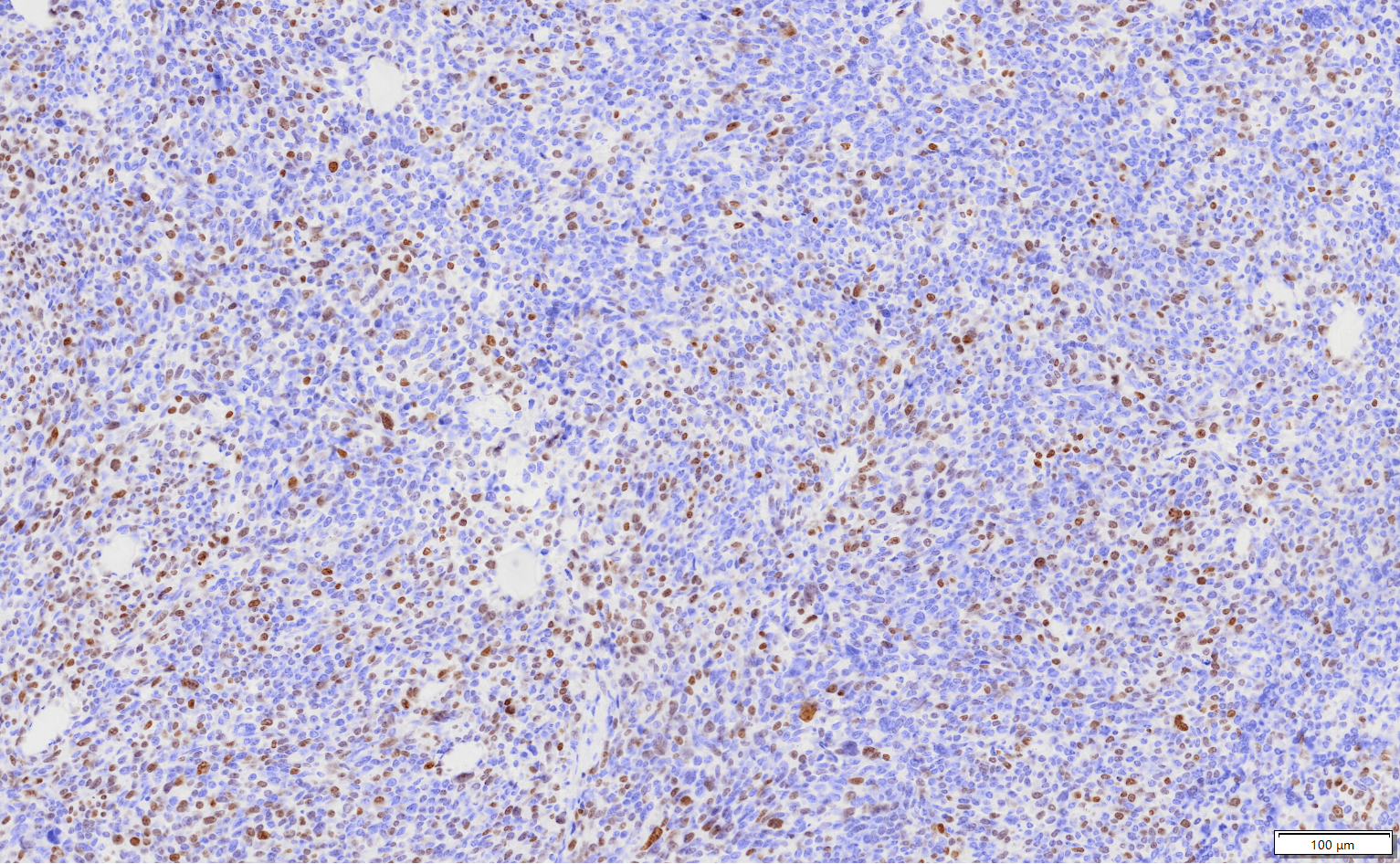

Supplement: Supplementary file 3 — Source data Fig. 1 [file 44319_2025_627_MOESM3_ESM.zip › Figure 1/1N/LUAD Ctrl Ki67.png]

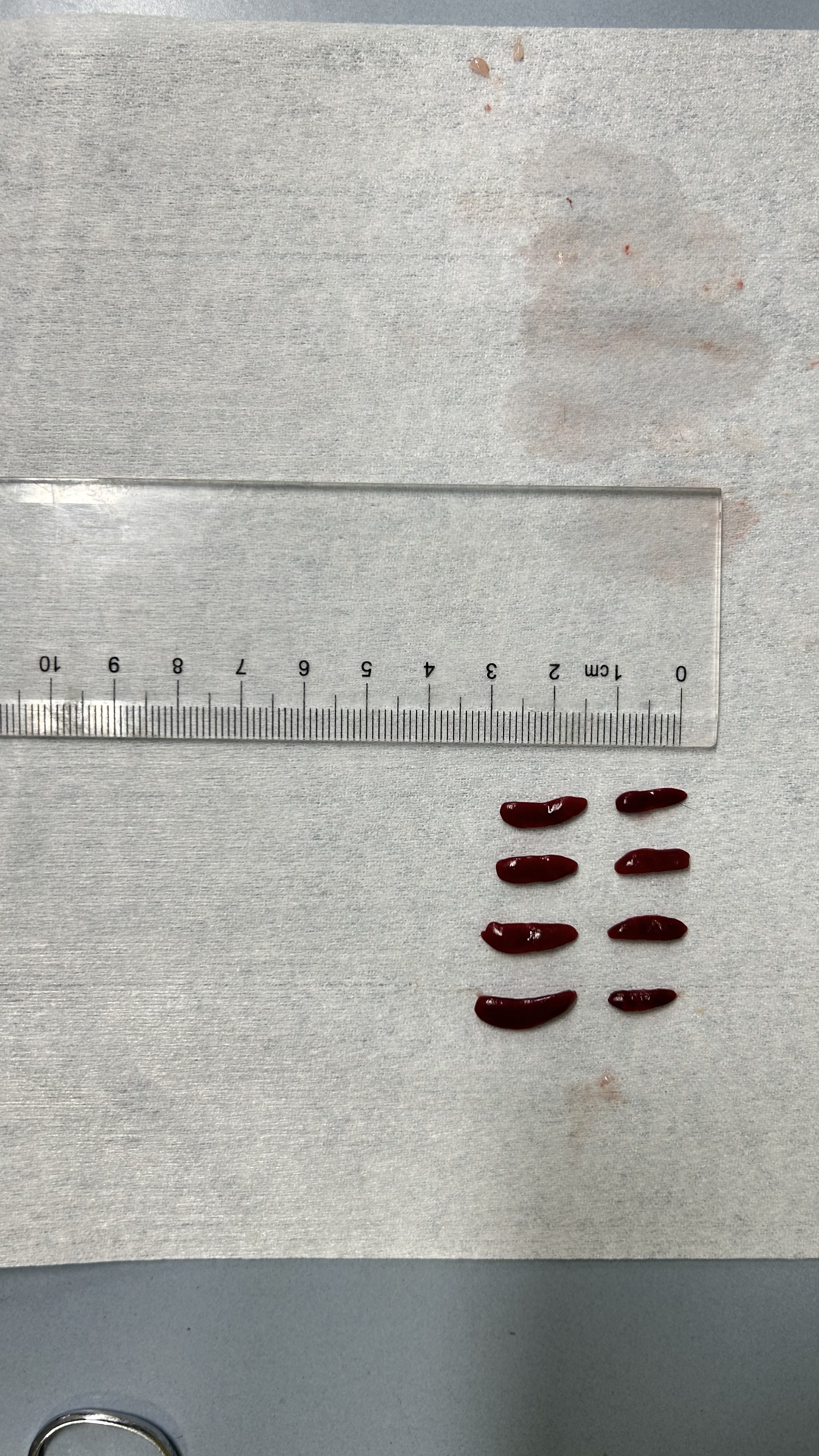

Supplement: Supplementary file 3 — Source data Fig. 1 [file 44319_2025_627_MOESM3_ESM.zip › Figure 1/1L/Spleen.jpg]

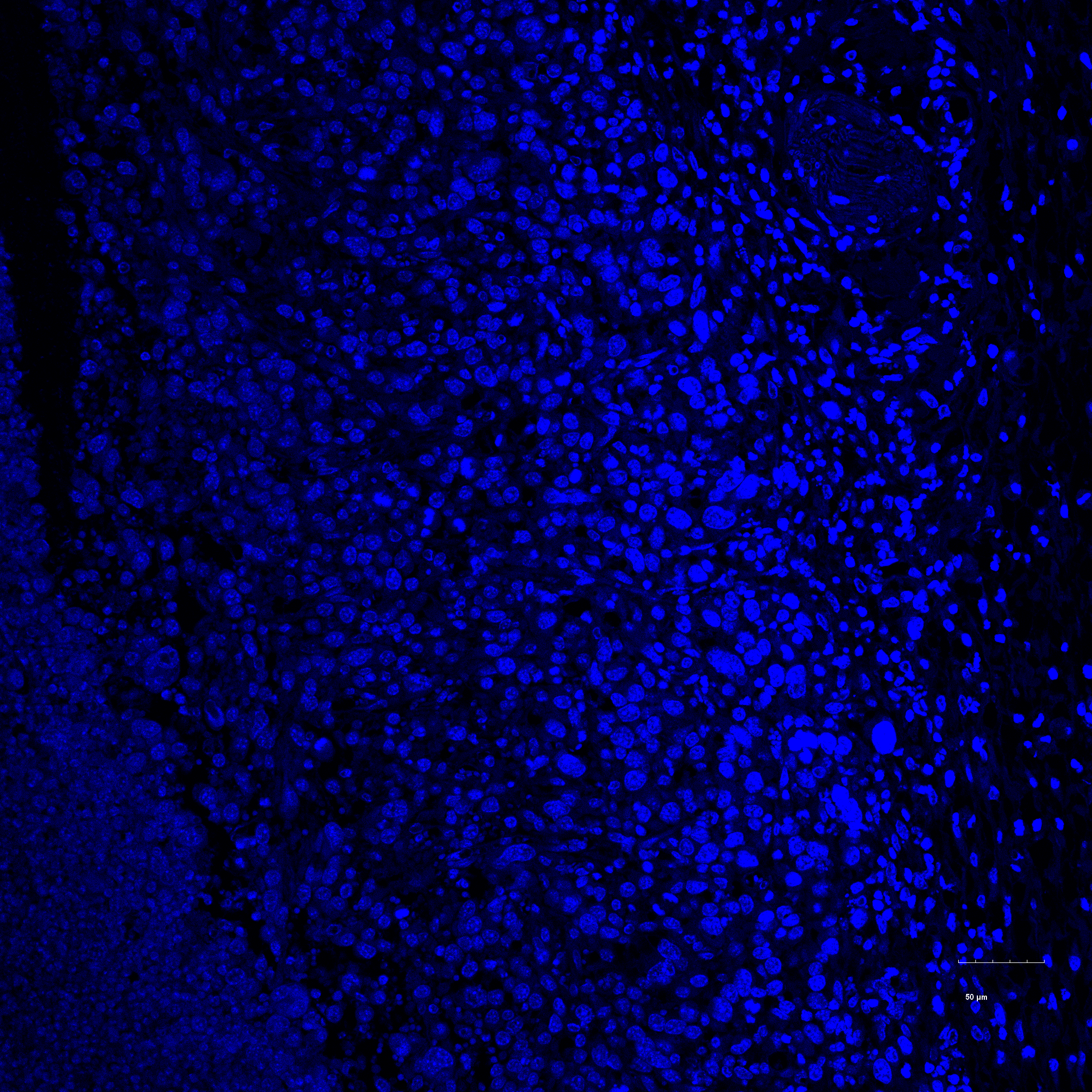

Supplement: Supplementary file 4 — Source data Fig. 2 [file 44319_2025_627_MOESM4_ESM.zip › Figure 2/2B/CD34 DAPI LLC A.f.png]

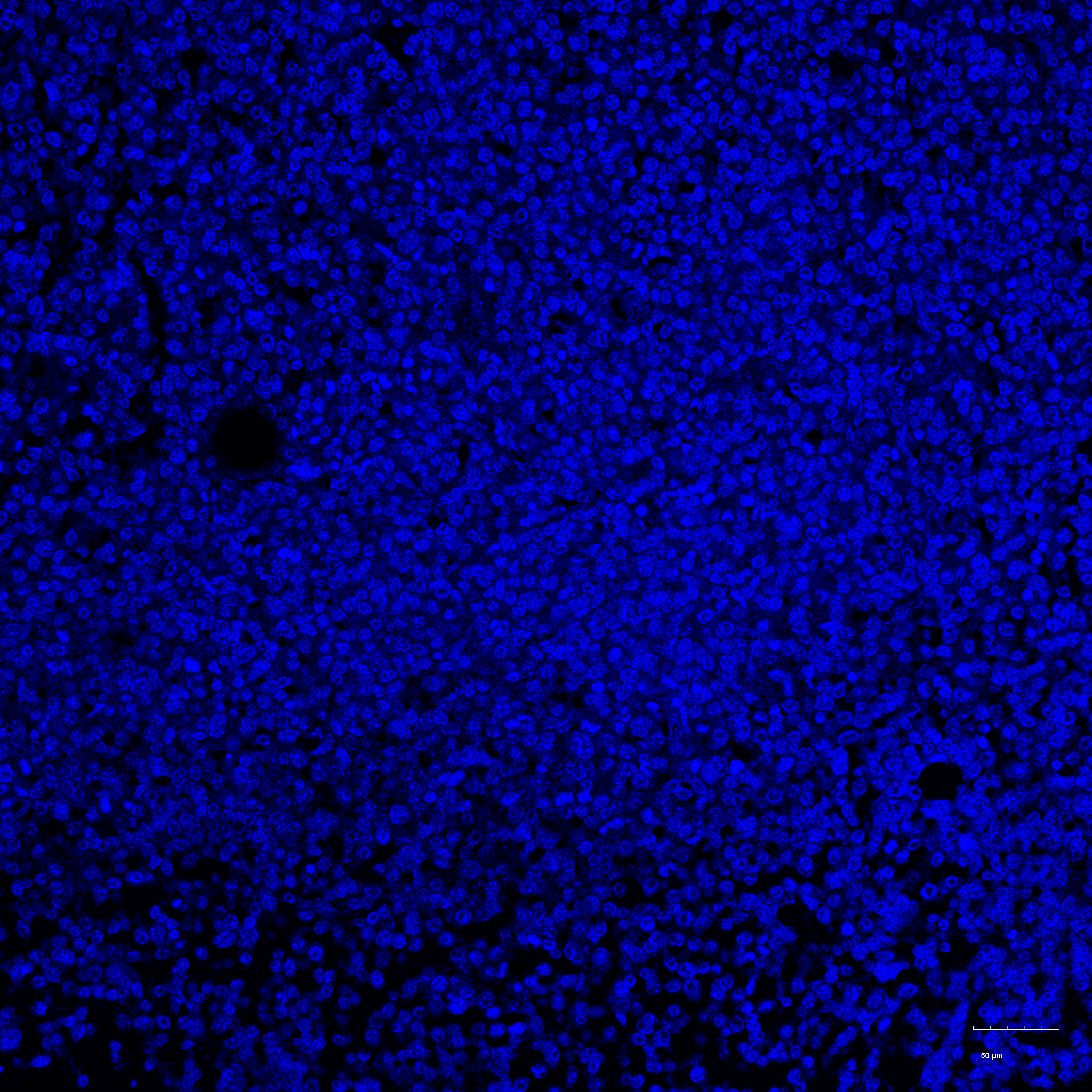

Supplement: Supplementary file 4 — Source data Fig. 2 [file 44319_2025_627_MOESM4_ESM.zip › Figure 2/2B/CD34 DAPI LLC Ctrl.tif]

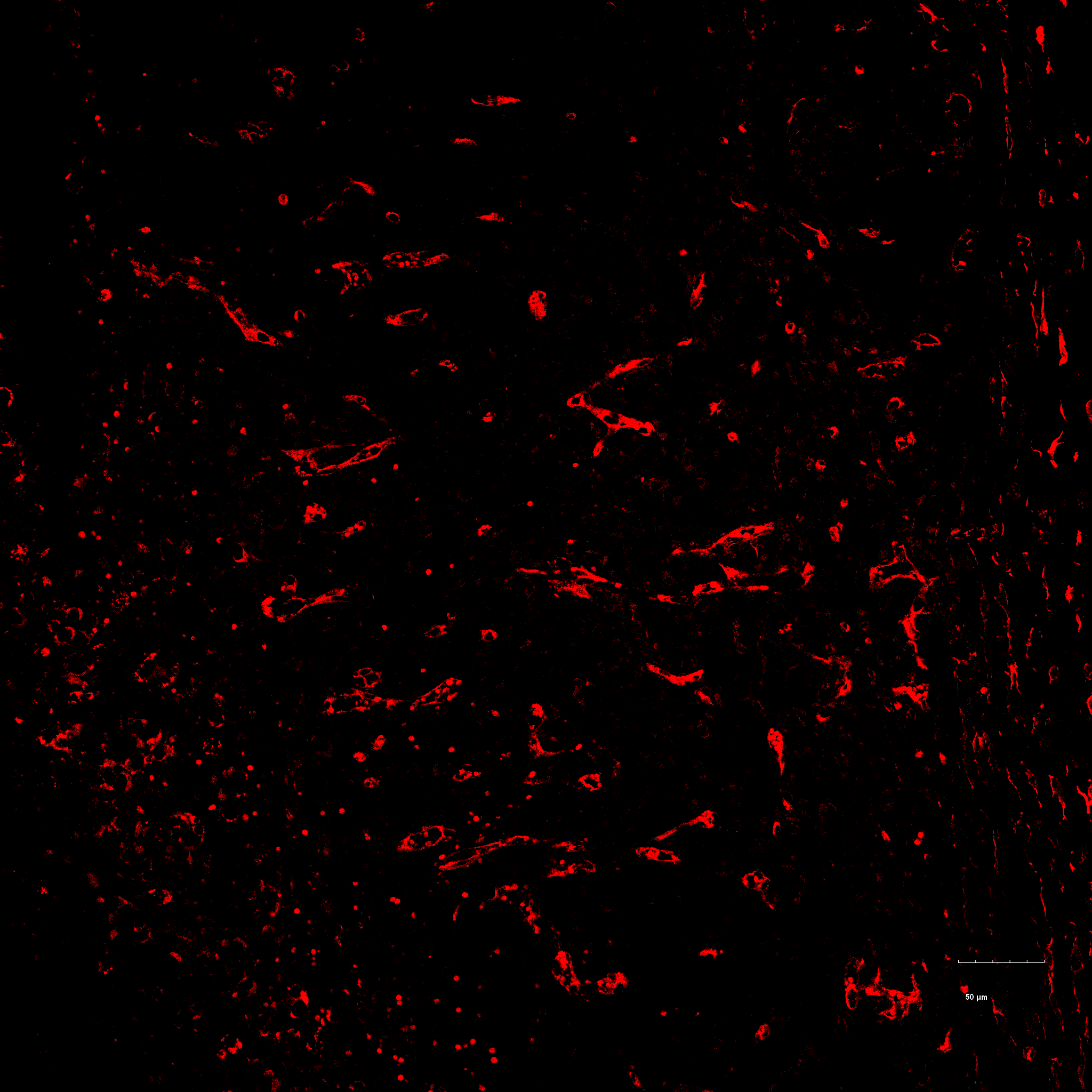

Supplement: Supplementary file 4 — Source data Fig. 2 [file 44319_2025_627_MOESM4_ESM.zip › Figure 2/2B/CD34 LLC A.f.png]

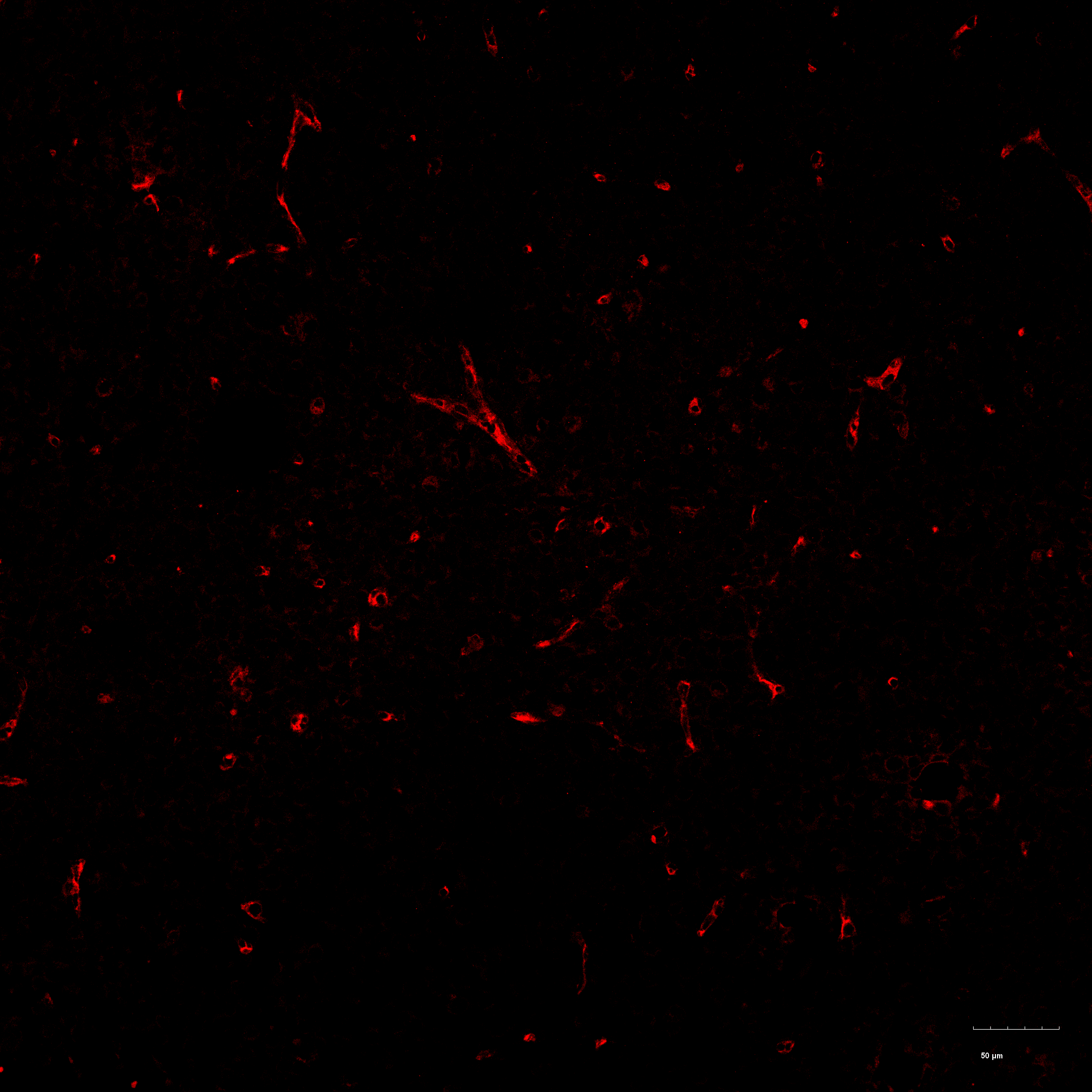

Supplement: Supplementary file 4 — Source data Fig. 2 [file 44319_2025_627_MOESM4_ESM.zip › Figure 2/2B/CD34 LLC Ctrl.tif]

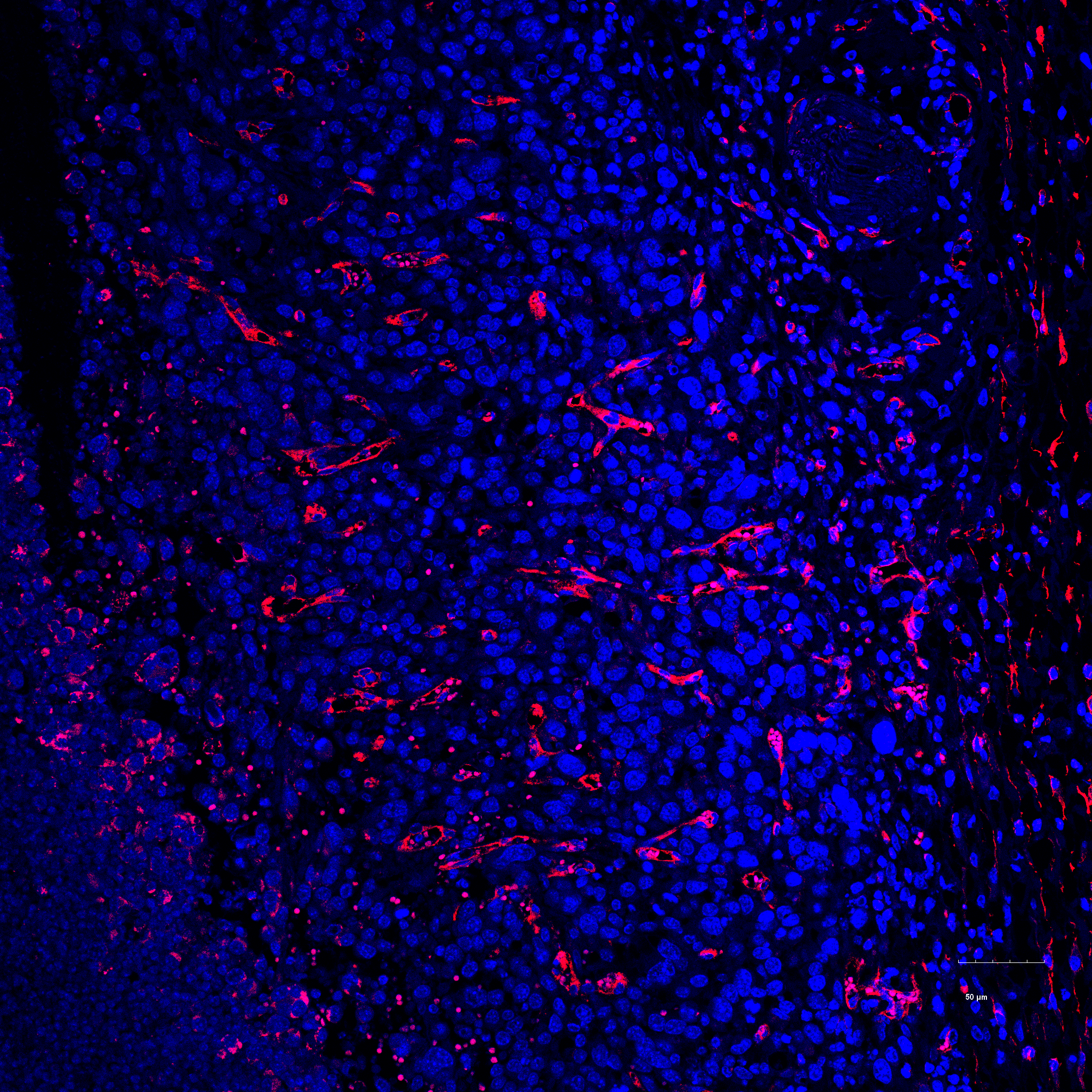

Supplement: Supplementary file 4 — Source data Fig. 2 [file 44319_2025_627_MOESM4_ESM.zip › Figure 2/2B/CD34 Merged LLC A.f.png]

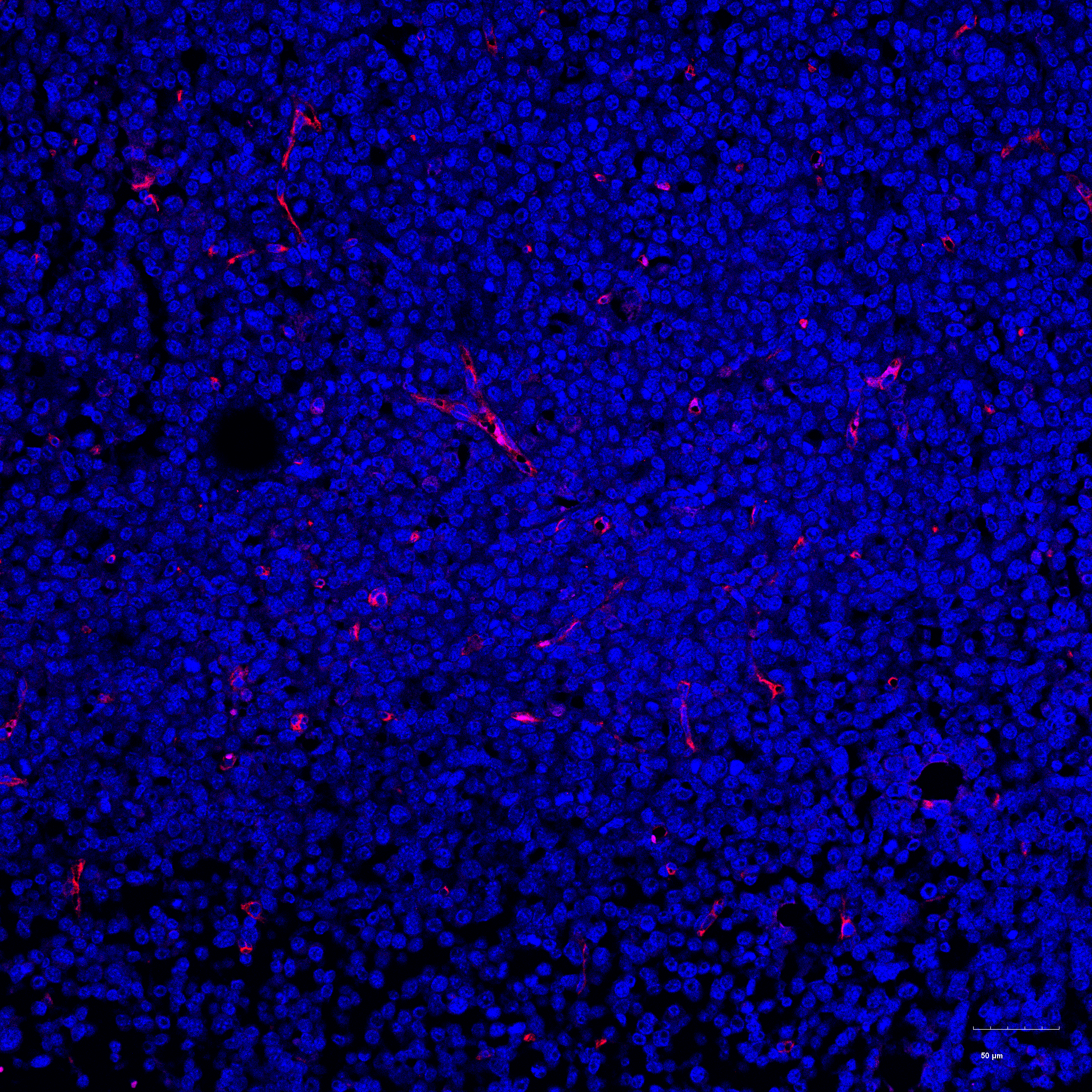

Supplement: Supplementary file 4 — Source data Fig. 2 [file 44319_2025_627_MOESM4_ESM.zip › Figure 2/2B/CD34 Merged LLC Ctrl.tif]

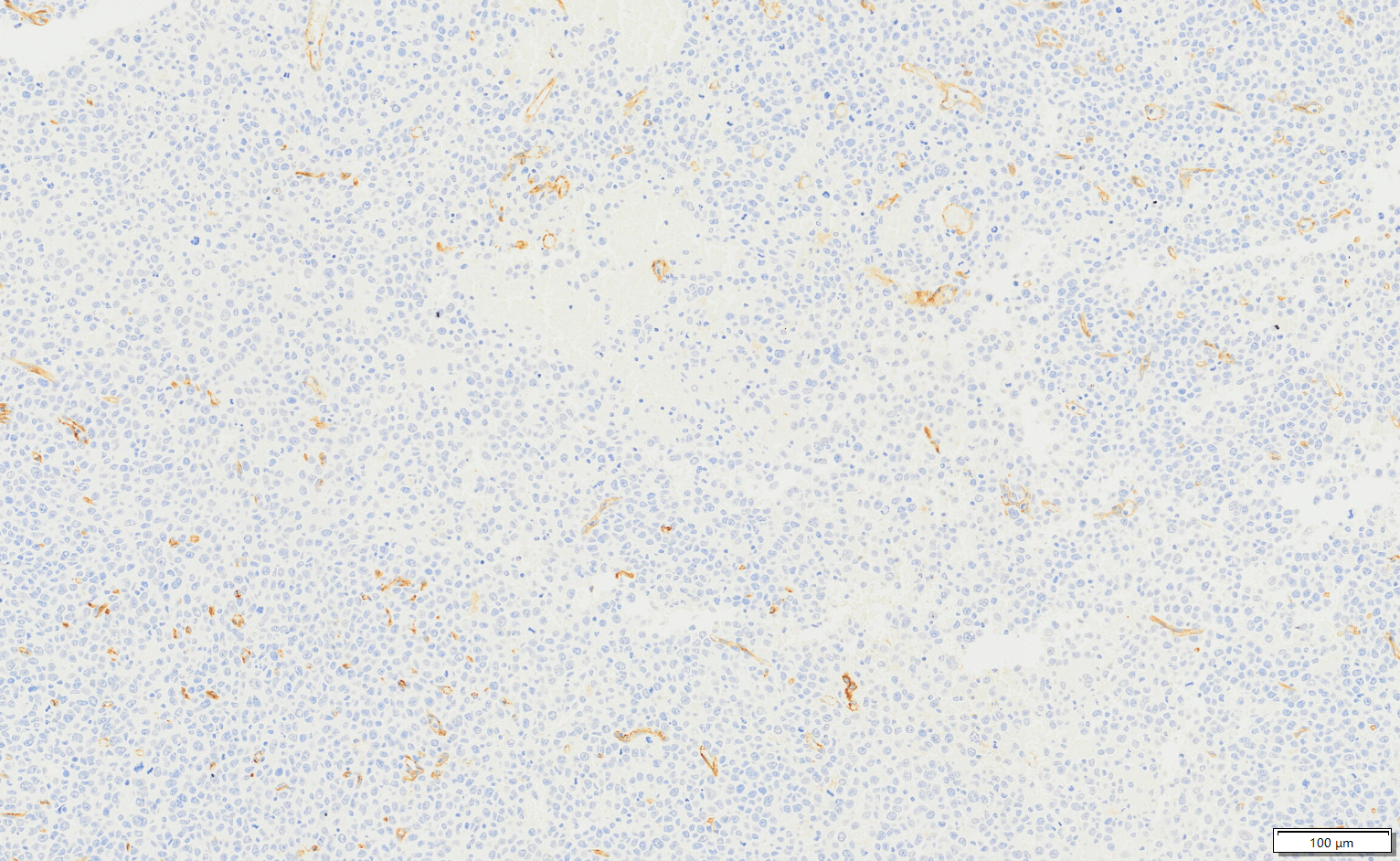

Supplement: Supplementary file 4 — Source data Fig. 2 [file 44319_2025_627_MOESM4_ESM.zip › Figure 2/2C/LLC Ctrl CD31.png]

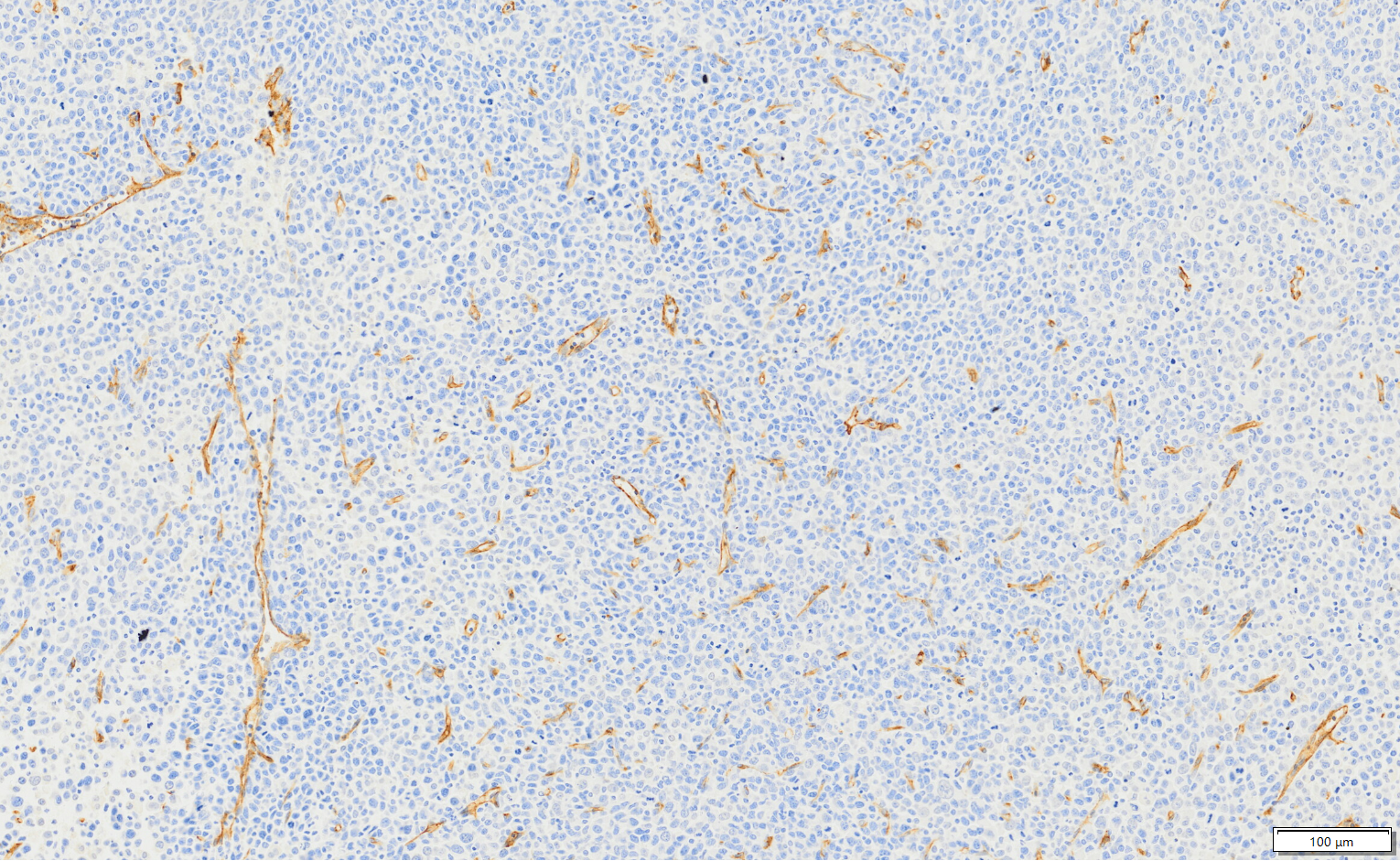

Supplement: Supplementary file 4 — Source data Fig. 2 [file 44319_2025_627_MOESM4_ESM.zip › Figure 2/2C/LLC A.f CD31.png]

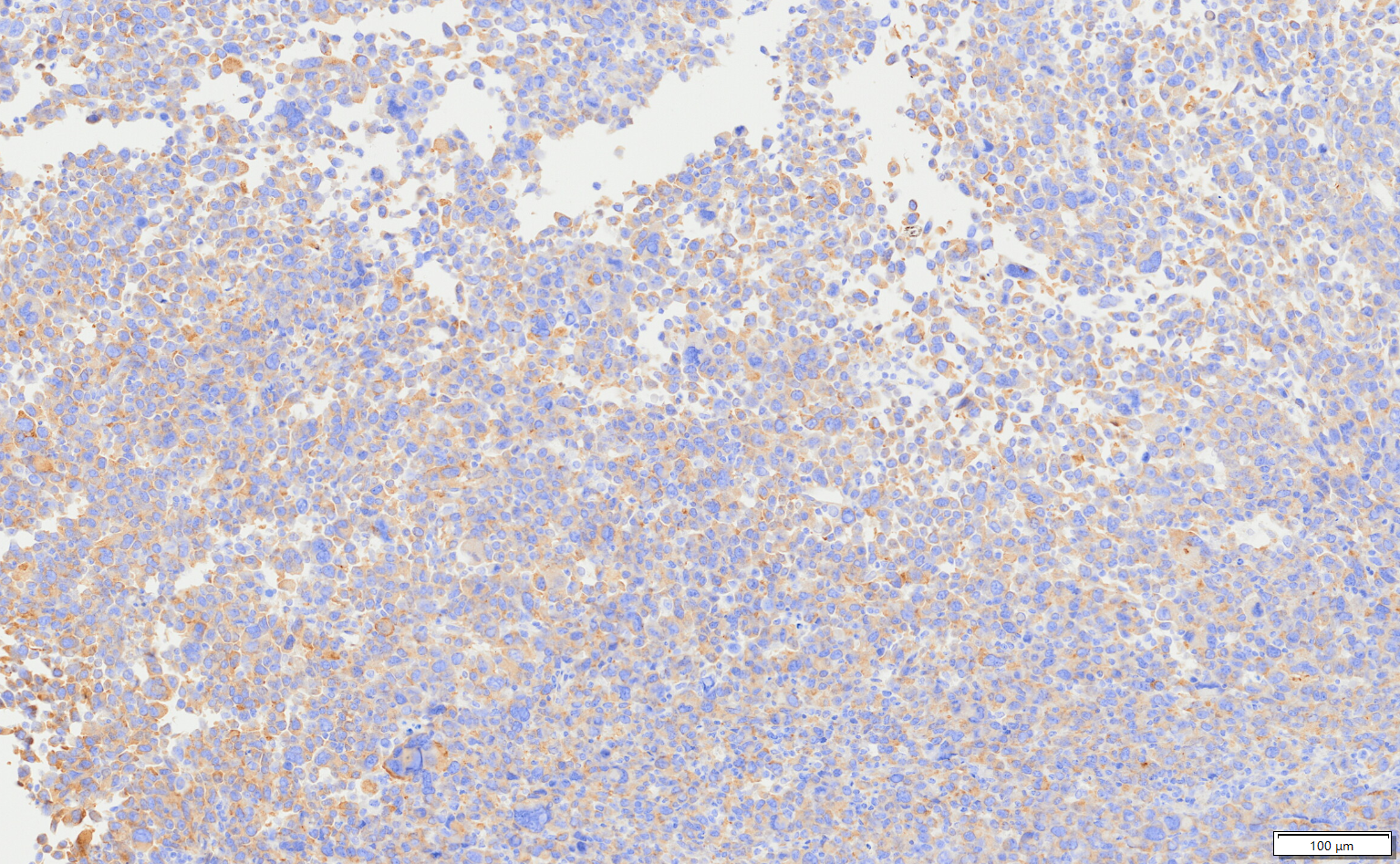

Supplement: Supplementary file 4 — Source data Fig. 2 [file 44319_2025_627_MOESM4_ESM.zip › Figure 2/2C/LLC A.f VEGF-A.png]

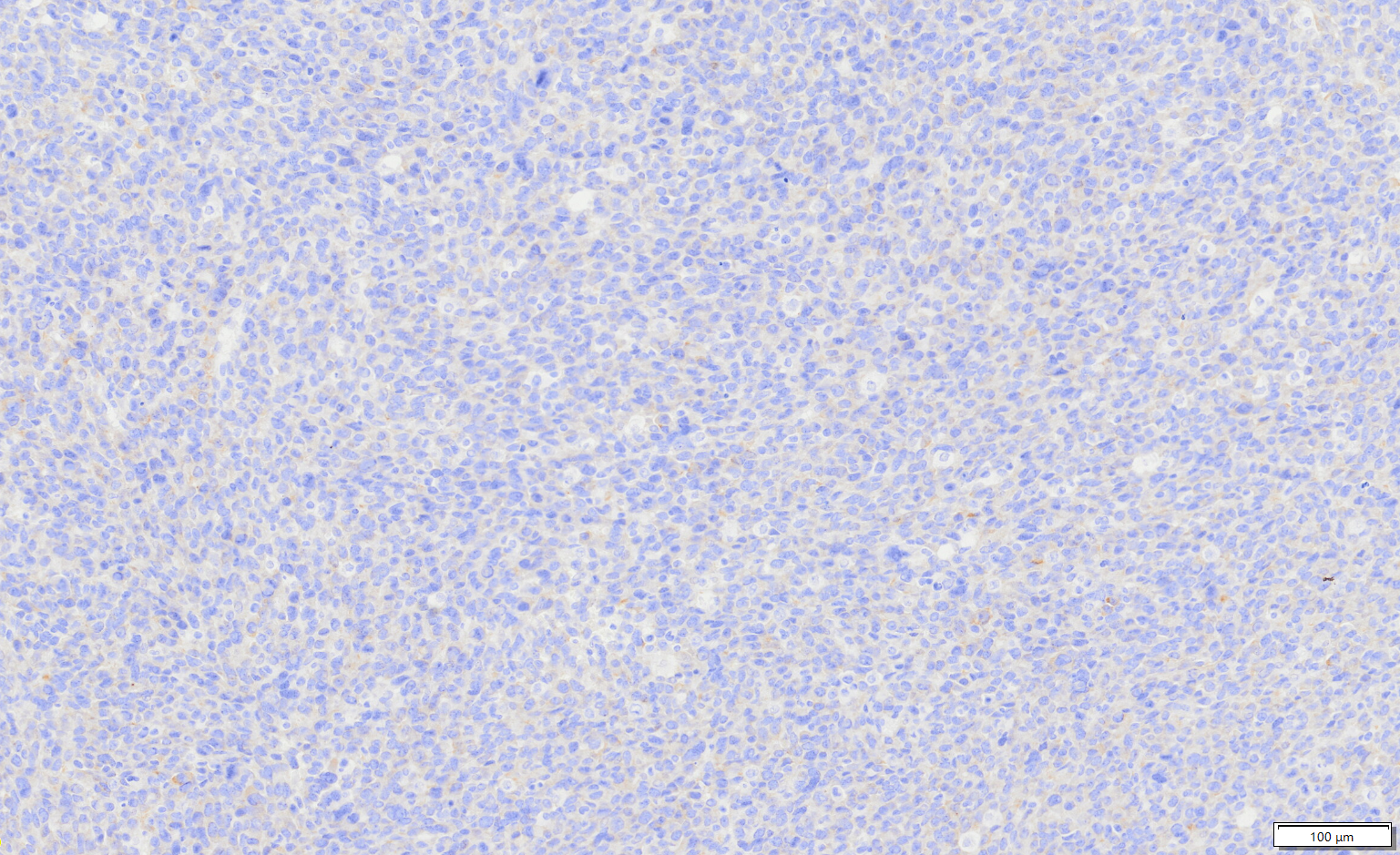

Supplement: Supplementary file 4 — Source data Fig. 2 [file 44319_2025_627_MOESM4_ESM.zip › Figure 2/2C/LLC Ctrl VEGF-A.png]

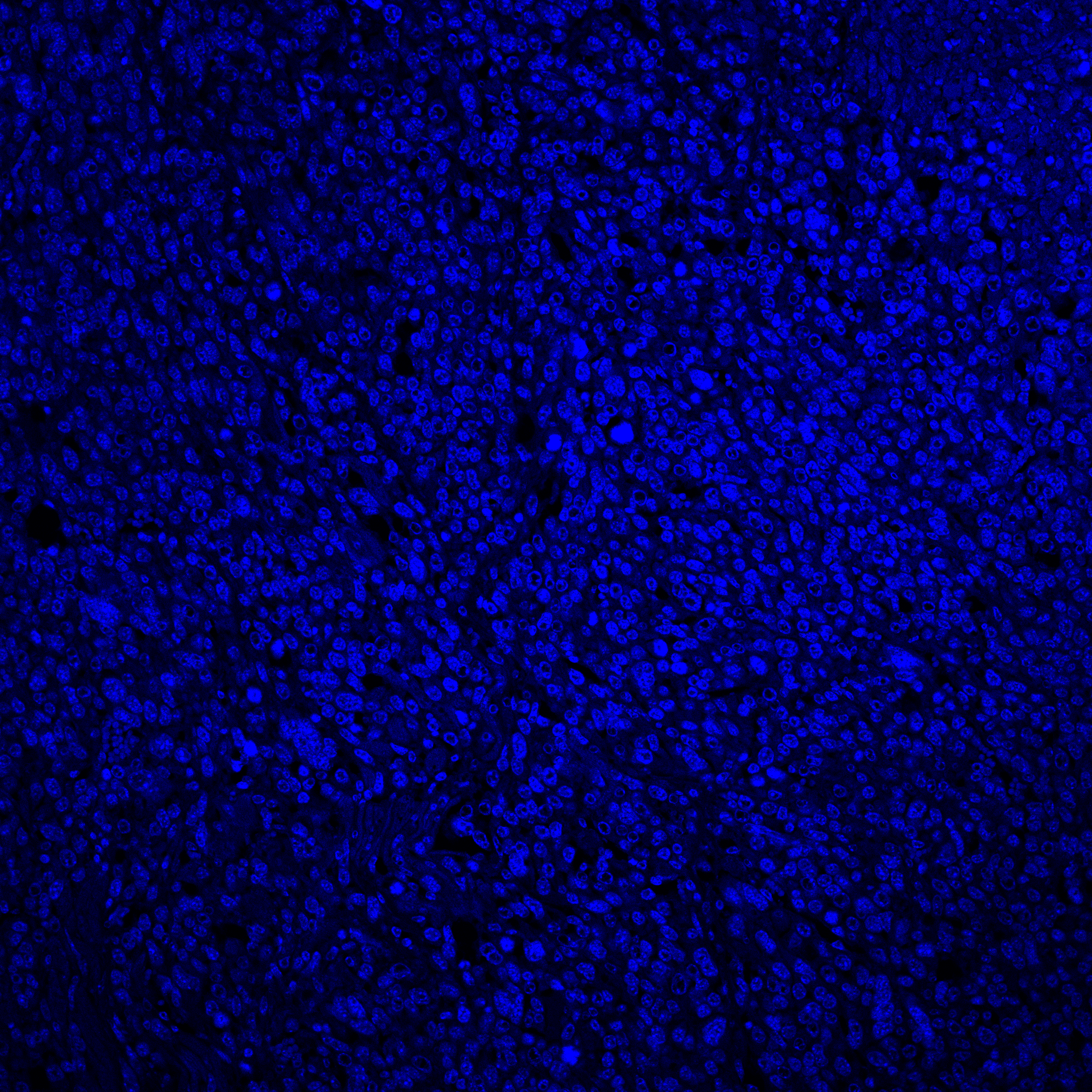

Supplement: Supplementary file 4 — Source data Fig. 2 [file 44319_2025_627_MOESM4_ESM.zip › Figure 2/2F/CD34 DAPI LUAD A.f.tif]

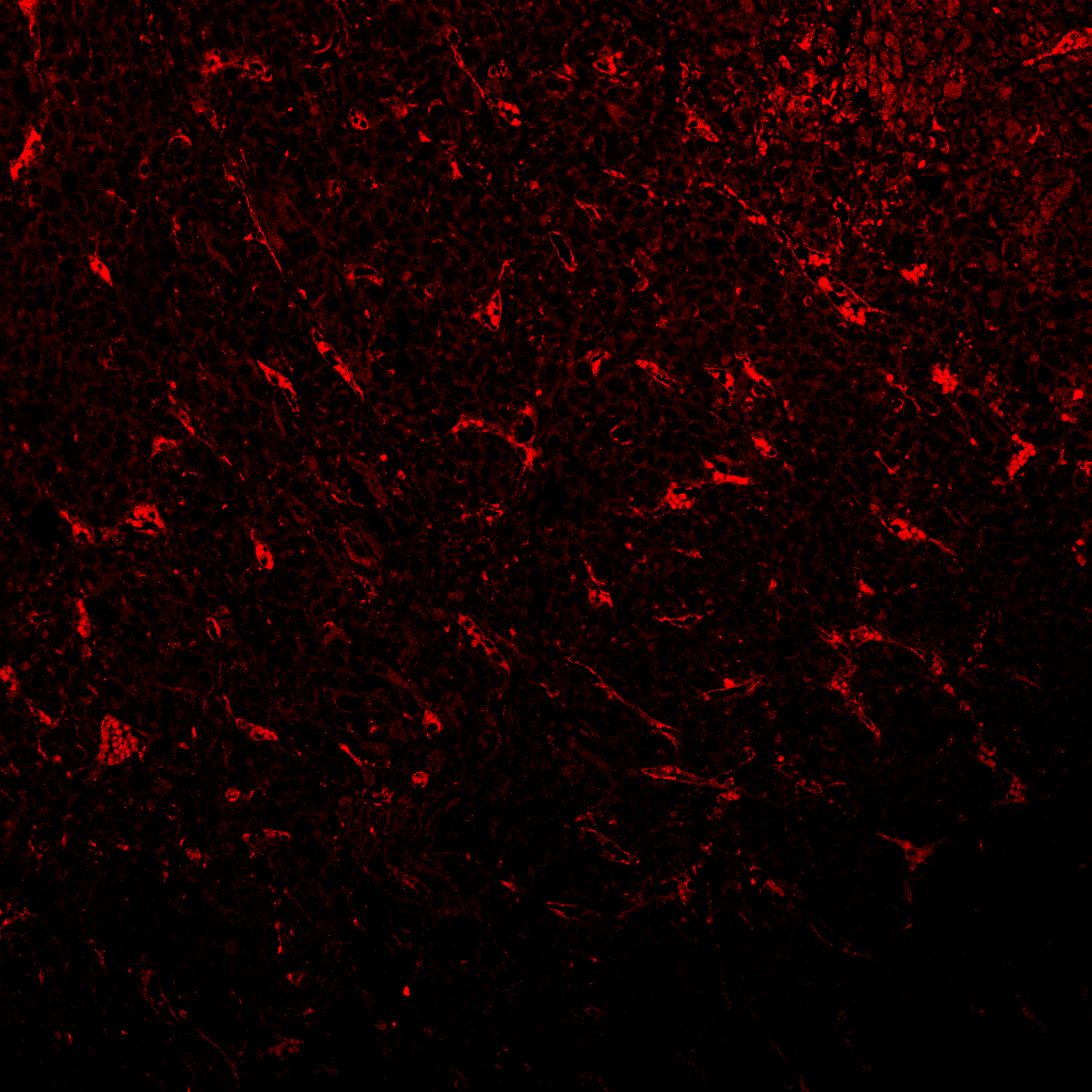

Supplement: Supplementary file 4 — Source data Fig. 2 [file 44319_2025_627_MOESM4_ESM.zip › Figure 2/2F/CD34 LUAD A.f.tif]

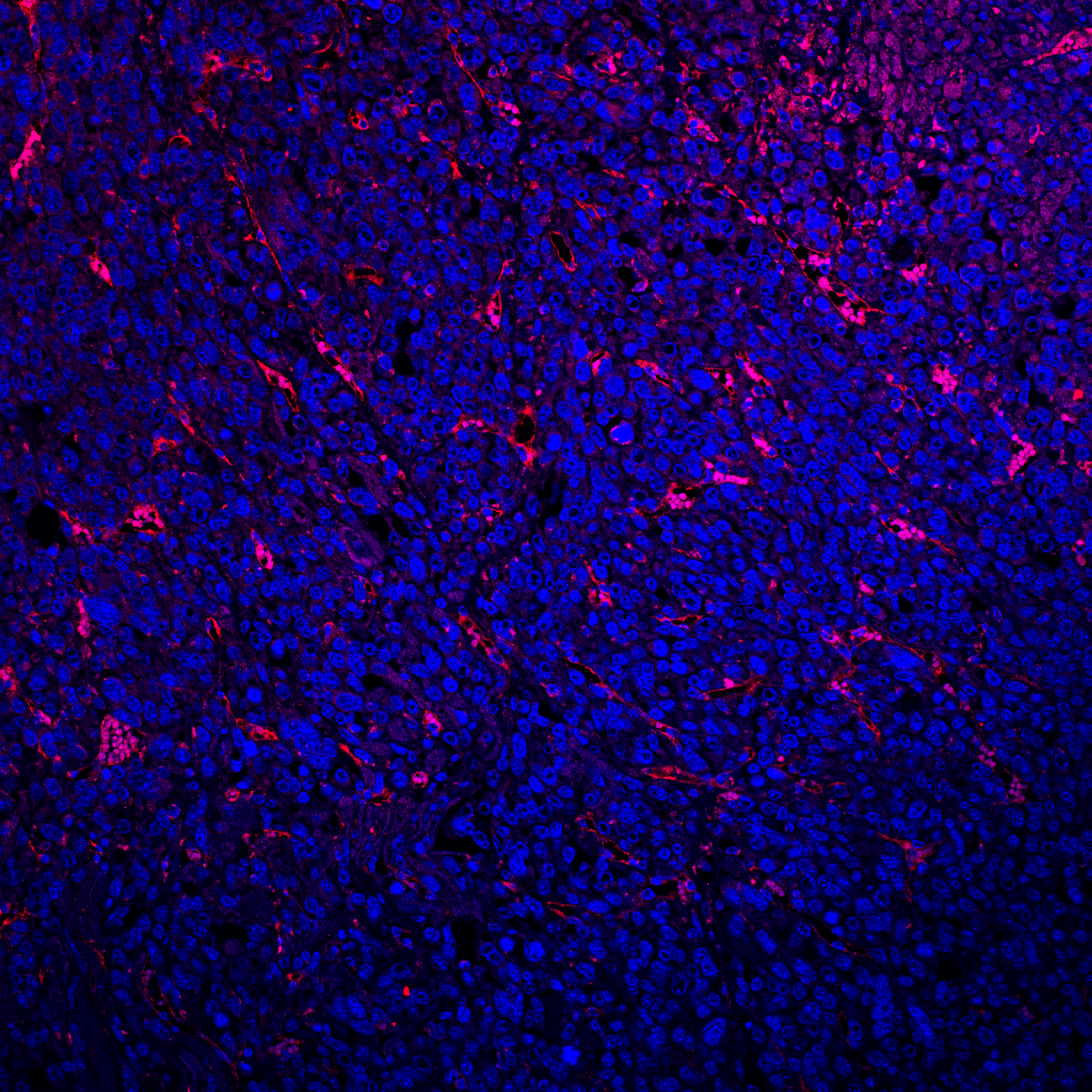

Supplement: Supplementary file 4 — Source data Fig. 2 [file 44319_2025_627_MOESM4_ESM.zip › Figure 2/2F/CD34 Merged LUAD A.f.tif]

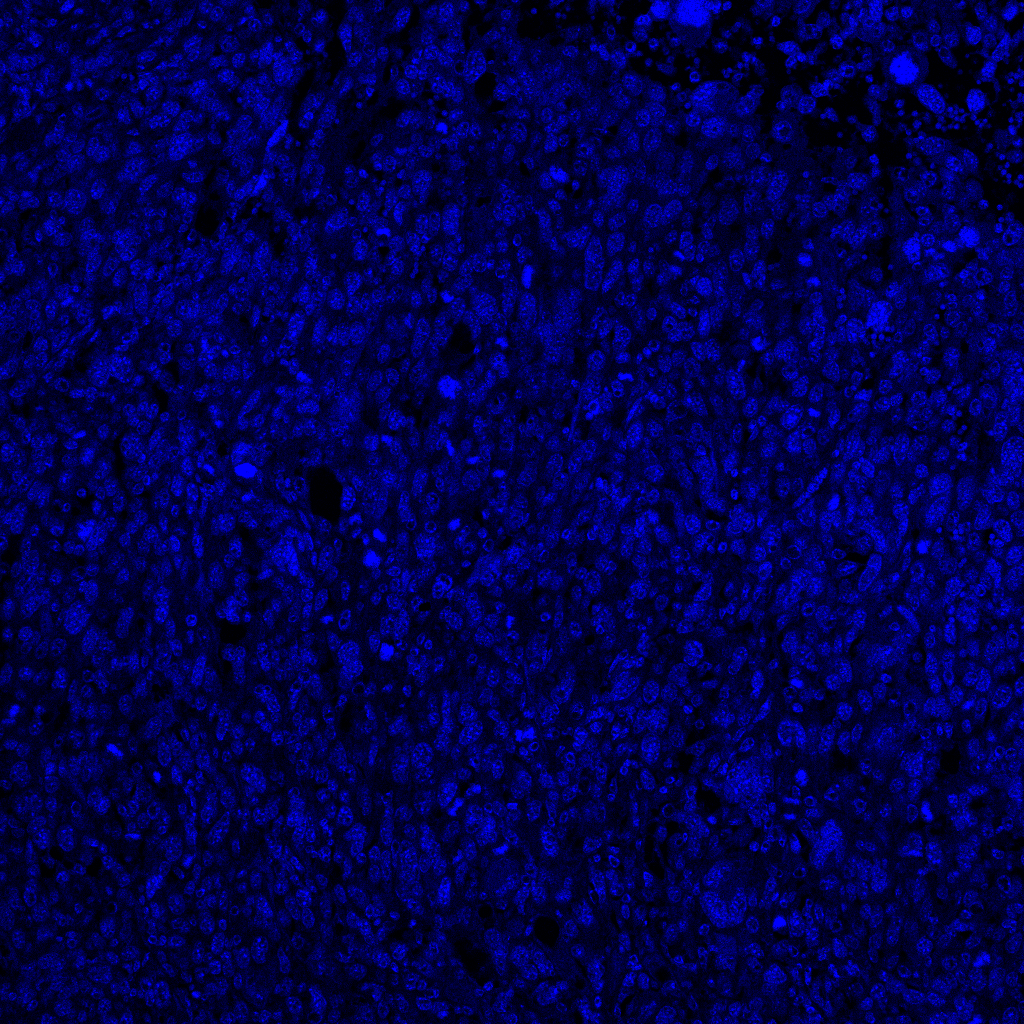

Supplement: Supplementary file 4 — Source data Fig. 2 [file 44319_2025_627_MOESM4_ESM.zip › Figure 2/2F/CD34 DAPI LUAD Ctrl.tif]

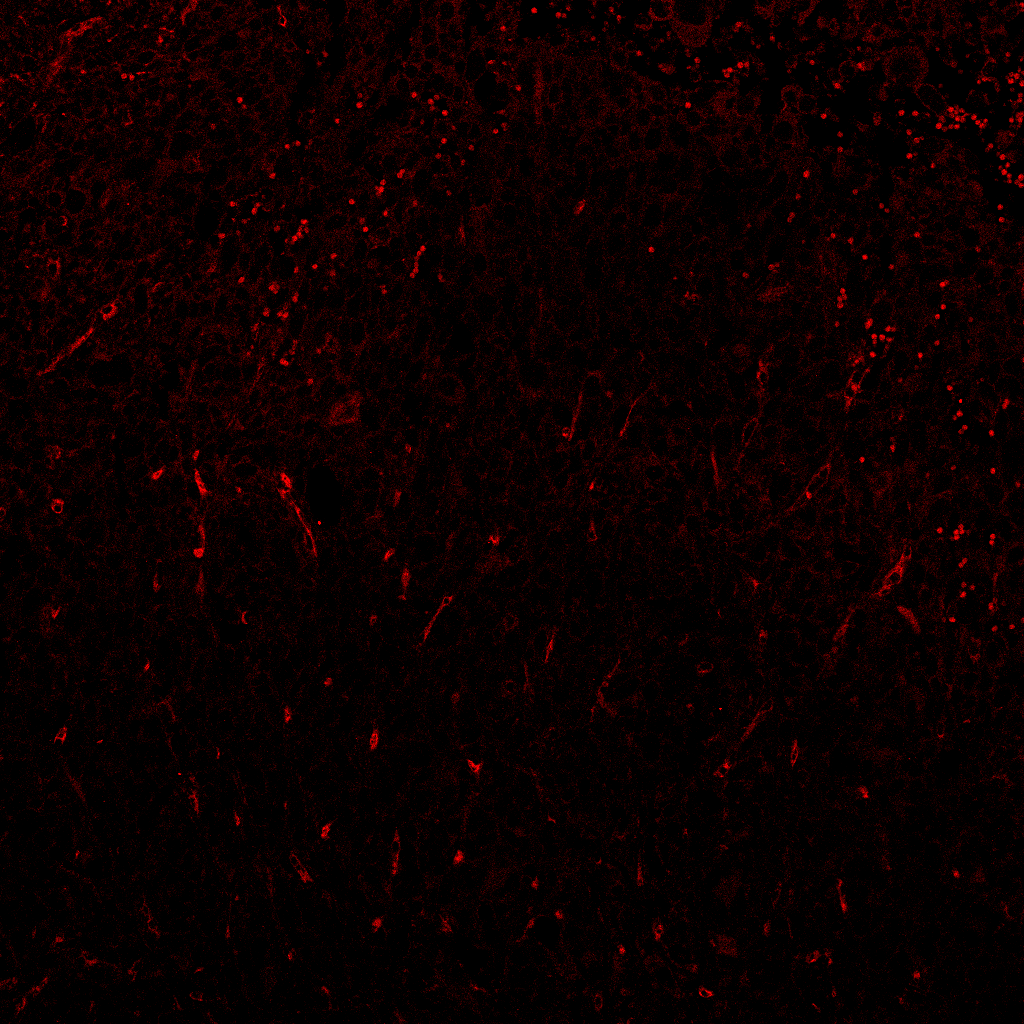

Supplement: Supplementary file 4 — Source data Fig. 2 [file 44319_2025_627_MOESM4_ESM.zip › Figure 2/2F/CD34 LUAD Ctrl.tif]

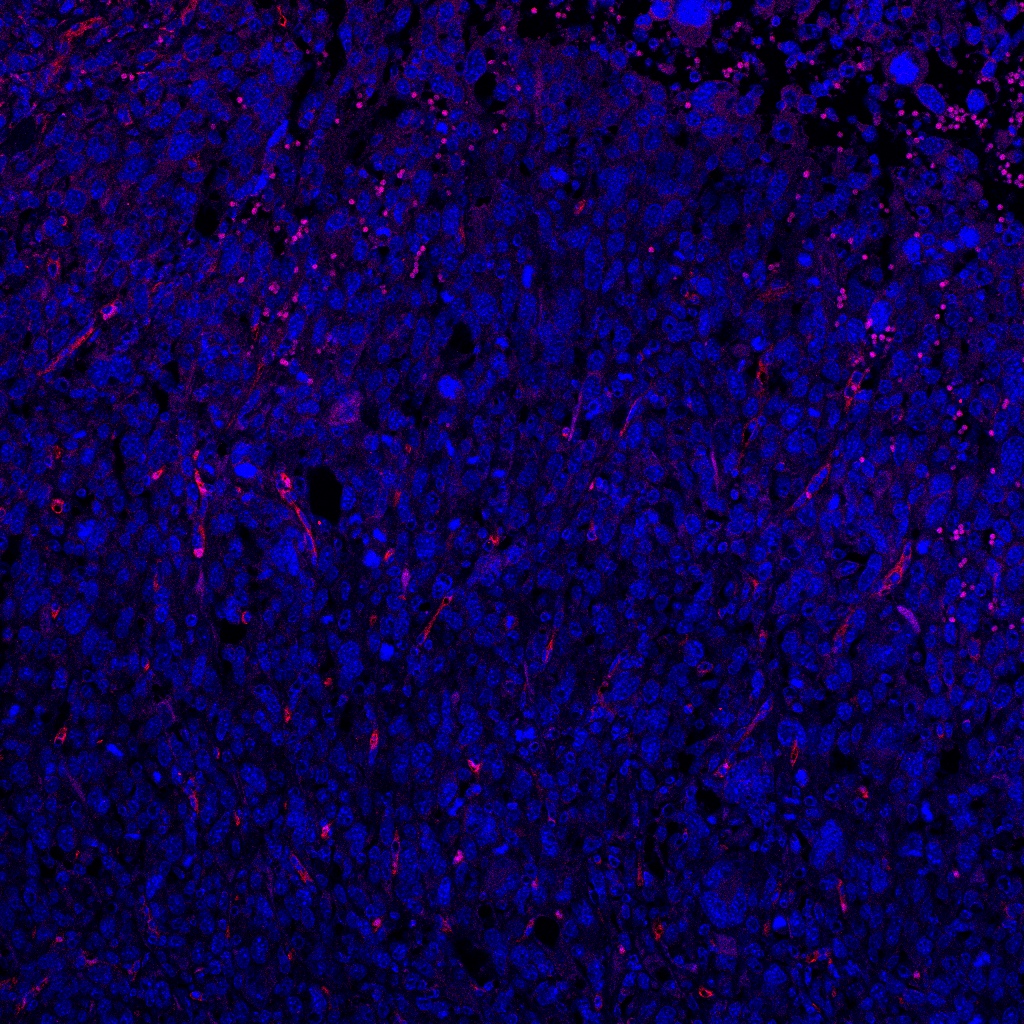

Supplement: Supplementary file 4 — Source data Fig. 2 [file 44319_2025_627_MOESM4_ESM.zip › Figure 2/2F/CD34 Merged LUAD Ctrl.tif]

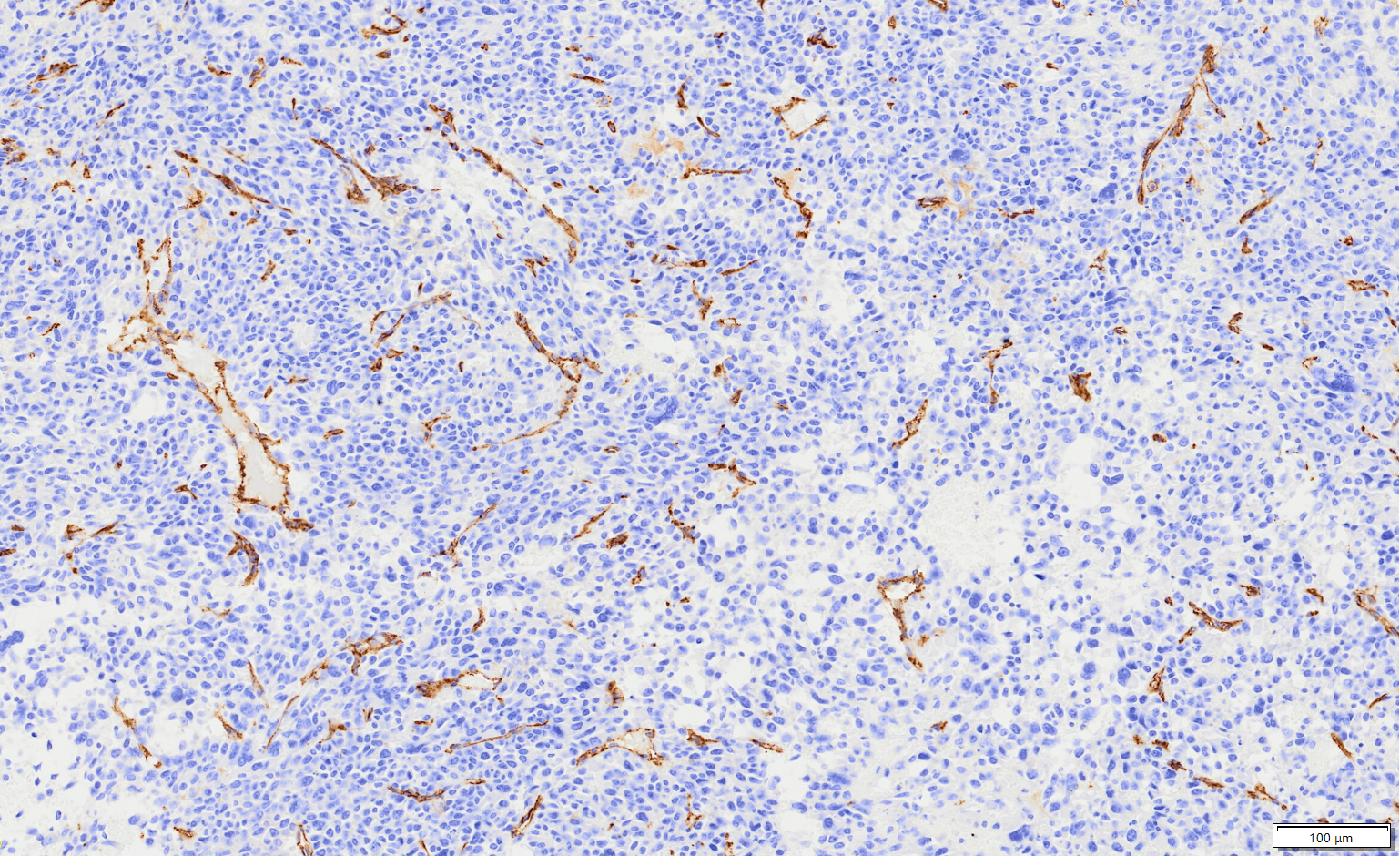

Supplement: Supplementary file 4 — Source data Fig. 2 [file 44319_2025_627_MOESM4_ESM.zip › Figure 2/2G/LUAD A.f CD31.png]

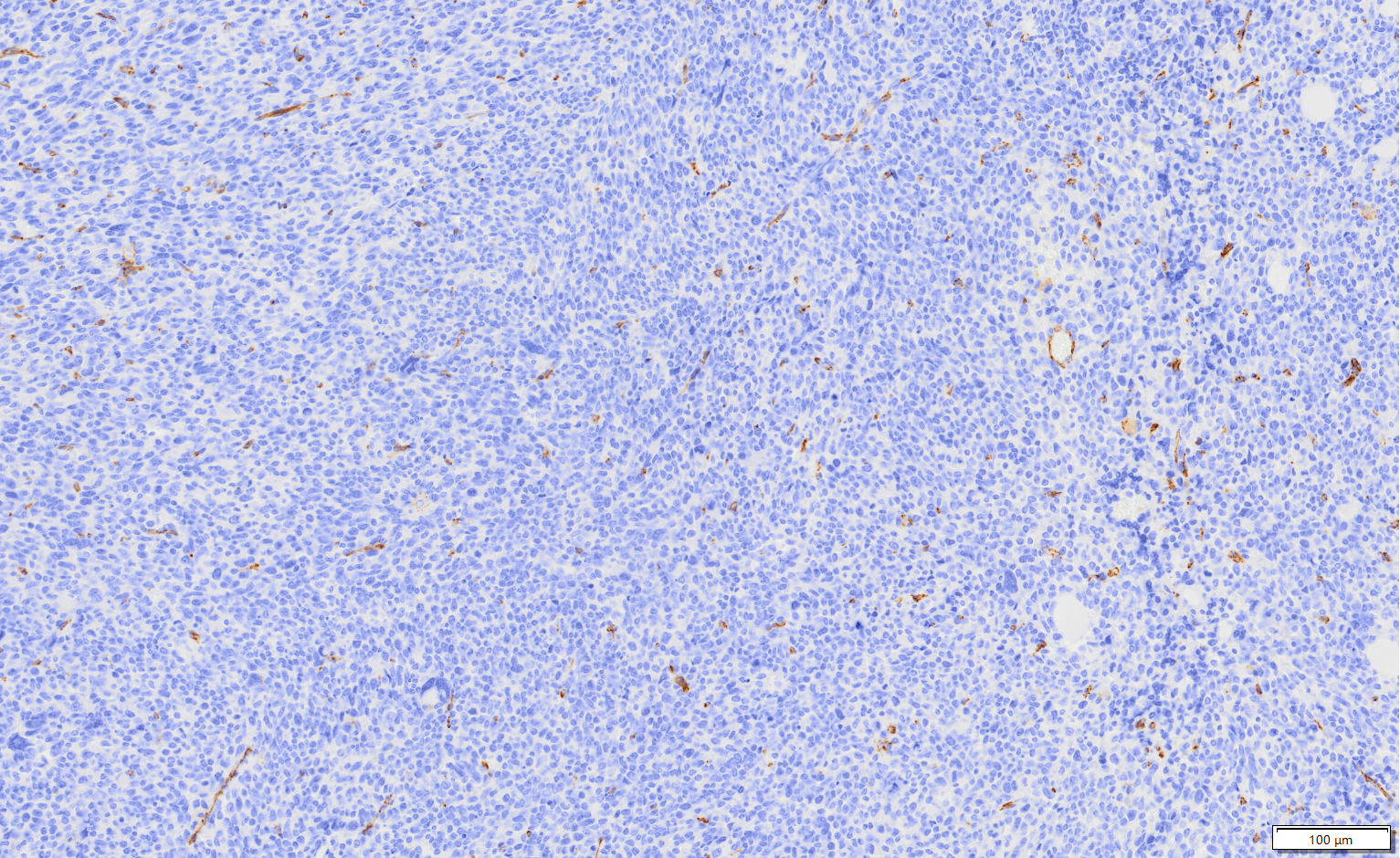

Supplement: Supplementary file 4 — Source data Fig. 2 [file 44319_2025_627_MOESM4_ESM.zip › Figure 2/2G/LUAD Ctrl CD31.png]

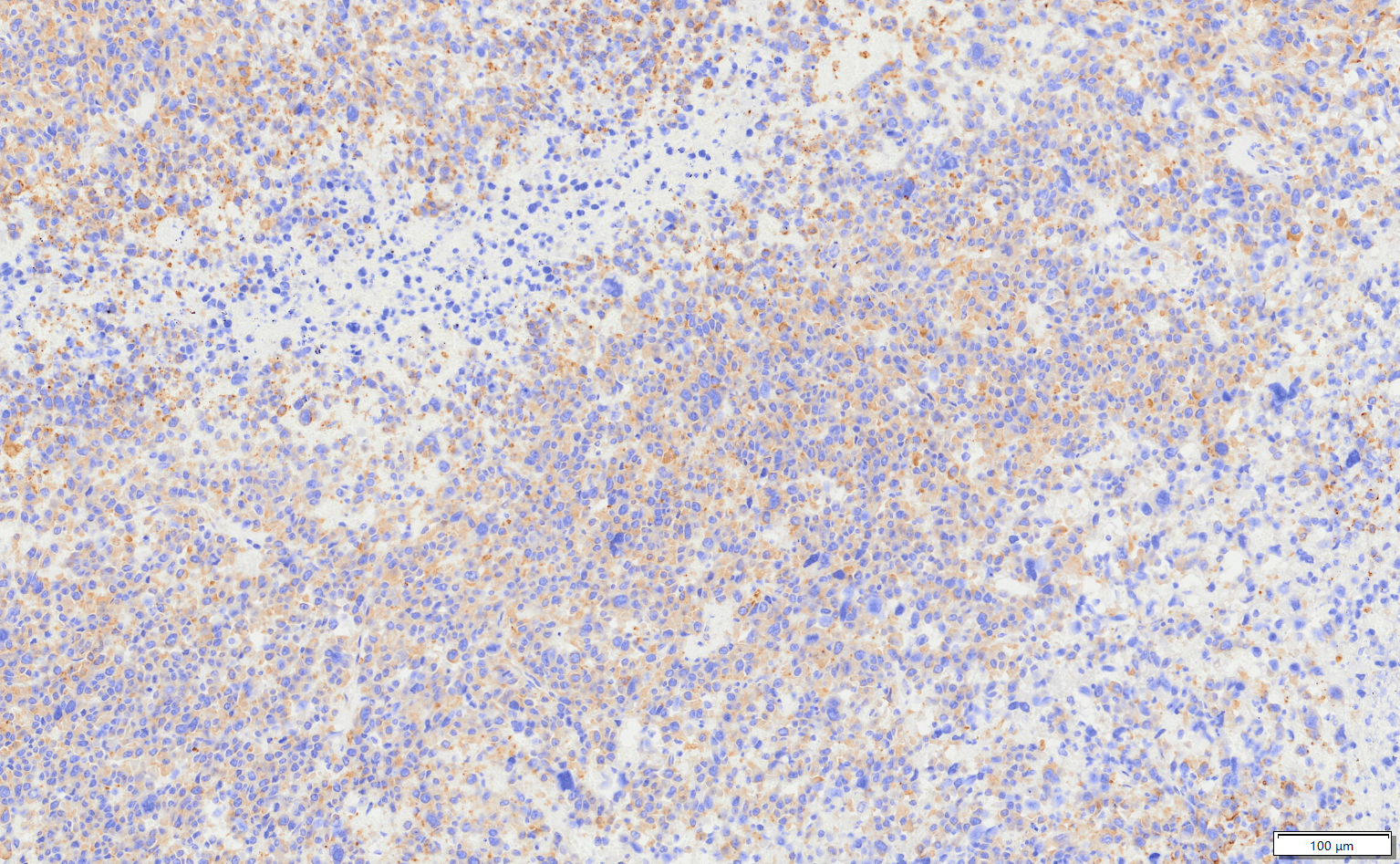

Supplement: Supplementary file 4 — Source data Fig. 2 [file 44319_2025_627_MOESM4_ESM.zip › Figure 2/2I/LUAD A.f VEGF-A.png]

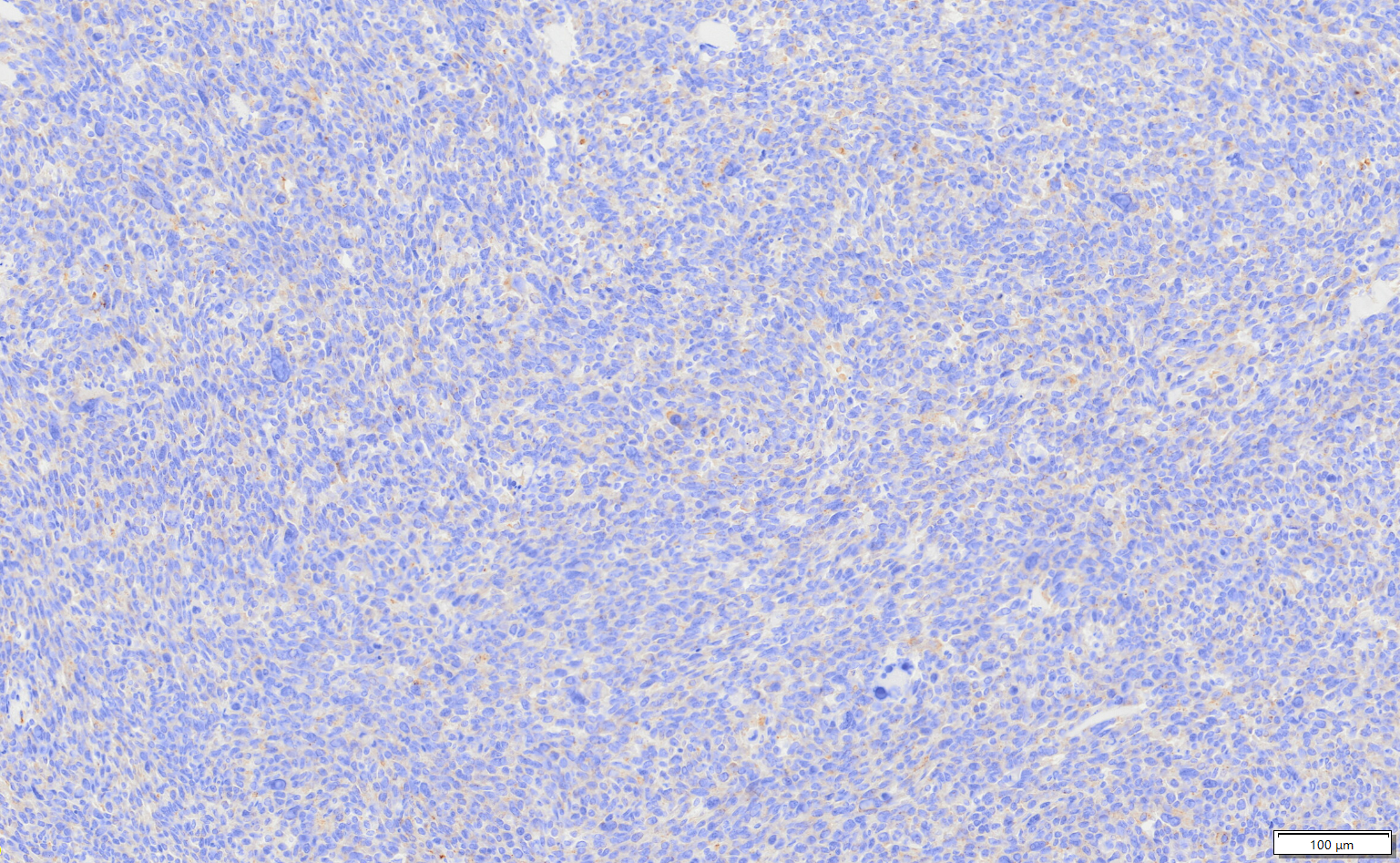

Supplement: Supplementary file 4 — Source data Fig. 2 [file 44319_2025_627_MOESM4_ESM.zip › Figure 2/2I/LUAD Ctrl VEGF-A.png]

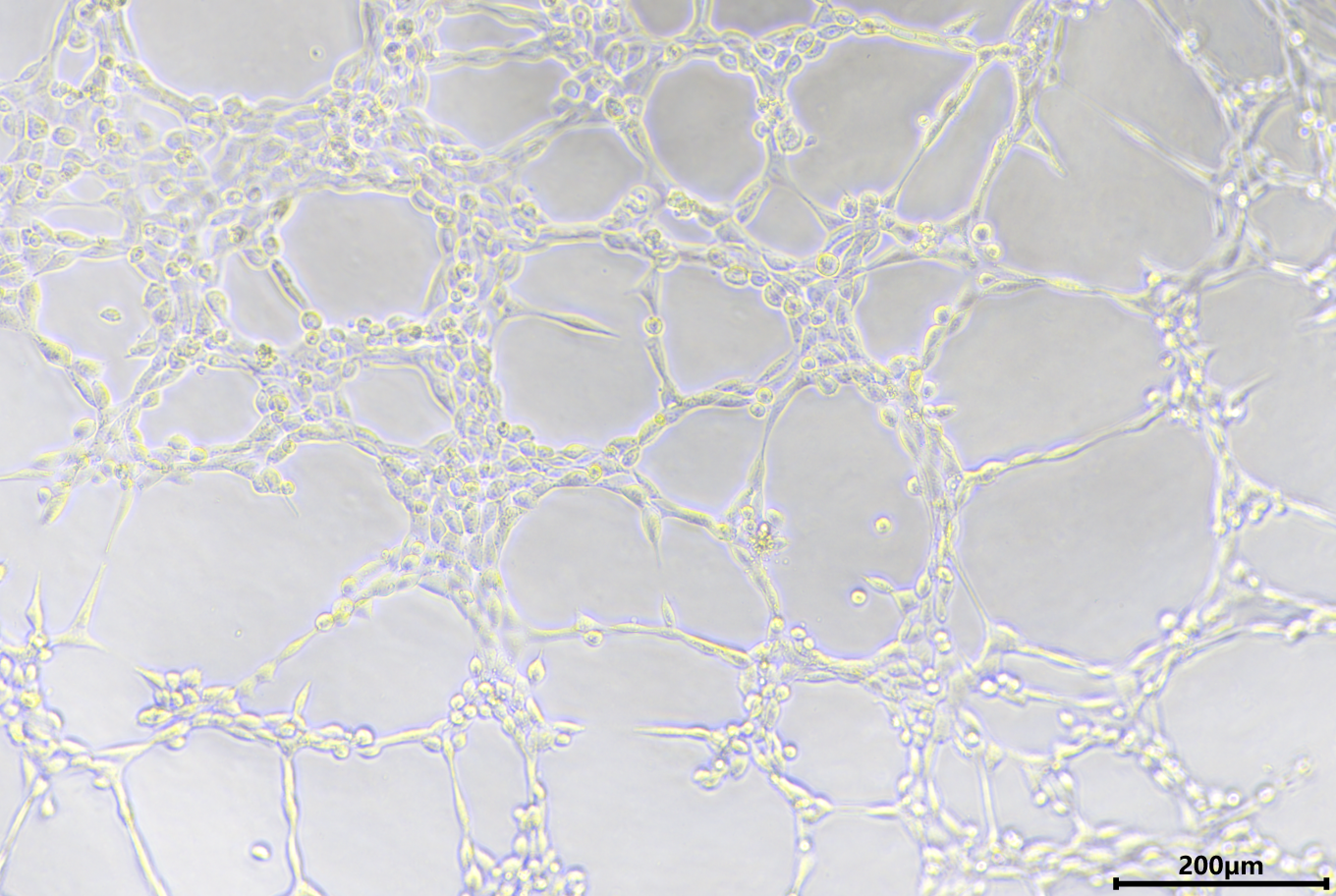

Supplement: Supplementary file 5 — Source data Fig. 3 [file 44319_2025_627_MOESM5_ESM.zip › Figure 3/3D/A. f.tiff]

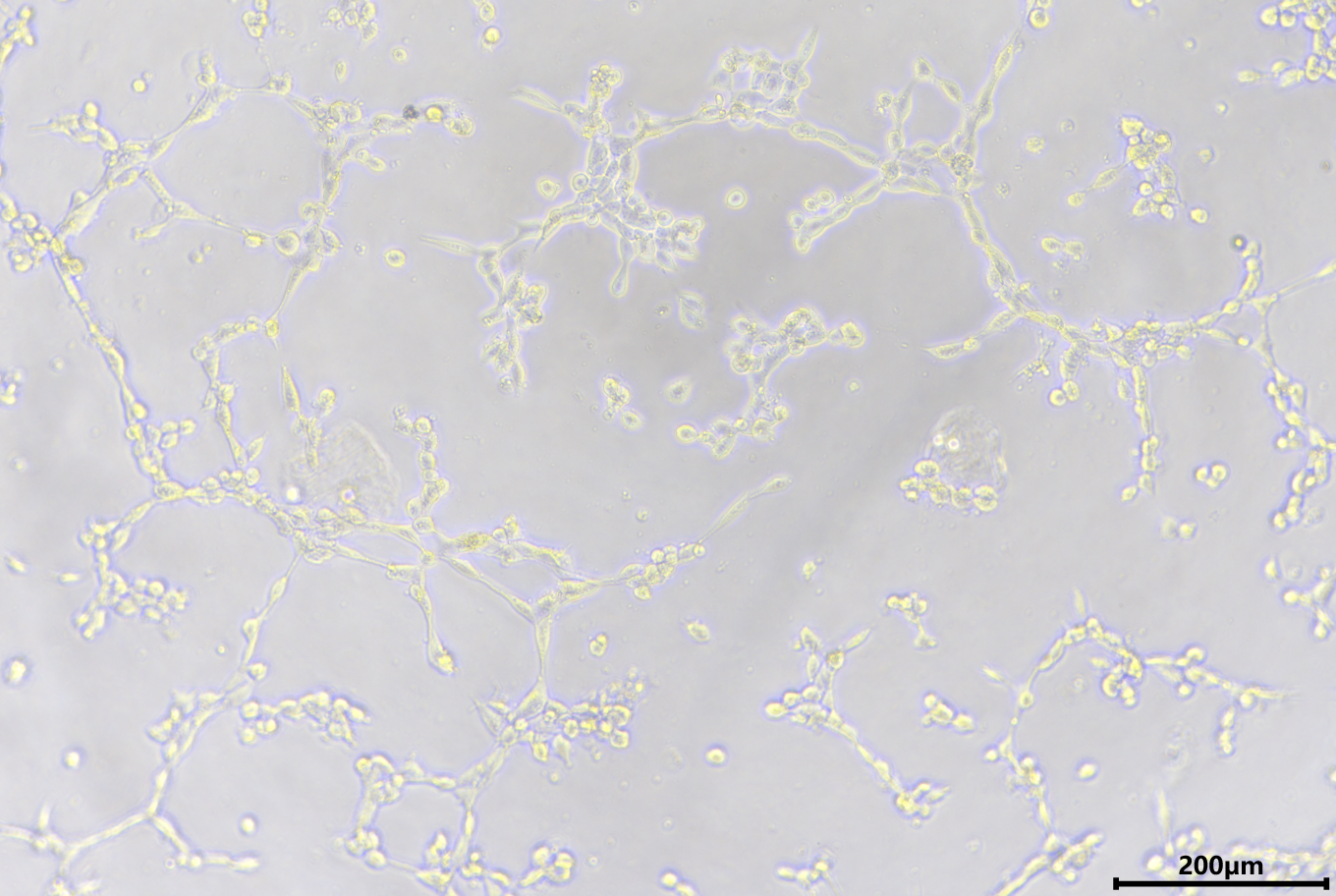

Supplement: Supplementary file 5 — Source data Fig. 3 [file 44319_2025_627_MOESM5_ESM.zip › Figure 3/3D/Ctrl.tiff]

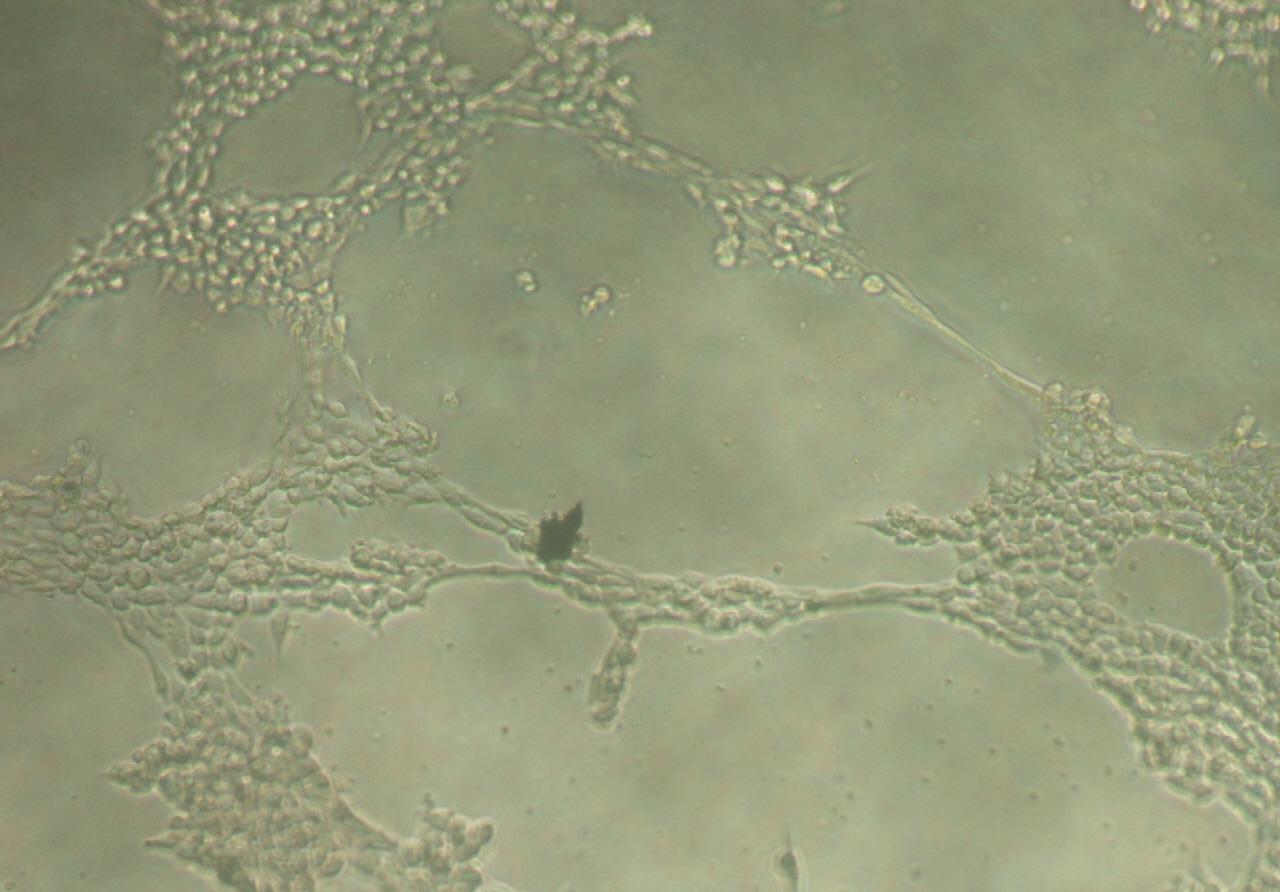

Supplement: Supplementary file 5 — Source data Fig. 3 [file 44319_2025_627_MOESM5_ESM.zip › Figure 3/3I/A.f.jpg]

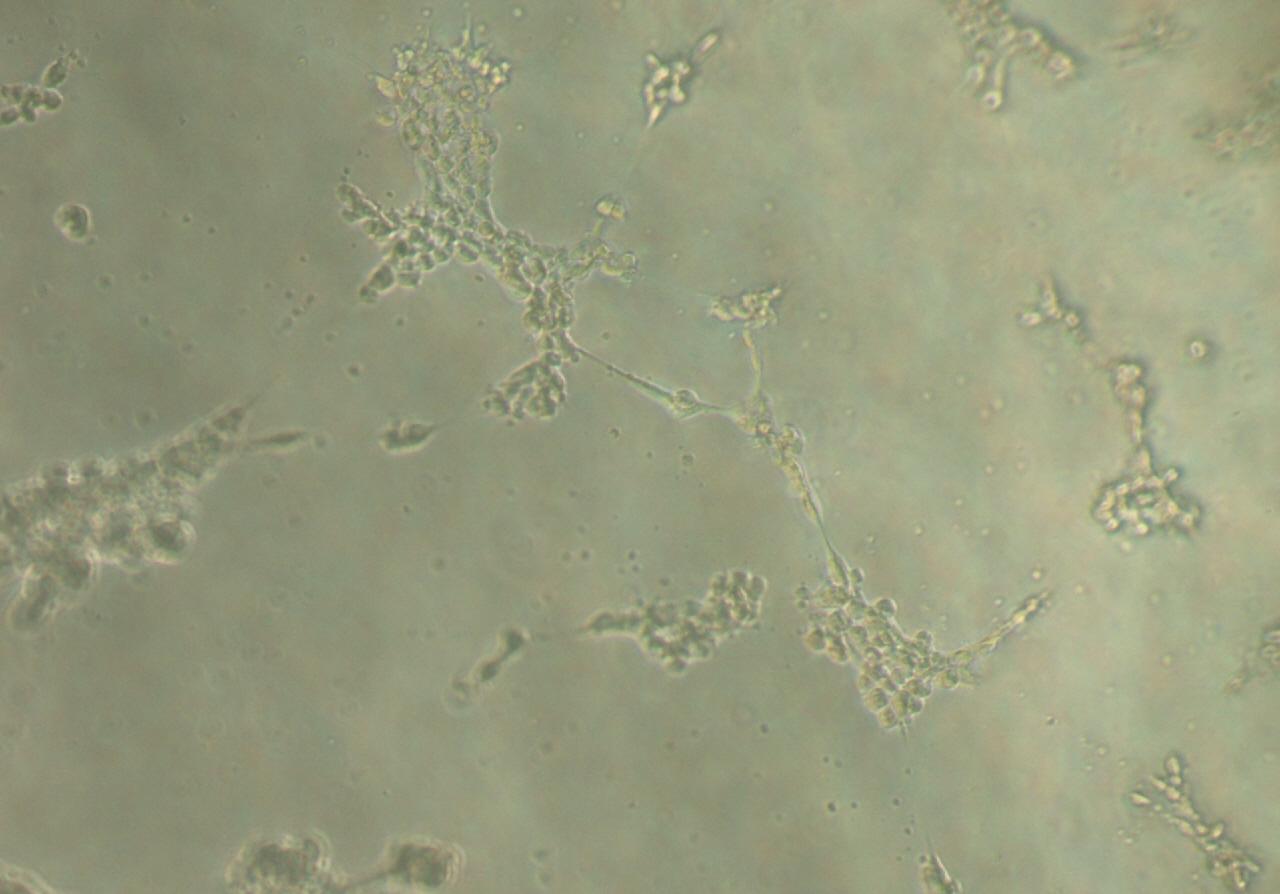

Supplement: Supplementary file 5 — Source data Fig. 3 [file 44319_2025_627_MOESM5_ESM.zip › Figure 3/3I/Ctrl.jpg]

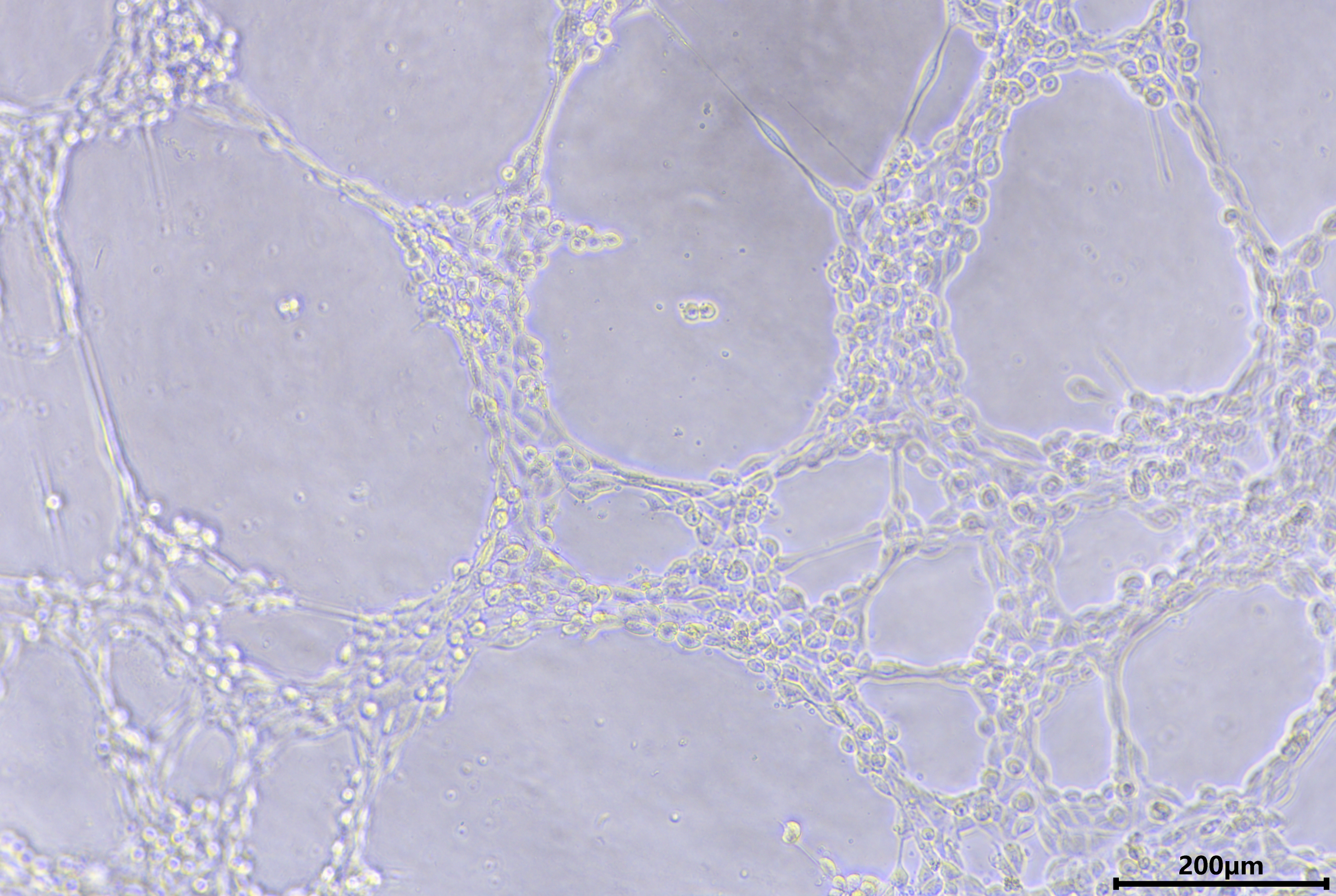

Supplement: Supplementary file 5 — Source data Fig. 3 [file 44319_2025_627_MOESM5_ESM.zip › Figure 3/3K/A.f.tiff]

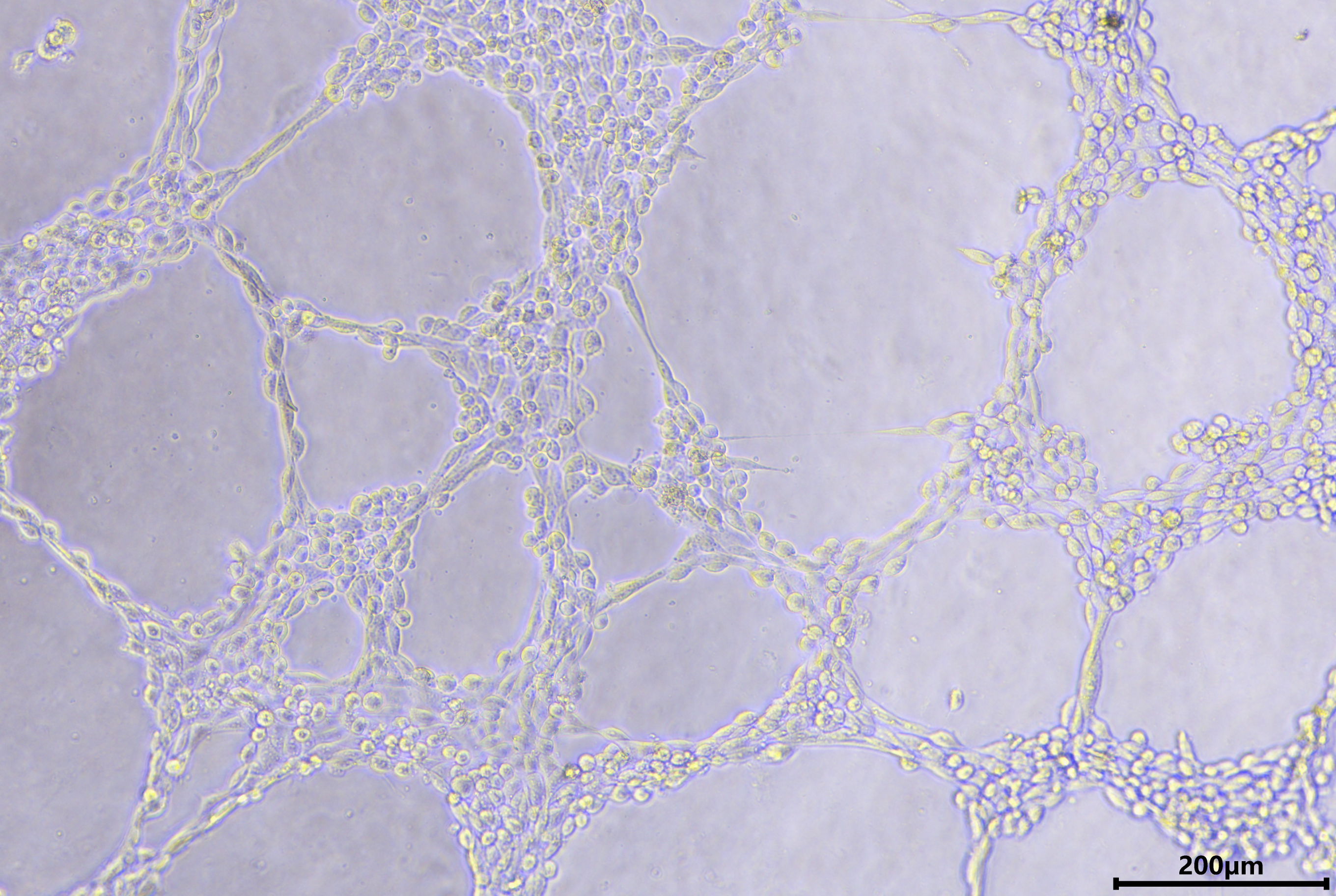

Supplement: Supplementary file 5 — Source data Fig. 3 [file 44319_2025_627_MOESM5_ESM.zip › Figure 3/3K/NC.tiff]

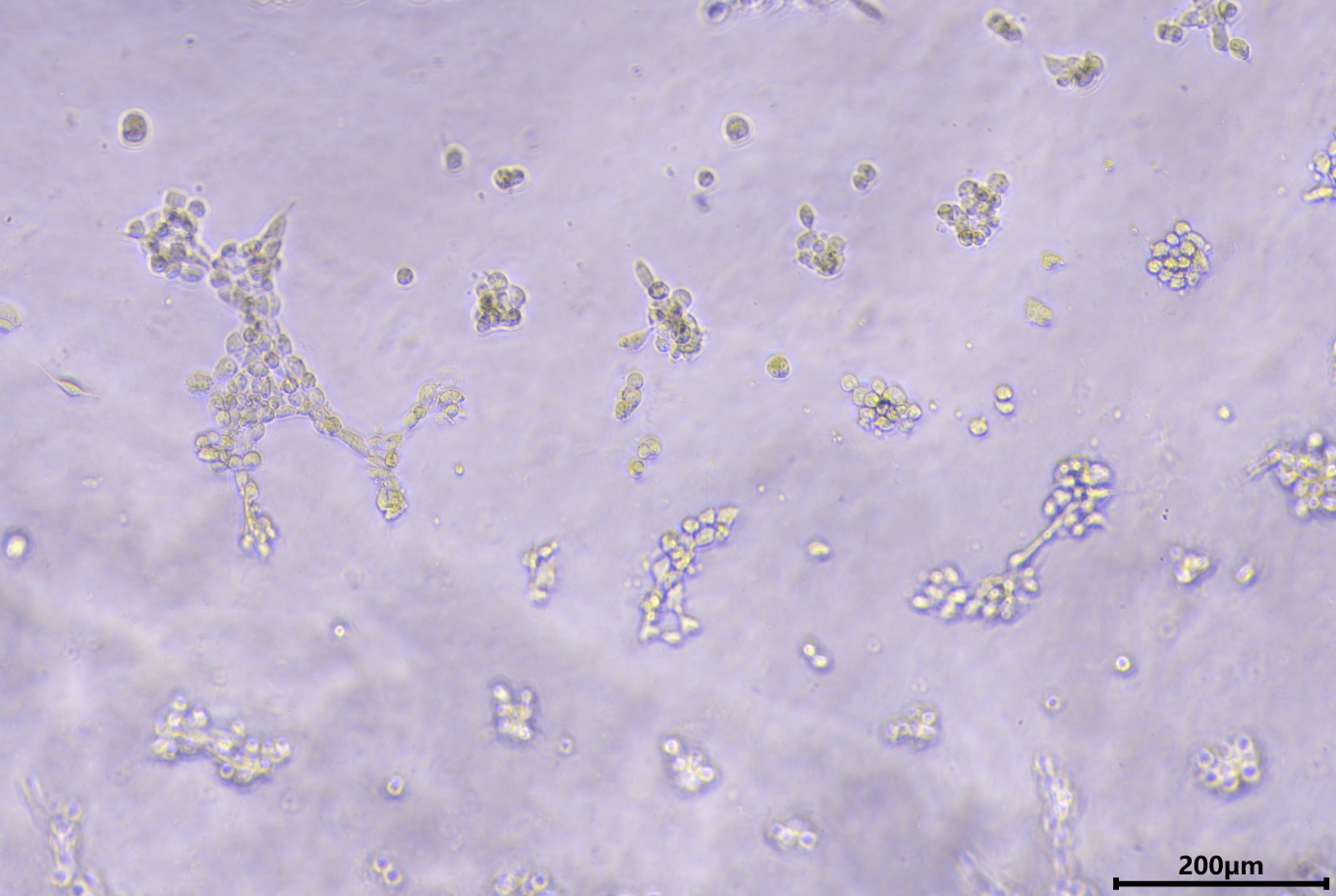

Supplement: Supplementary file 5 — Source data Fig. 3 [file 44319_2025_627_MOESM5_ESM.zip › Figure 3/3K/siVEGF-A.tiff]

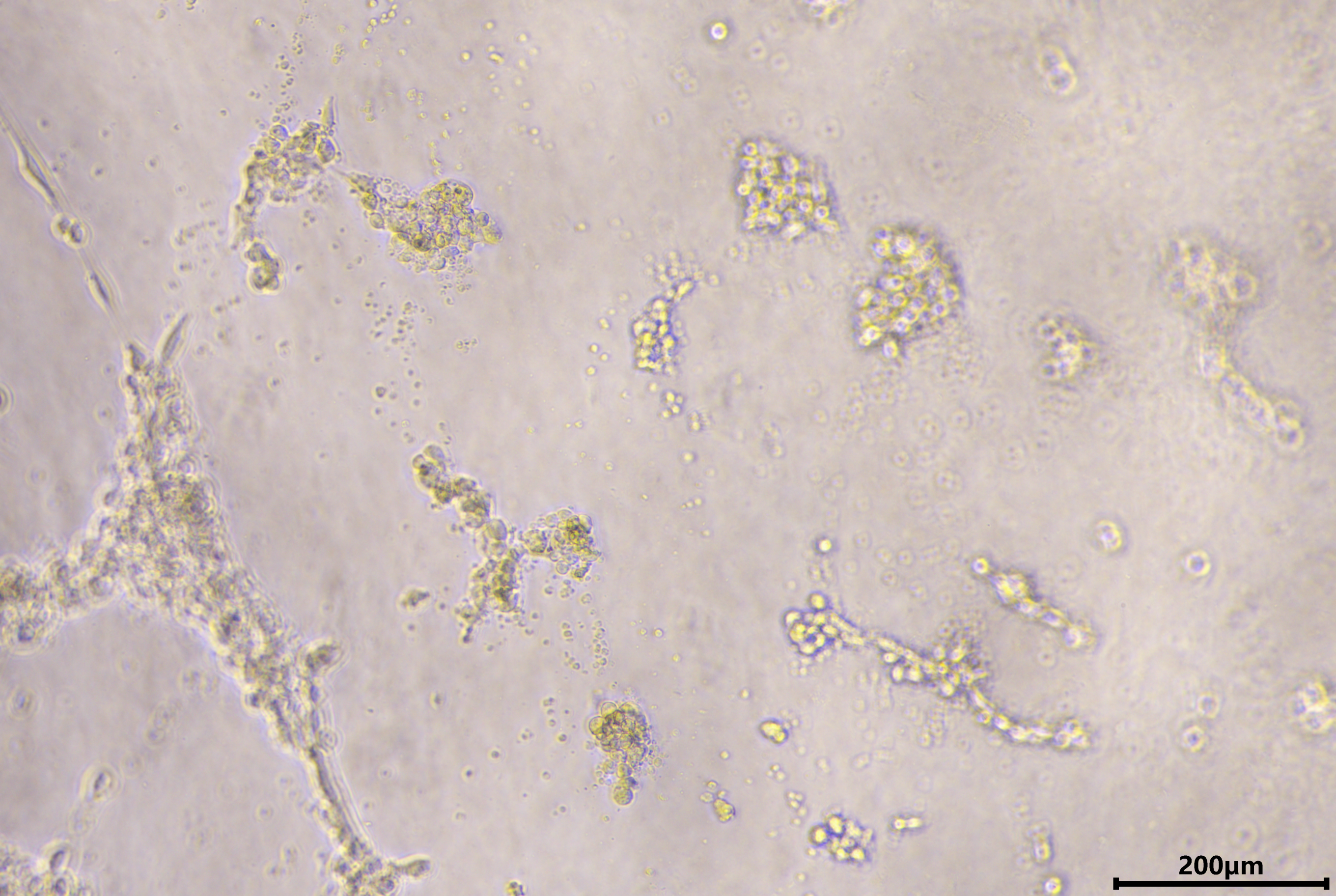

Supplement: Supplementary file 5 — Source data Fig. 3 [file 44319_2025_627_MOESM5_ESM.zip › Figure 3/3K/siVEGF-A+A.f.tiff]

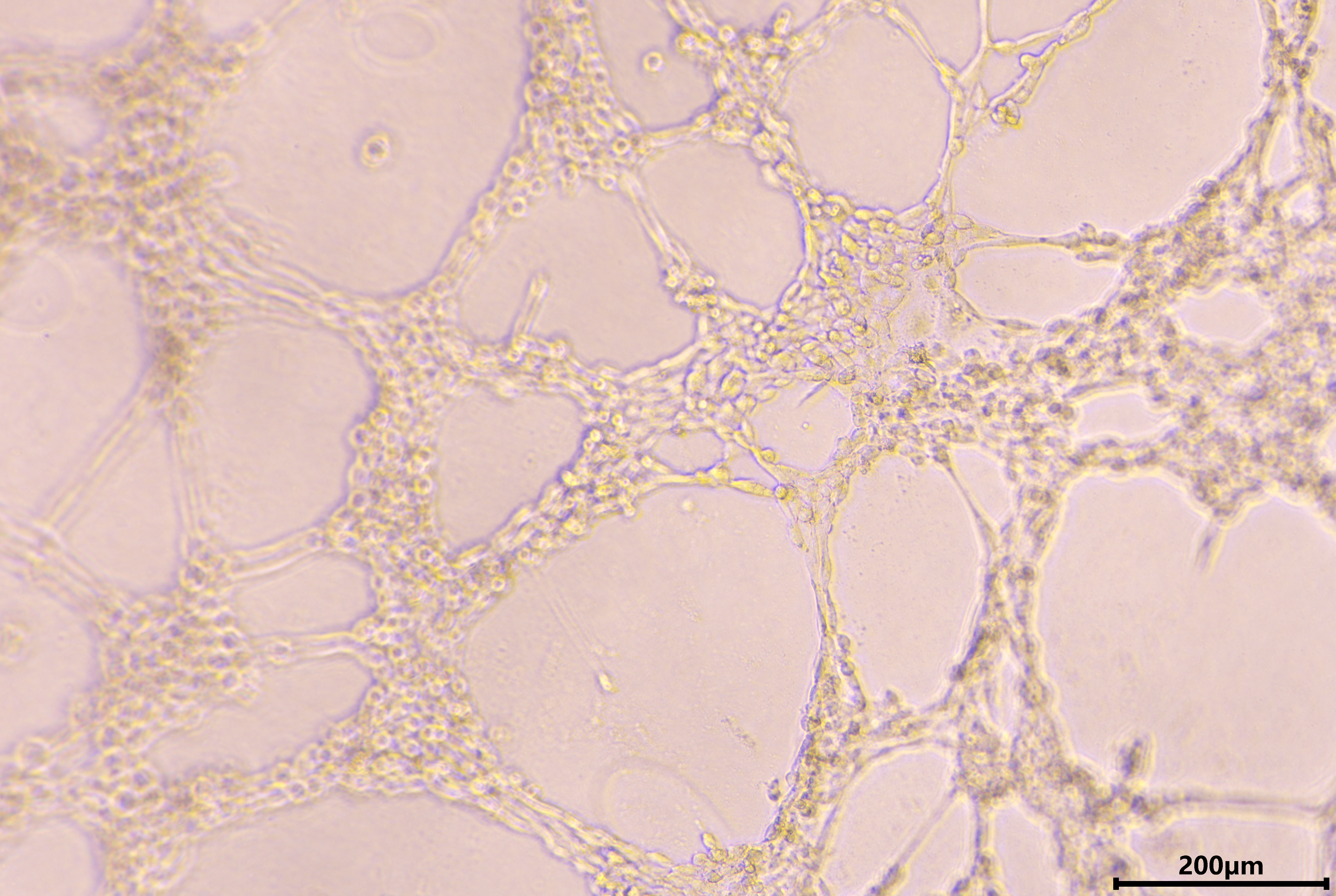

Supplement: Supplementary file 5 — Source data Fig. 3 [file 44319_2025_627_MOESM5_ESM.zip › Figure 3/3L/A.f.png]

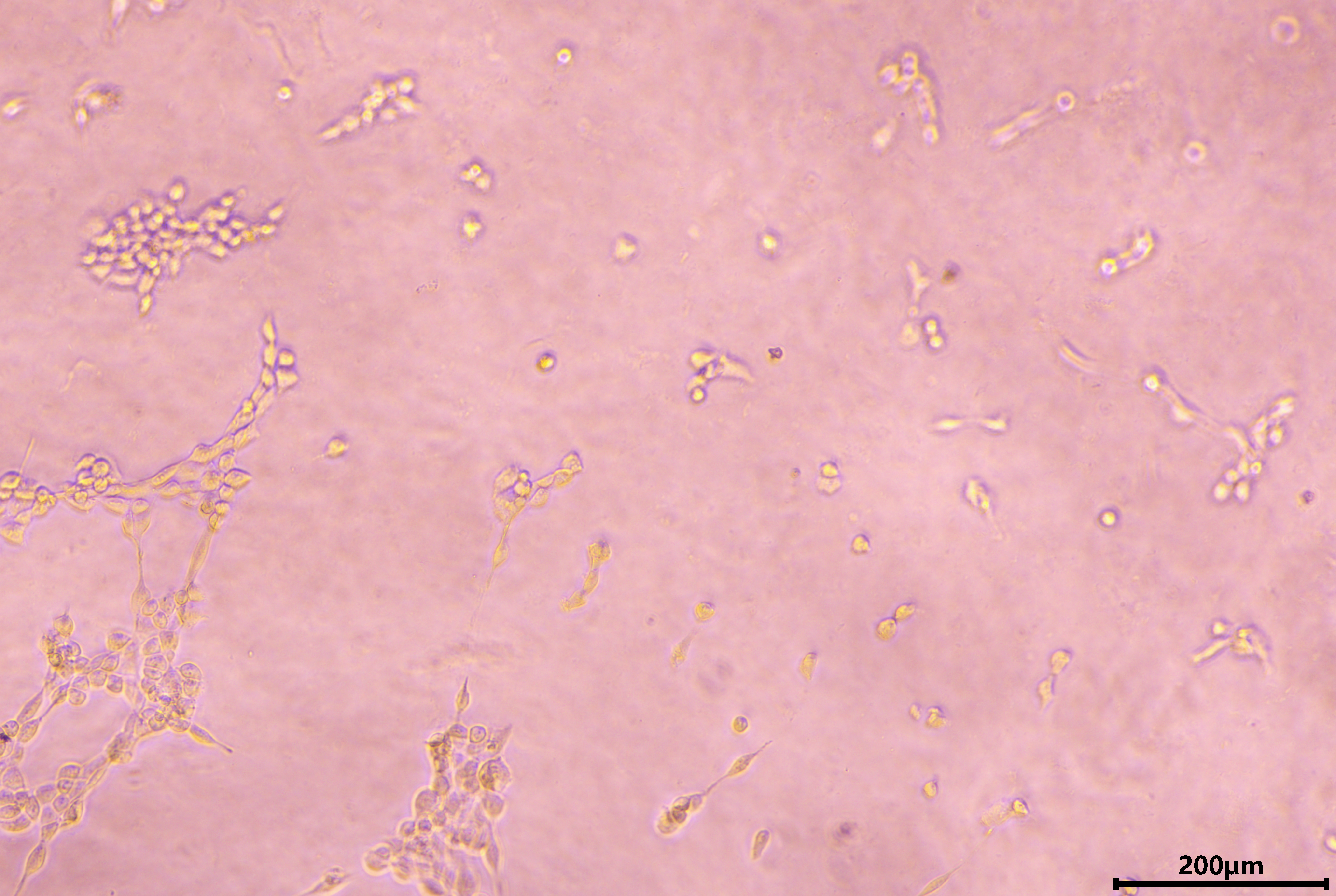

Supplement: Supplementary file 5 — Source data Fig. 3 [file 44319_2025_627_MOESM5_ESM.zip › Figure 3/3L/anti-VEGF-A.tif]

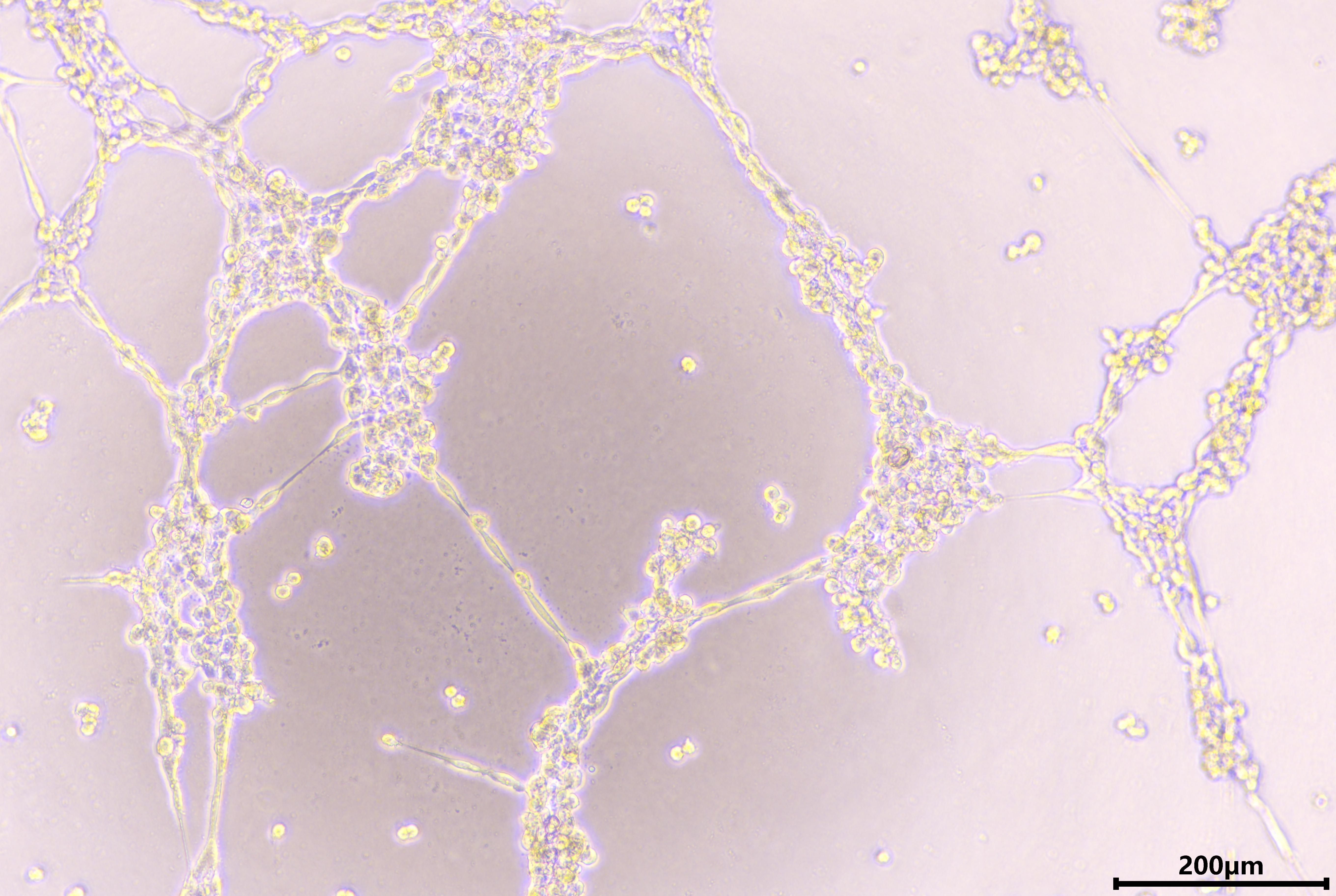

Supplement: Supplementary file 5 — Source data Fig. 3 [file 44319_2025_627_MOESM5_ESM.zip › Figure 3/3L/anti-VEGF-A+A.f.png]

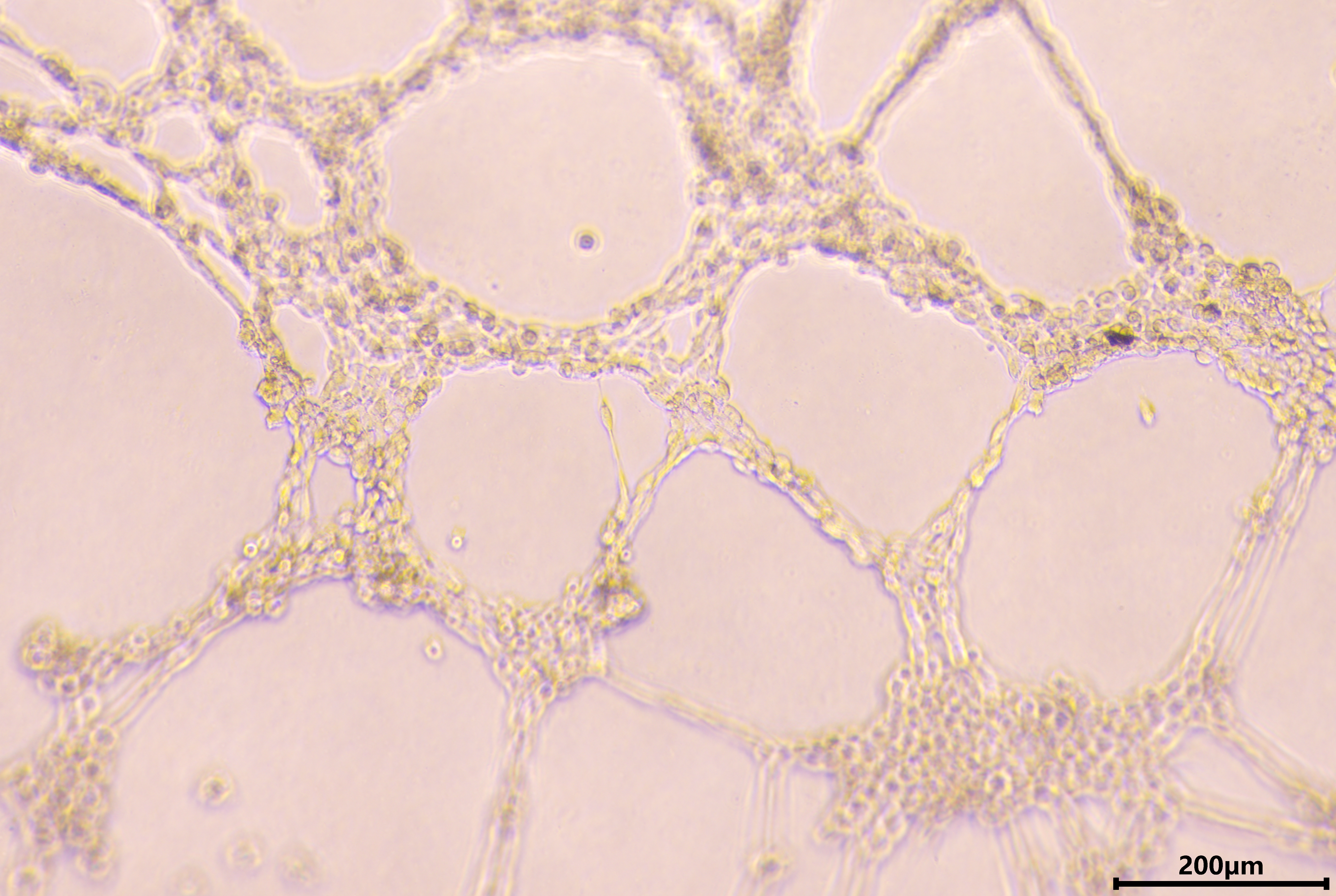

Supplement: Supplementary file 5 — Source data Fig. 3 [file 44319_2025_627_MOESM5_ESM.zip › Figure 3/3L/Ctrl.png]

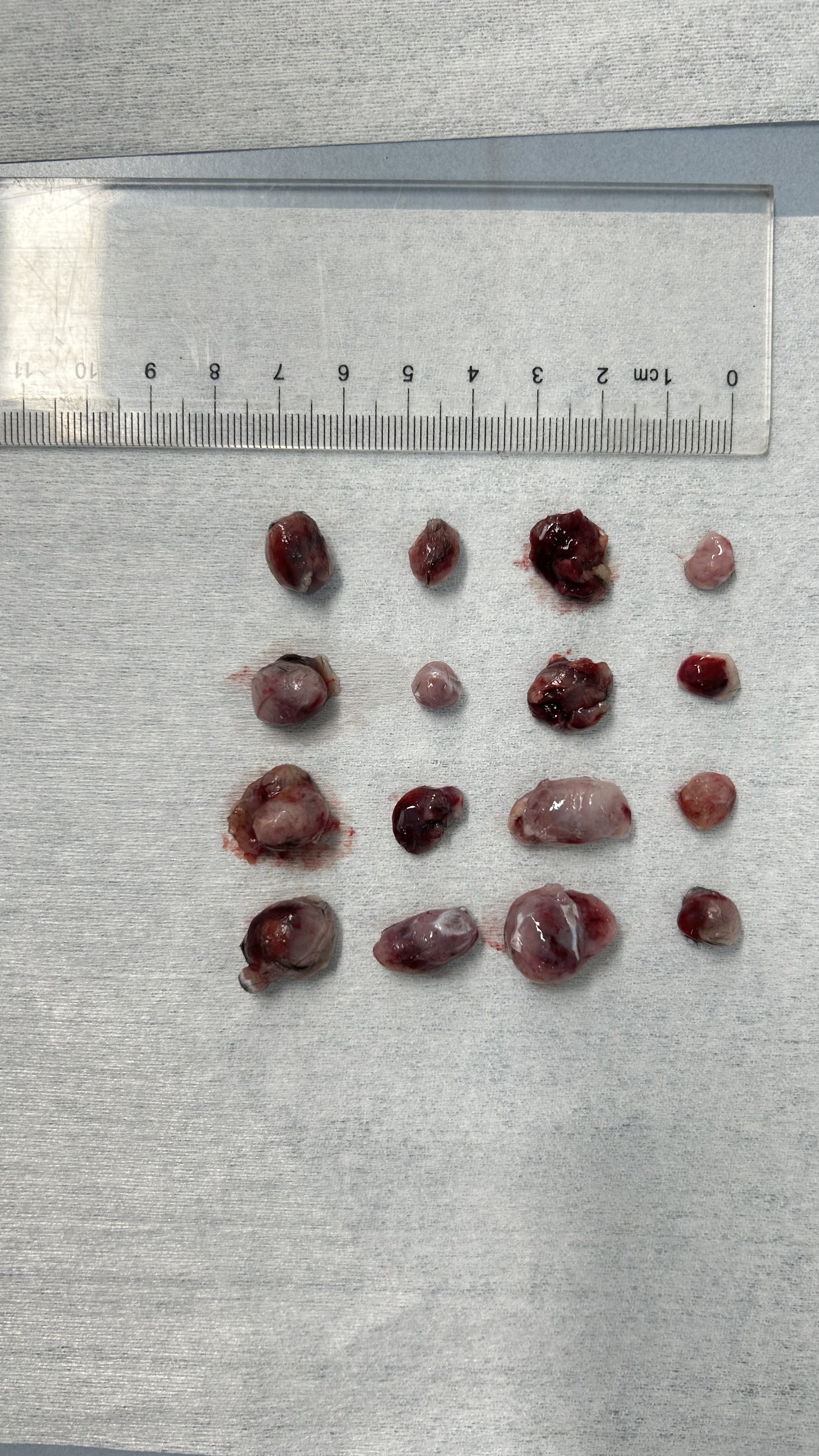

Supplement: Supplementary file 6 — Source data Fig. 4 [file 44319_2025_627_MOESM6_ESM.zip › Figure 4/4C/Tumor.jpg]

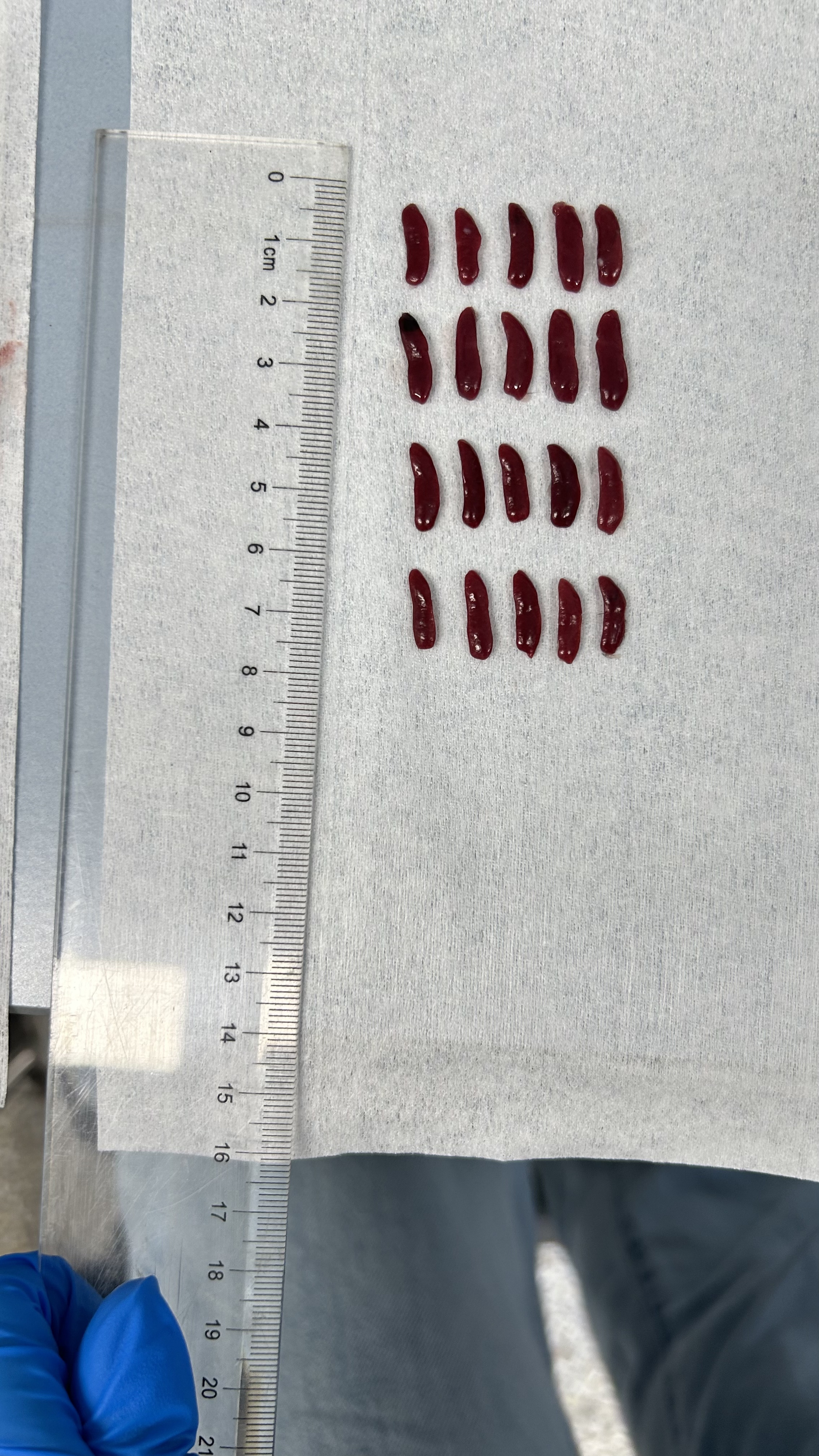

Supplement: Supplementary file 6 — Source data Fig. 4 [file 44319_2025_627_MOESM6_ESM.zip › Figure 4/4E/Spleen.jpg]

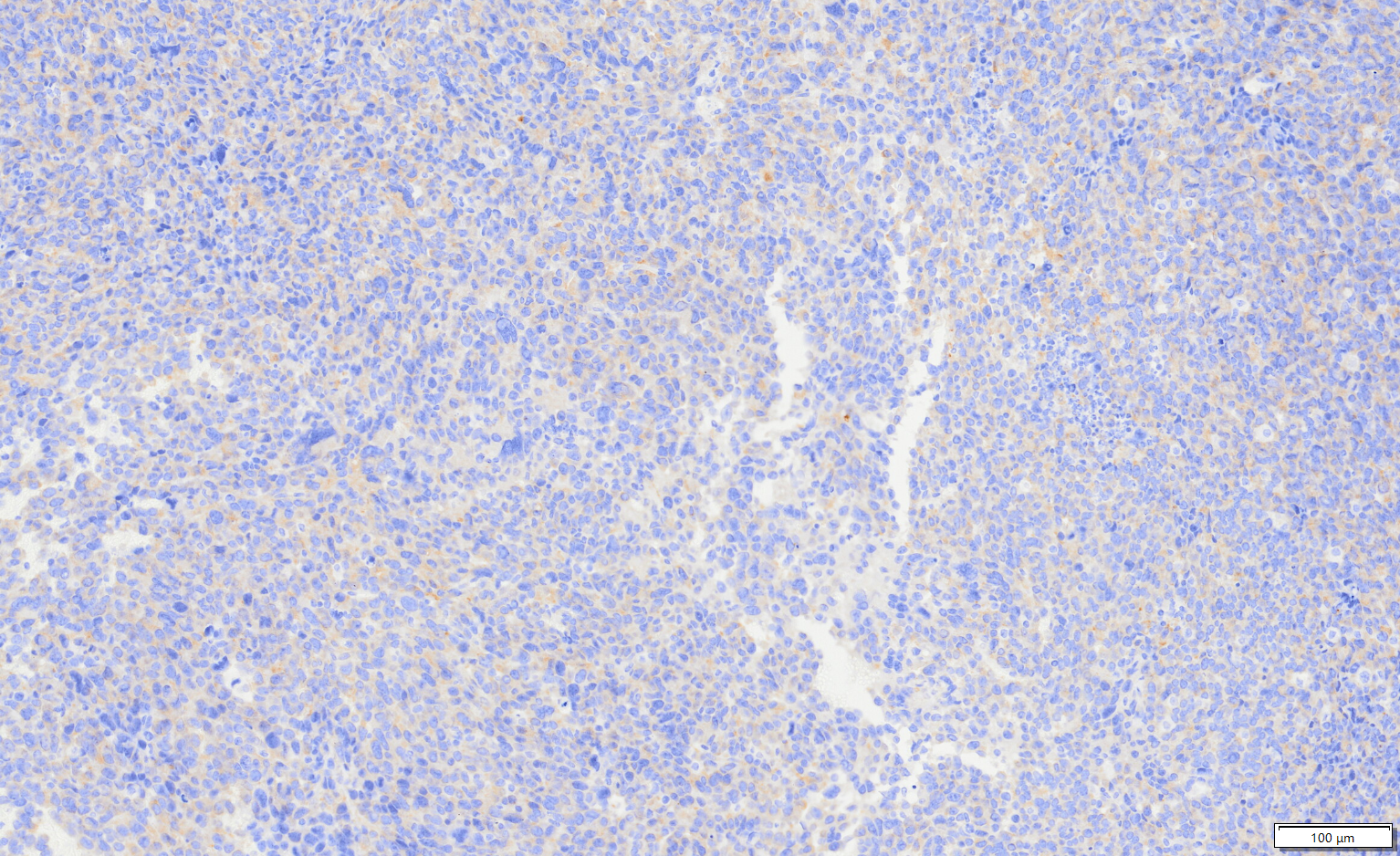

Supplement: Supplementary file 6 — Source data Fig. 4 [file 44319_2025_627_MOESM6_ESM.zip › Figure 4/4H/LLC Ctrl VEGF-A.png]

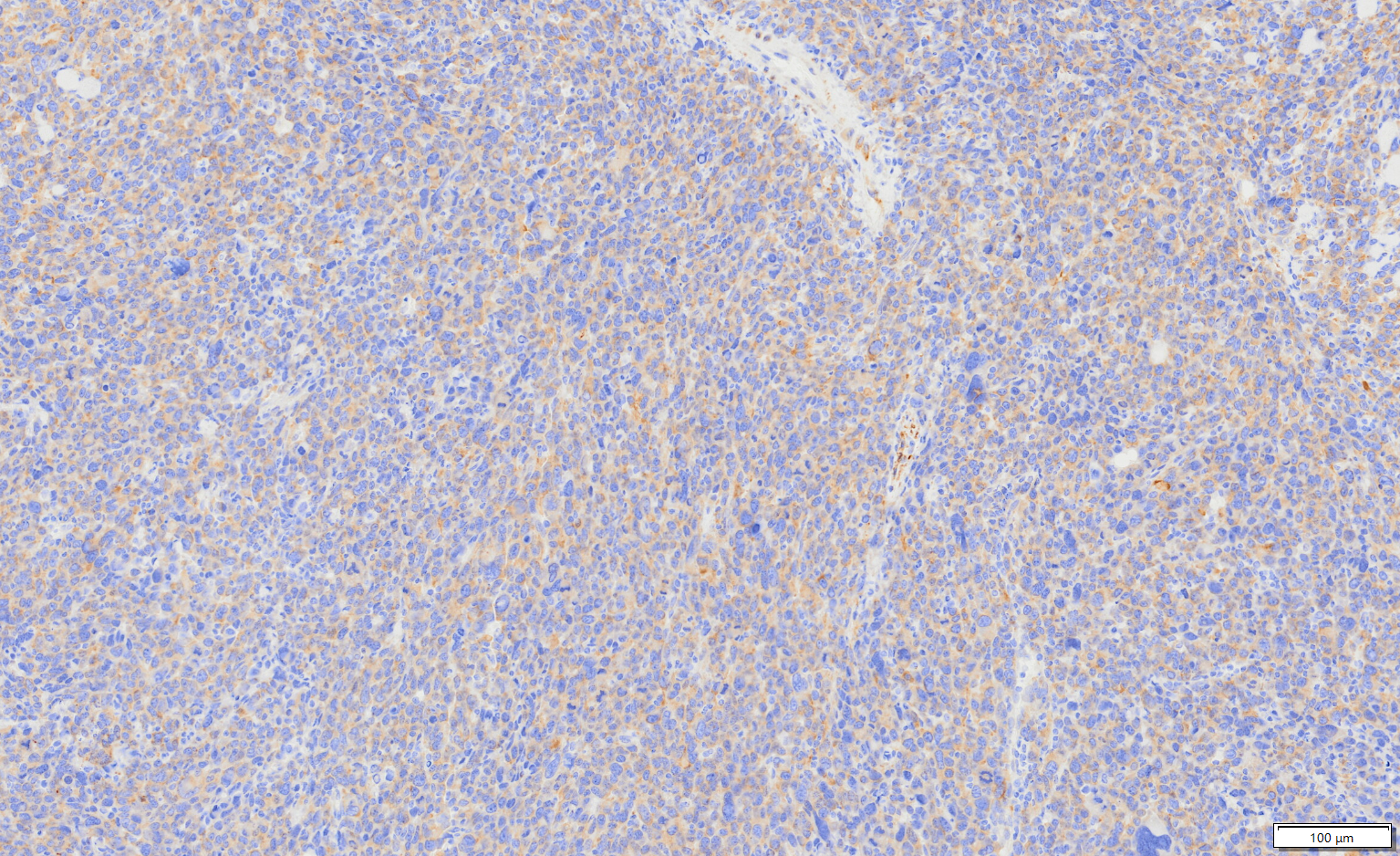

Supplement: Supplementary file 6 — Source data Fig. 4 [file 44319_2025_627_MOESM6_ESM.zip › Figure 4/4H/LLC A.f VEGF-A.png]

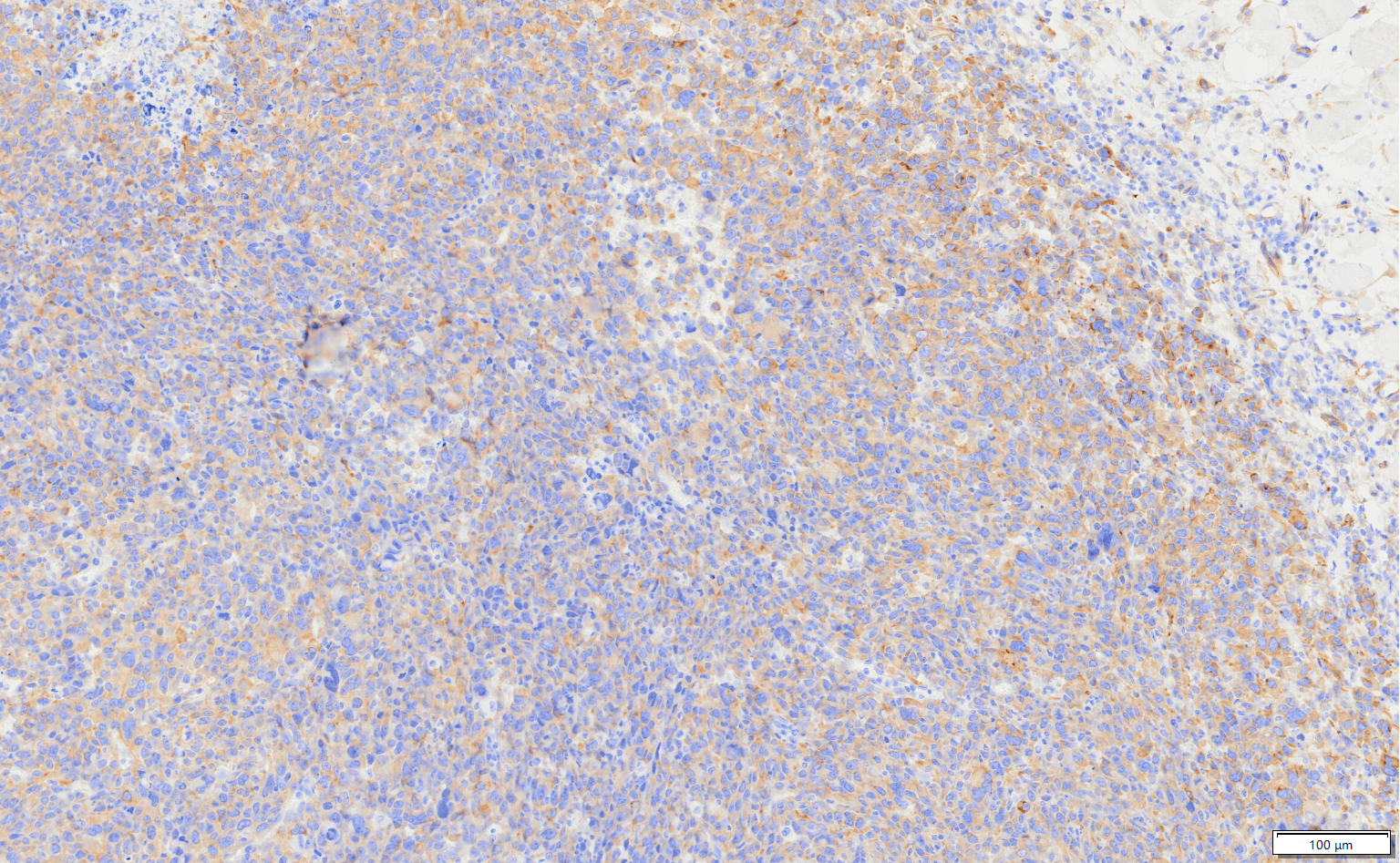

Supplement: Supplementary file 6 — Source data Fig. 4 [file 44319_2025_627_MOESM6_ESM.zip › Figure 4/4H/LLC A.f+IgG VEGF-A.png]

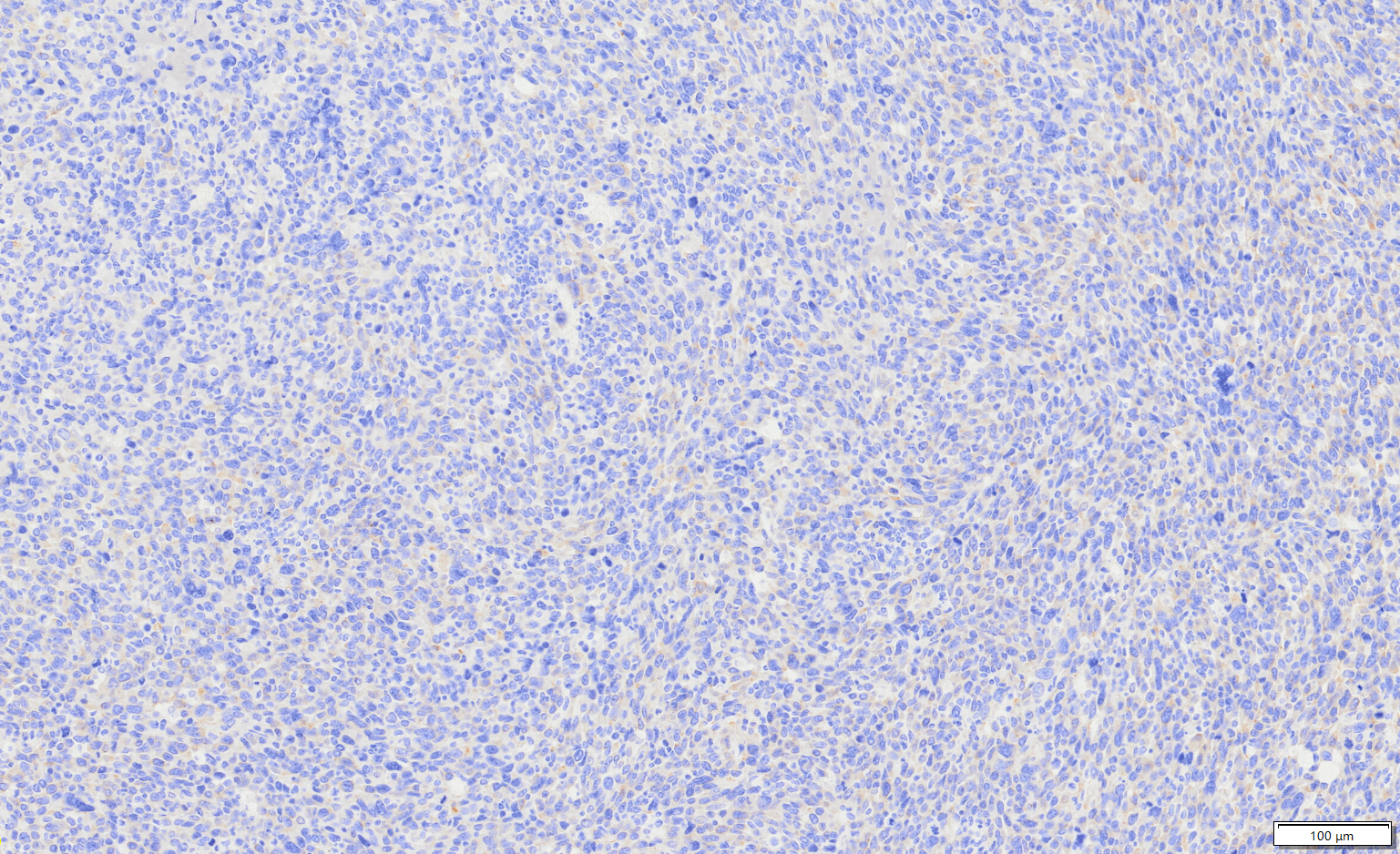

Supplement: Supplementary file 6 — Source data Fig. 4 [file 44319_2025_627_MOESM6_ESM.zip › Figure 4/4H/LLC A.f+ly6G VEGF-A.png]

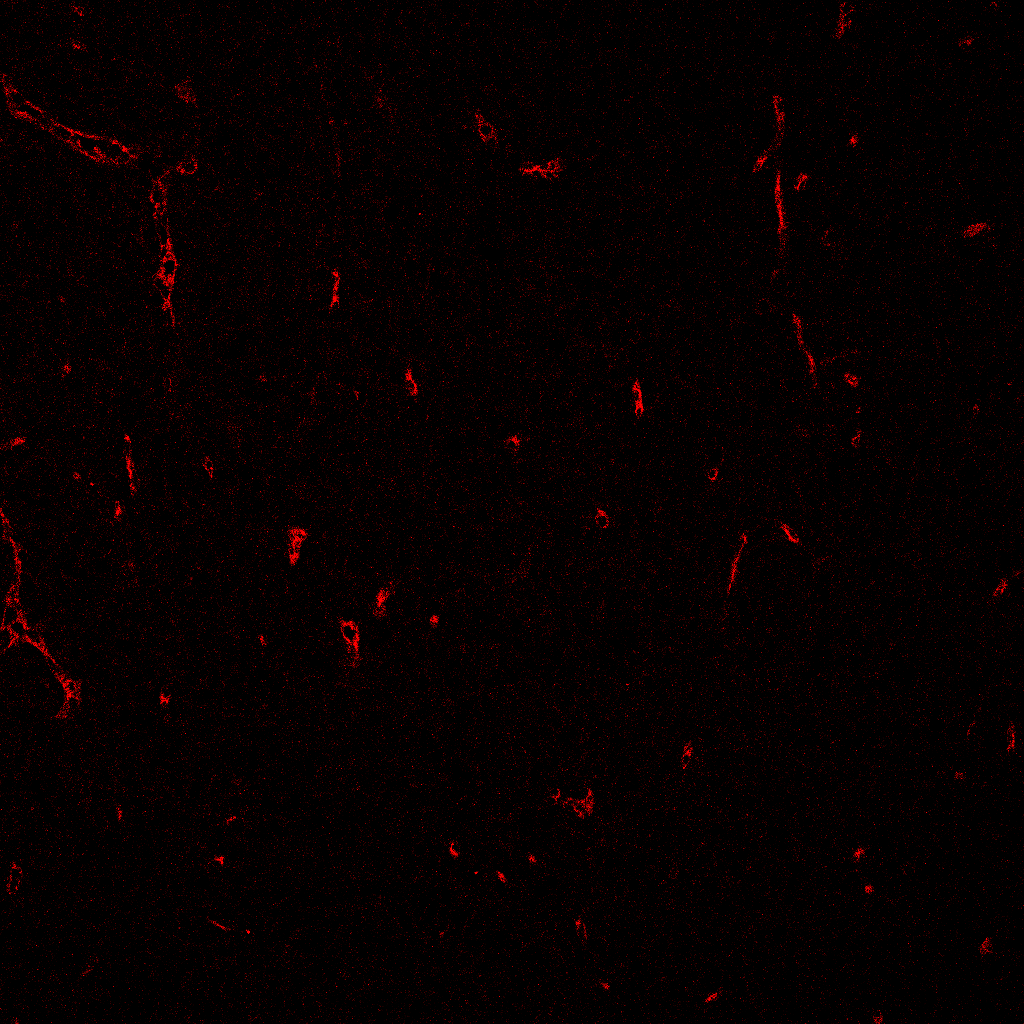

Supplement: Supplementary file 6 — Source data Fig. 4 [file 44319_2025_627_MOESM6_ESM.zip › Figure 4/4J/CD34 LLC Ctrl.tif]

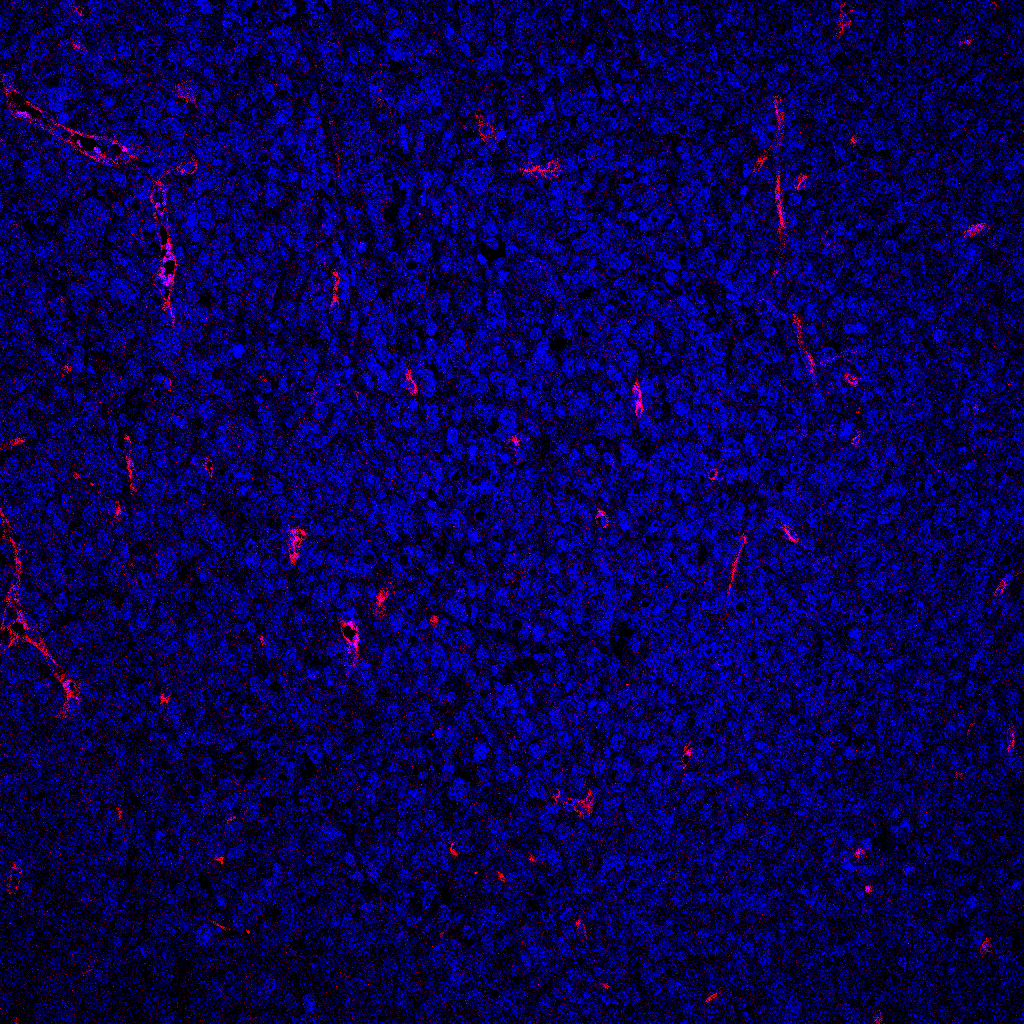

Supplement: Supplementary file 6 — Source data Fig. 4 [file 44319_2025_627_MOESM6_ESM.zip › Figure 4/4J/Merged LLC Ctrl.tif]

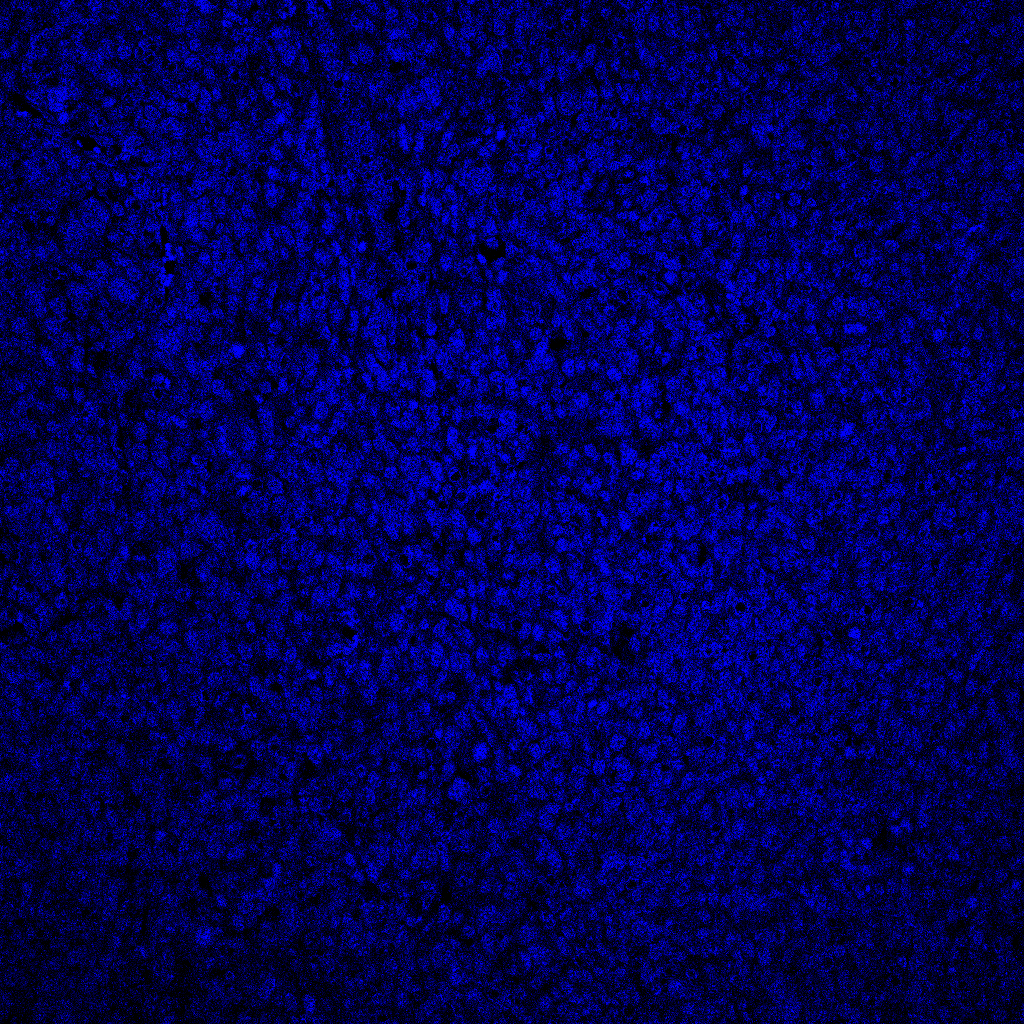

Supplement: Supplementary file 6 — Source data Fig. 4 [file 44319_2025_627_MOESM6_ESM.zip › Figure 4/4J/DAPI LLC Ctrl.tif]

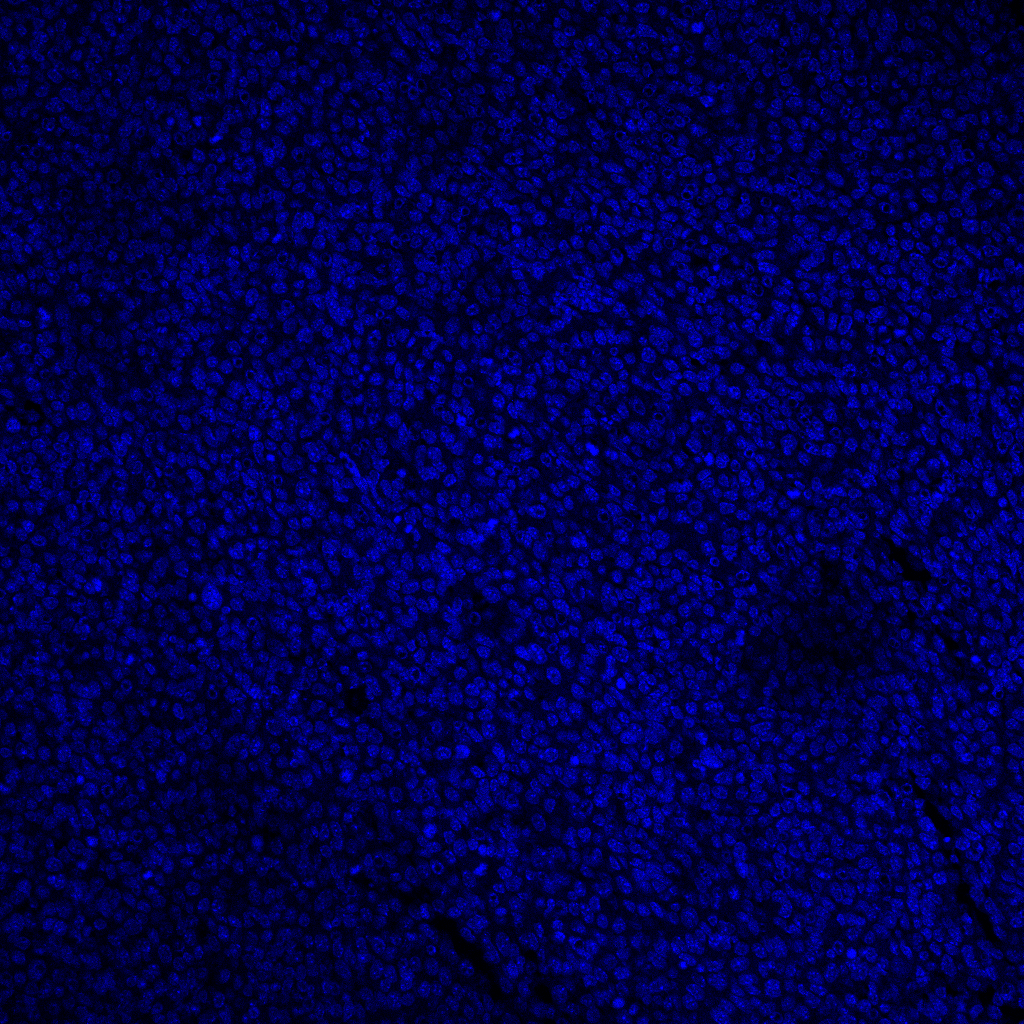

Supplement: Supplementary file 6 — Source data Fig. 4 [file 44319_2025_627_MOESM6_ESM.zip › Figure 4/4J/DAPI LLC A.f.tif]

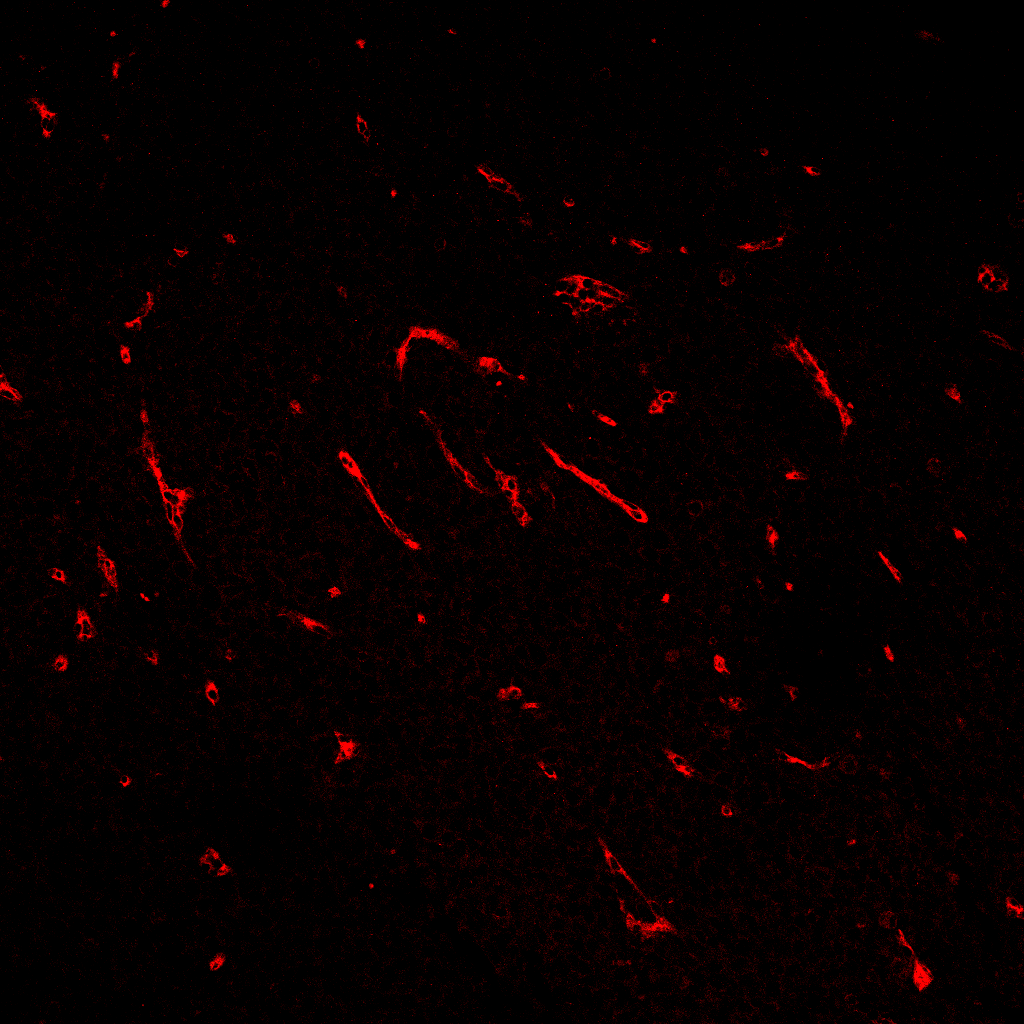

Supplement: Supplementary file 6 — Source data Fig. 4 [file 44319_2025_627_MOESM6_ESM.zip › Figure 4/4J/CD34 LLC A.f.tif]

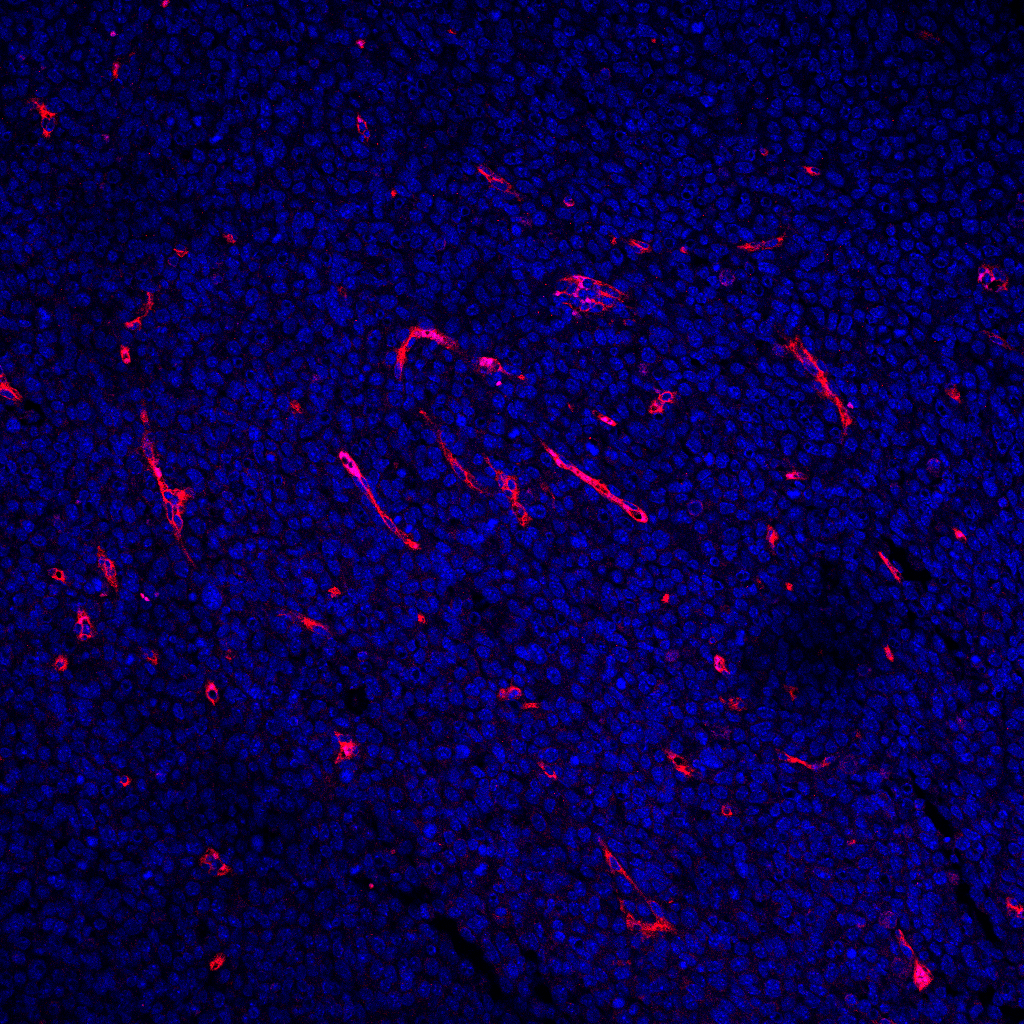

Supplement: Supplementary file 6 — Source data Fig. 4 [file 44319_2025_627_MOESM6_ESM.zip › Figure 4/4J/Merged LLC A.f.tif]

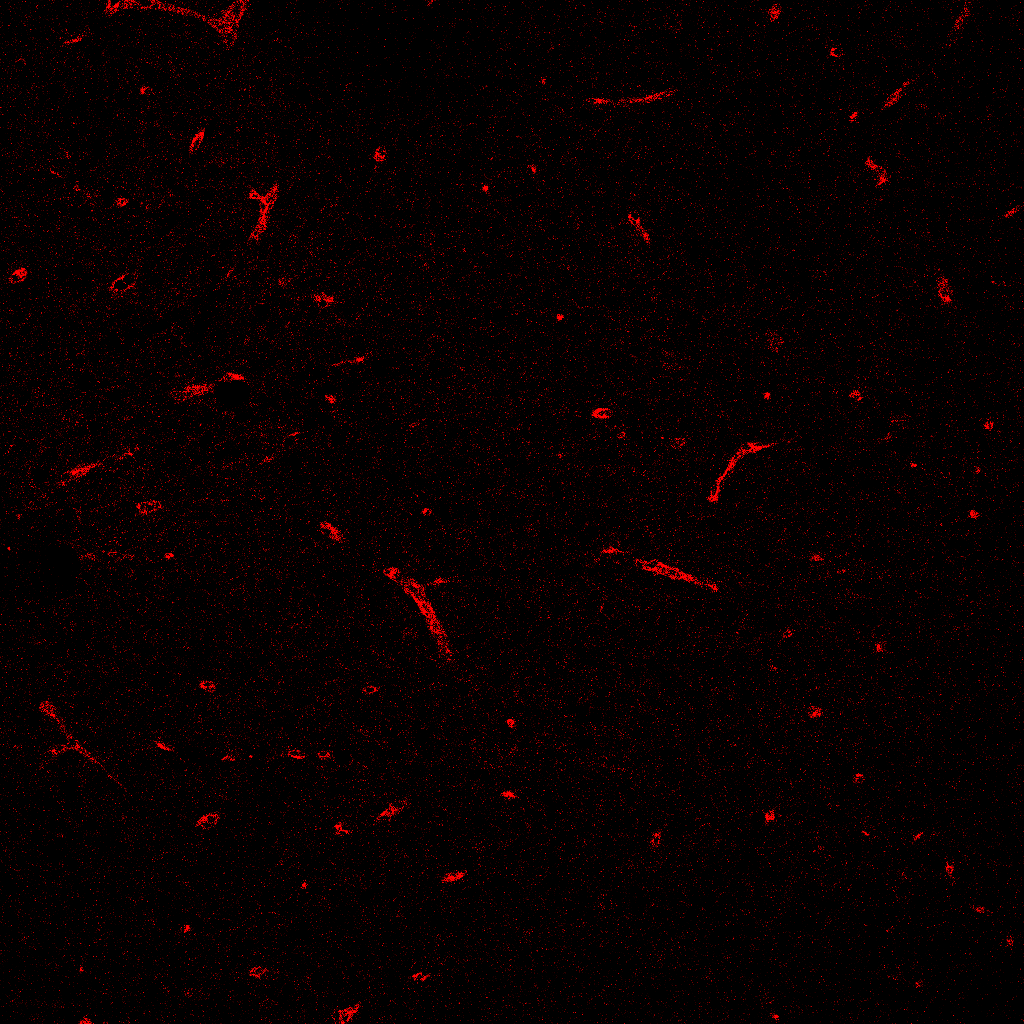

Supplement: Supplementary file 6 — Source data Fig. 4 [file 44319_2025_627_MOESM6_ESM.zip › Figure 4/4J/CD34 LLC A.f+anti-Ly6G.tif]

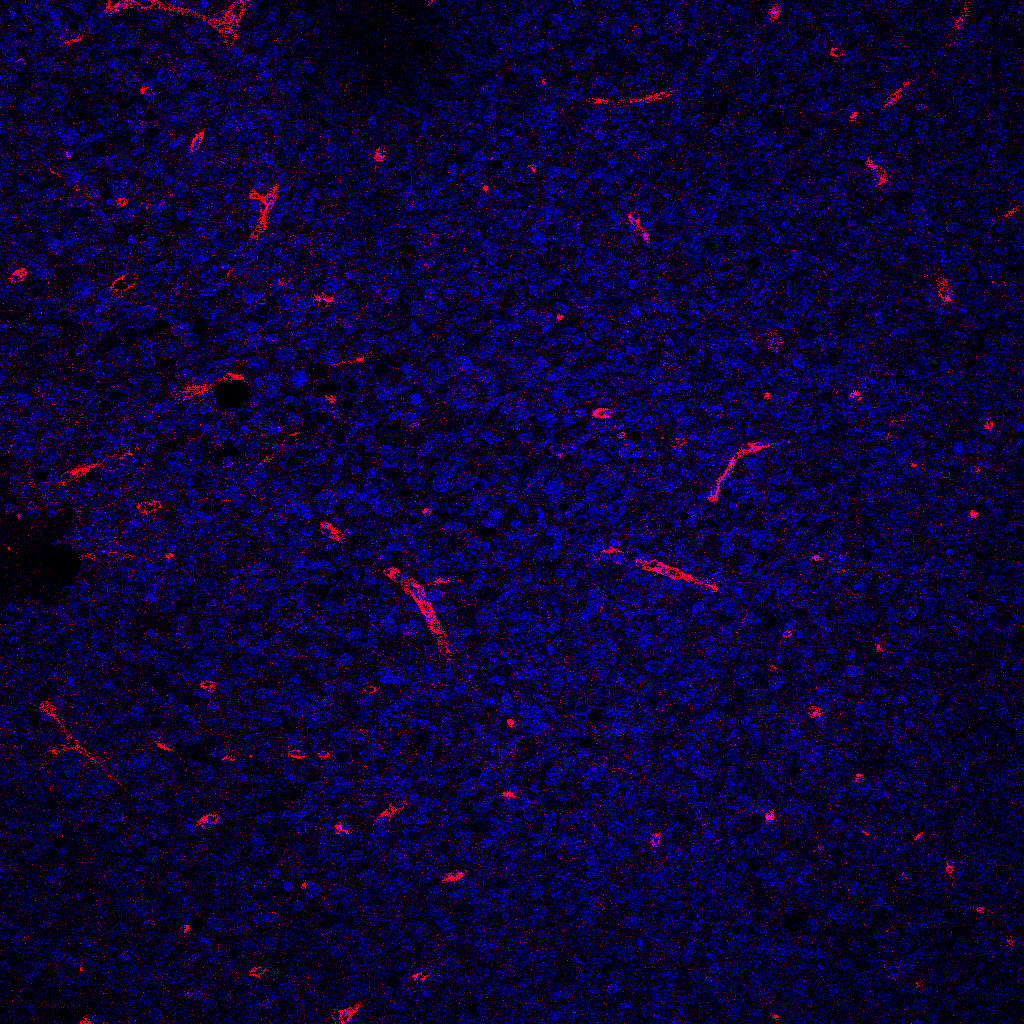

Supplement: Supplementary file 6 — Source data Fig. 4 [file 44319_2025_627_MOESM6_ESM.zip › Figure 4/4J/Merged LLC A.f+anti-Ly6G.tif]

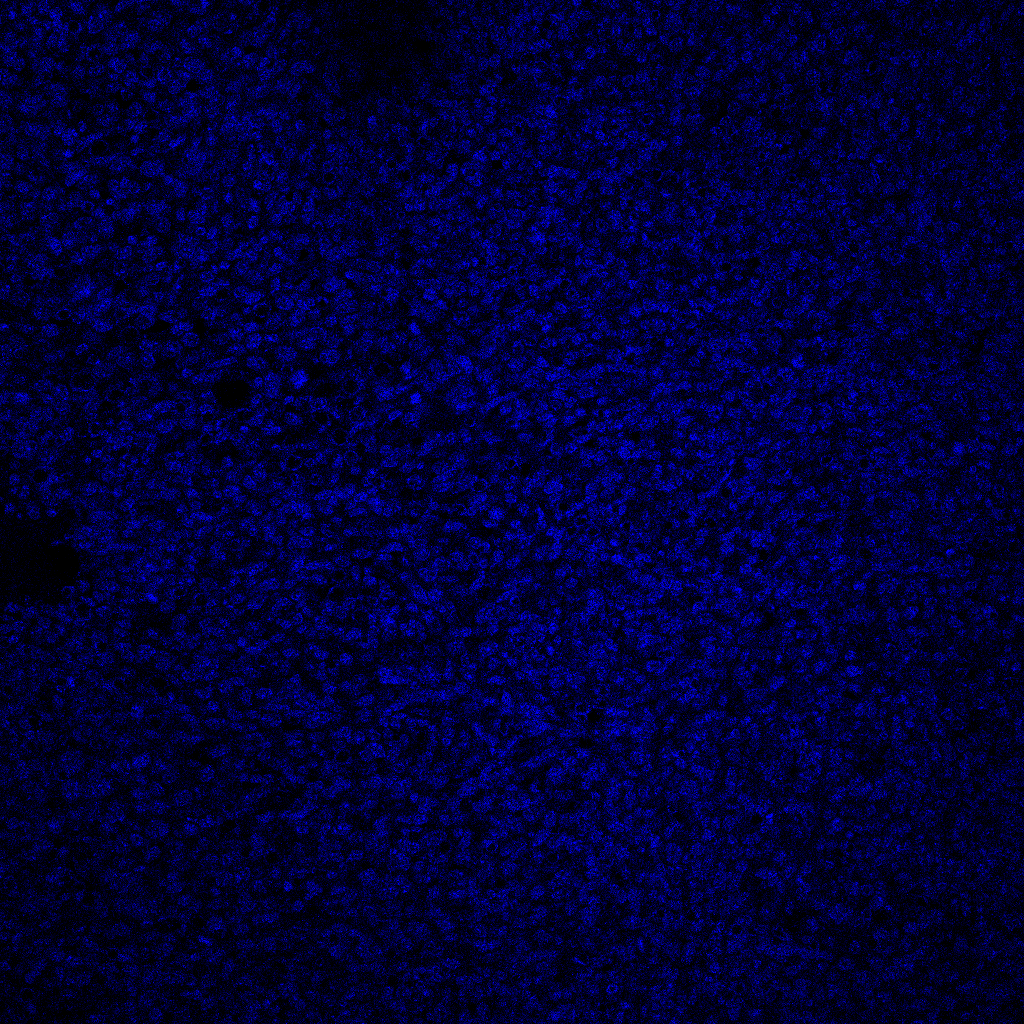

Supplement: Supplementary file 6 — Source data Fig. 4 [file 44319_2025_627_MOESM6_ESM.zip › Figure 4/4J/DAPI LLC A.f+anti-Ly6G.tif]

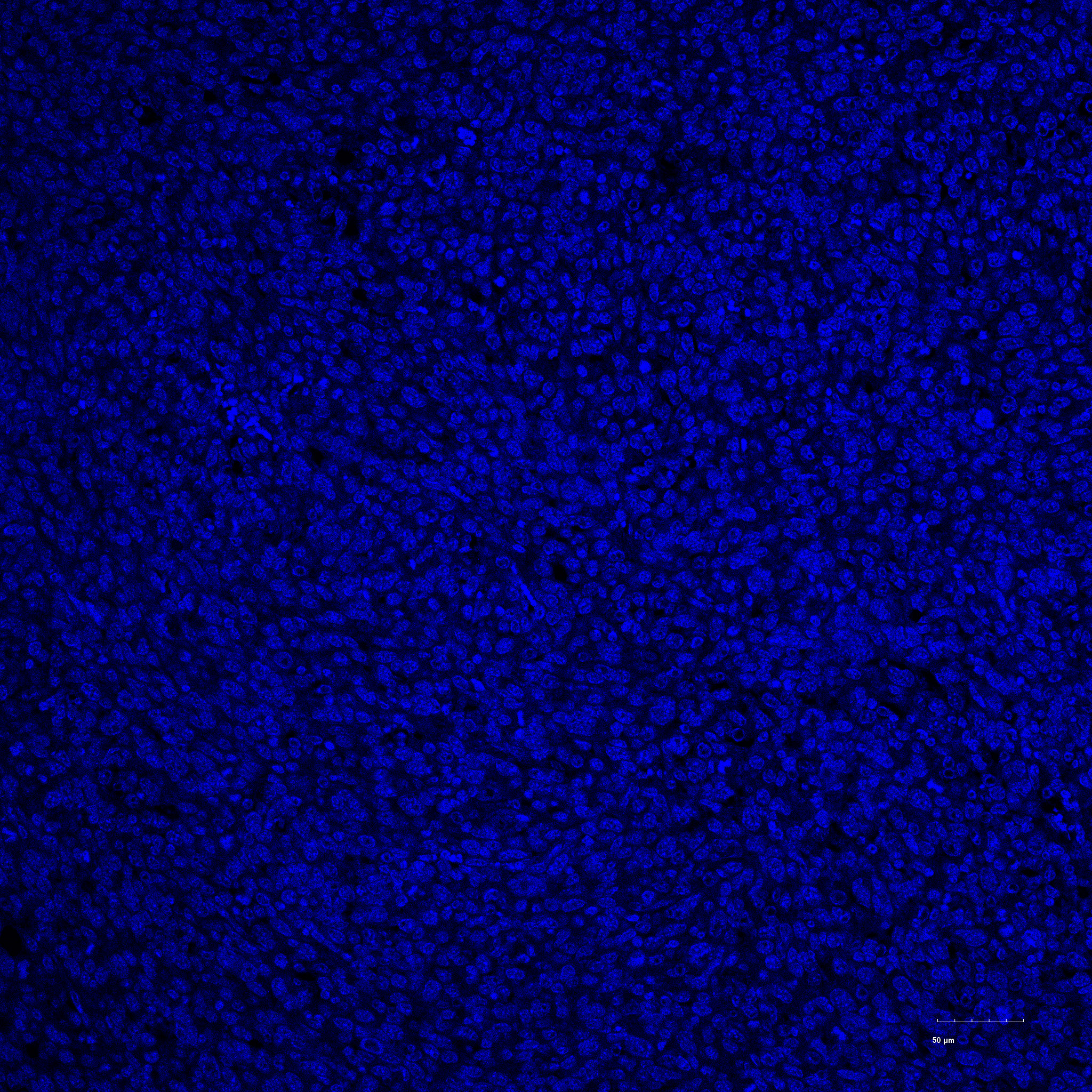

Supplement: Supplementary file 6 — Source data Fig. 4 [file 44319_2025_627_MOESM6_ESM.zip › Figure 4/4J/DAPI LLC A.f+IgG.png]

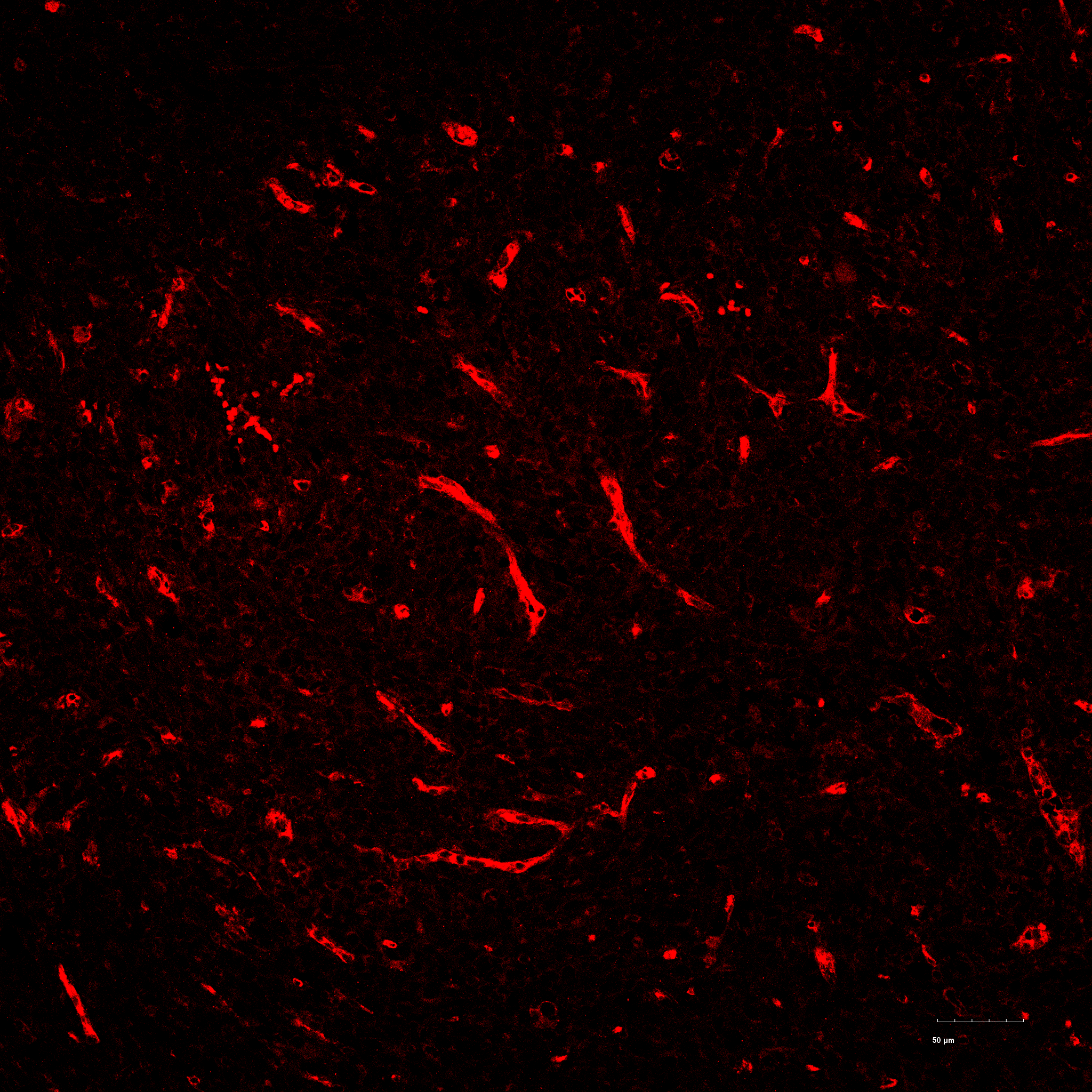

Supplement: Supplementary file 6 — Source data Fig. 4 [file 44319_2025_627_MOESM6_ESM.zip › Figure 4/4J/CD34 LLC A.f+IgG.png]

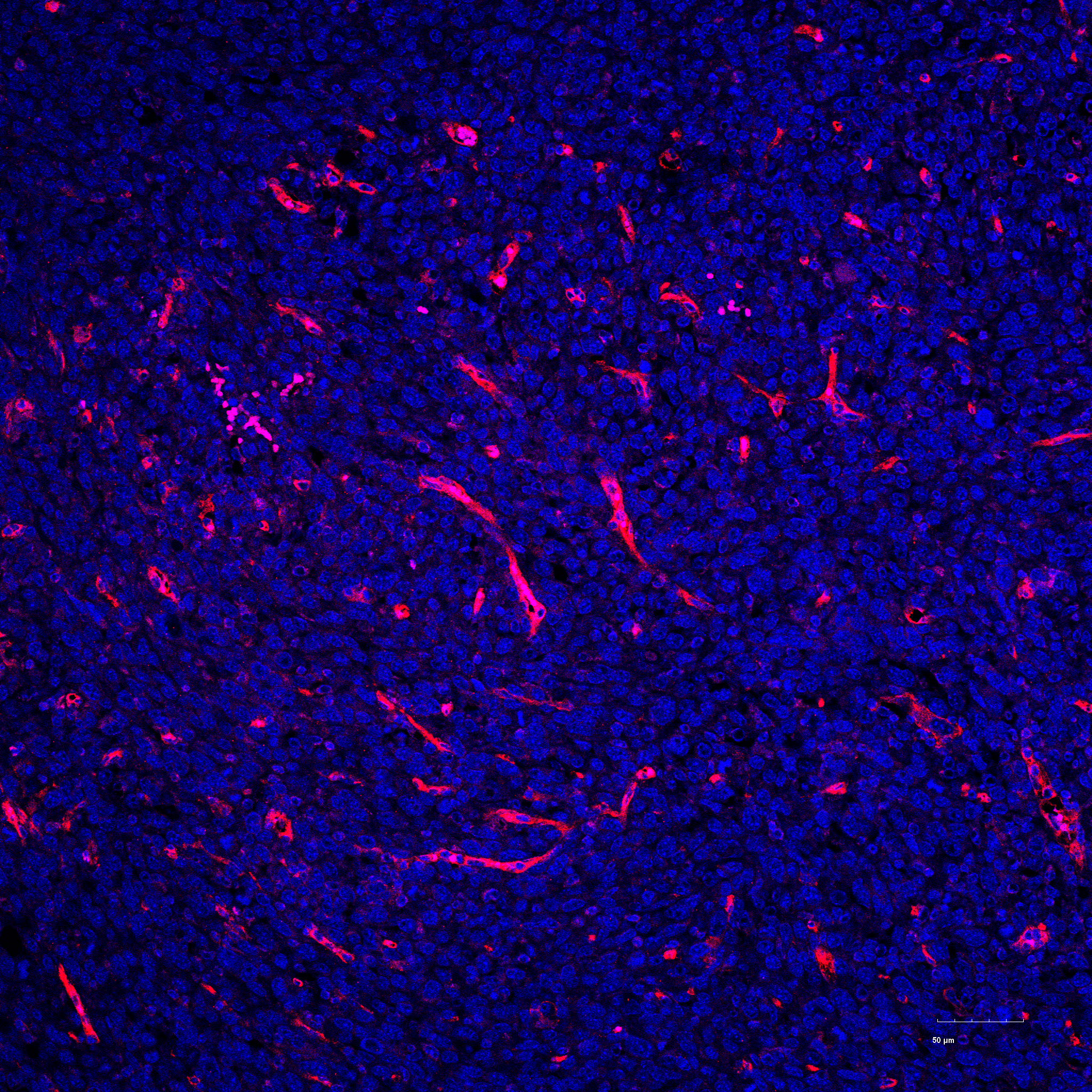

Supplement: Supplementary file 6 — Source data Fig. 4 [file 44319_2025_627_MOESM6_ESM.zip › Figure 4/4J/Merged LLC A.f+IgG.png]

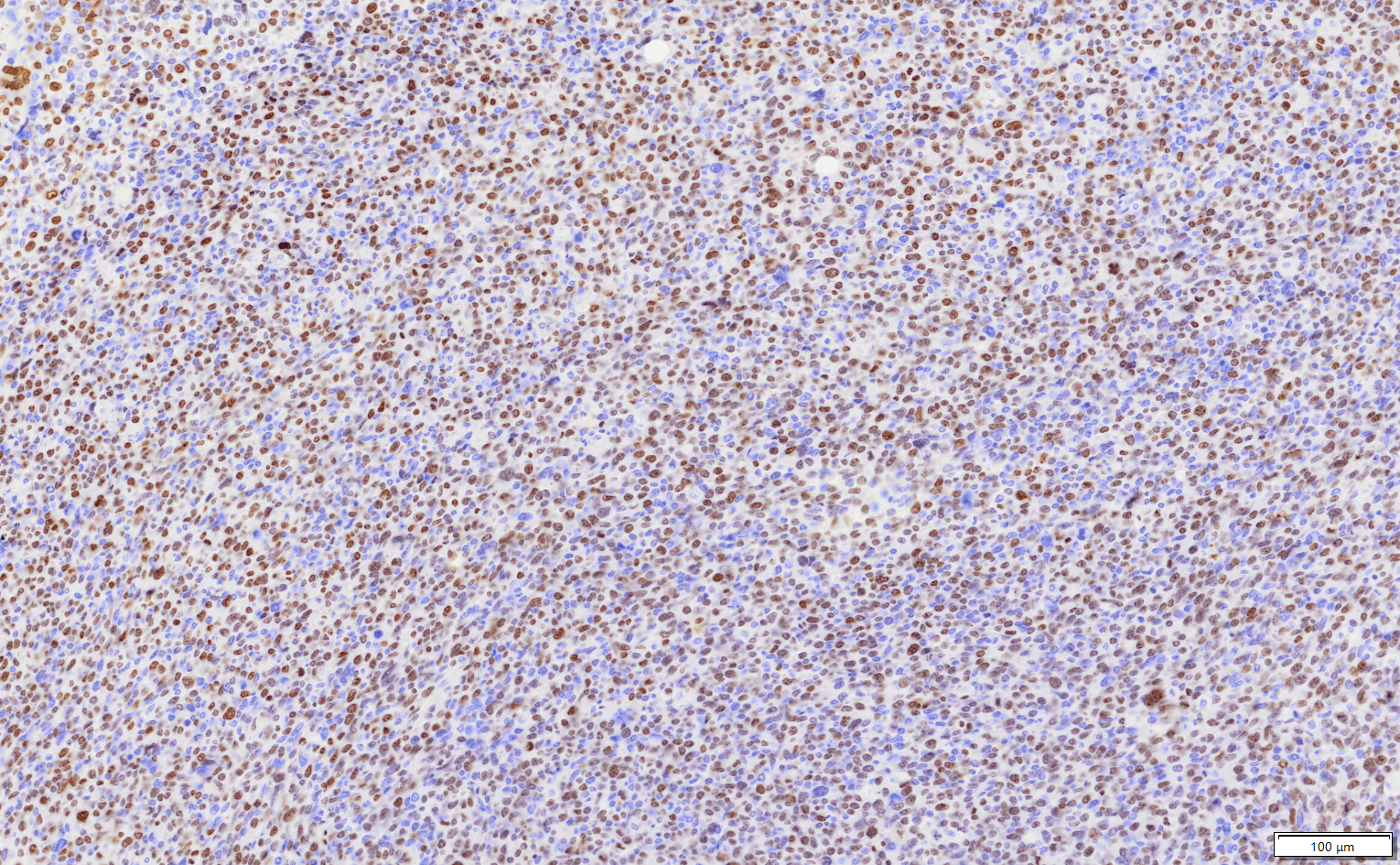

Supplement: Supplementary file 6 — Source data Fig. 4 [file 44319_2025_627_MOESM6_ESM.zip › Figure 4/4G/LLC A.f+anti-lgg ki67.png]

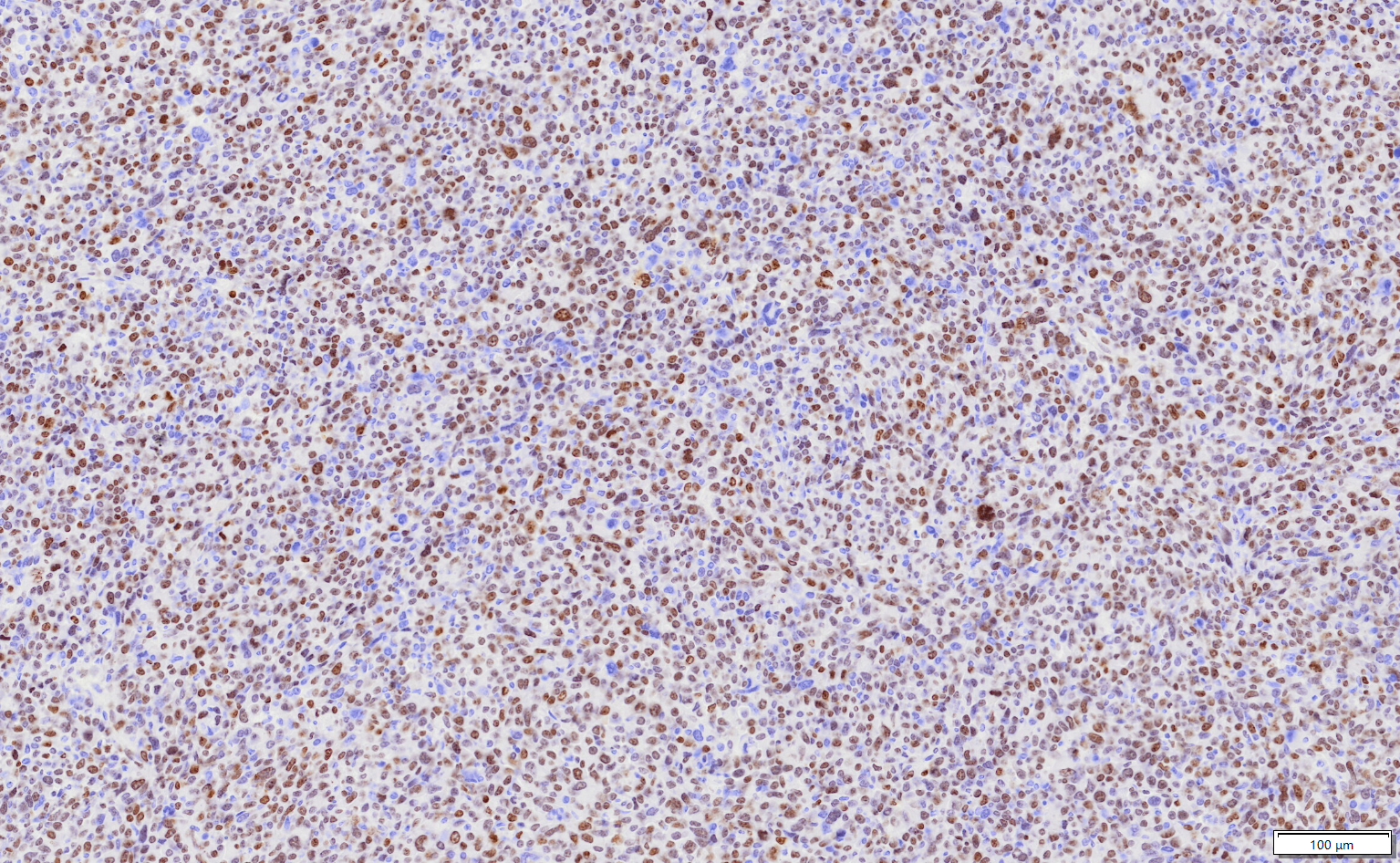

Supplement: Supplementary file 6 — Source data Fig. 4 [file 44319_2025_627_MOESM6_ESM.zip › Figure 4/4G/LLC A.f ki67.png]

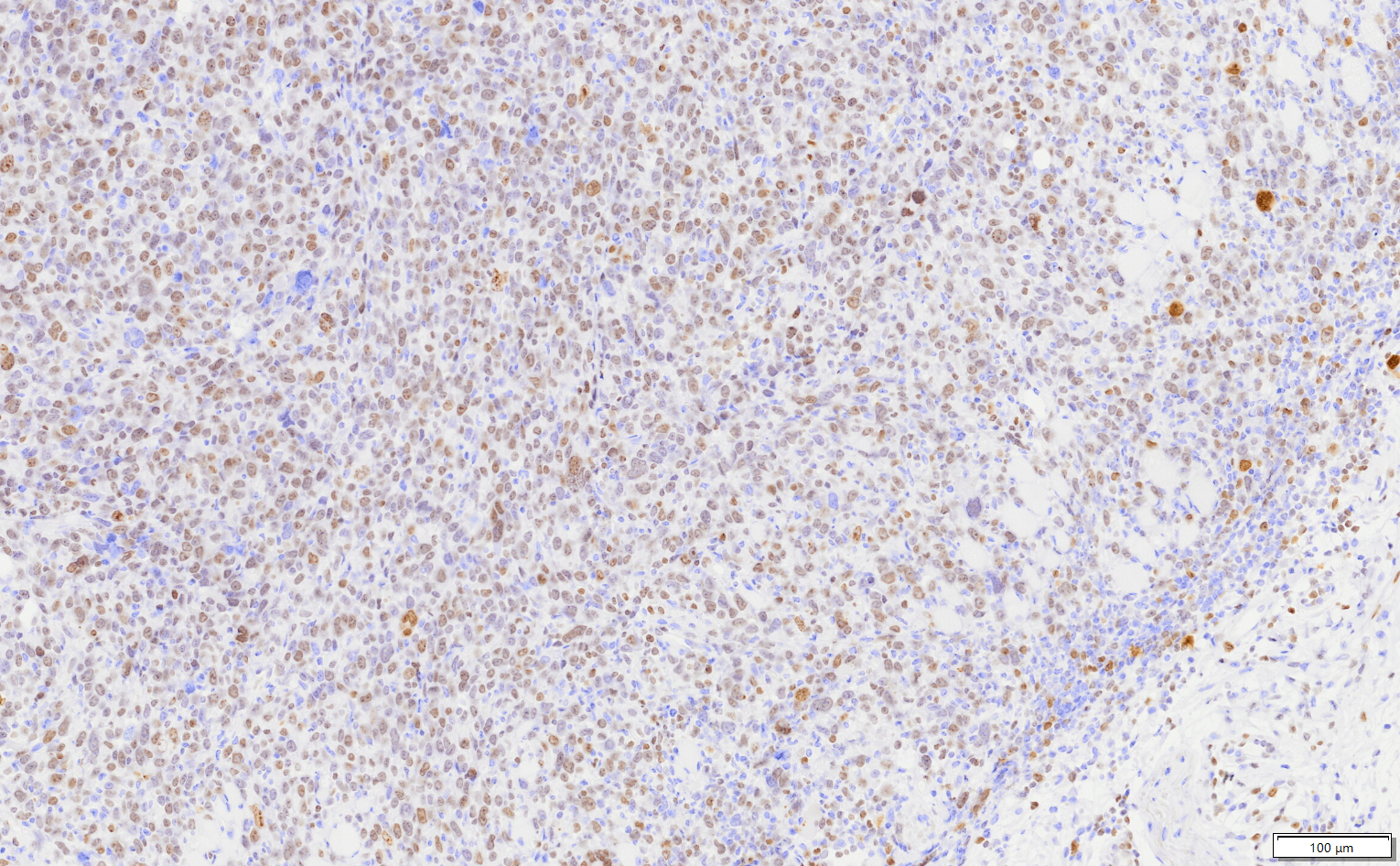

Supplement: Supplementary file 6 — Source data Fig. 4 [file 44319_2025_627_MOESM6_ESM.zip › Figure 4/4G/LLC A.f+anti-ly6g ki67.png]

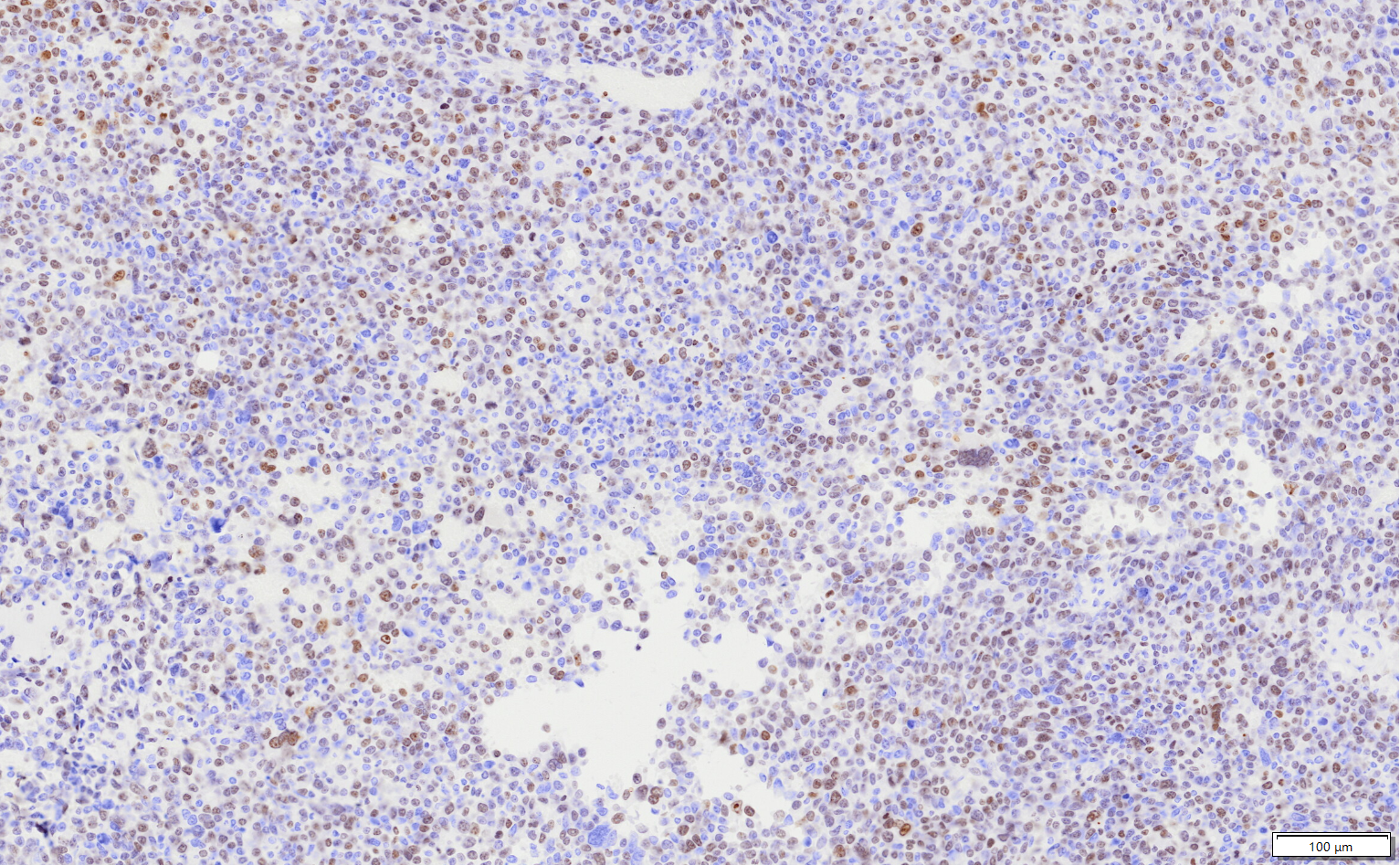

Supplement: Supplementary file 6 — Source data Fig. 4 [file 44319_2025_627_MOESM6_ESM.zip › Figure 4/4G/LLC Ctrl ki67.png]

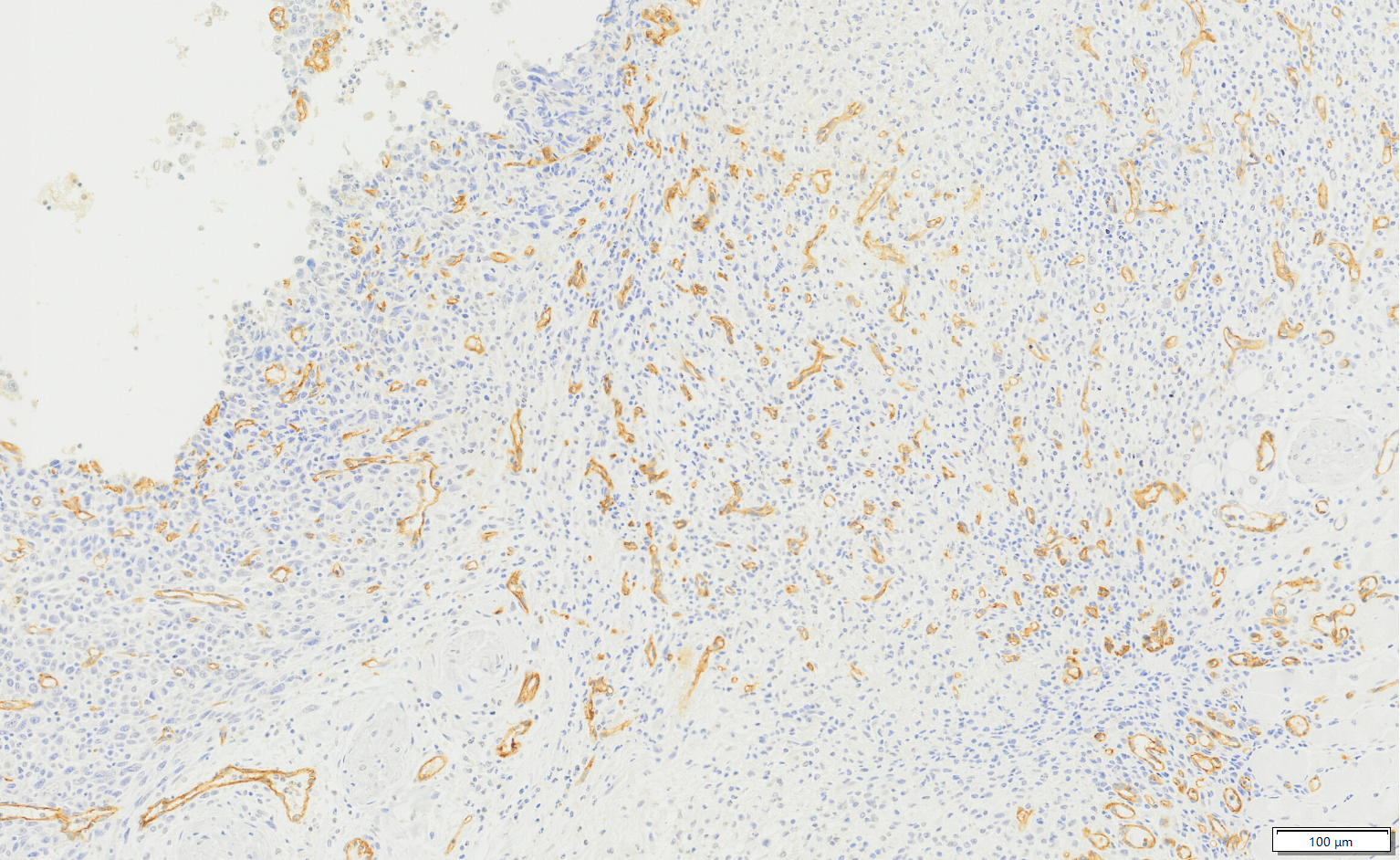

Supplement: Supplementary file 6 — Source data Fig. 4 [file 44319_2025_627_MOESM6_ESM.zip › Figure 4/4I/3 LLC A.f CD31.png]

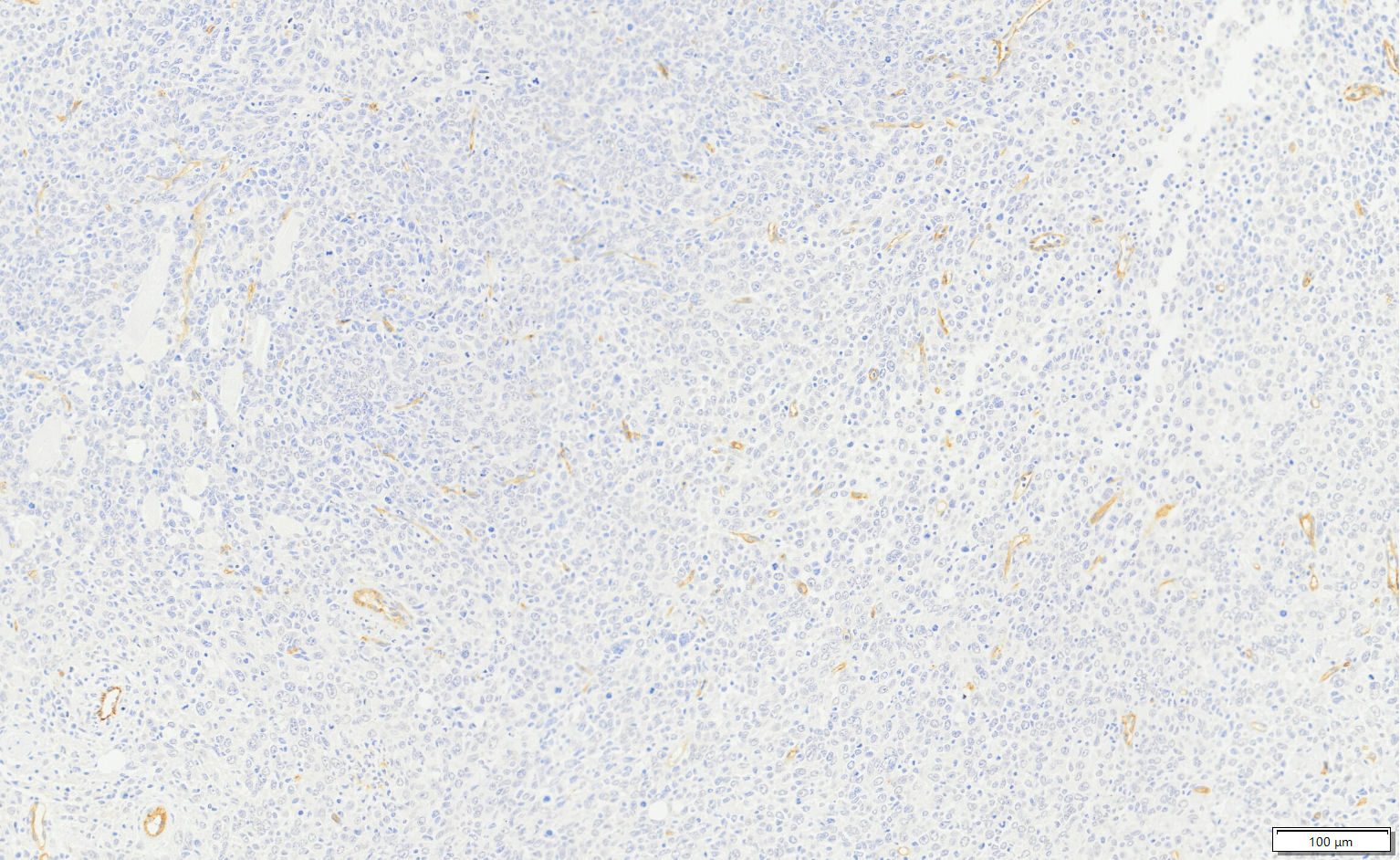

Supplement: Supplementary file 6 — Source data Fig. 4 [file 44319_2025_627_MOESM6_ESM.zip › Figure 4/4I/3 LLC Ctrl CD31.png]

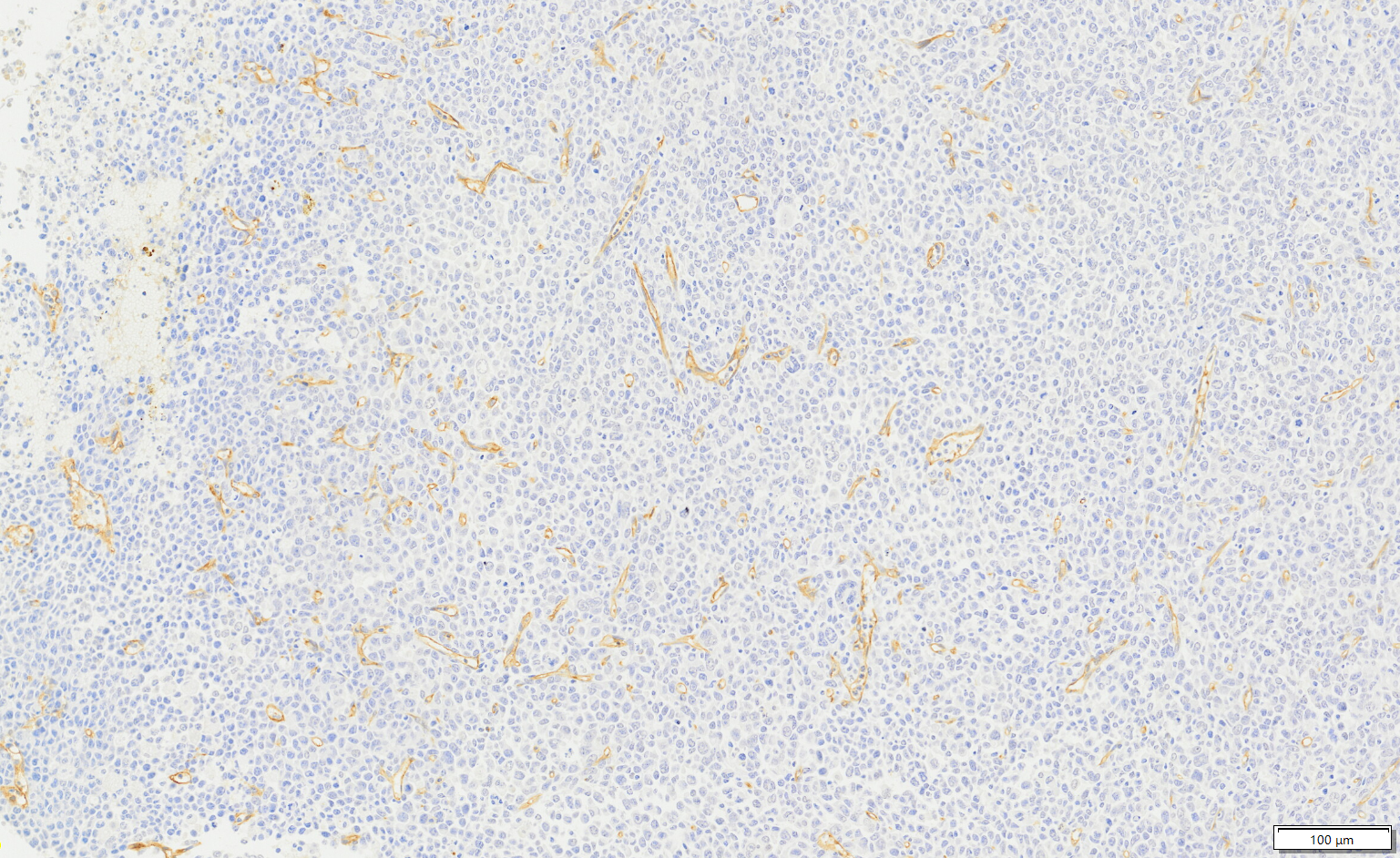

Supplement: Supplementary file 6 — Source data Fig. 4 [file 44319_2025_627_MOESM6_ESM.zip › Figure 4/4I/LLC A.f IgG CD31.png]

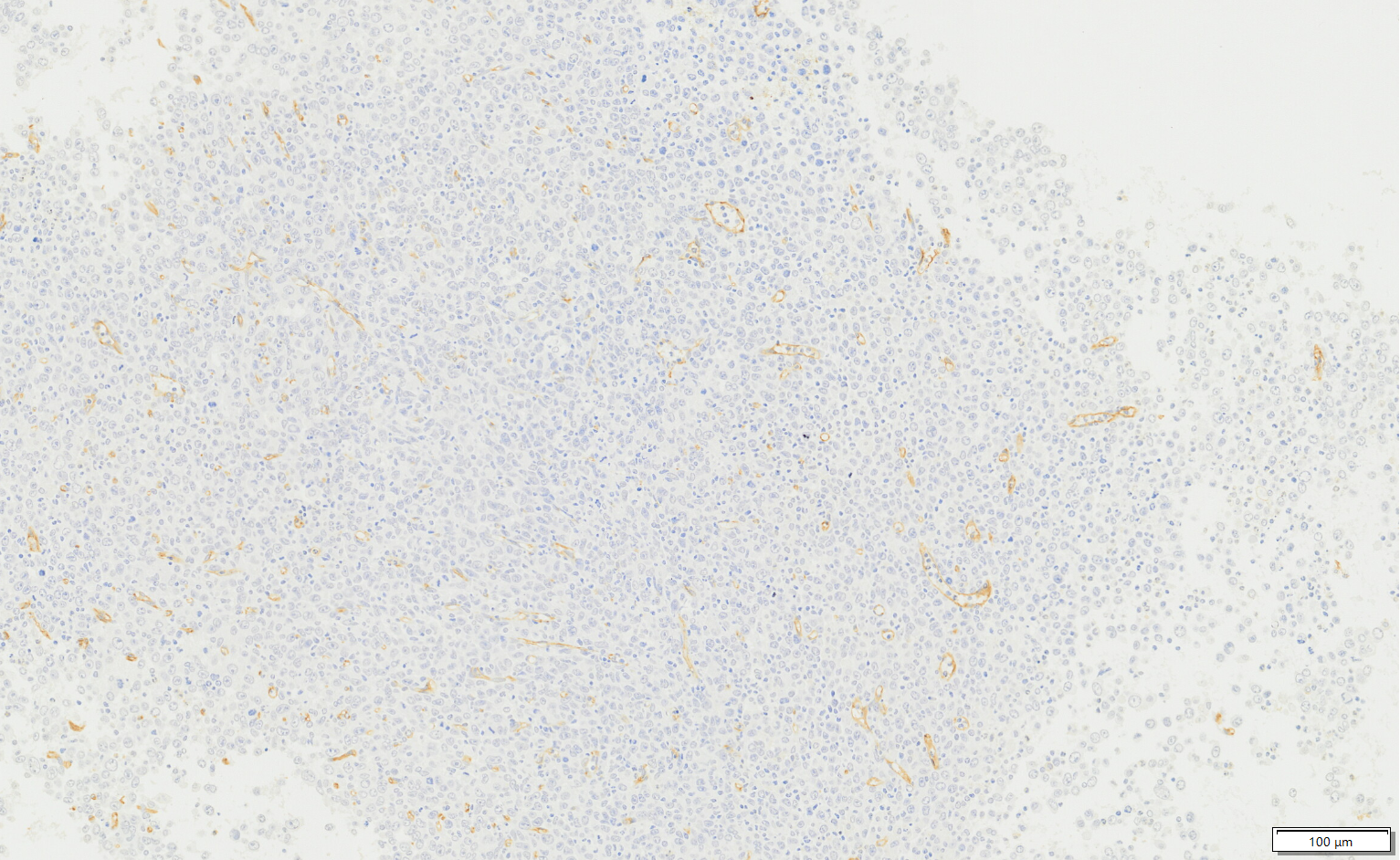

Supplement: Supplementary file 6 — Source data Fig. 4 [file 44319_2025_627_MOESM6_ESM.zip › Figure 4/4I/LLC A.f LY6G CD31.png]

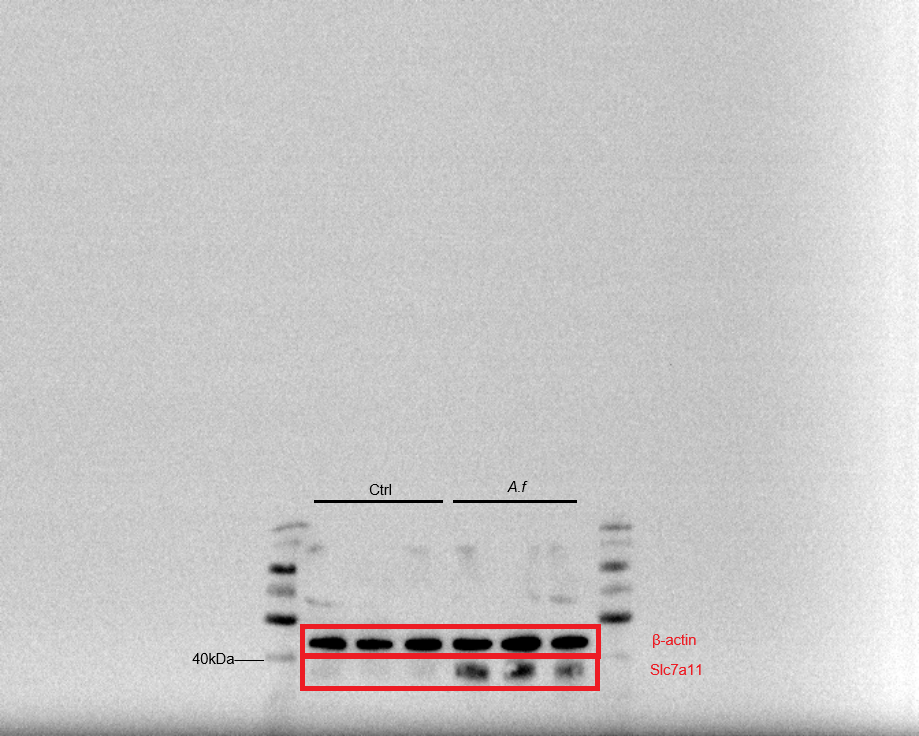

Supplement: Supplementary file 7 — Source data Fig. 5 [file 44319_2025_627_MOESM7_ESM.zip › Figure 5/5C/SLC7A11.tif]

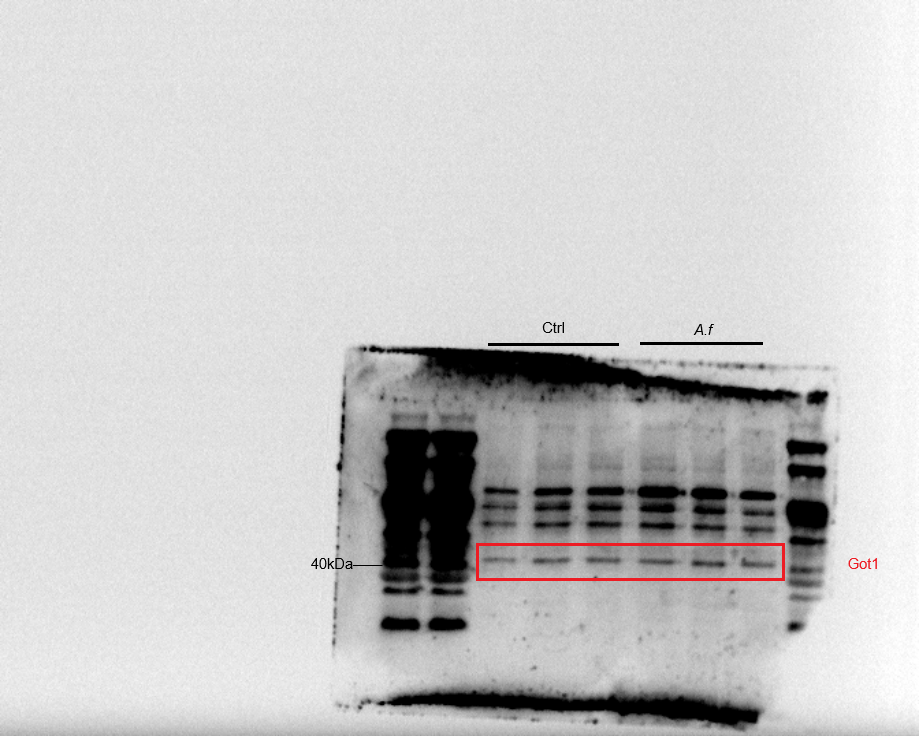

Supplement: Supplementary file 7 — Source data Fig. 5 [file 44319_2025_627_MOESM7_ESM.zip › Figure 5/5C/Got1.tif]

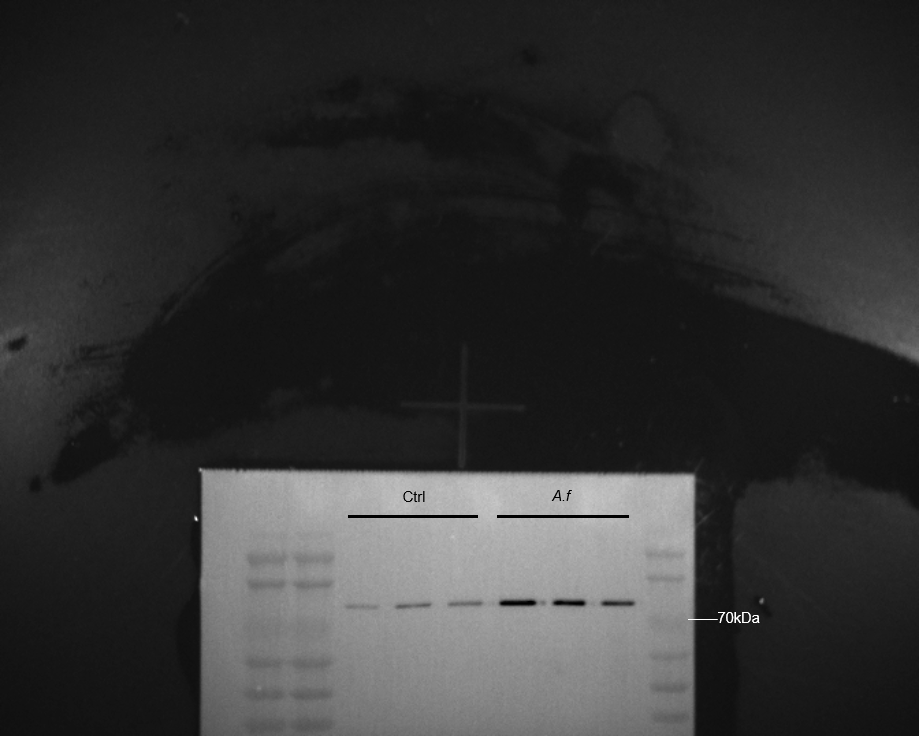

Supplement: Supplementary file 7 — Source data Fig. 5 [file 44319_2025_627_MOESM7_ESM.zip › Figure 5/5C/gclc.tif]

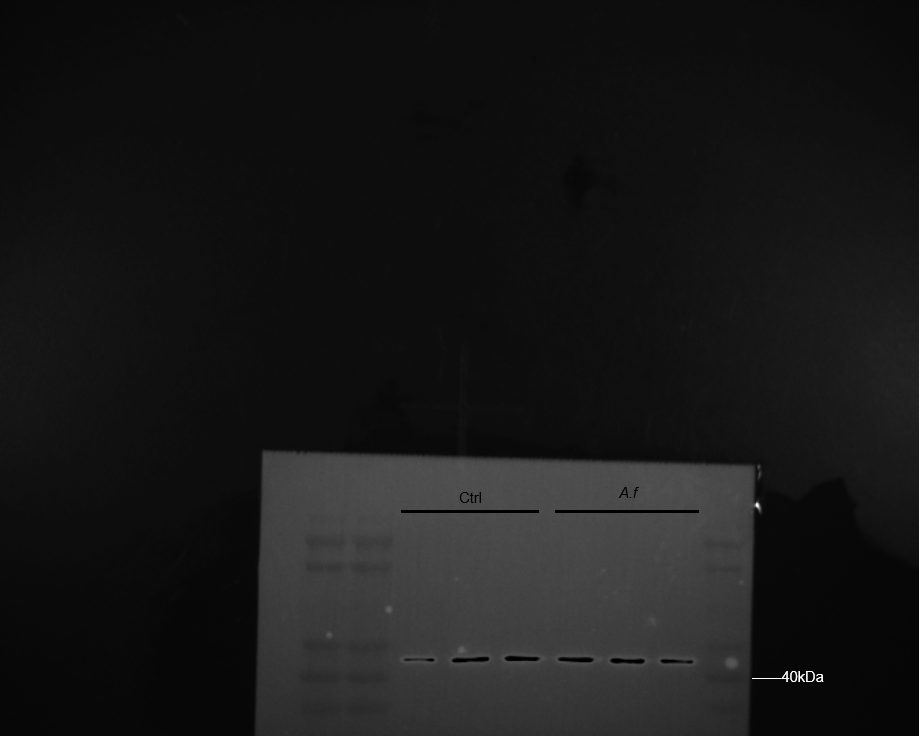

Supplement: Supplementary file 7 — Source data Fig. 5 [file 44319_2025_627_MOESM7_ESM.zip › Figure 5/5C/β-actin.tif]

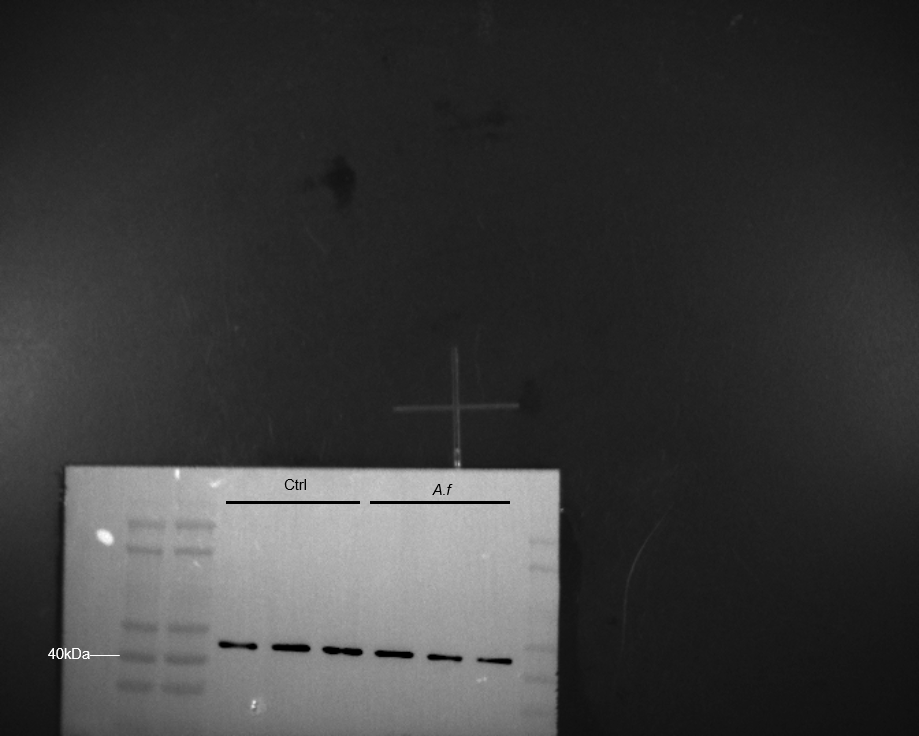

Supplement: Supplementary file 7 — Source data Fig. 5 [file 44319_2025_627_MOESM7_ESM.zip › Figure 5/5C/β-actin-2.png]

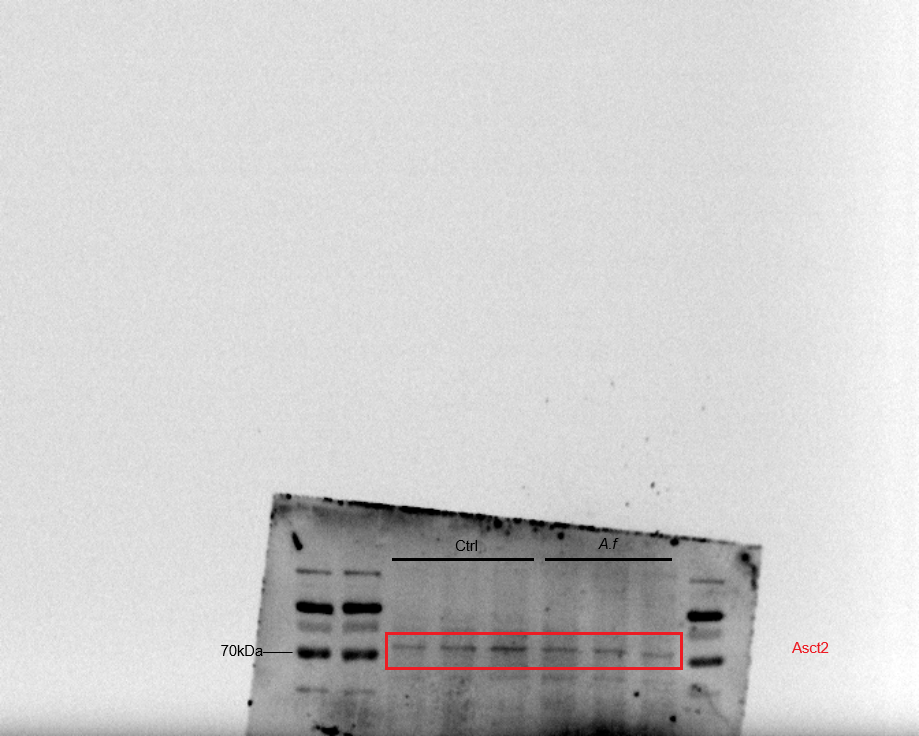

Supplement: Supplementary file 7 — Source data Fig. 5 [file 44319_2025_627_MOESM7_ESM.zip › Figure 5/5C/Asct2.tif]

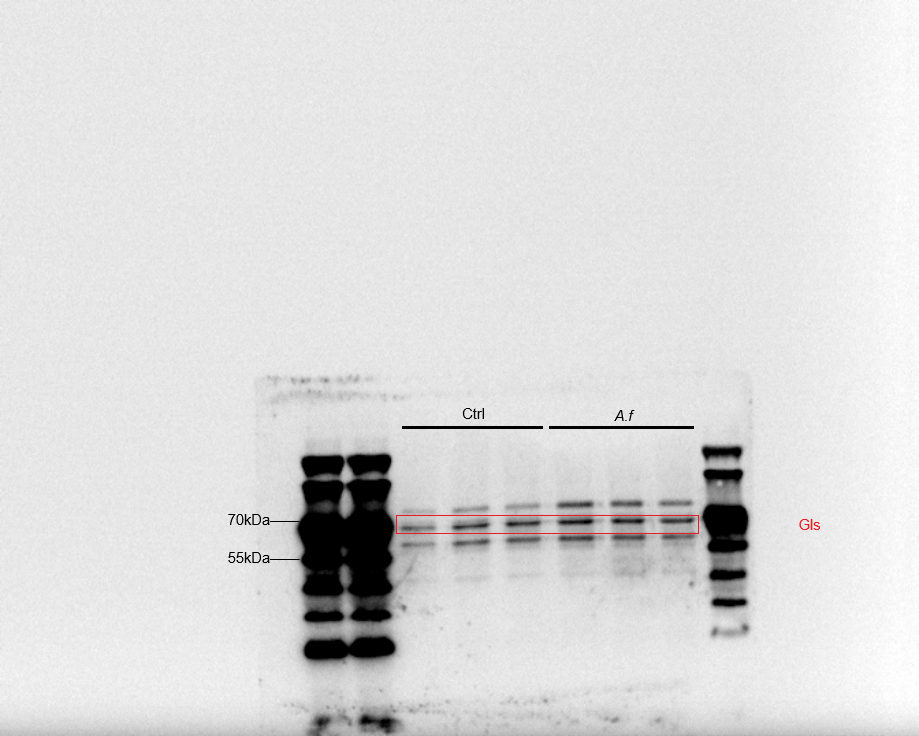

Supplement: Supplementary file 7 — Source data Fig. 5 [file 44319_2025_627_MOESM7_ESM.zip › Figure 5/5C/Gls.tif]

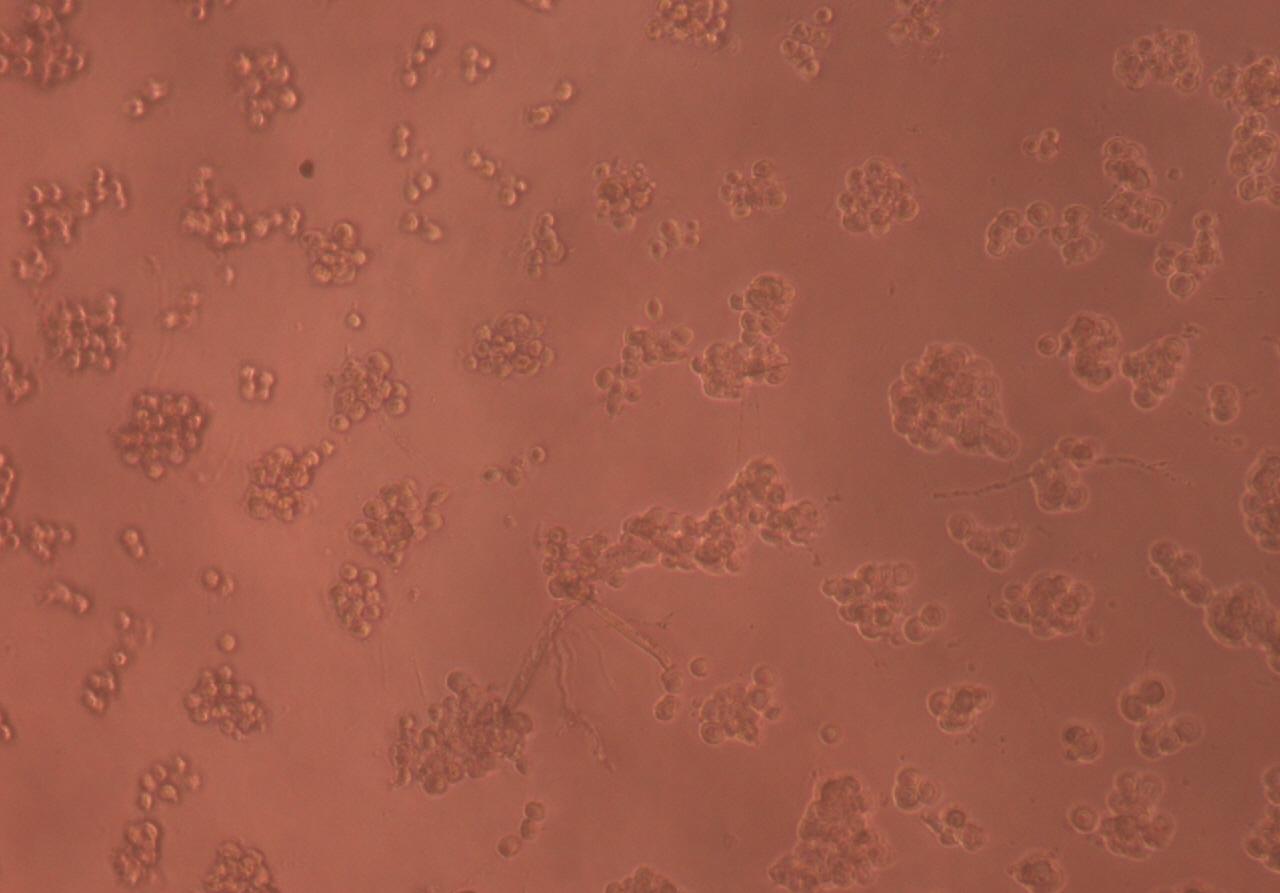

Supplement: Supplementary file 7 — Source data Fig. 5 [file 44319_2025_627_MOESM7_ESM.zip › Figure 5/5D/A.f+SASP.jpg]

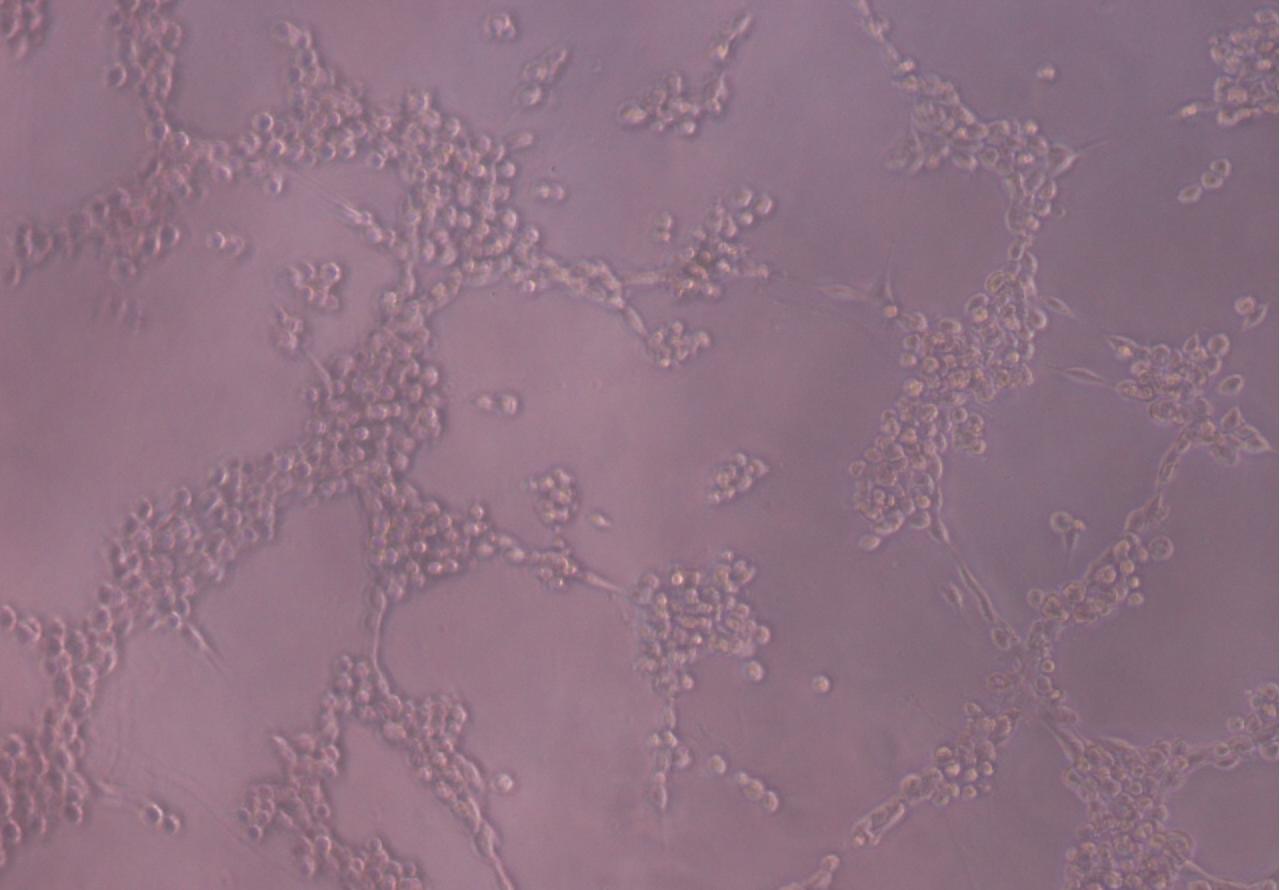

Supplement: Supplementary file 7 — Source data Fig. 5 [file 44319_2025_627_MOESM7_ESM.zip › Figure 5/5D/A.f.jpg]

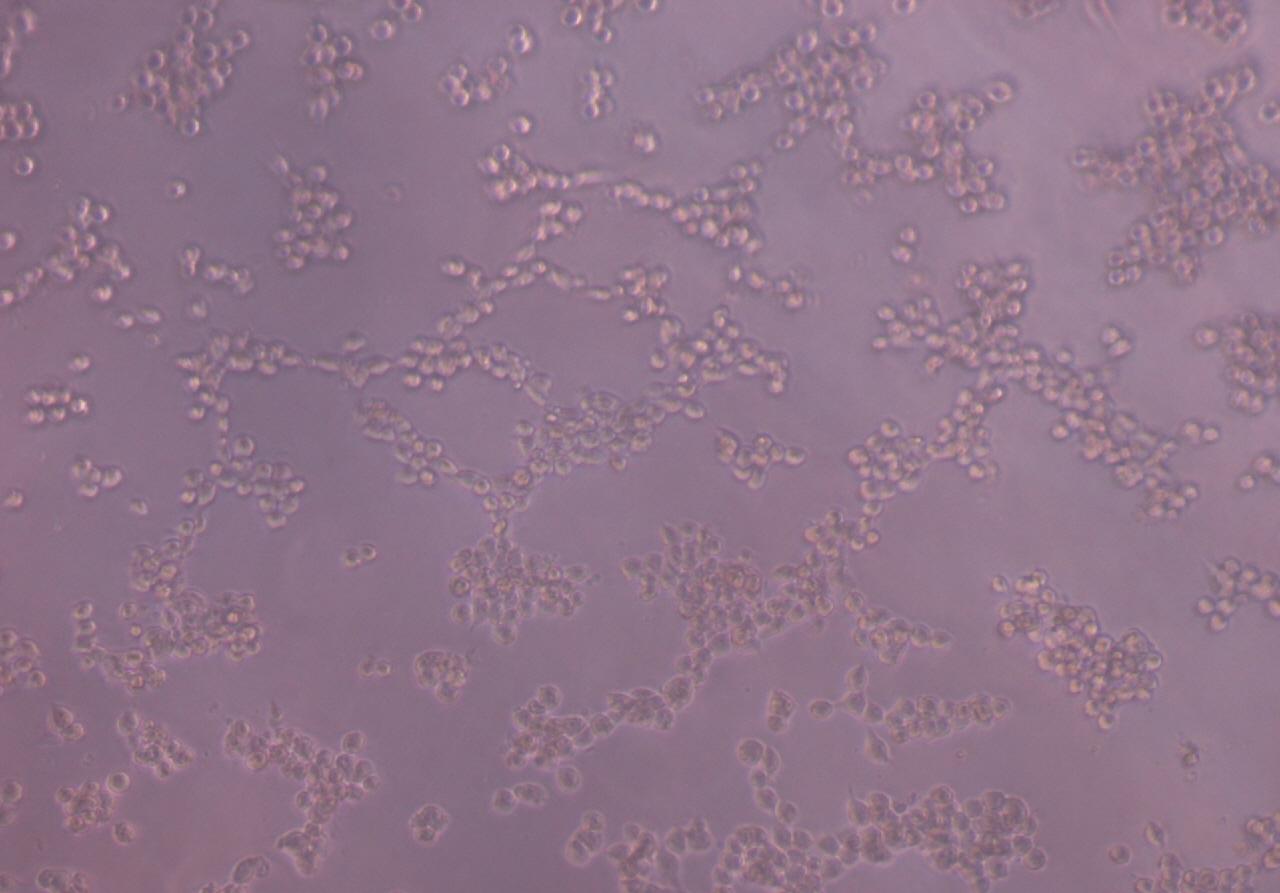

Supplement: Supplementary file 7 — Source data Fig. 5 [file 44319_2025_627_MOESM7_ESM.zip › Figure 5/5D/Ctrl.jpg]

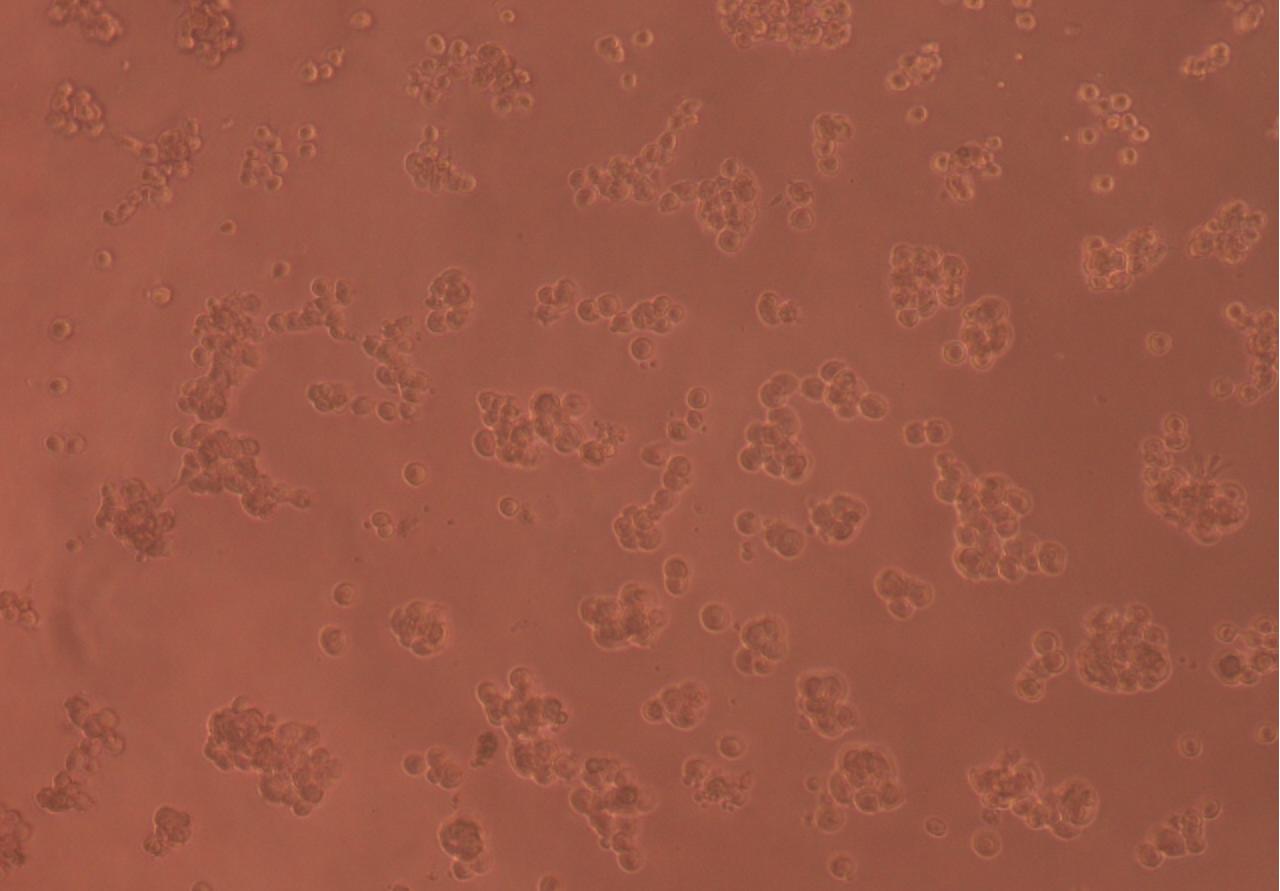

Supplement: Supplementary file 7 — Source data Fig. 5 [file 44319_2025_627_MOESM7_ESM.zip › Figure 5/5D/SASP.jpg]

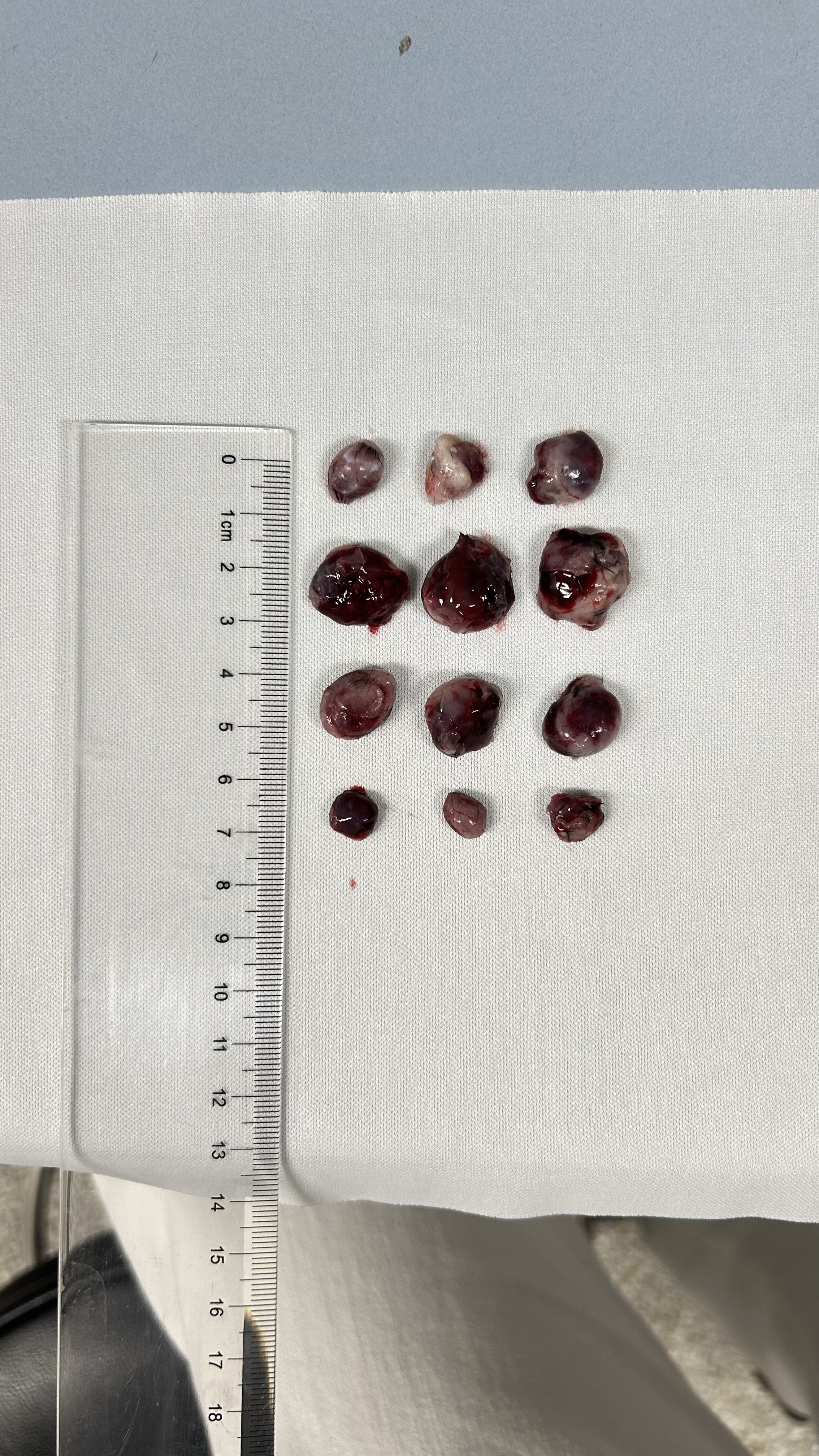

Supplement: Supplementary file 7 — Source data Fig. 5 [file 44319_2025_627_MOESM7_ESM.zip › Figure 5/5H/Tumor.jpg]

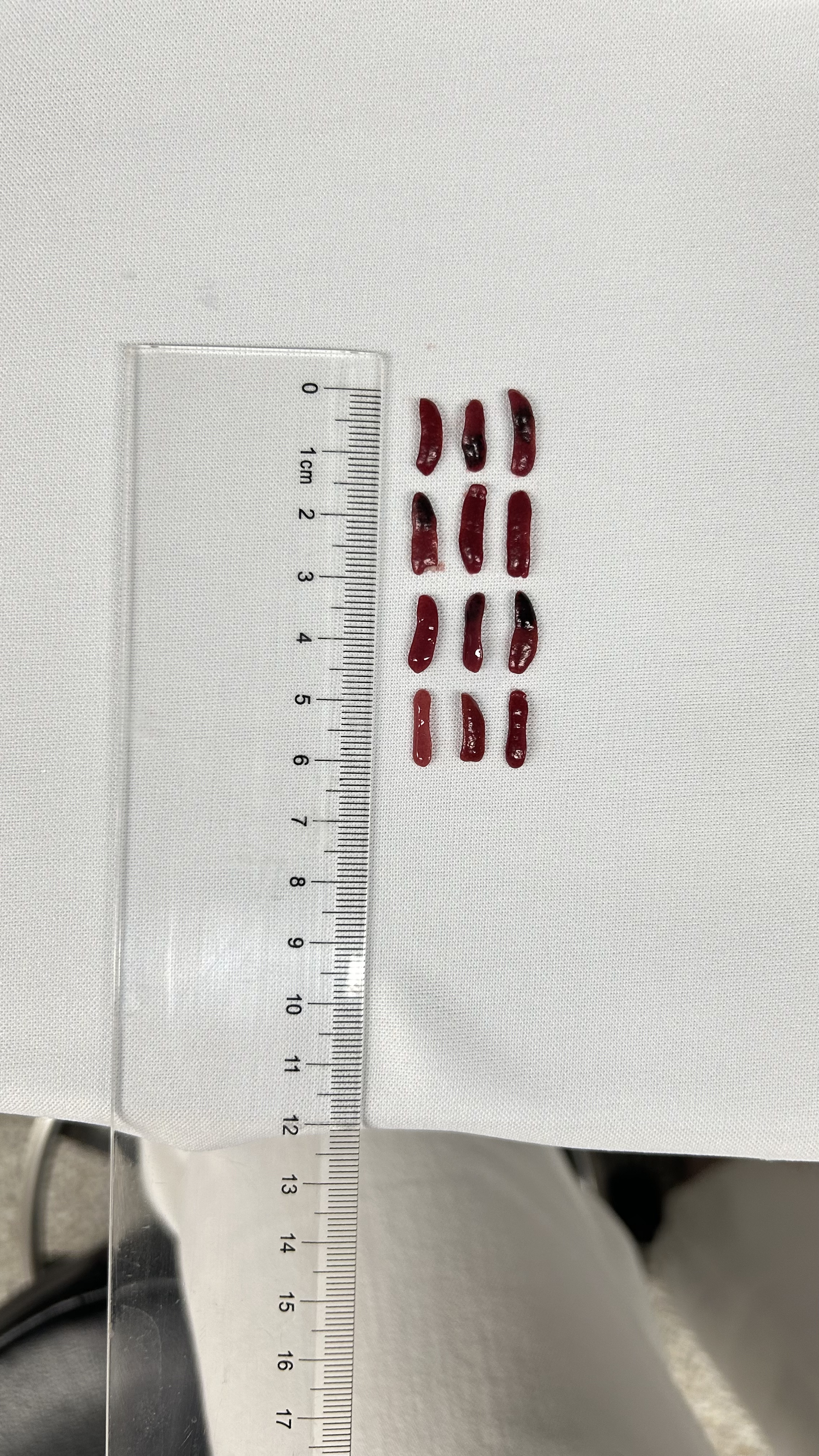

Supplement: Supplementary file 7 — Source data Fig. 5 [file 44319_2025_627_MOESM7_ESM.zip › Figure 5/5J/Spleen.jpg]

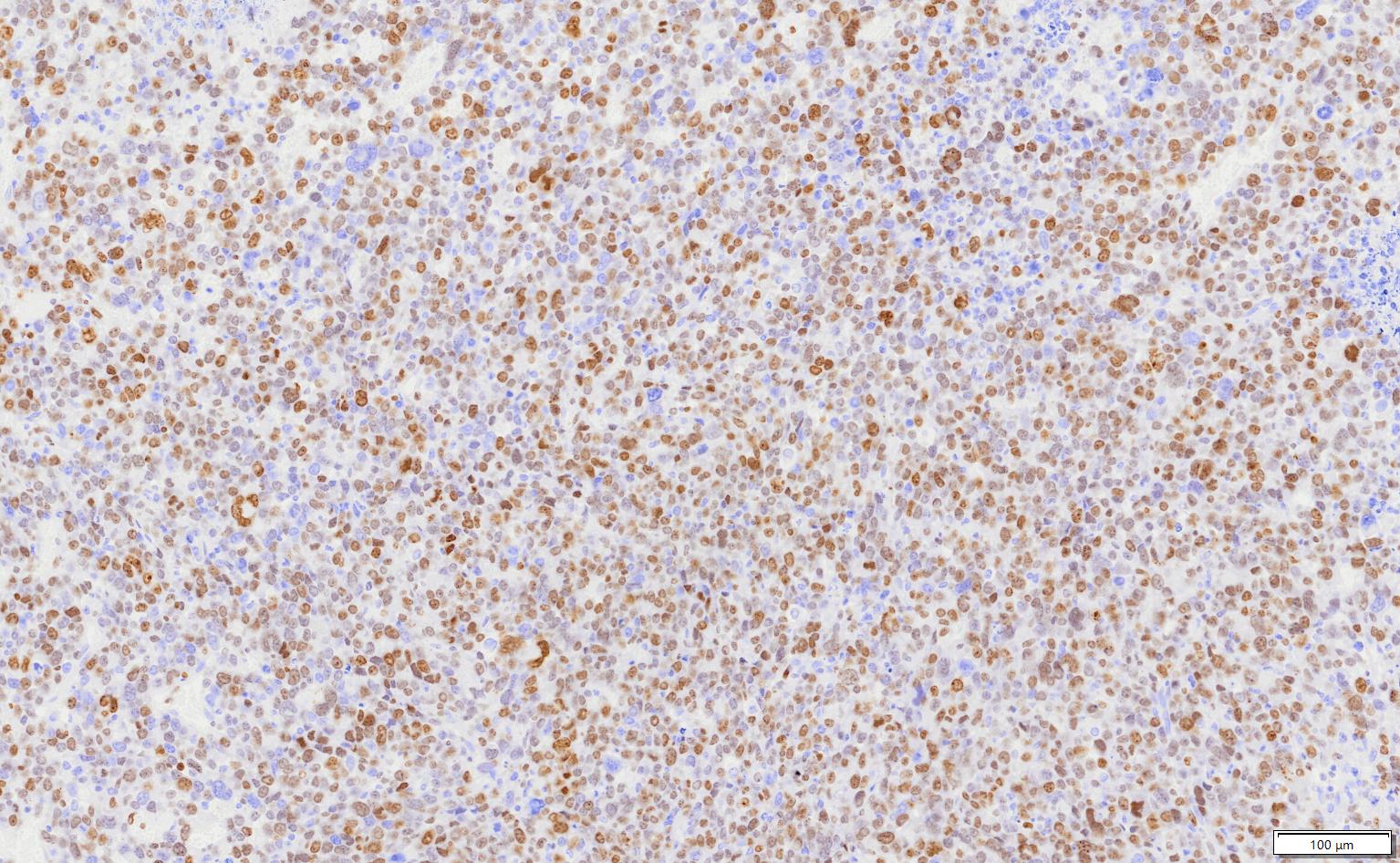

Supplement: Supplementary file 7 — Source data Fig. 5 [file 44319_2025_627_MOESM7_ESM.zip › Figure 5/5L/LLC A.f Ki67.png]

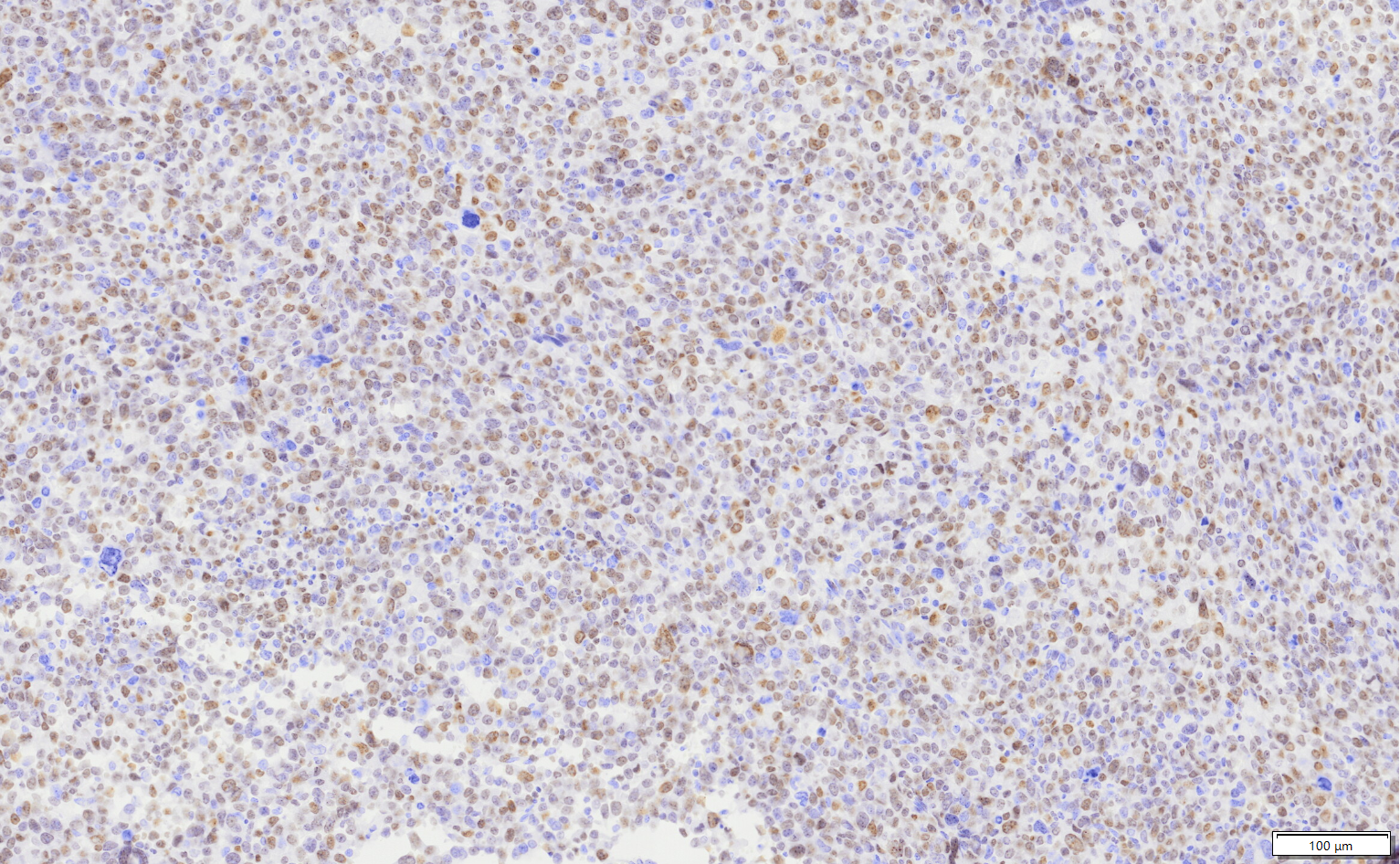

Supplement: Supplementary file 7 — Source data Fig. 5 [file 44319_2025_627_MOESM7_ESM.zip › Figure 5/5L/LLC A.f SASP Ki67.png]

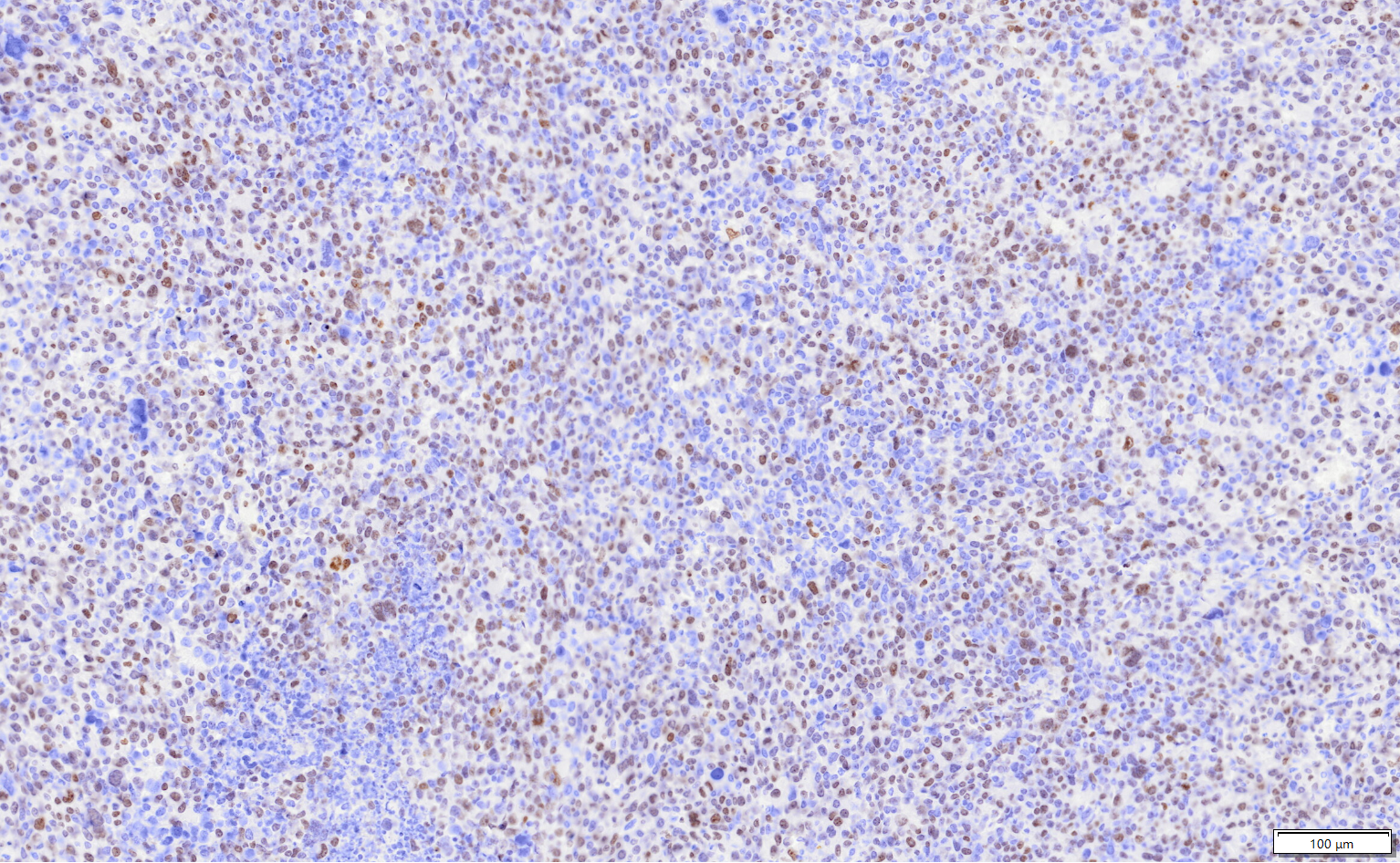

Supplement: Supplementary file 7 — Source data Fig. 5 [file 44319_2025_627_MOESM7_ESM.zip › Figure 5/5L/LLC Ctrl Ki67.png]

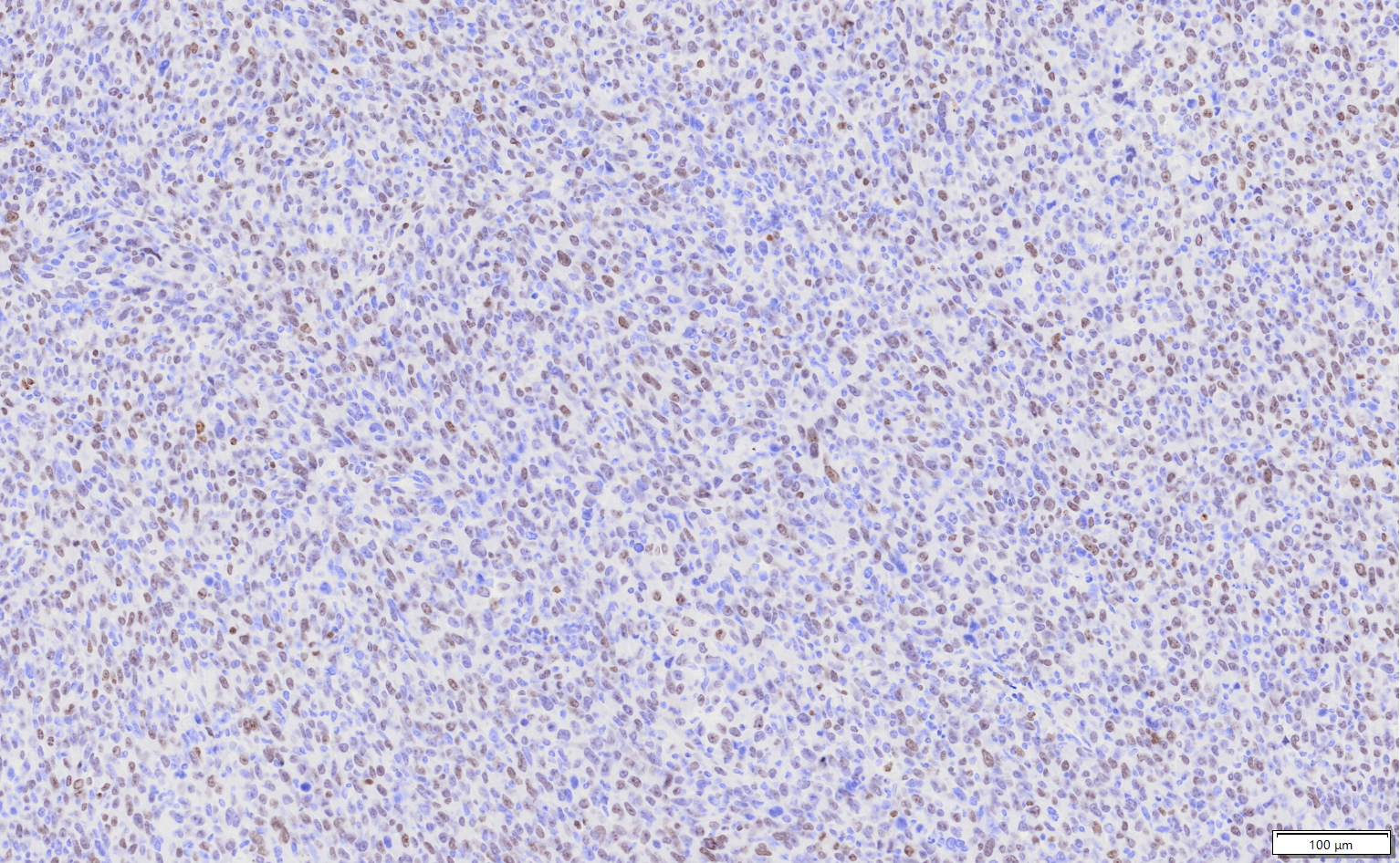

Supplement: Supplementary file 7 — Source data Fig. 5 [file 44319_2025_627_MOESM7_ESM.zip › Figure 5/5L/LLC SASP Ki67.png]

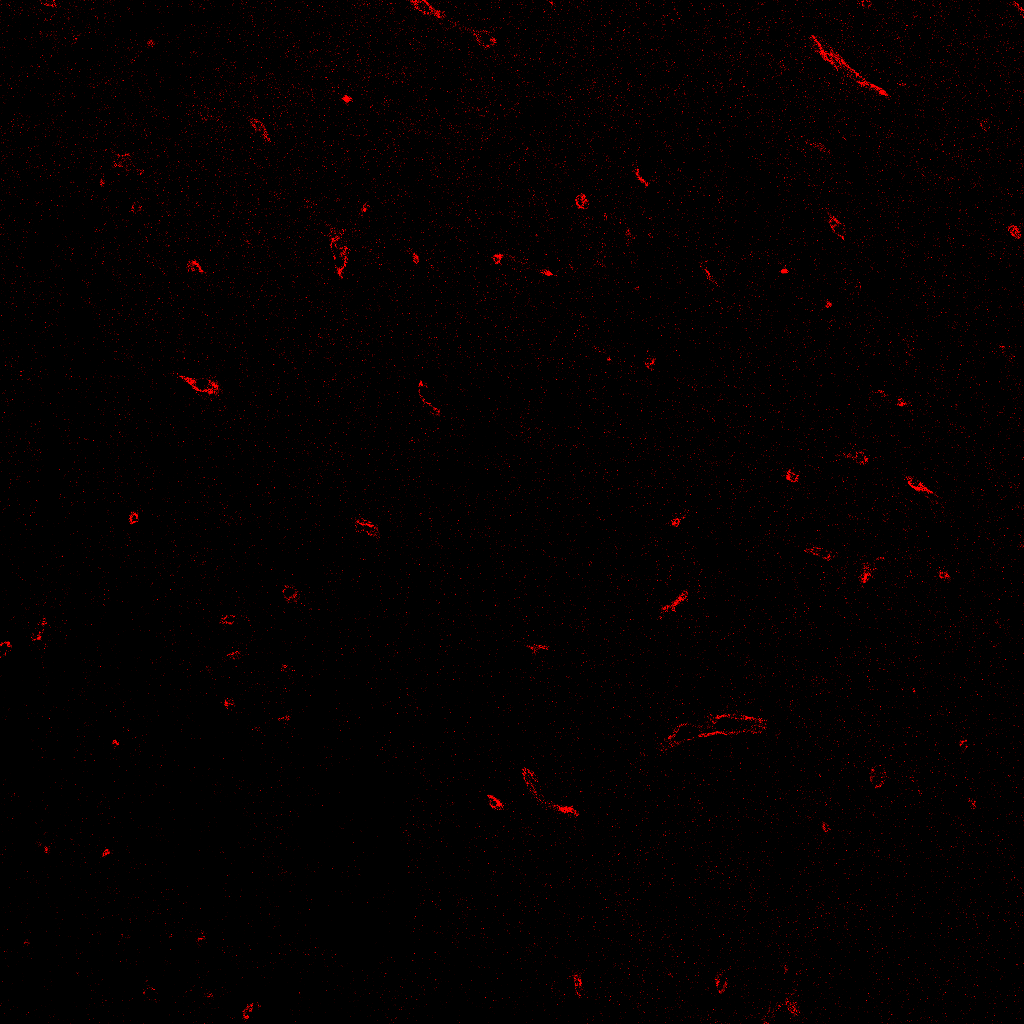

Supplement: Supplementary file 7 — Source data Fig. 5 [file 44319_2025_627_MOESM7_ESM.zip › Figure 5/5O/LLC Ctrl CD34.tif]
