# Supplementary figures and images for: Aspergillus fumigatus promotes tumor angiogenesis via SLC7A11 on myeloid-derived suppressor cells (part 2 of 2)
Source: EMBO Rep. 2025 Nov 17;26(24):6266–91. doi: 10.1038/s44319-025-00627-x (PMC12715260; doi:10.1038/s44319-025-00627-x)

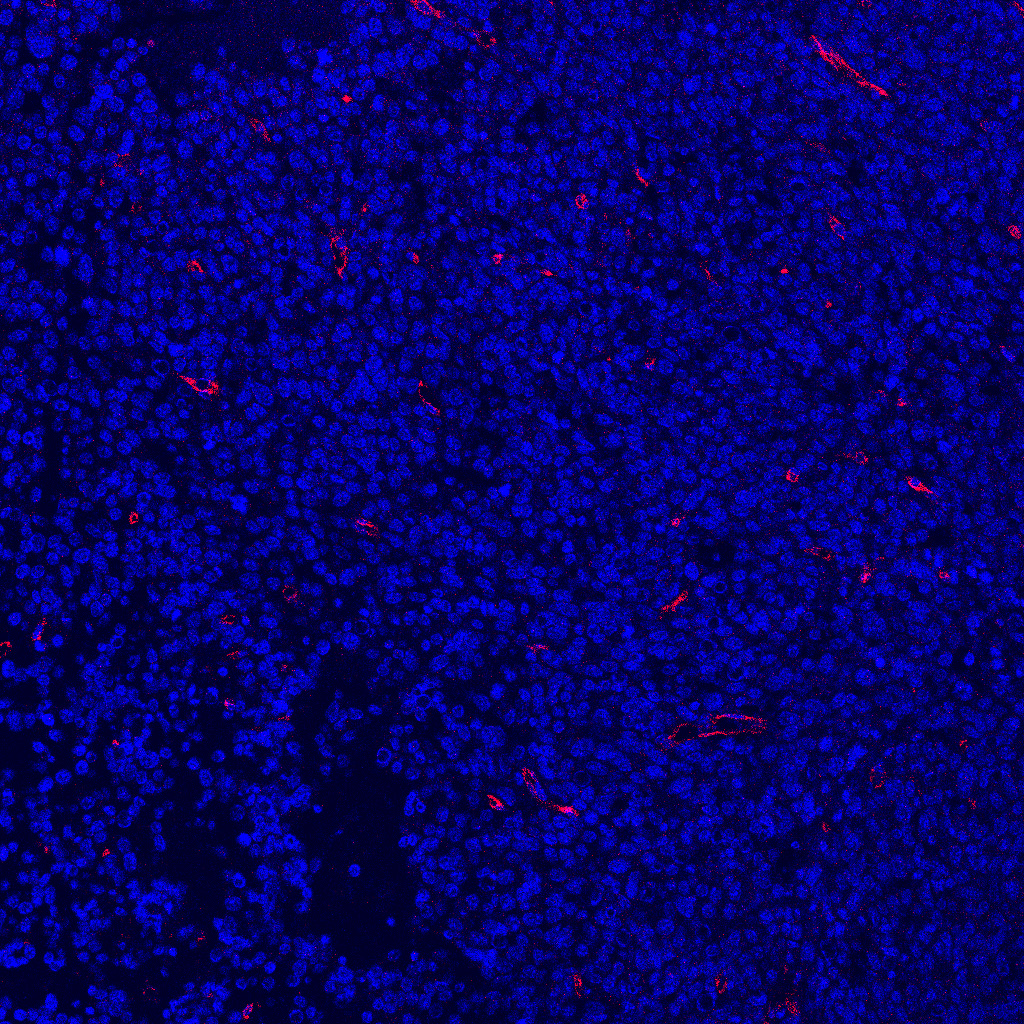

Supplement: Supplementary file 7 — Source data Fig. 5 [file 44319_2025_627_MOESM7_ESM.zip › Figure 5/5O/LLC Ctrl Merged.tif]

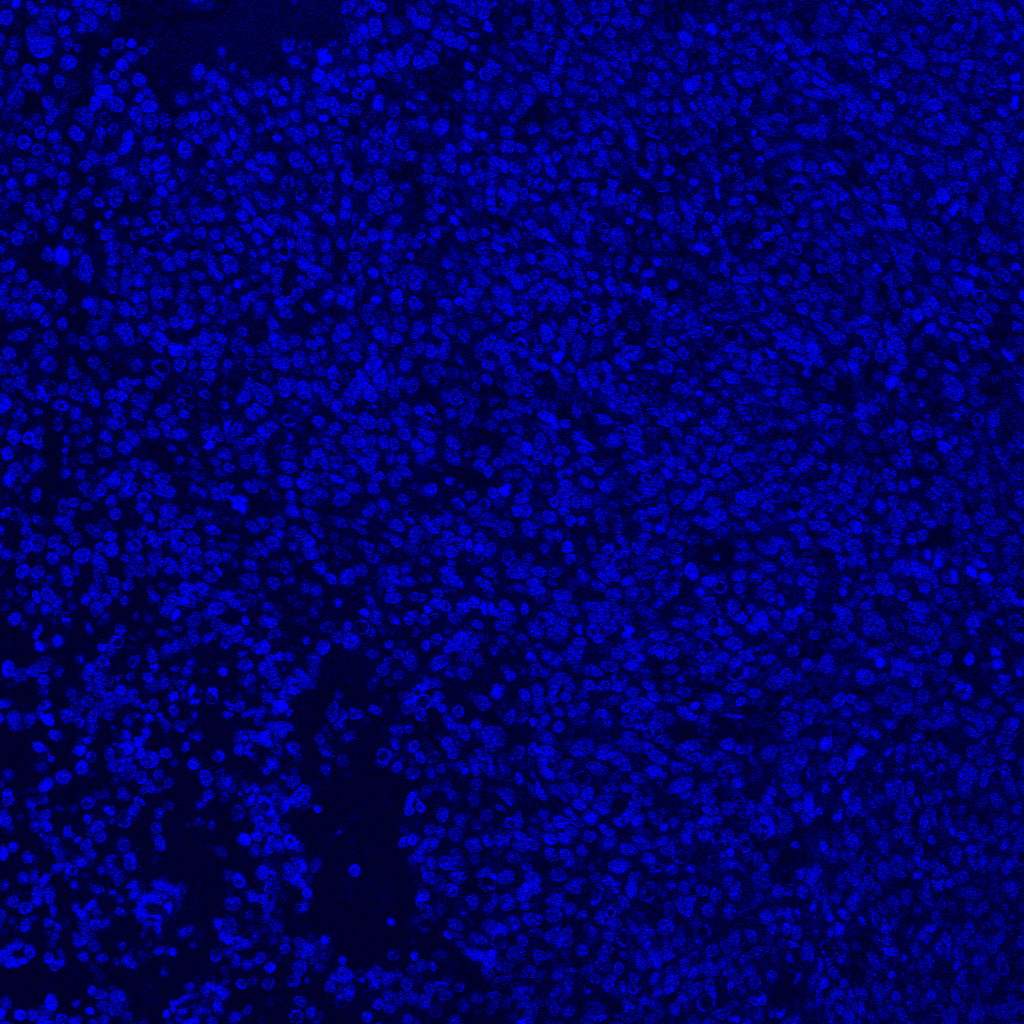

Supplement: Supplementary file 7 — Source data Fig. 5 [file 44319_2025_627_MOESM7_ESM.zip › Figure 5/5O/LLC Ctrl DAPI.tif]

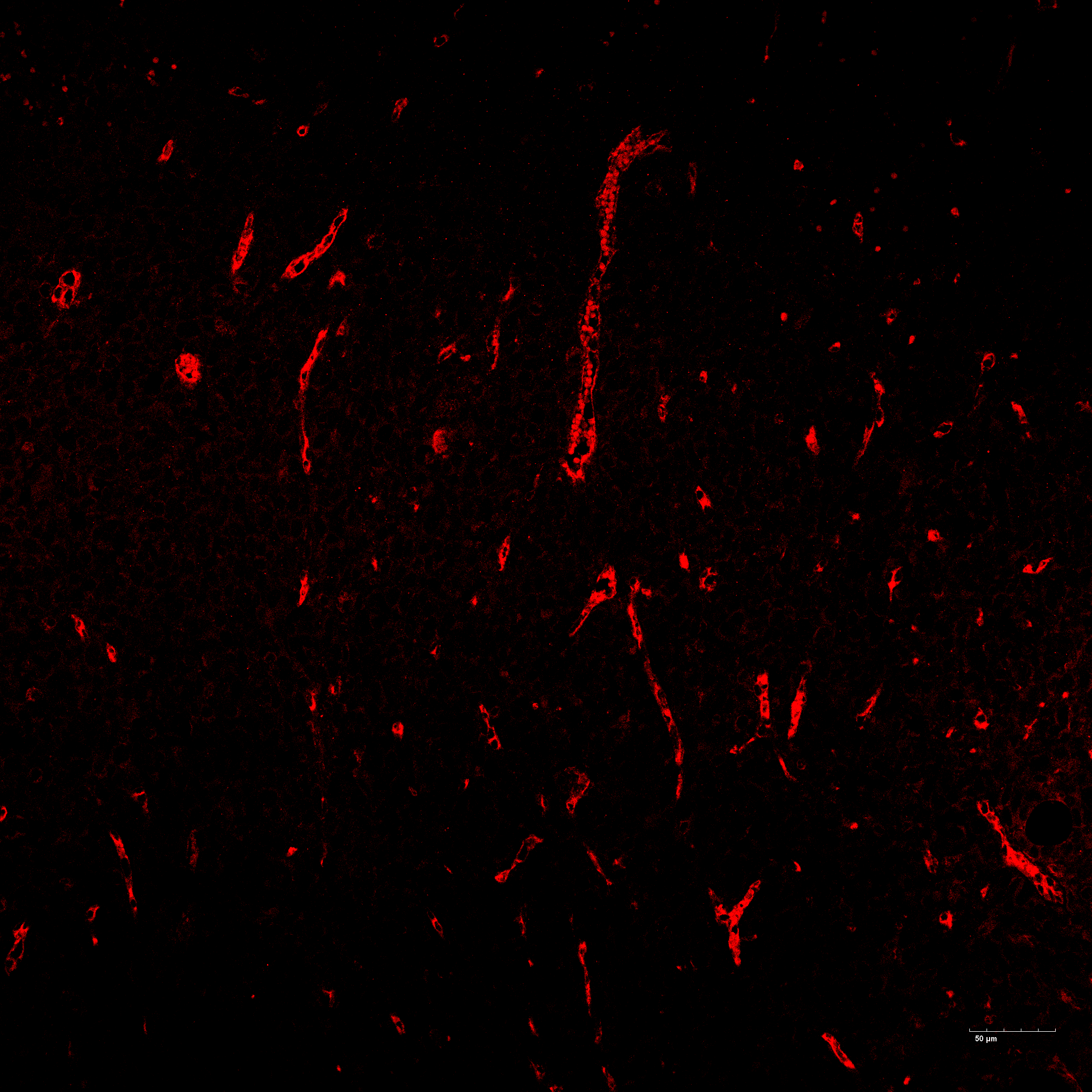

Supplement: Supplementary file 7 — Source data Fig. 5 [file 44319_2025_627_MOESM7_ESM.zip › Figure 5/5O/LLC Af CD34.png]

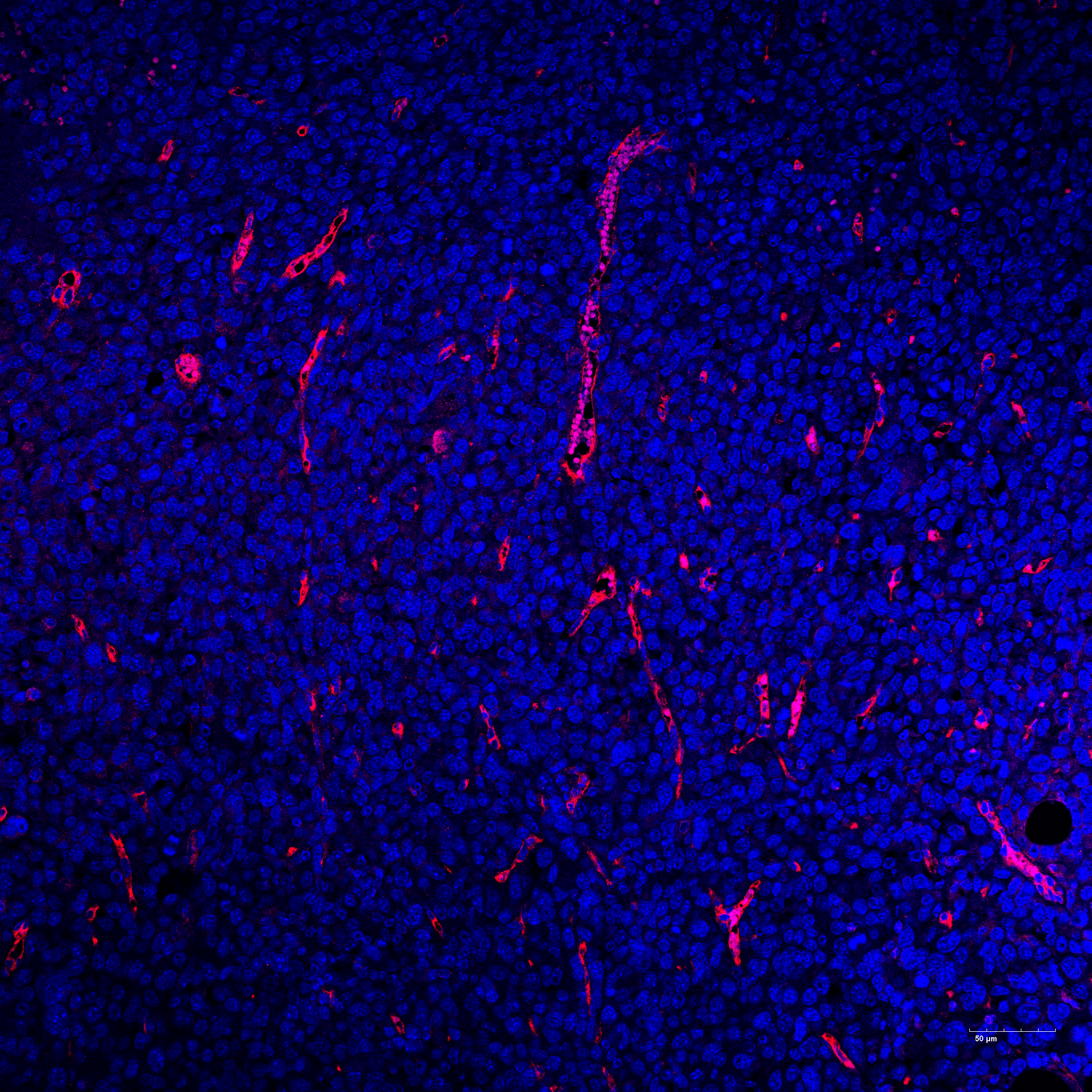

Supplement: Supplementary file 7 — Source data Fig. 5 [file 44319_2025_627_MOESM7_ESM.zip › Figure 5/5O/LLC AF Merged.png]

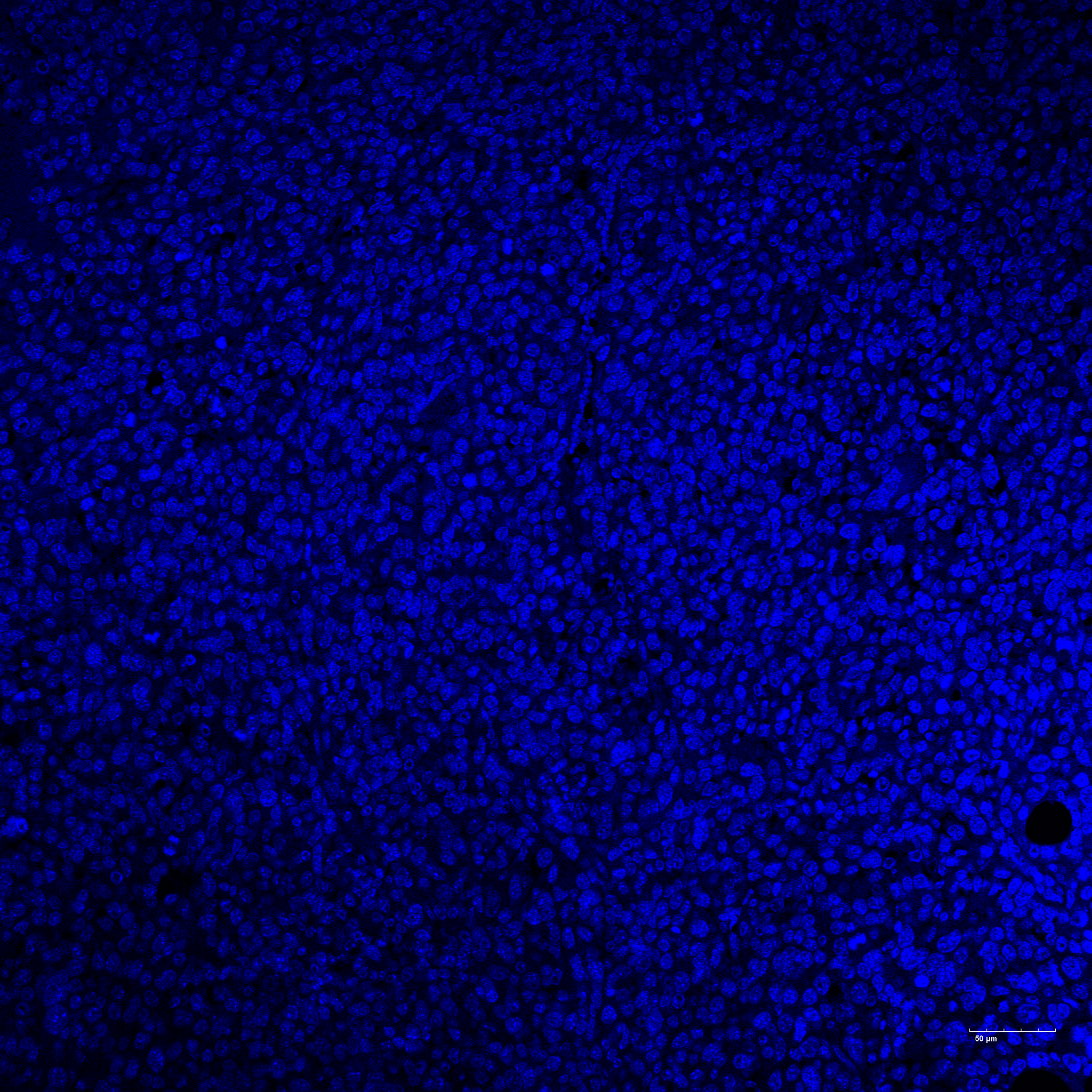

Supplement: Supplementary file 7 — Source data Fig. 5 [file 44319_2025_627_MOESM7_ESM.zip › Figure 5/5O/LLC Af DAPI.png]

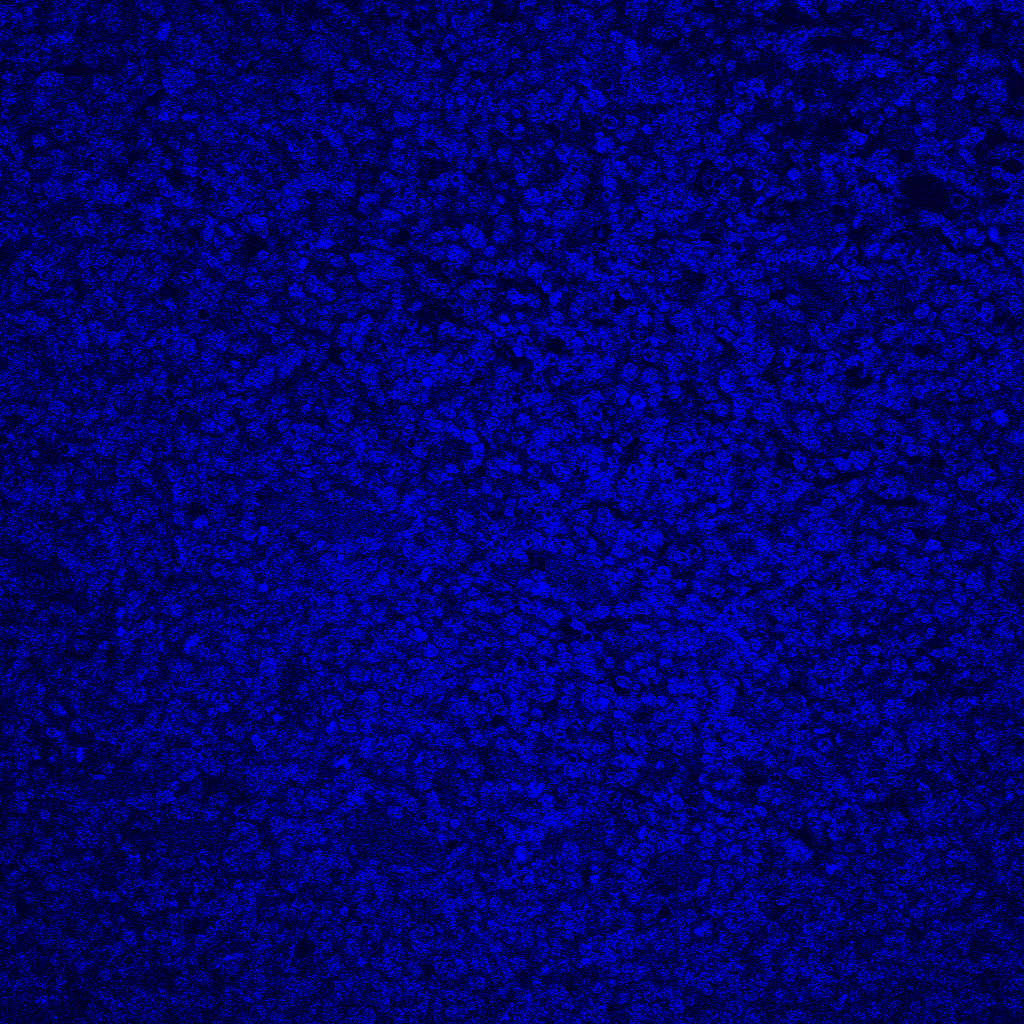

Supplement: Supplementary file 7 — Source data Fig. 5 [file 44319_2025_627_MOESM7_ESM.zip › Figure 5/5O/LLC Af SASP DAPI.tif]

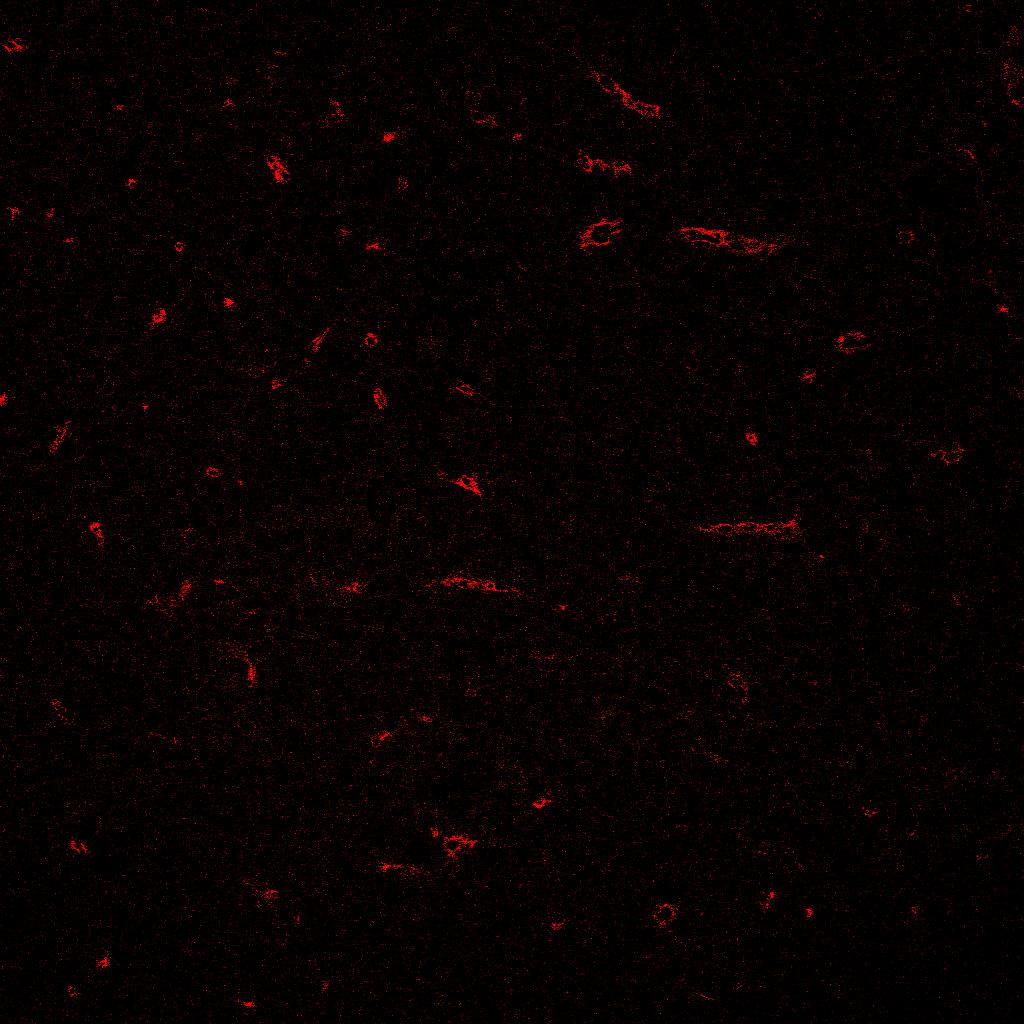

Supplement: Supplementary file 7 — Source data Fig. 5 [file 44319_2025_627_MOESM7_ESM.zip › Figure 5/5O/LLC Af SASP CD34.tif]

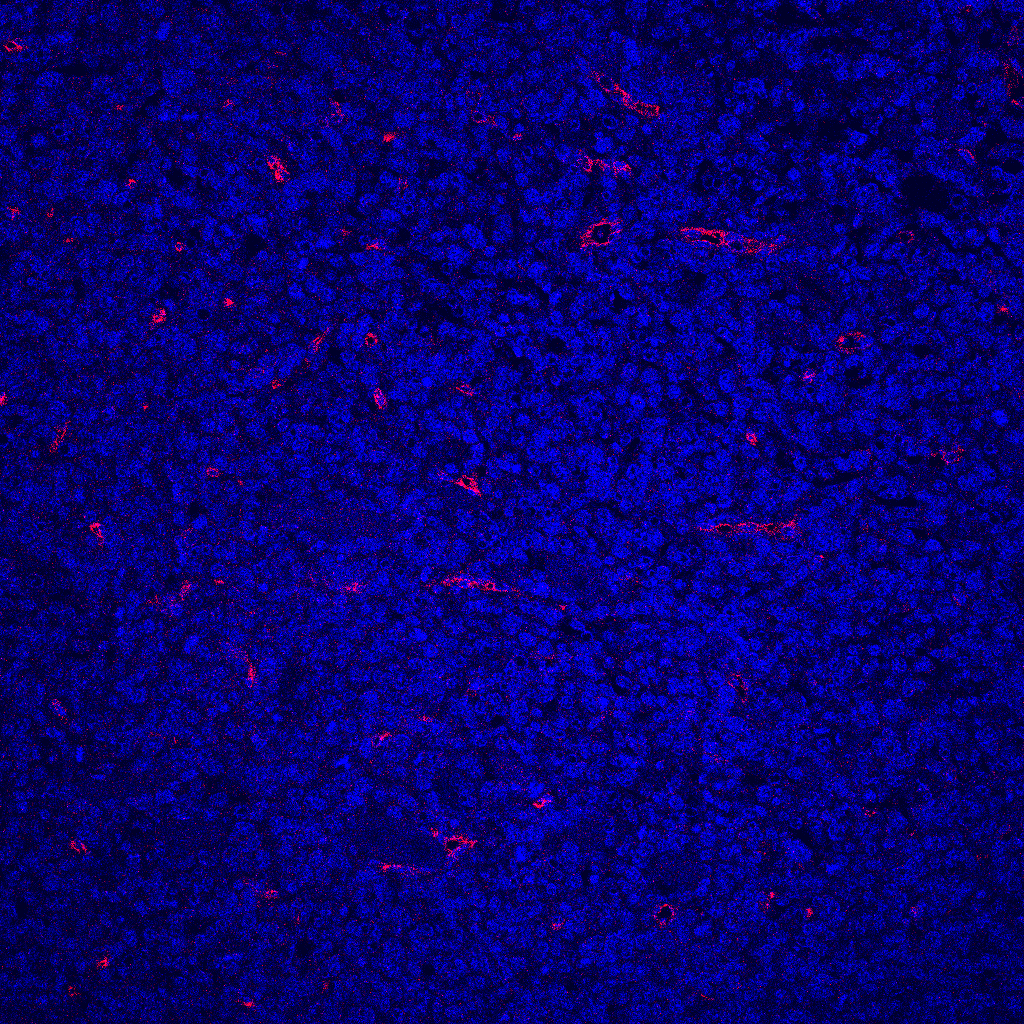

Supplement: Supplementary file 7 — Source data Fig. 5 [file 44319_2025_627_MOESM7_ESM.zip › Figure 5/5O/LLC Af SASP Merged.tif]

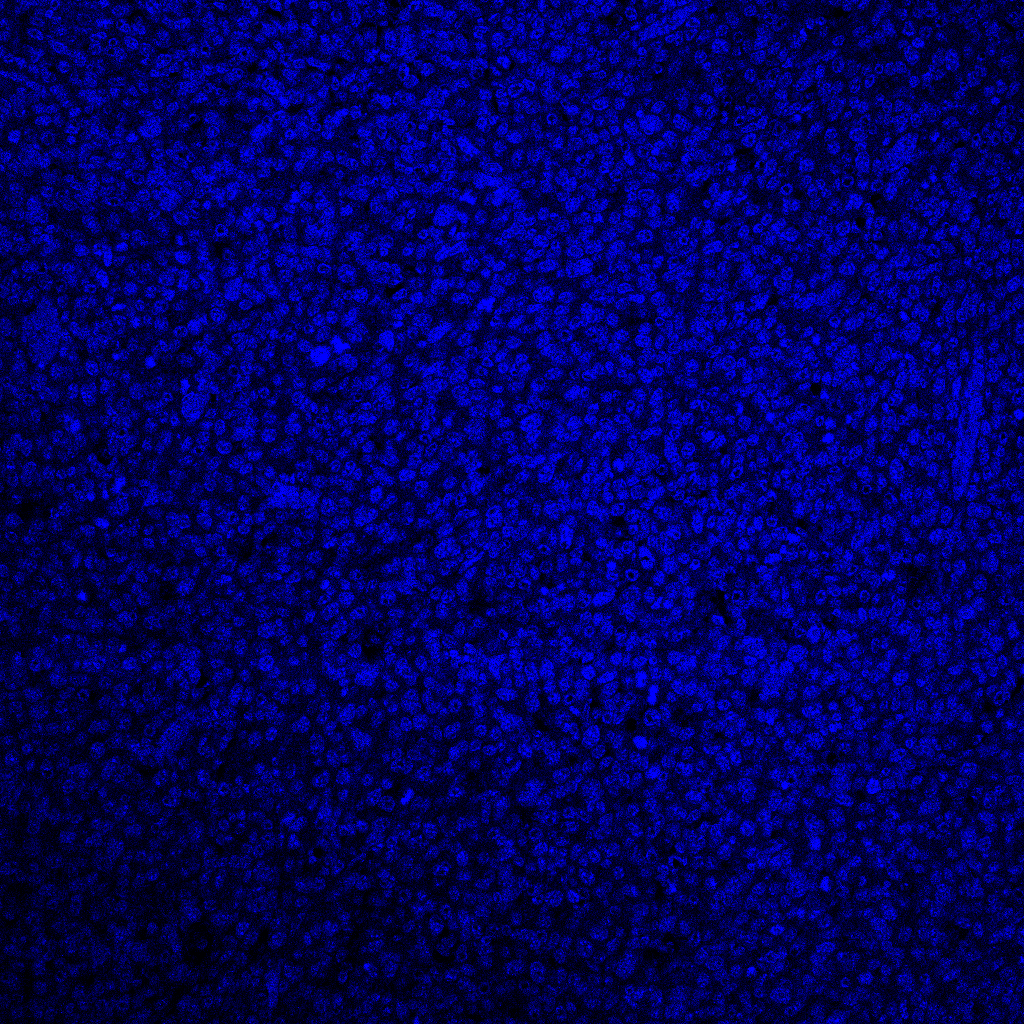

Supplement: Supplementary file 7 — Source data Fig. 5 [file 44319_2025_627_MOESM7_ESM.zip › Figure 5/5O/LLC SASP DAPI.tif]

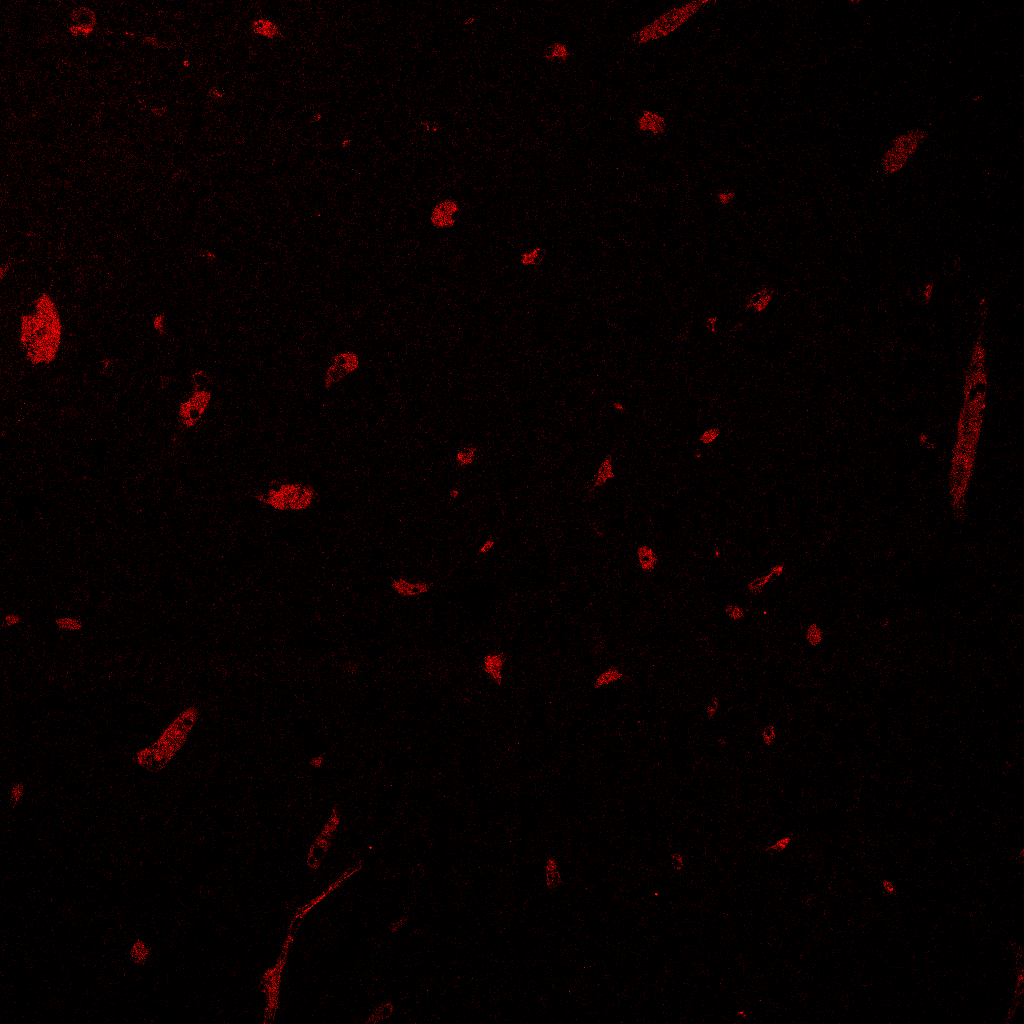

Supplement: Supplementary file 7 — Source data Fig. 5 [file 44319_2025_627_MOESM7_ESM.zip › Figure 5/5O/LLC SASP CD34.tif]

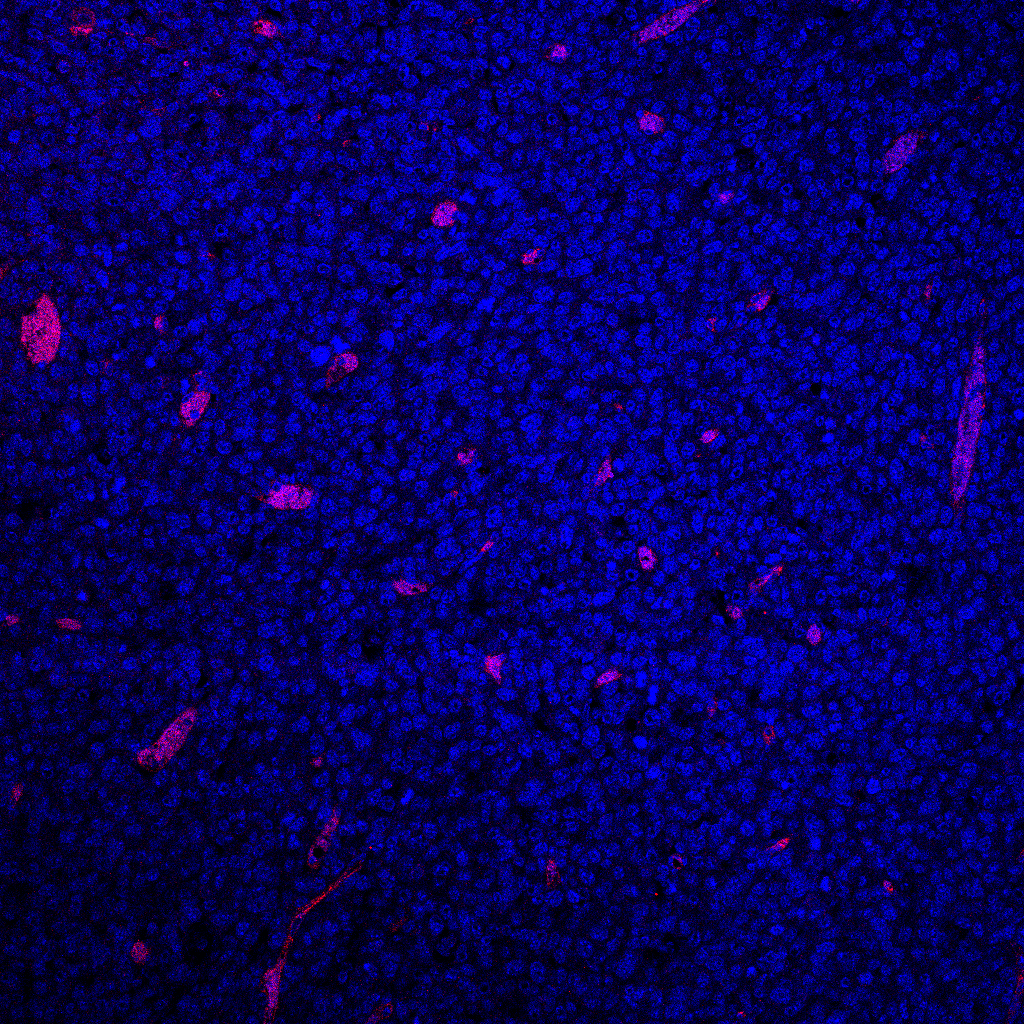

Supplement: Supplementary file 7 — Source data Fig. 5 [file 44319_2025_627_MOESM7_ESM.zip › Figure 5/5O/LLC SASP Merged.tif]

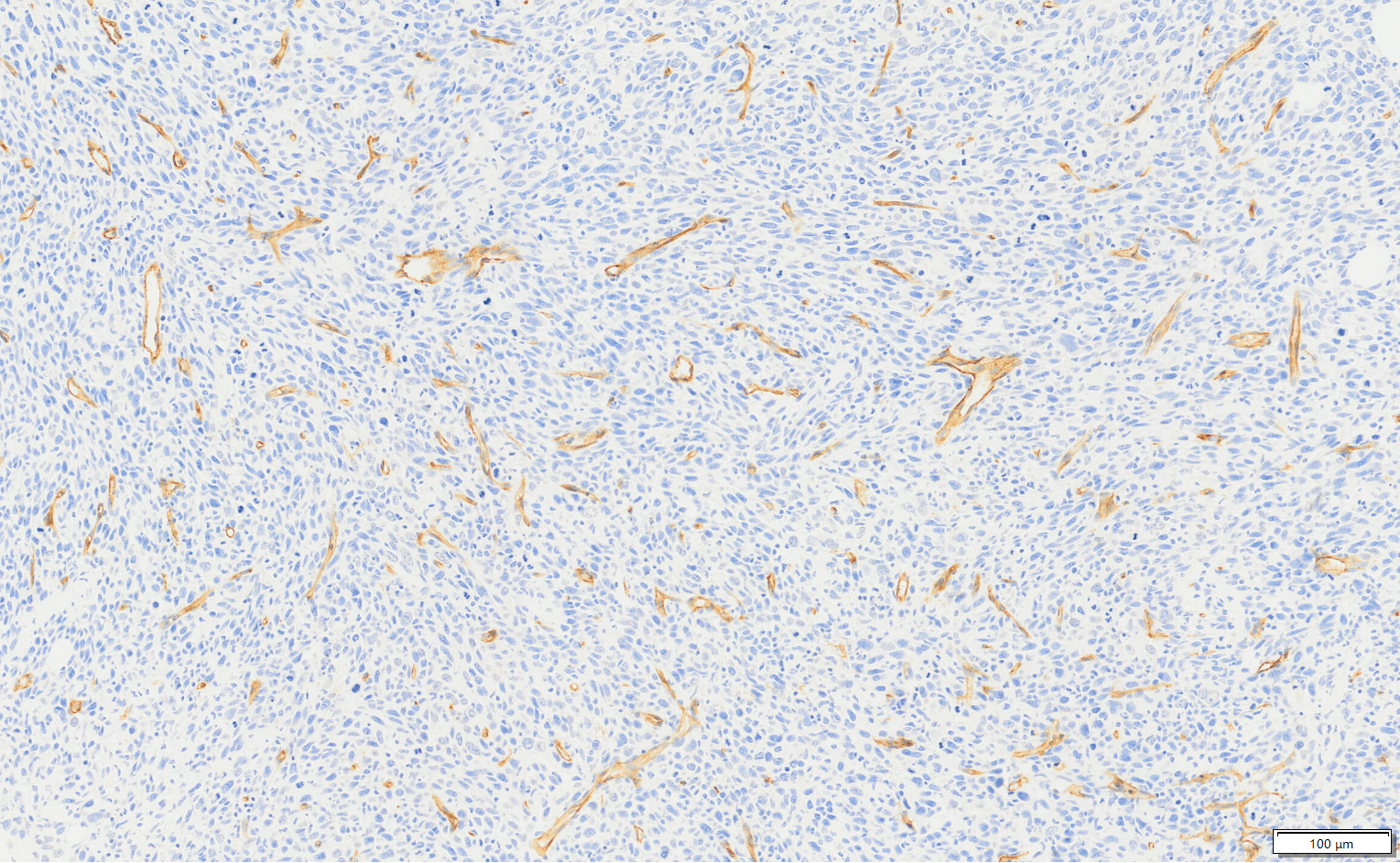

Supplement: Supplementary file 7 — Source data Fig. 5 [file 44319_2025_627_MOESM7_ESM.zip › Figure 5/5N/LLC A.f CD31.png]

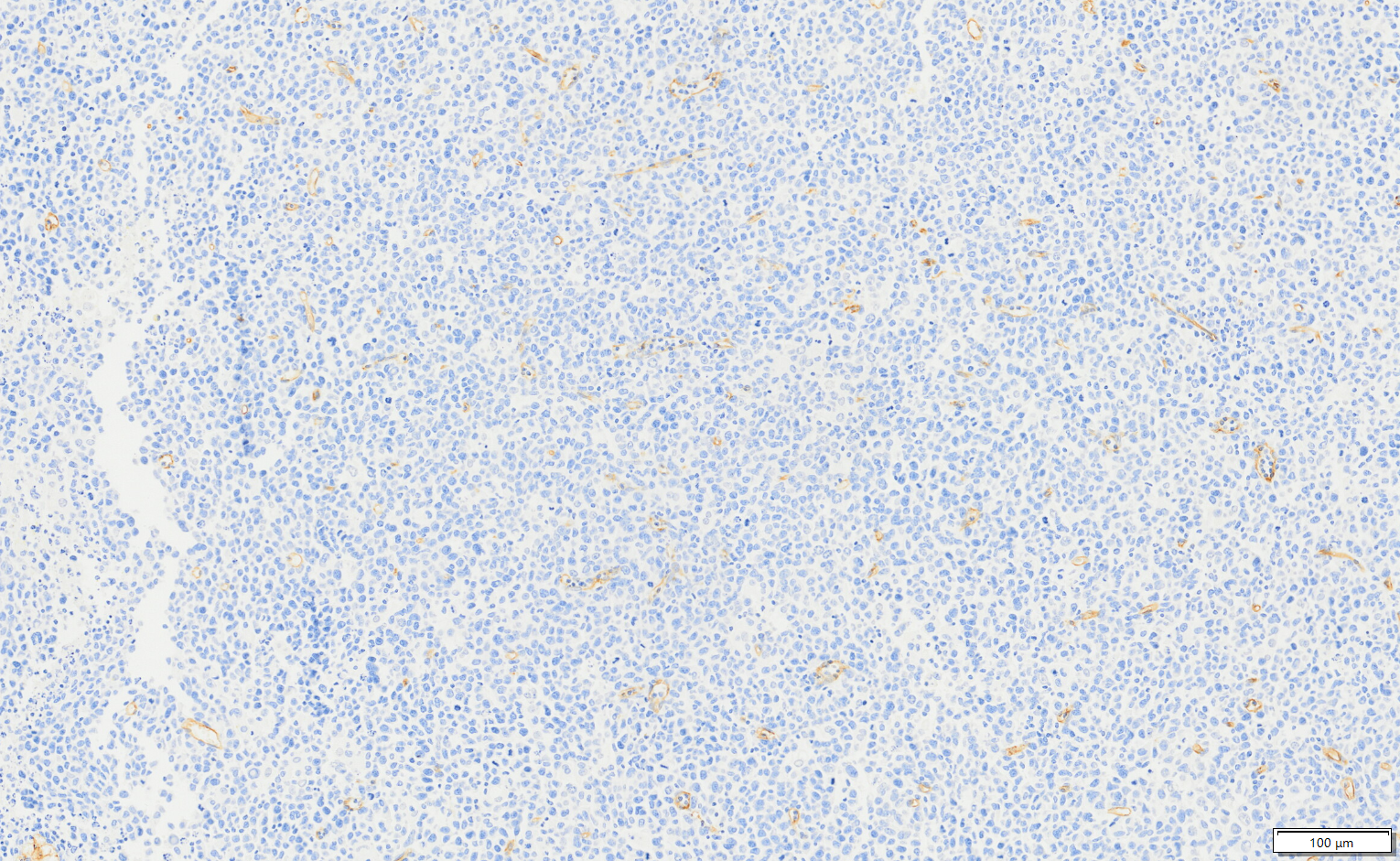

Supplement: Supplementary file 7 — Source data Fig. 5 [file 44319_2025_627_MOESM7_ESM.zip › Figure 5/5N/LLC A.f SASP CD31.png]

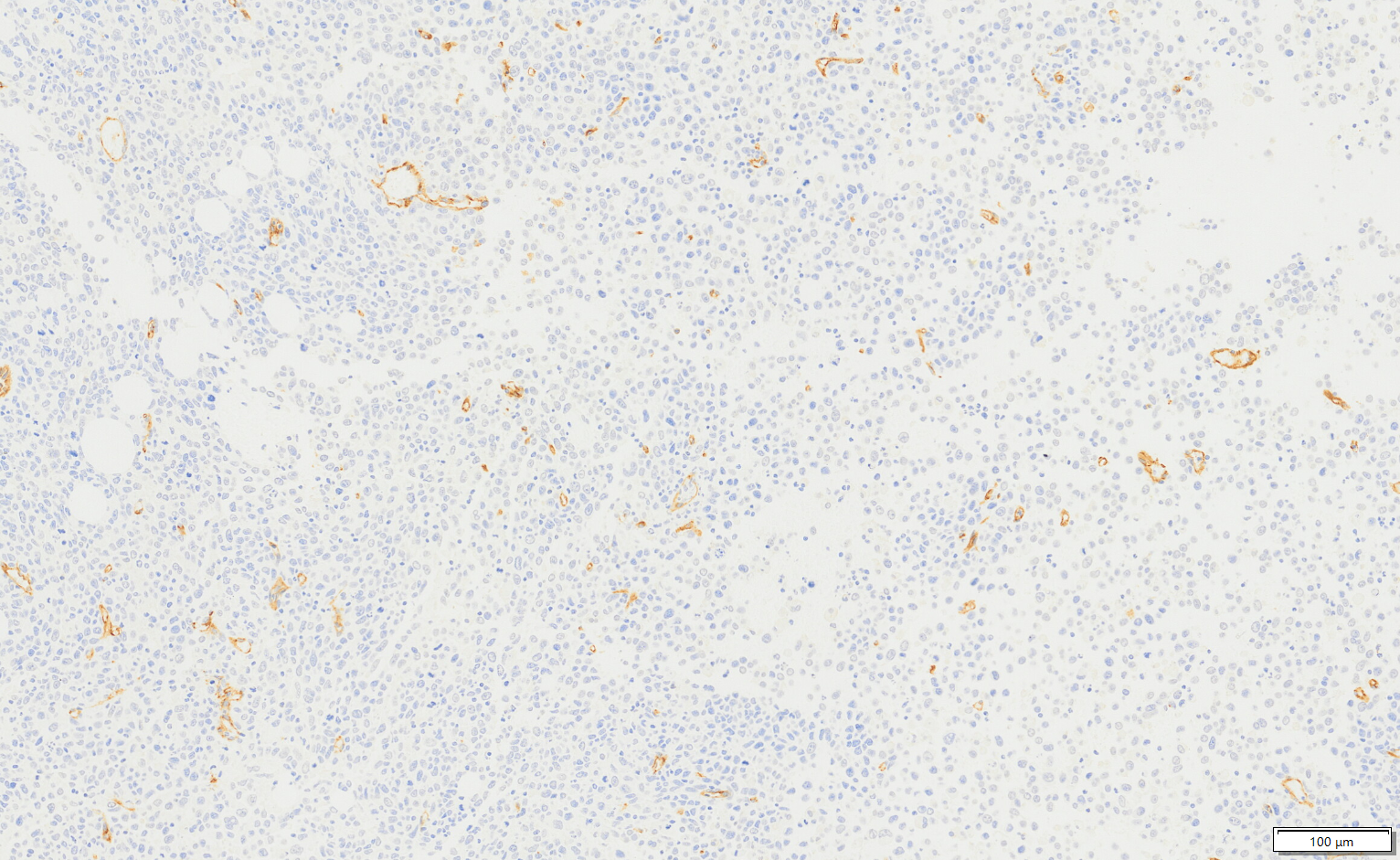

Supplement: Supplementary file 7 — Source data Fig. 5 [file 44319_2025_627_MOESM7_ESM.zip › Figure 5/5N/LLC Ctrl CD31.png]

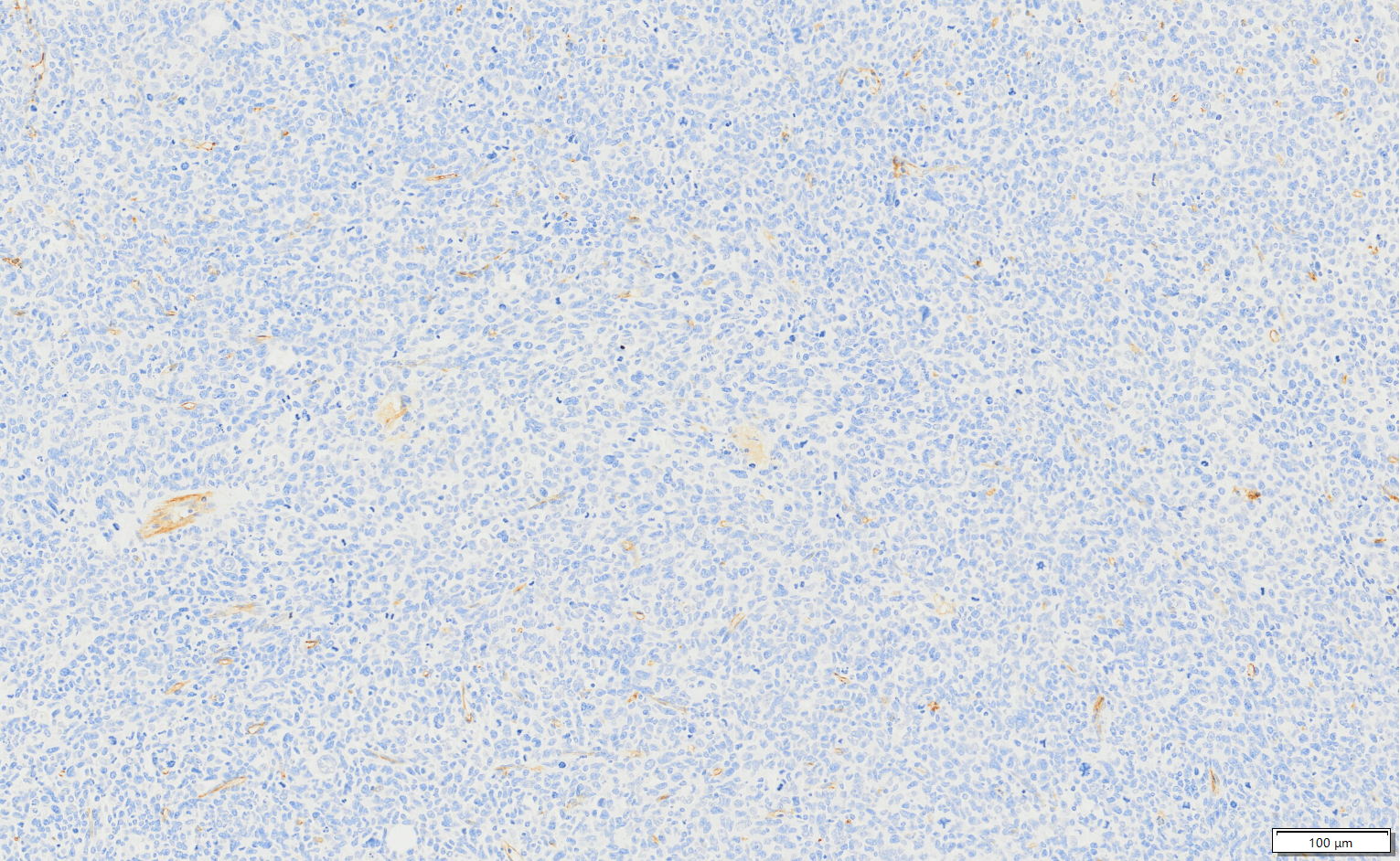

Supplement: Supplementary file 7 — Source data Fig. 5 [file 44319_2025_627_MOESM7_ESM.zip › Figure 5/5N/LLC SASP CD31.png]

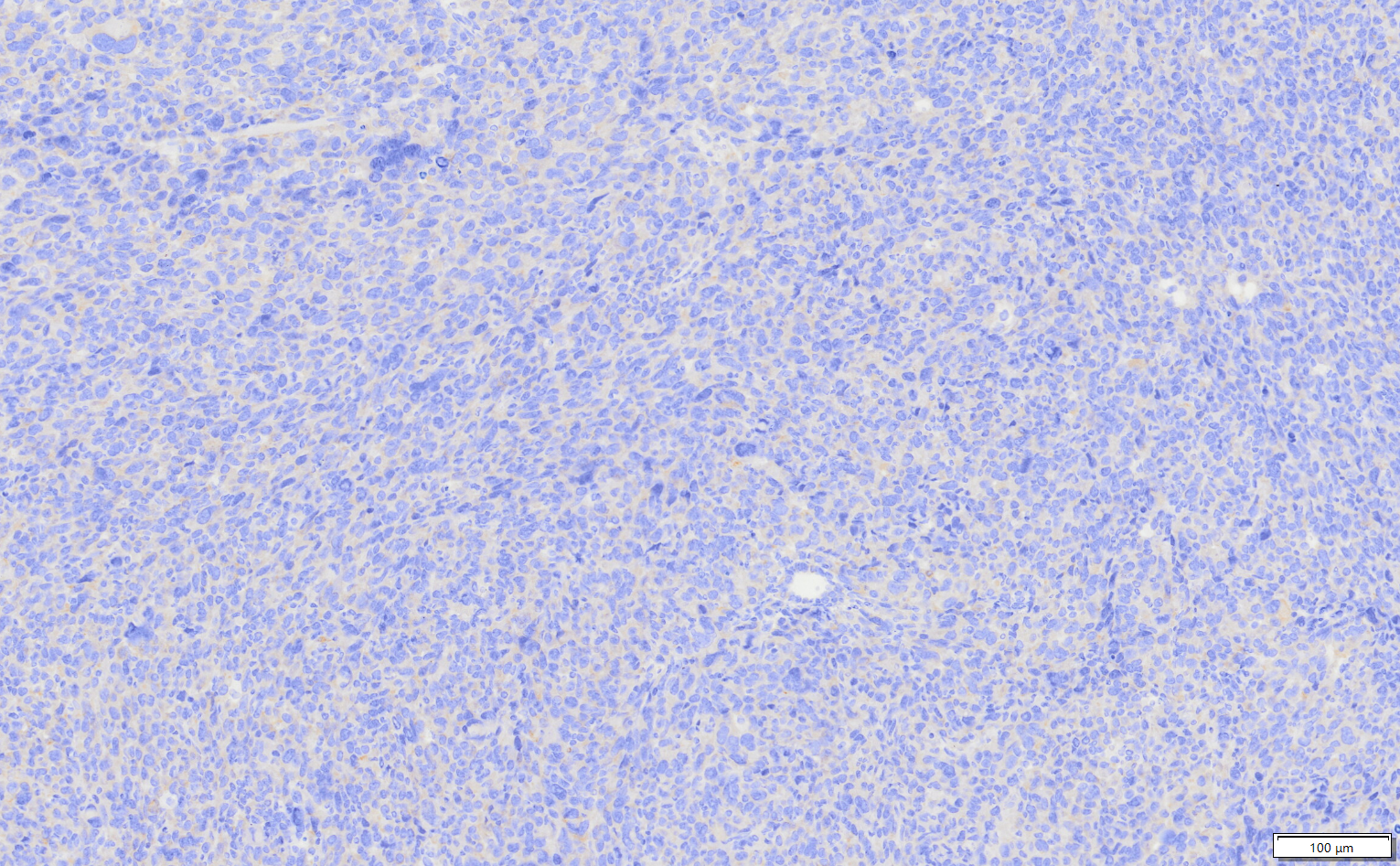

Supplement: Supplementary file 7 — Source data Fig. 5 [file 44319_2025_627_MOESM7_ESM.zip › Figure 5/5M/LLC A.f SASP VEGF-A.png]

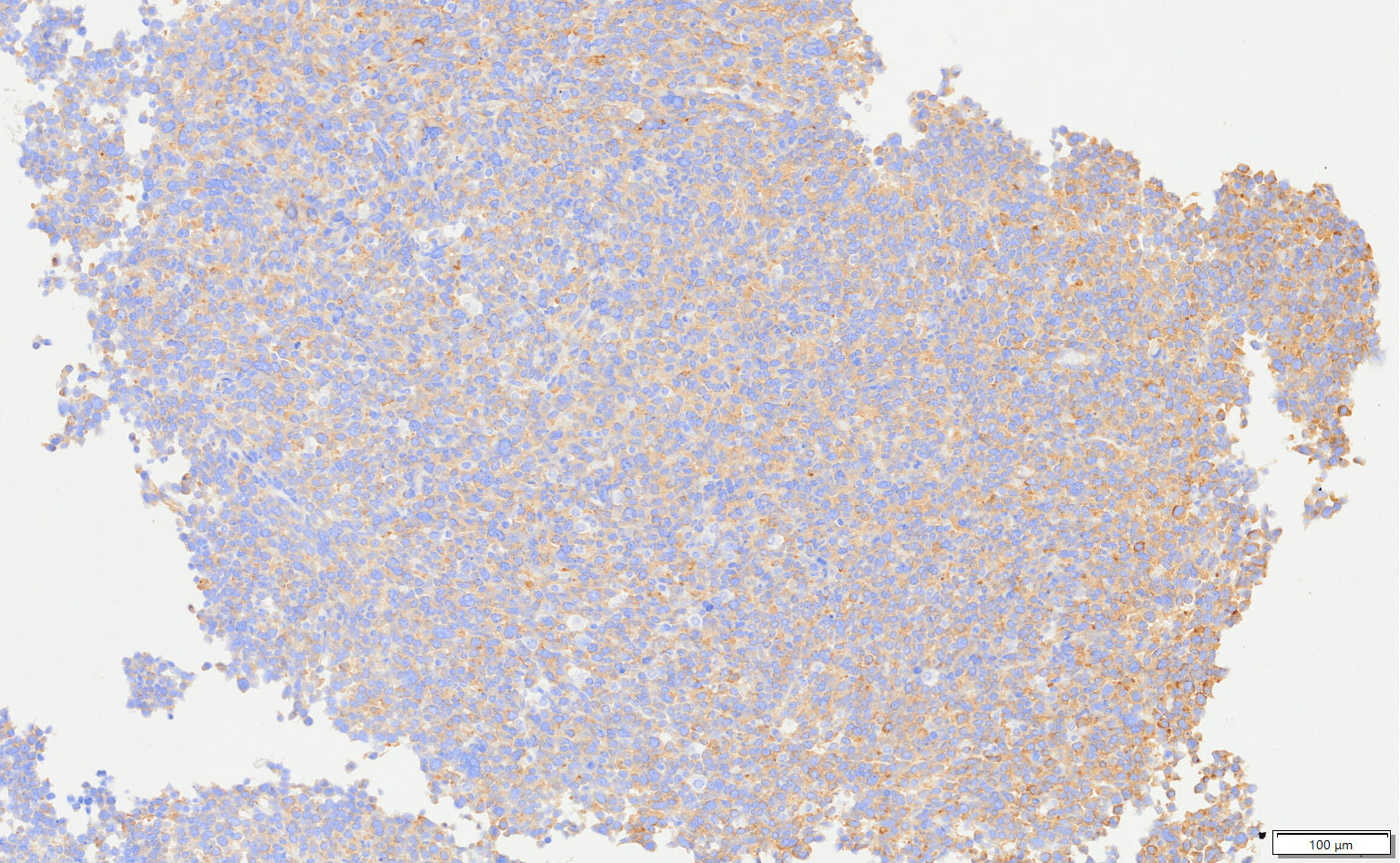

Supplement: Supplementary file 7 — Source data Fig. 5 [file 44319_2025_627_MOESM7_ESM.zip › Figure 5/5M/LLC A.f VEGF-A.png]

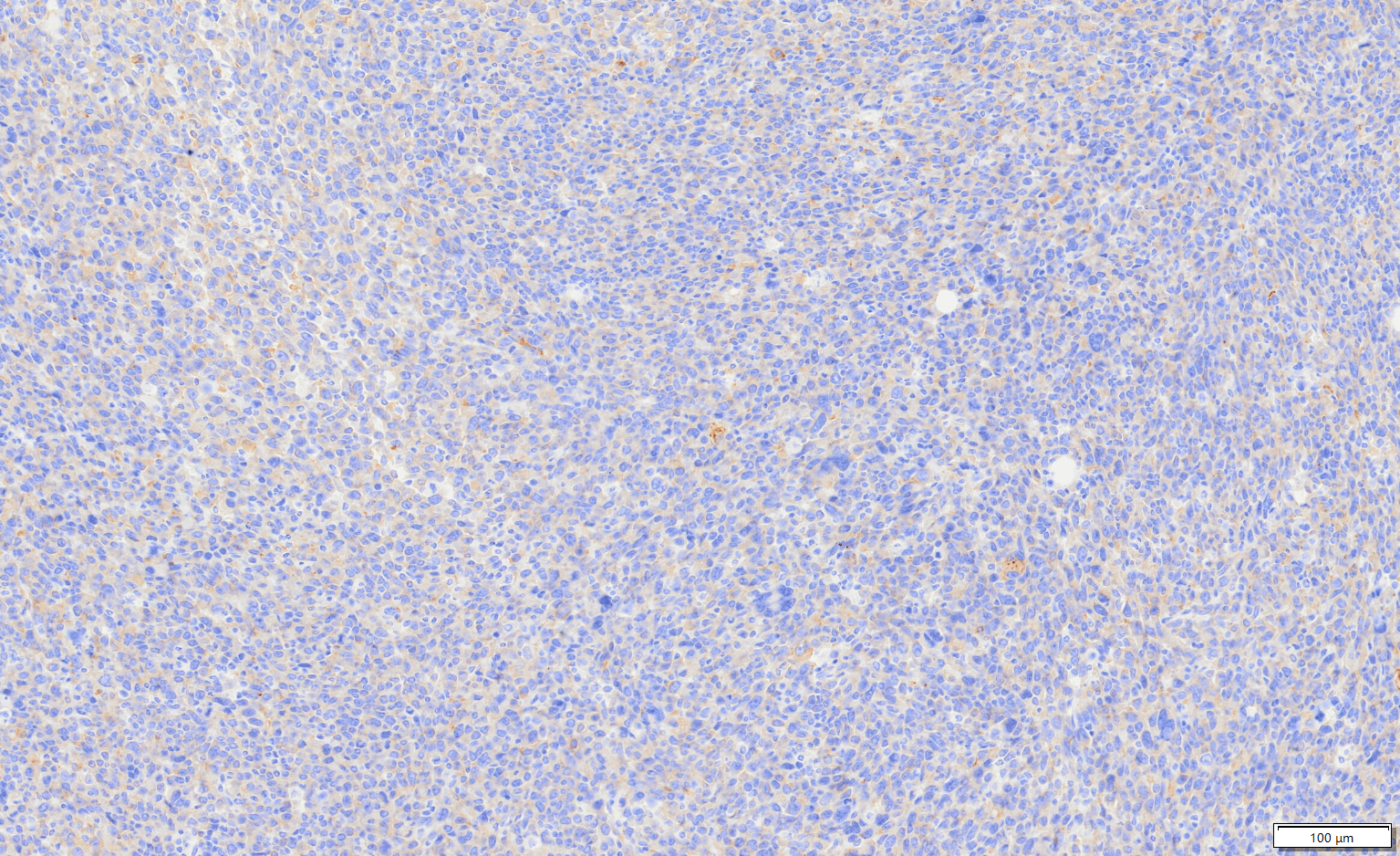

Supplement: Supplementary file 7 — Source data Fig. 5 [file 44319_2025_627_MOESM7_ESM.zip › Figure 5/5M/LLC Ctrl VEGF-A.png]

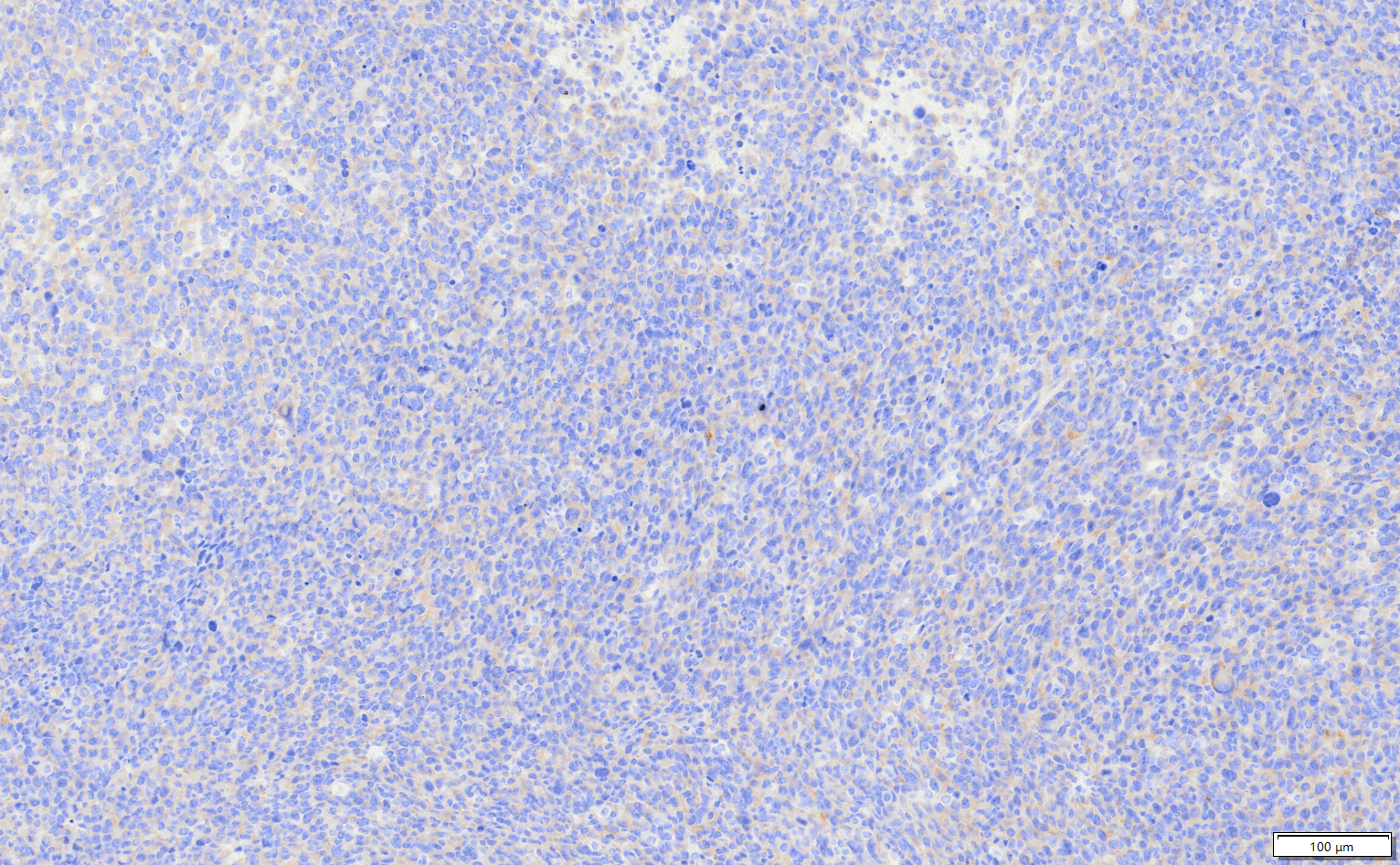

Supplement: Supplementary file 7 — Source data Fig. 5 [file 44319_2025_627_MOESM7_ESM.zip › Figure 5/5M/LLC sasp VEGF-A.png]

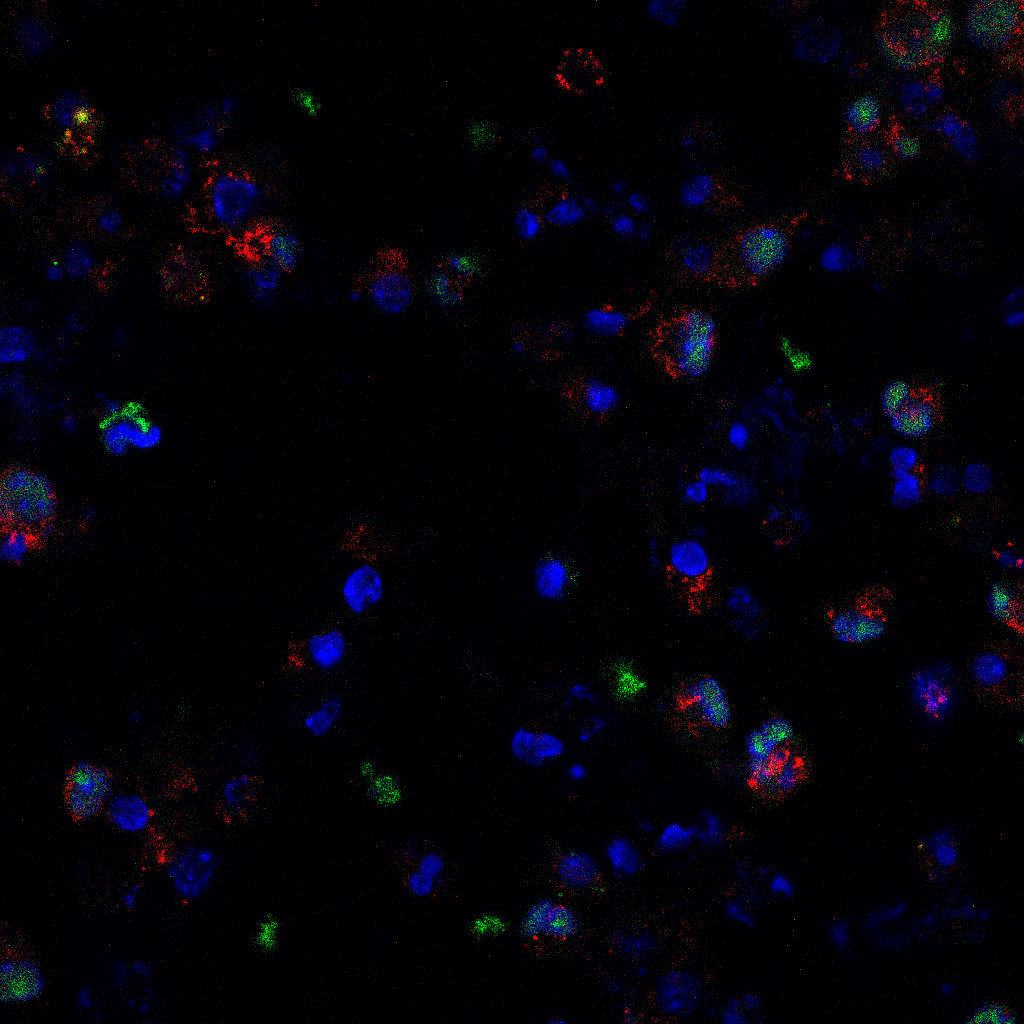

Supplement: Supplementary file 8 — Source data Fig. 6 [file 44319_2025_627_MOESM8_ESM.zip › Figure 6/6B/Ctrl/MDSC Ctrl Merged.tif]

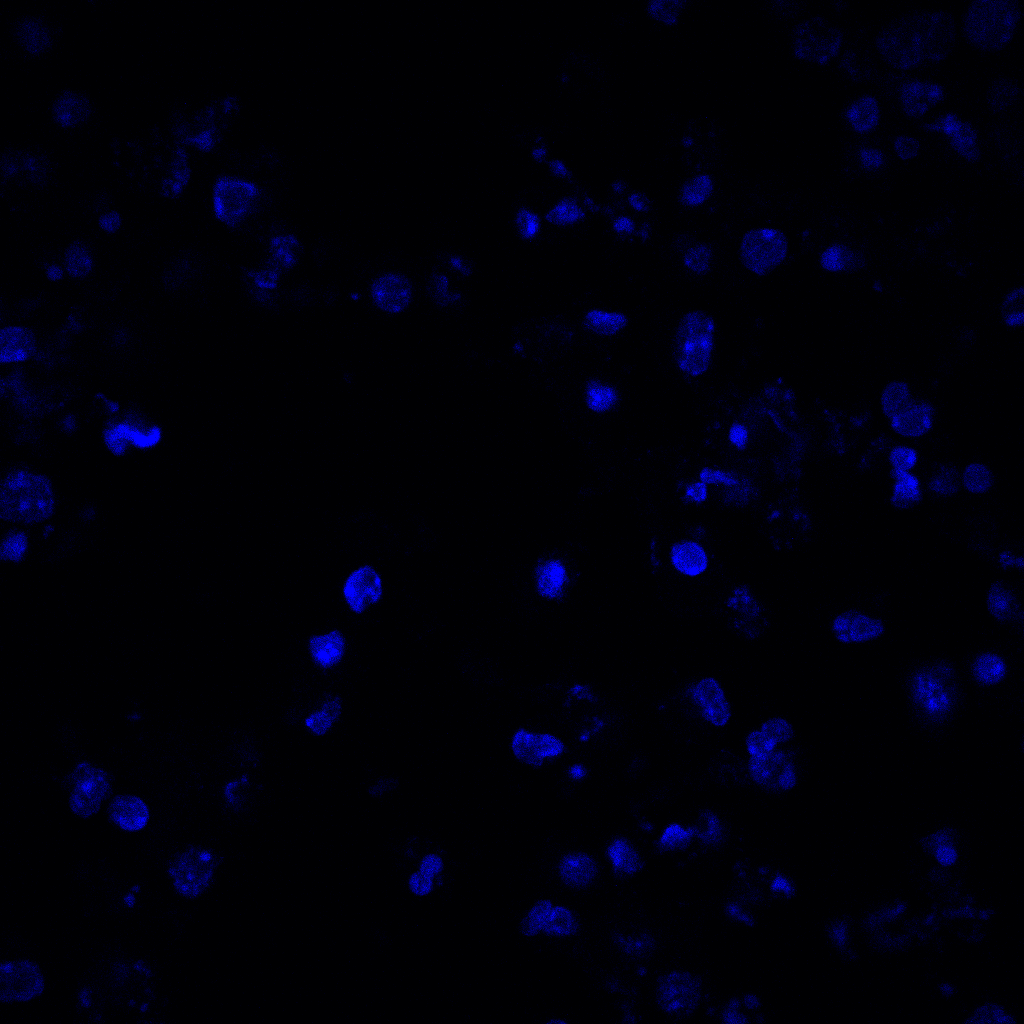

Supplement: Supplementary file 8 — Source data Fig. 6 [file 44319_2025_627_MOESM8_ESM.zip › Figure 6/6B/Ctrl/MDSC Ctrl DAPI.tif]

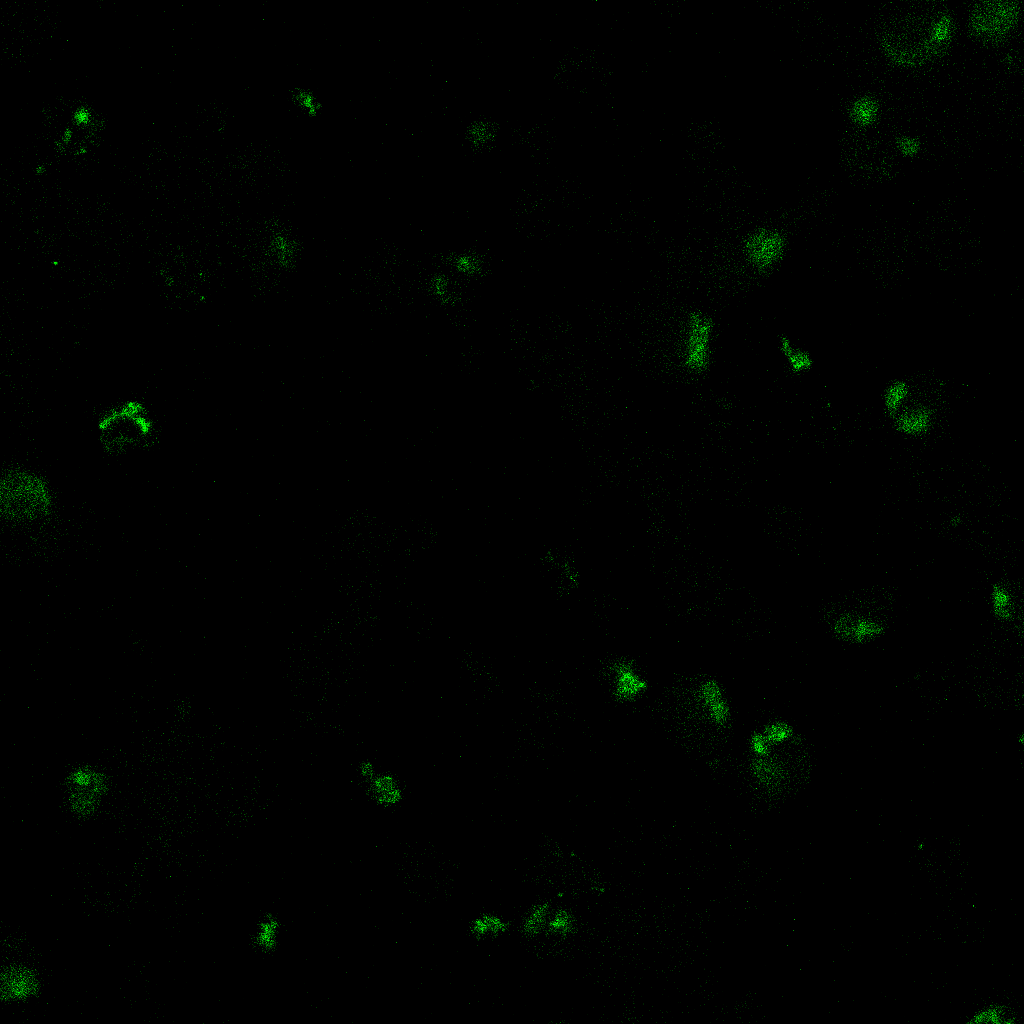

Supplement: Supplementary file 8 — Source data Fig. 6 [file 44319_2025_627_MOESM8_ESM.zip › Figure 6/6B/Ctrl/MDSC Ctrl HMGB1.tif]

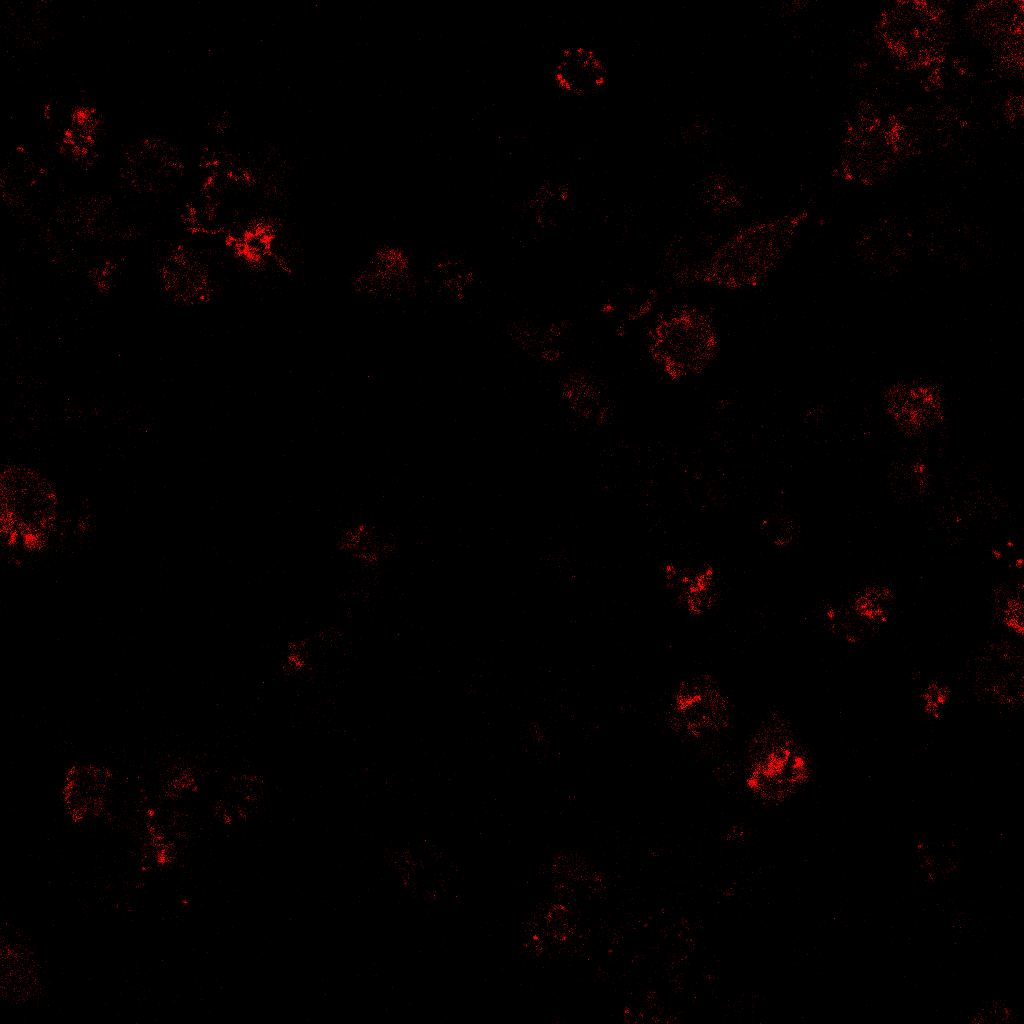

Supplement: Supplementary file 8 — Source data Fig. 6 [file 44319_2025_627_MOESM8_ESM.zip › Figure 6/6B/Ctrl/MDSC Ctrl Slc7a11.tif]

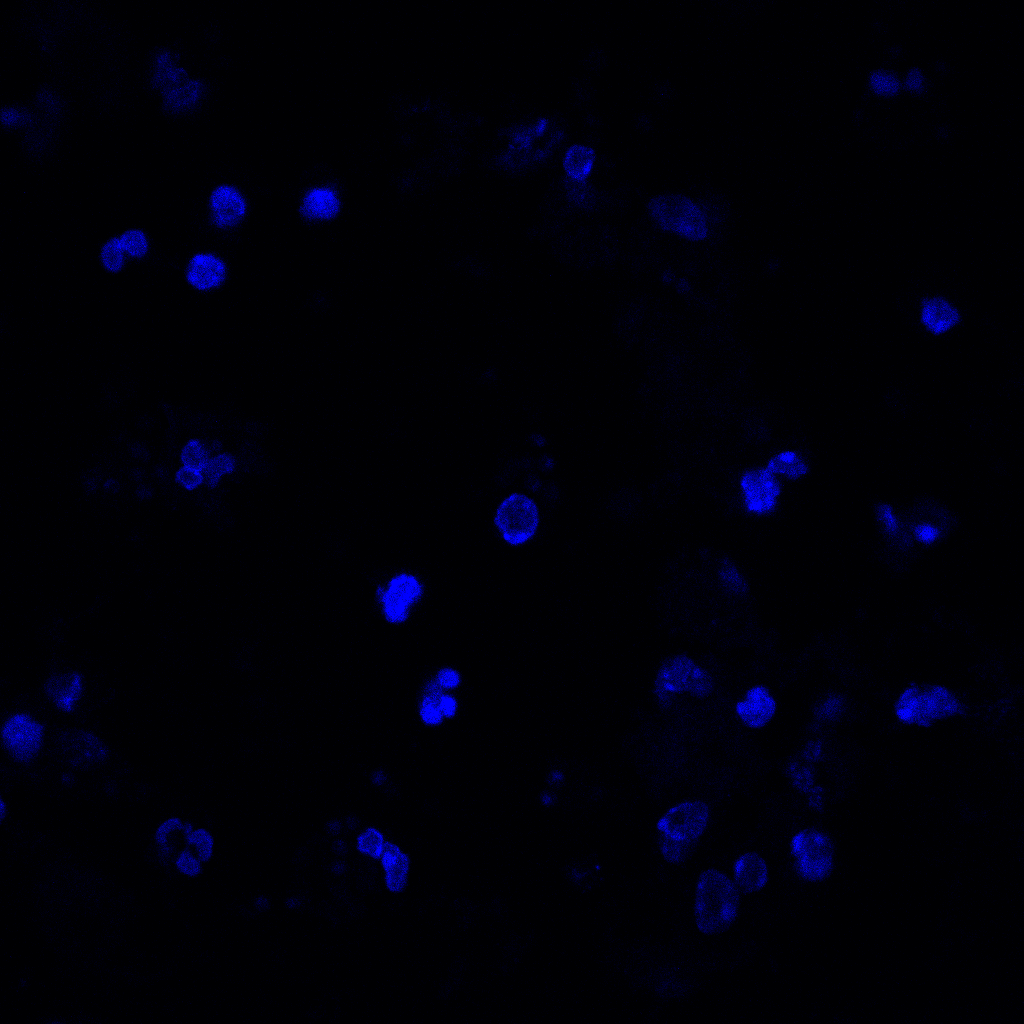

Supplement: Supplementary file 8 — Source data Fig. 6 [file 44319_2025_627_MOESM8_ESM.zip › Figure 6/6B/A.f/MDSC A.f DAPI.tif]

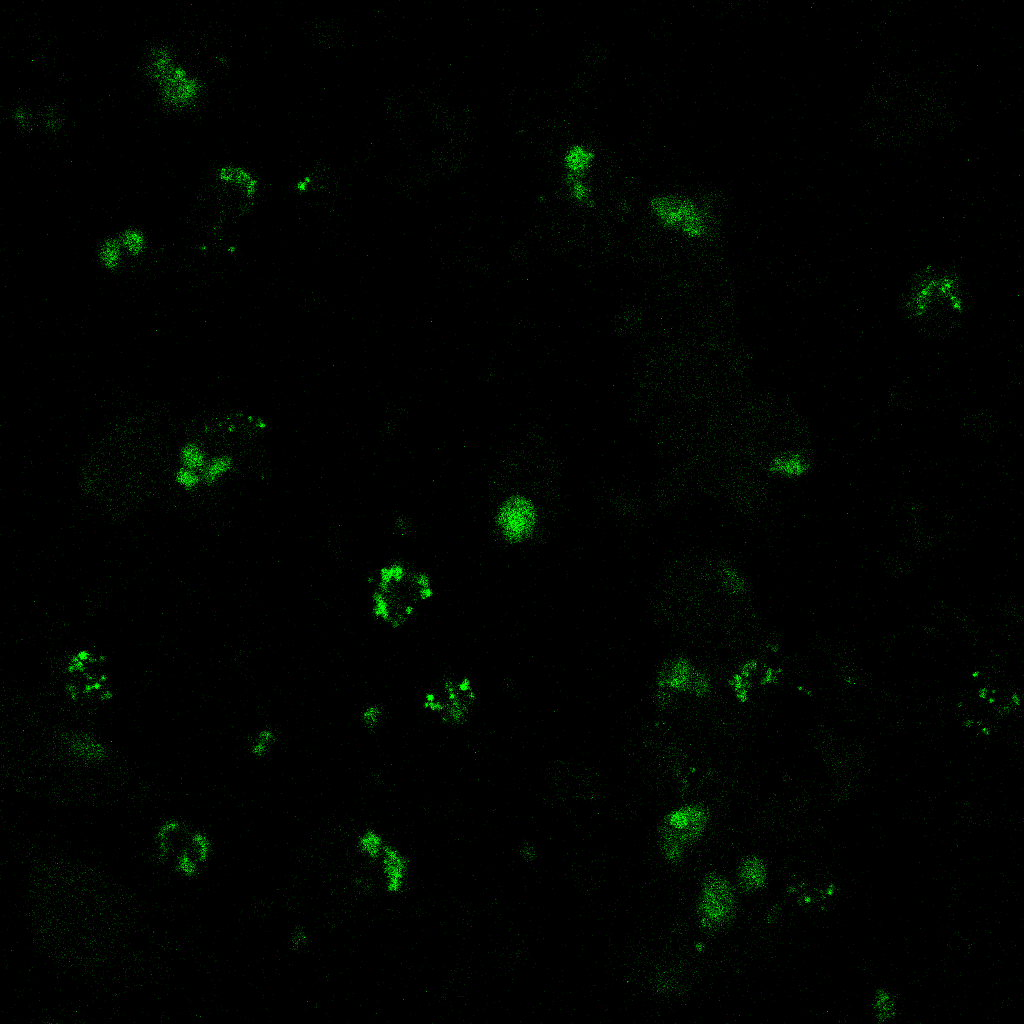

Supplement: Supplementary file 8 — Source data Fig. 6 [file 44319_2025_627_MOESM8_ESM.zip › Figure 6/6B/A.f/MDSC A.f Hmgb1.tif]

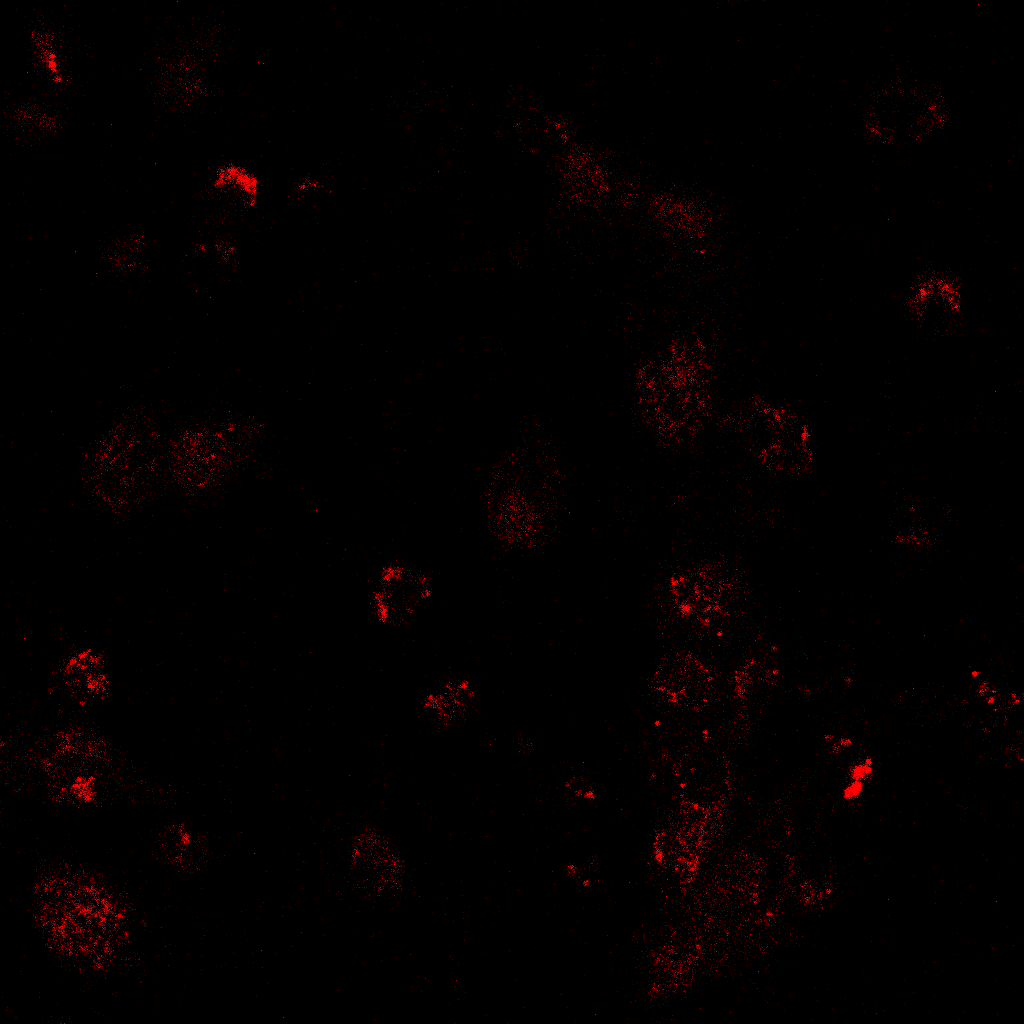

Supplement: Supplementary file 8 — Source data Fig. 6 [file 44319_2025_627_MOESM8_ESM.zip › Figure 6/6B/A.f/MDSC A.f Slc7a11.tif]

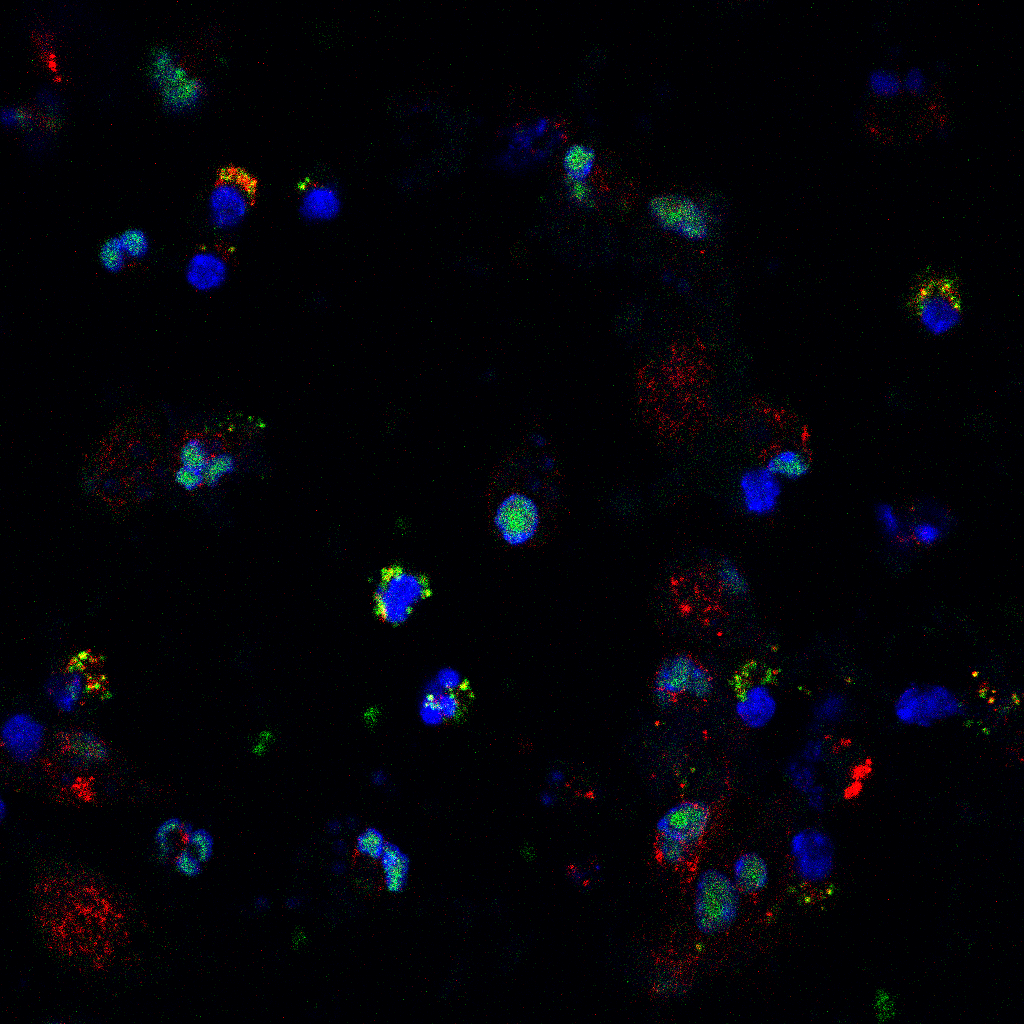

Supplement: Supplementary file 8 — Source data Fig. 6 [file 44319_2025_627_MOESM8_ESM.zip › Figure 6/6B/A.f/MDSC A.f merged.tif]

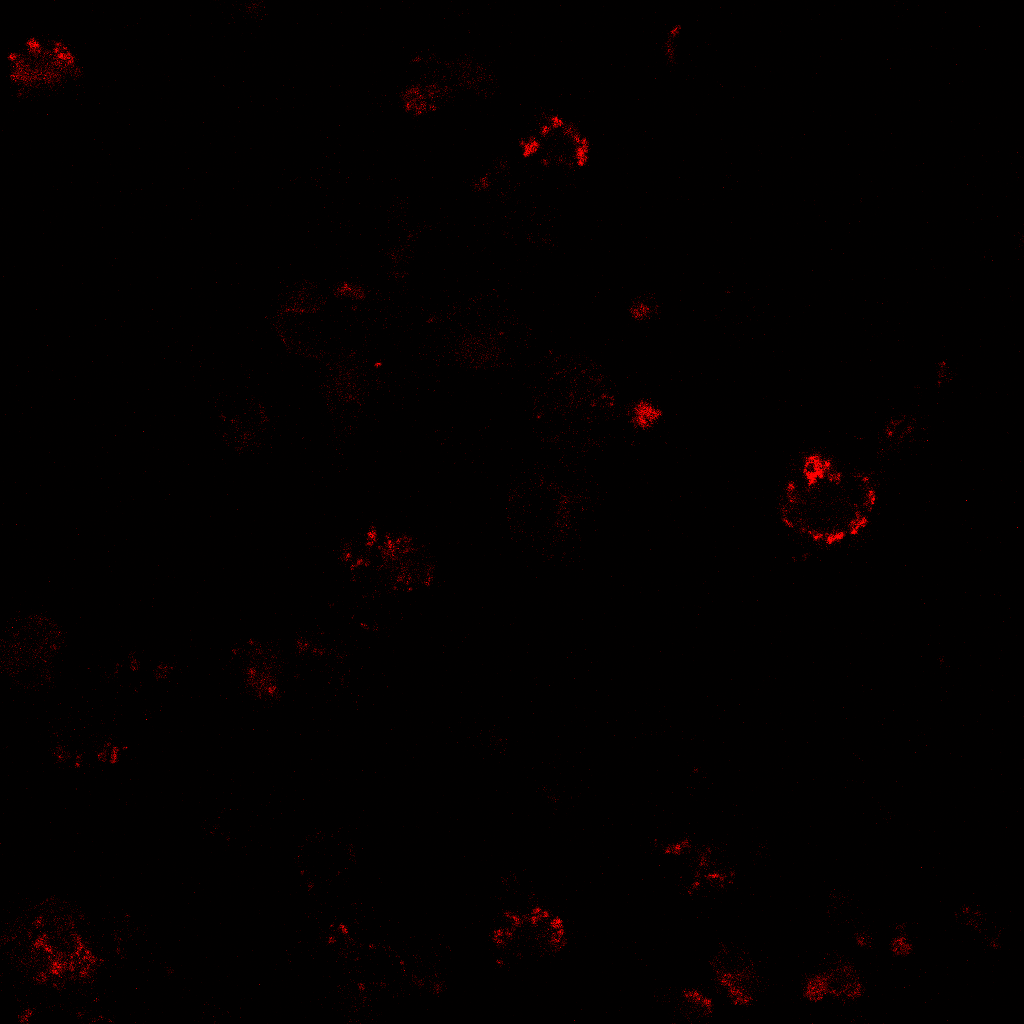

Supplement: Supplementary file 8 — Source data Fig. 6 [file 44319_2025_627_MOESM8_ESM.zip › Figure 6/6B/A.f Glycyrrhiz/MDSC A.f Glycyrrhiz Slc7a11.tif]

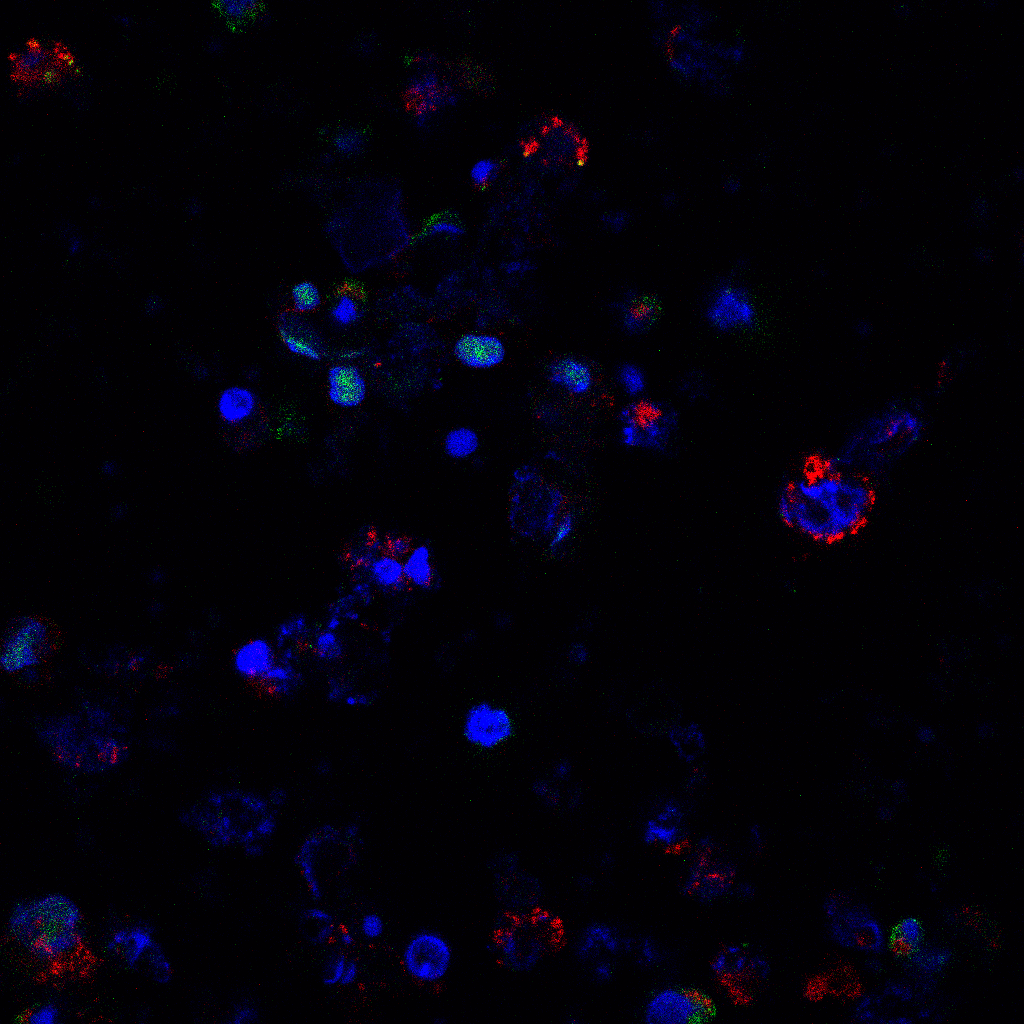

Supplement: Supplementary file 8 — Source data Fig. 6 [file 44319_2025_627_MOESM8_ESM.zip › Figure 6/6B/A.f Glycyrrhiz/MDSC A.f Glycyrrhiz merged.tif]

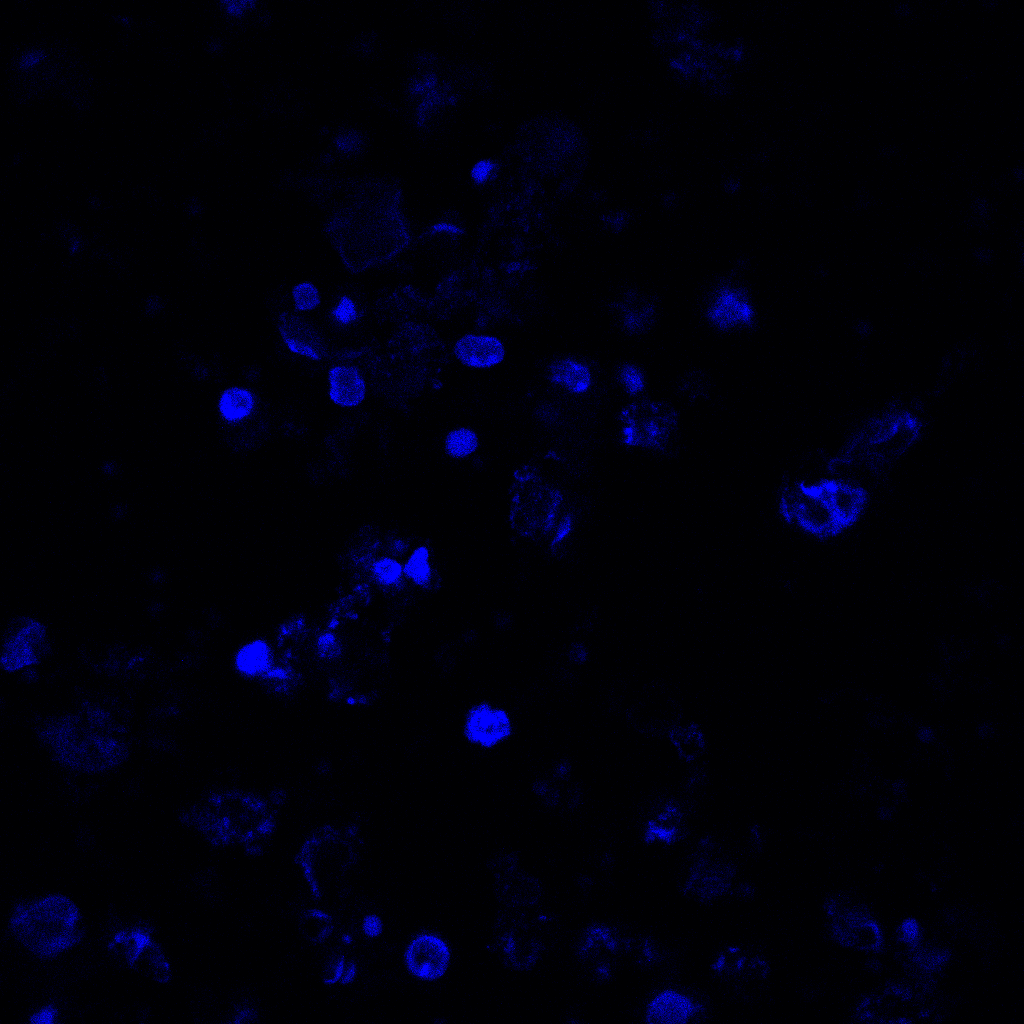

Supplement: Supplementary file 8 — Source data Fig. 6 [file 44319_2025_627_MOESM8_ESM.zip › Figure 6/6B/A.f Glycyrrhiz/MDSC A.f Glycyrrhiz DAPI.tif]

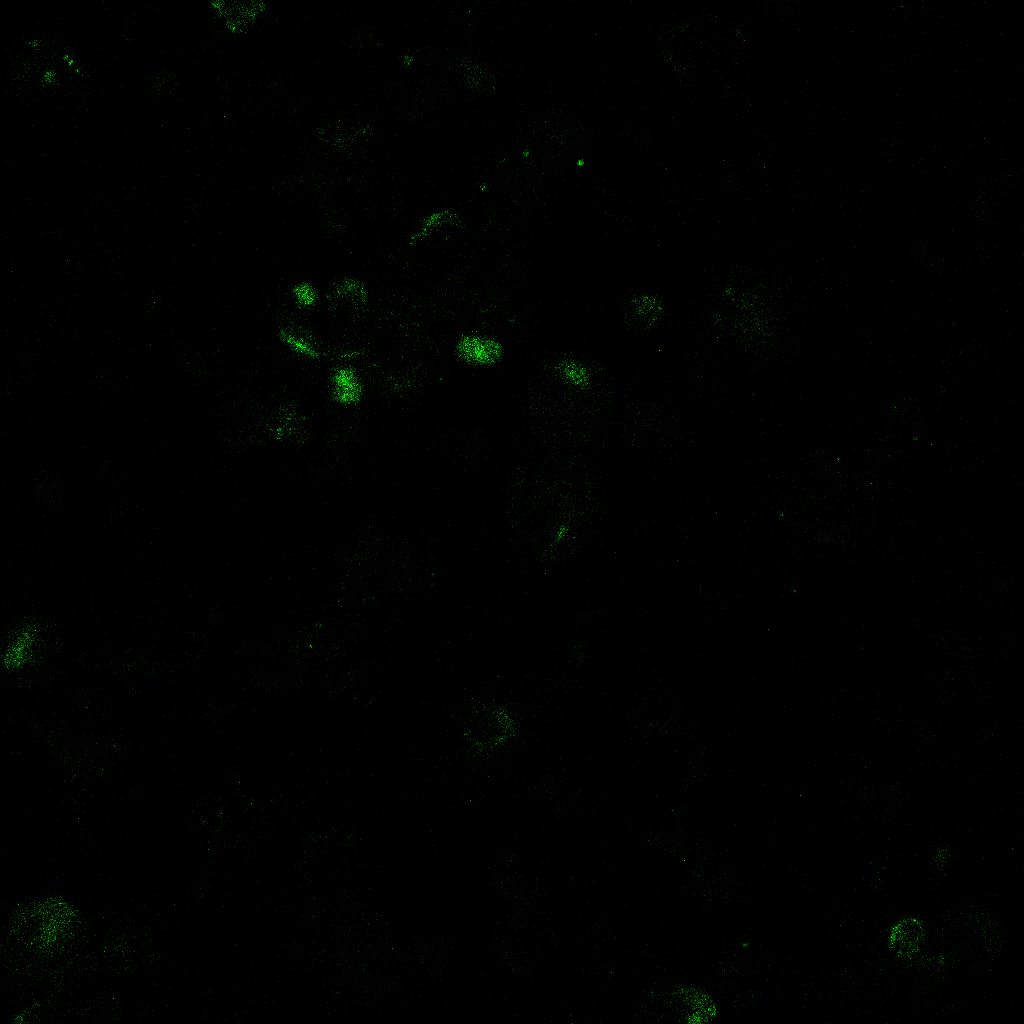

Supplement: Supplementary file 8 — Source data Fig. 6 [file 44319_2025_627_MOESM8_ESM.zip › Figure 6/6B/A.f Glycyrrhiz/MDSC A.f Glycyrrhiz Hmgb1.tif]

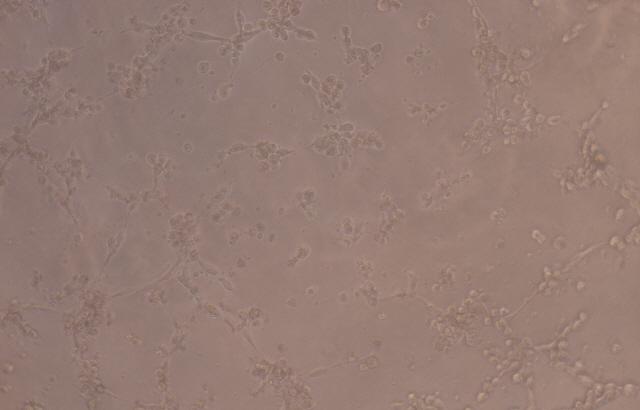

Supplement: Supplementary file 8 — Source data Fig. 6 [file 44319_2025_627_MOESM8_ESM.zip › Figure 6/6G/A.f Glycyrrhizin.jpg]

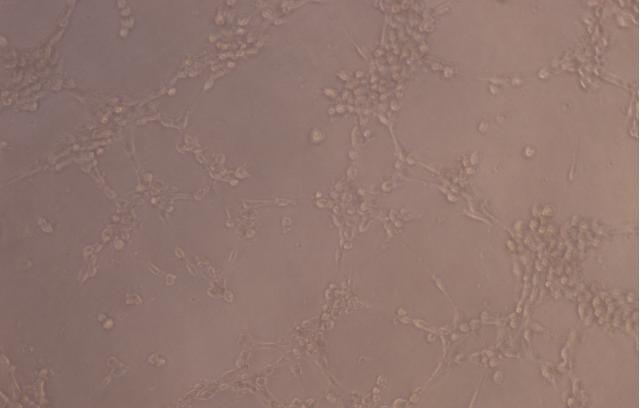

Supplement: Supplementary file 8 — Source data Fig. 6 [file 44319_2025_627_MOESM8_ESM.zip › Figure 6/6G/A.f.jpg]

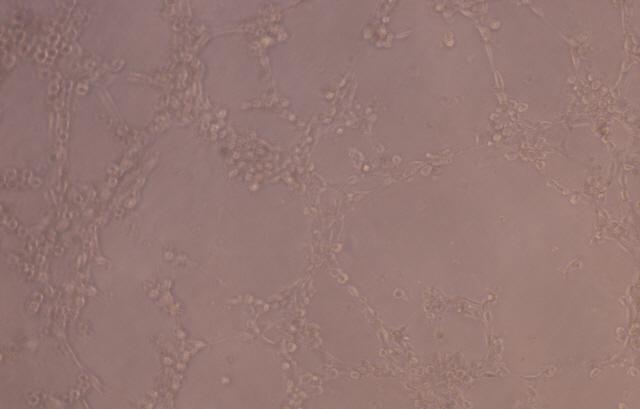

Supplement: Supplementary file 8 — Source data Fig. 6 [file 44319_2025_627_MOESM8_ESM.zip › Figure 6/6G/Ctrl.jpg]

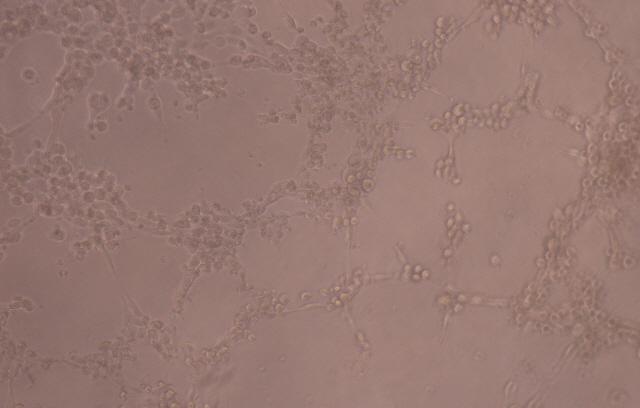

Supplement: Supplementary file 8 — Source data Fig. 6 [file 44319_2025_627_MOESM8_ESM.zip › Figure 6/6G/Glycyrrhizin.jpg]

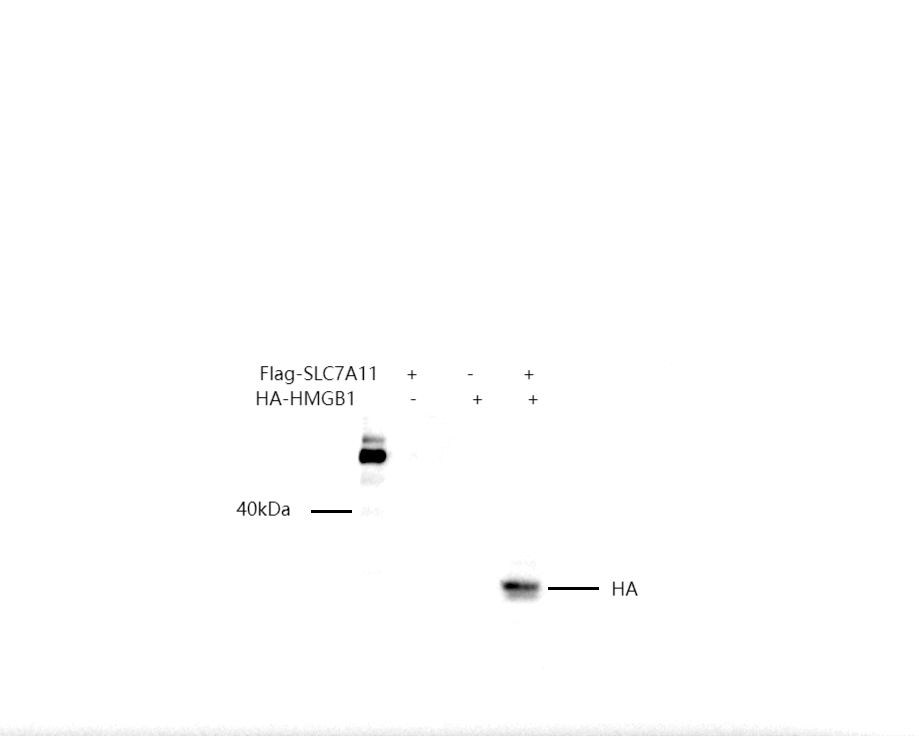

Supplement: Supplementary file 8 — Source data Fig. 6 [file 44319_2025_627_MOESM8_ESM.zip › Figure 6/6H/IP-FLAG IB-HA.png]

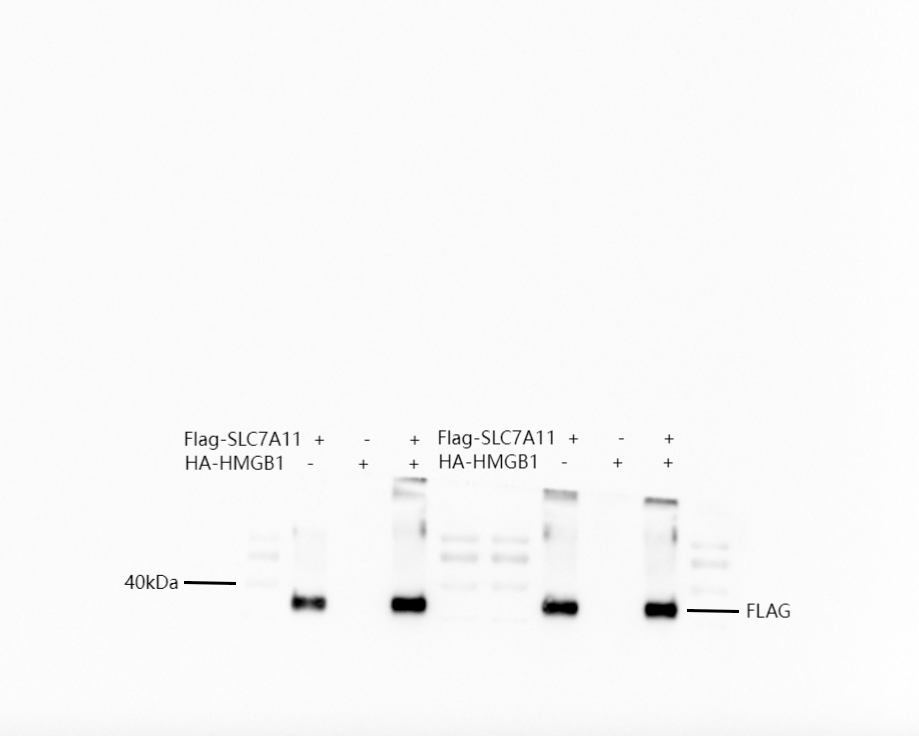

Supplement: Supplementary file 8 — Source data Fig. 6 [file 44319_2025_627_MOESM8_ESM.zip › Figure 6/6H/IP-FLAG IB-FLAG.png]

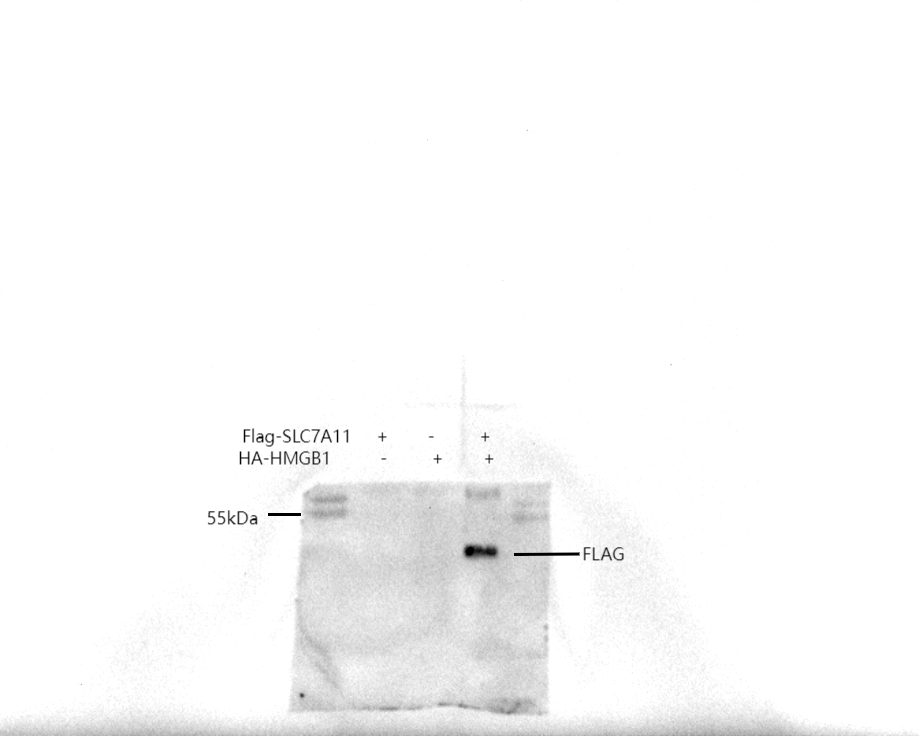

Supplement: Supplementary file 8 — Source data Fig. 6 [file 44319_2025_627_MOESM8_ESM.zip › Figure 6/6H/IP-HA IB-FLAG.png]

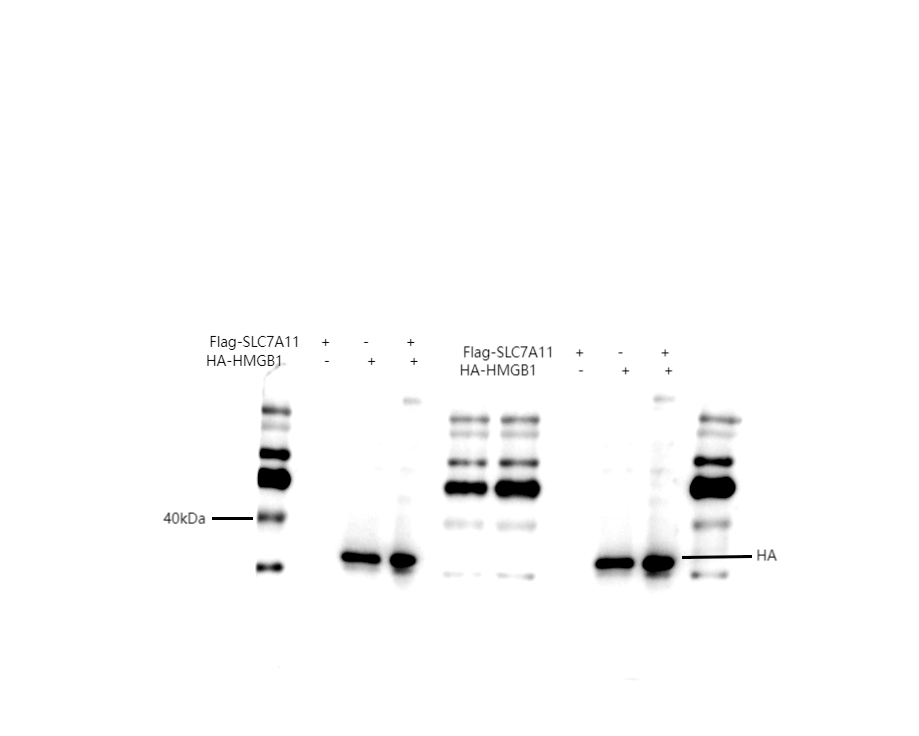

Supplement: Supplementary file 8 — Source data Fig. 6 [file 44319_2025_627_MOESM8_ESM.zip › Figure 6/6H/IP-HA IB-HA.png]

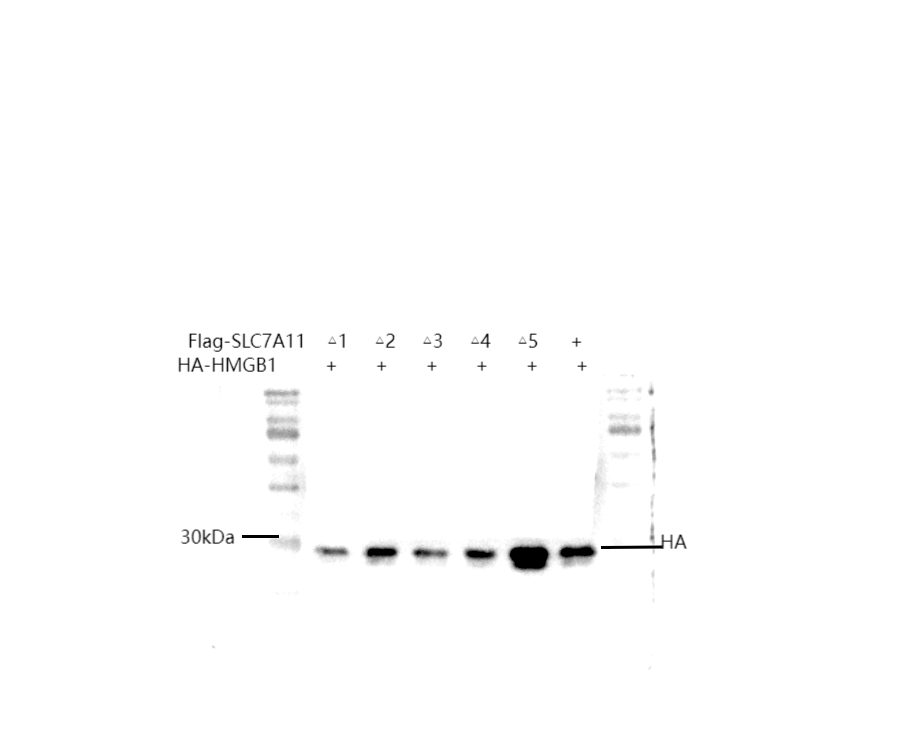

Supplement: Supplementary file 8 — Source data Fig. 6 [file 44319_2025_627_MOESM8_ESM.zip › Figure 6/6I/XCT+mutation+hMGB1+HA-HA-1.png]

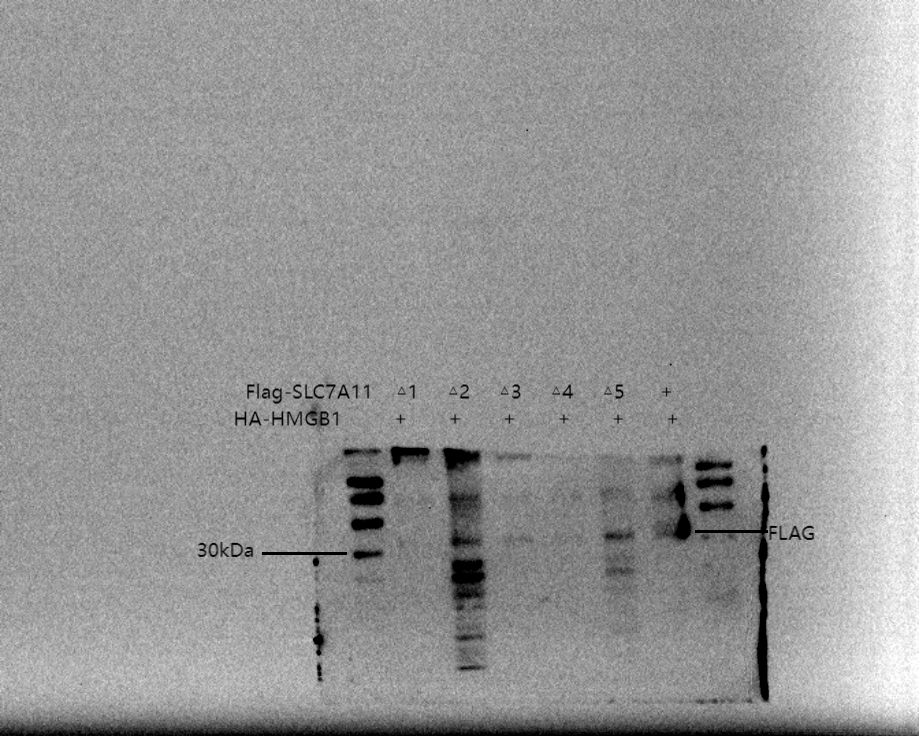

Supplement: Supplementary file 8 — Source data Fig. 6 [file 44319_2025_627_MOESM8_ESM.zip › Figure 6/6I/XCT+mutation+hMGB1+HA-flag-4.png]
