# Supplementary material for: Aspergillus fumigatus promotes tumor angiogenesis via SLC7A11 on myeloid-derived suppressor cells
Source: EMBO Rep. 2025 Nov 17;26(24):6266–91. doi: 10.1038/s44319-025-00627-x (PMC12715260; doi:10.1038/s44319-025-00627-x)
Supplement: Supplementary file 9 — Expanded View Figures [file 44319_2025_627_MOESM9_ESM.pdf]

Expanded View Figures

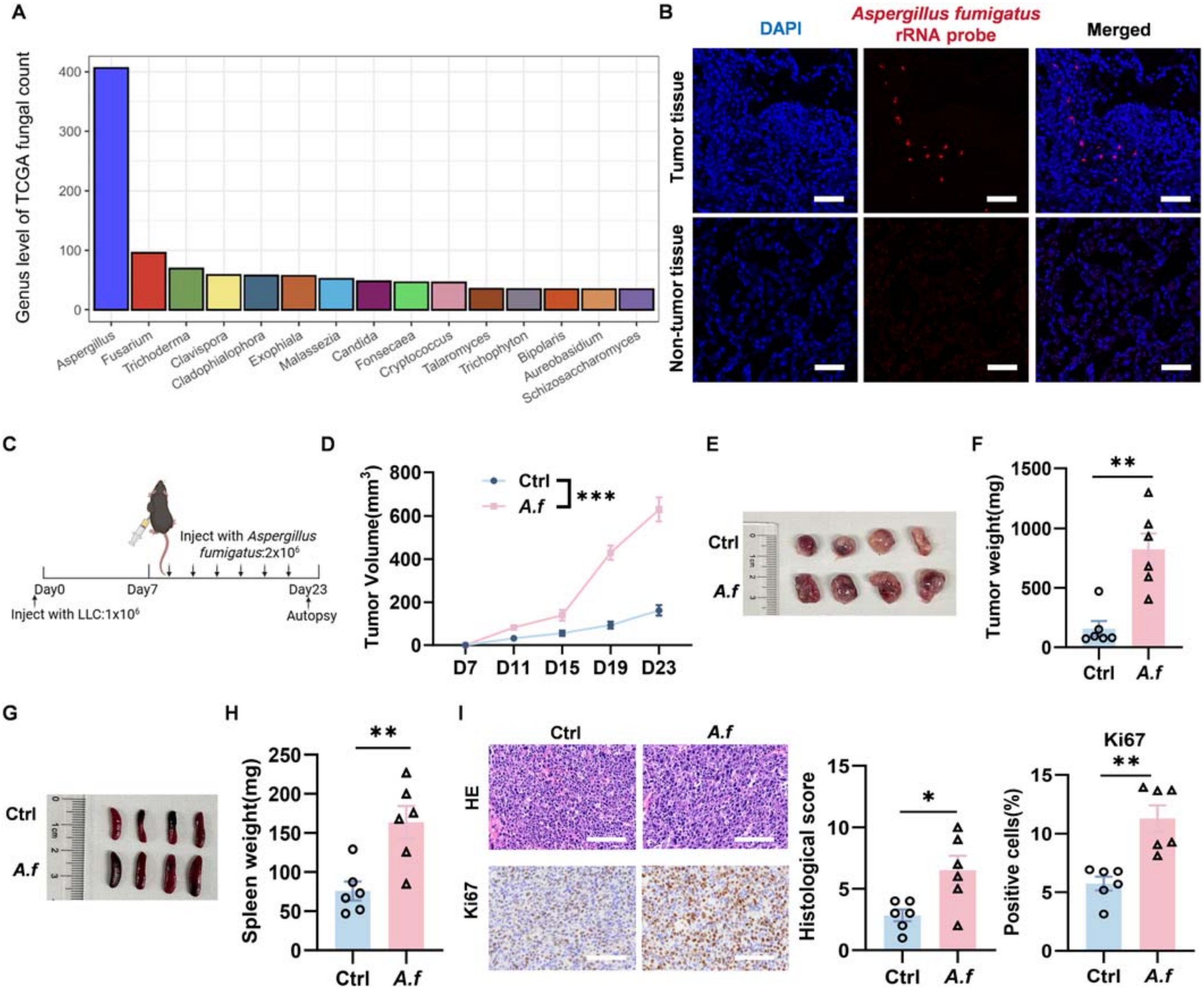

Figure EV1. *A. fumigatus* promotes the development of lung cancer.

(**A**) The distribution of different fungal genera in LUAD was analyzed based on the decontaminated TCGA fungal count data. (**B**) Detection of *A. fumigatus* in human lung cancer by FISH (scale bars, 50 µm). (**C**) Lewis lung cancer cells (1×10^6) were injected subcutaneously into the right flank of mice ($n = 6$ per group). Live *A. fumigatus* (2×10^6) was injected peritumorally three times per week for two weeks. All tumor-bearing mice were executed on day 23 (Created with BioRender.com). (**D**) Tumor-volume curve was calculated after injection every four days ($p = 0.0006$) ($n = 6$ biological replicates). (**E**) Representative images of tumors from different groups. (**F**) Tumors isolated from mice were weighed ($p = 0.0011$) ($n = 6$ biological replicates). (**G**) Representative images of spleens from different groups. (**H**) Spleens isolated from mice were weighed ($p = 0.0046$) ($n = 6$ biological replicates). (**I**) Histological analysis of tumors was shown by HE staining (scale bars, 100 µm) ($p = 0.0161$) Immunohistochemical analysis of Ki67 in tumors (scale bars, 100 µm) ($p = 0.0014$) ($n = 6$ biological replicates). Data information: Data with error bars are represented as mean ± SEM. Tumor growth curves were analyzed by two-way ANOVA. Other data were analyzed using unpaired Student's t-test. *$p < 0.05$, **$p < 0.01$, ***$p < 0.001$ as determined by unpaired Student's t-test or two-way ANOVA.

 © The Author(s)

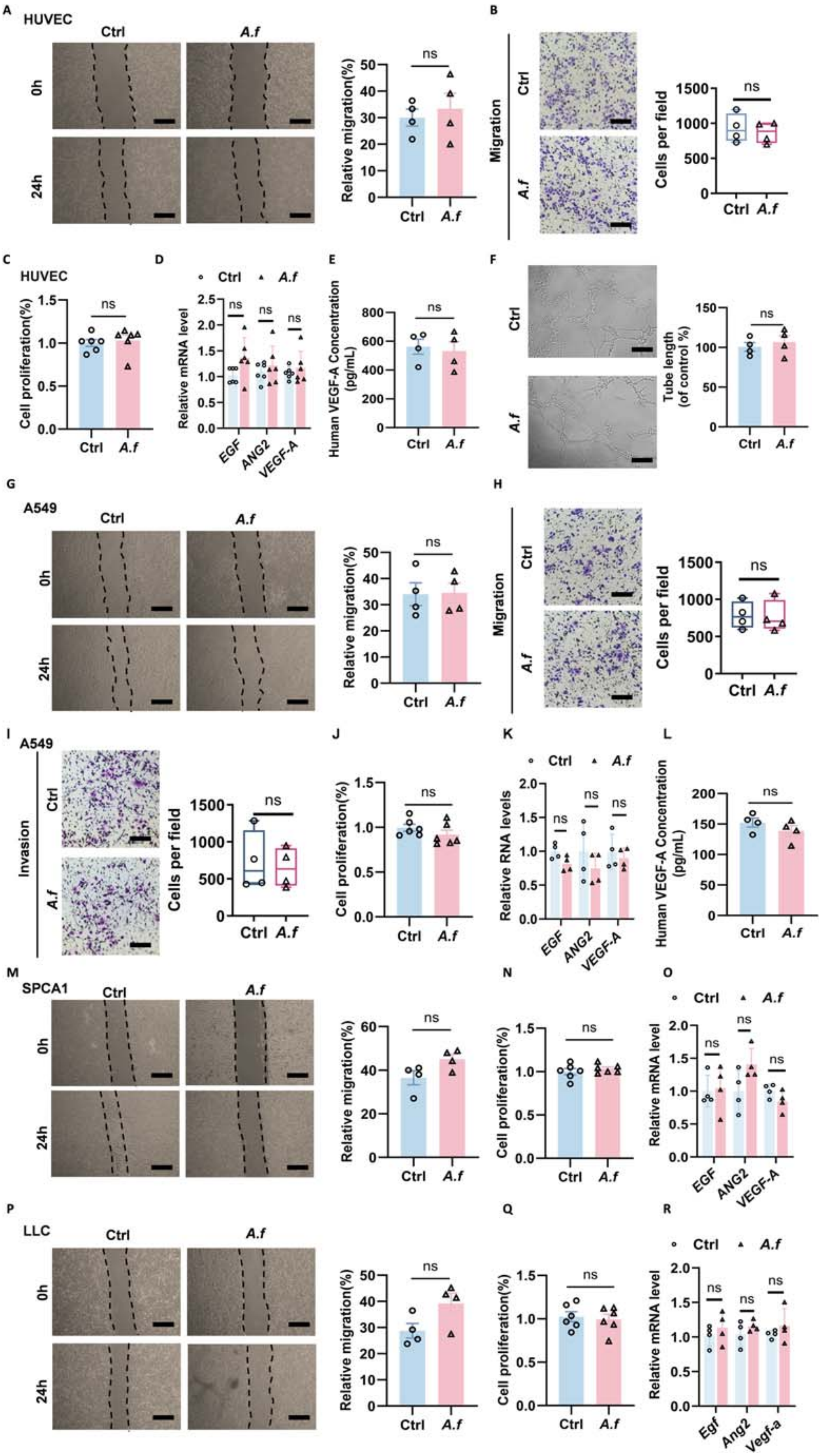
A
HUVEC
Ctrl
A.f
0h
24h
Relative migration(%)
ns
Ctrl
A.f
B
Migration
Ctrl
A.f
Cells per field
ns
C
HUVEC
Cell proliferation(%)
ns
D
Ctrl
A.f
Relative mRNA level
ns
EGF
ANG2
VEGF-A
E
Human VEGF-A Concentration (pg/mL)
ns
F
Tube length (of control %)
ns
G
A549
Ctrl
A.f
0h
24h
Relative migration(%)
ns
H
Migration
Cells per field
ns
I
A549
Invasion
Ctrl
A.f
Cells per field
ns
J
Cell proliferation(%)
ns
K
Relative RNA levels
ns
EGF
ANG2
VEGF-A
L
Human VEGF-A Concentration (pg/mL)
ns
M
SPCA1
Ctrl
A.f
0h
24h
Relative migration(%)
ns
N
Cell proliferation(%)
ns
O
Relative mRNA level
ns
EGF
ANG2
VEGF-A
P
LLC
Ctrl
A.f
0h
24h
Relative migration(%)
ns
Q
Cell proliferation(%)
ns
R
Relative mRNA level
ns
Egf
Ang2
Vegf-a

Figure EV2. *A. fumigatus* has no effect on migratory, cell viability and pro-angiogenic function of endothelial cells and lung cancer cells.

Heat-inactivated *A. fumigatus* (MOI = 2) was used in the co-culture with HUVEC cells. (**A**, **B**) The migratory ability of HUVEC cells was examined by scratch assasy (scale bars, 500 µm) (p = 0.6314) and Transwell migration assay (scale bars, 200 µm) (p = 0.6492). For Ctrl in box plot: Minima = 728; Maxima = 1197; Centre = 895; Box bounds = (774.5, 1083); Lower Whisker = 728; Upper Whisker = 1197; (25th percentile, 75th percentile) = (774.5, 1083). For *A. f* in box plot: Minima = 700; Maxima = 1001; Centre = 885.5; Box bounds = (741, 995); Lower Whisker = 700; Upper Whisker = 1001 (25th percentile, 75th percentile) = (741, 995) (n = 4 biological replicates). (**C**) The cell viability of HUVEC cells was detected using CCK8 (p = 0.3095) (n = 6 biological replicates). (**D**) The gene expression related to angiogenesis was detected by qPCR. (*EGF*: p = 0.0649; *ANG2*: p = 0.4822; *VEGF-A*: p = 0.4939) (n = 6 biological replicates). (**E**) ELISA analysis of VEGF-A secretion in the culture supernatant (p = 0.7275) (n = 4 biological replicates). (**F**) The lumen-forming capacity of HUVEC cells was examined by tube-formation assay (scale bars, 200 µm) (p = 0.5628) (n = 4 biological replicates). Heat-inactivated *A. fumigatus* (MOI = 2) was used in the co-culture with A549 cells. (**G**, **H**) The migratory ability was examined by scratch assay (scale bars, 500 µm) (p = 0.9337) and Transwell migration assay (scale bars, 200 µm) (p = 0.9053). For Ctrl in box plot: Minima = 594; Maxima = 1019; Centre = 764; Box bounds = (649, 921.5); Lower Whisker = 594; Upper Whisker = 1019; (25th percentile, 75th percentile) = (649, 921.5). For *A. f* in box plot: Minima = 583; Maxima = 1078; Centre = 705; Box bounds = (632.5, 903); Lower Whisker = 583; Upper Whisker = 1078; (25th percentile, 75th percentile) = (632.5, 903) (n = 4 biological replicates). (**I**) The invasion ability was examined by Transwell invasion assay (scale bars, 200 µm) (p = 0.7494). For Ctrl in box plot: Minima = 428; Maxima = 1284; Centre = 608; Box bounds = (438, 1026); Lower Whisker = 428; Upper Whisker = 1284; (25th percentile, 75th percentile) = (438, 1026). For *A. f* in box plot: Minima = 387; Maxima = 951; Centre = 634.5; Box bounds = (430.5, 873); Lower Whisker = 387; Upper Whisker = 951; (25th percentile, 75th percentile) = (430.5, 873) (n = 4 biological replicates). (**J**) The cell viability was assessed by CCK8 (p = 0.2511) (n = 4 biological replicates). (**K**) The gene expression related to angiogenesis was detected by qPCR (*EGF*: p = 0.0565; *ANG2*: p = 0.3317; *VEGF-A*: p = 0.5663) (n = 4 biological replicates). (**L**) VEGF-A secretion was analyzed by ELISA (p = 0.2824) (n = 4 biological replicates). Heat-inactivated *A. fumigatus* (MOI = 2) was used in the co-culture with SPCA1 cells. (**M**) The migratory ability was examined by scratch assay (scale bars, 500 µm) (p = 0.0735) (n = 4 biological replicates). (**N**) The cell viability was assessed by CCK8 (p = 0.4244) (n = 6 biological replicates). (**O**) The gene expression related to angiogenesis was detected by qPCR. (*EGF*: p = 0.6857; *ANG2*: p = 0.0862; *VEGF-A*: p = 0.1387) (n = 4 biological replicates). Heat-inactivated *A. fumigatus* (MOI = 2) was used in the co-culture with LLC cells. (**P**) The migratory ability was examined by scratch test (scale bars, 500 µm) (p = 0.0747) (n = 4 biological replicates). (**Q**) The cell viability was assessed by CCK8 (p = 0.7188) (n = 6 biological replicates). (**R**) The gene expression related to angiogenesis was detected by qPCR. (*Egf*: p = 0.4545; *Ang2*: p = 0.2894; *Vegf-a*: p = 0.3724) (n = 4 biological replicates). Data information: Data with error bars are represented as mean ± SEM. Normality was assessed using the Shapiro-Wilk test. Two-group comparisons used the unpaired t-test (normal) or Mann-Whitney U test (non-normal). ns indicating no significance.

© The Author(s)

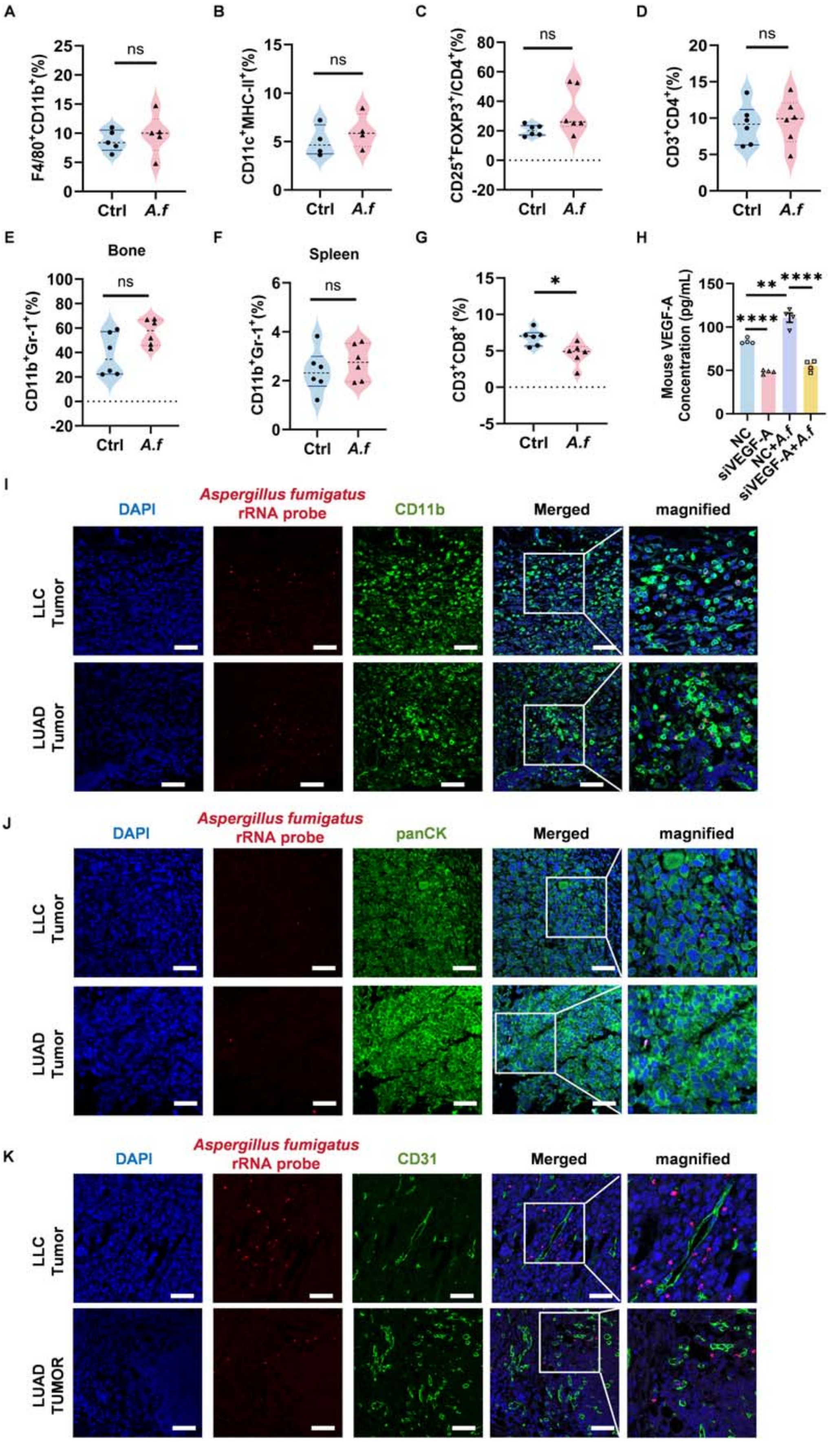

A
F4/80+CD11b+(%)
ns
Ctrl A.f
B
CD11c+MHC-II+(%)
ns
Ctrl A.f
C
CD25+FOXP3+/CD4+(%)
ns
Ctrl A.f
D
CD3+CD4+(%)
ns
Ctrl A.f
E
Bone
CD11b+Gr-1+(%)
ns
Ctrl A.f
F
Spleen
CD11b+Gr-1+(%)
ns
Ctrl A.f
G
CD3+CD8+ (%)
*
Ctrl A.f
H
Mouse VEGF-A Concentration (pg/mL)
NC
siVEGF-A
NC+A.f
siVEGF-A+A.f
I
DAPI
Aspergillus fumigatus rRNA probe
CD11b
Merged
magnified
LLC Tumor
LUAD Tumor
J
DAPI
Aspergillus fumigatus rRNA probe
panCK
Merged
magnified
LLC Tumor
LUAD Tumor
K
DAPI
Aspergillus fumigatus rRNA probe
CD31
Merged
magnified
LLC Tumor
LUAD TUMOR

Figure EV3. *A. fumigatus* specifically co-localizes with $CD11b^+$ myeloid cells without altering $CD4^+$ T cells, macrophages, DCs or Tregs in the tumor microenvironment.

(**A–C**) The percentage of macrophages (F4/80^+ and $CD11b^+$ cells) ($p = 0.5584$), dendritic cells ($CD11c^+MHCII^+$) ($p = 0.4231$) and Treg cells ($CD25^+FOXP3^+CD4^+$) ($p = 0.0772$) in tumor tissues were detected by flow cytometry ($n = 4$–6 biological replicates). (**D**) Proportions of $CD3^+CD4^+$ T cells ($p = 0.7962$) in tumor tissues were detected by flow cytometry. ($n = 6$ biological replicates) (**E**, **F**) Proportions of total MDSCs ($CD11b^+Gr\text{-}1^+$) in bone and spleen tissues were detected by flow cytometry (Bone: $p = 0.0506$) (Spleen: $p = 0.4595$) ($n = 5$–6 biological replicates). (**G**) Proportions of $CD3^+CD8^+$ T cells in tumor tissues were detected by flow cytometry ($p = 0.0154$) ($n = 6$ biological replicates). (**H**) VEGF-A secretion in the culture supernatant of MDSCs was measured by ELISA. (NC and siVEGF-A: $p < 0.0001$; NC and NC + *A. f*: $p = 0.0027$; NC + *A. f* and siVEGF-A + *A. f*: $p < 0.0001$) ($n = 4$ biological replicates). (**I–K**) Representative FISH images showing *A. fumigatus* (red) and $CD11b^+$ myeloid cells (green), $panCK^+$ tumor cells (green), $CD31^+$ endothelium (green). Nuclei counterstained with DAPI (blue) (scale bars, 50μm). Data information: Data with error bars are represented as mean ± SEM. *$p < 0.05$, **$p < 0.01$, ****$p < 0.0001$ as determined by unpaired Student's t-test. ns indicating no significance.

 © The Author(s)

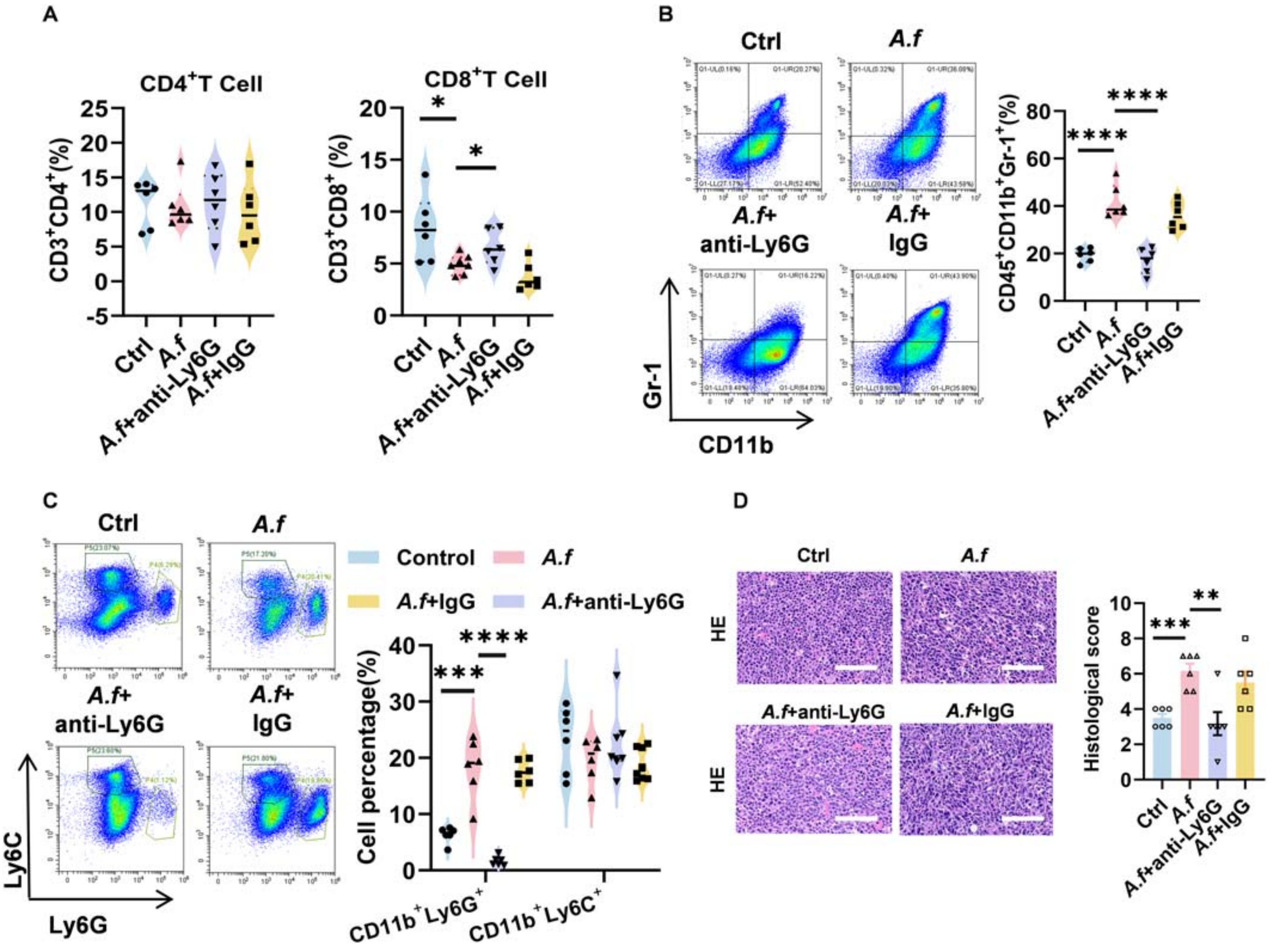

Figure EV4. G-MDSCs reduction enhances the immune response in tumor-bearing mice treated with peritumor *A. fumigatus* injections.

(**A**) Proportions of T cell subgroups ($CD3^+CD4^+$ T cells and $CD3^+CD8^+$ T cells) in tumor tissues were detected by flow cytometry. ($CD8^+$ T cells: Ctrl and *A. f*: $p = 0.0181$. *A f* and *A. f* + antiLy6G: $p = 0.0439$) ($n = 6$ biological replicates). (**B**, **C**) Proportions of total MDSCs ($CD45^+CD11b^+Gr\text{-}1^+$), G-MDSCs ($CD11b^+Ly6G^+$) and M-MDSCs ($CD11b^+Ly6C^+$) in tumor tissues were detected by flow cytometry (MDSCs: Ctrl and *A. f*: $p < 0.0001$. *A f* and *A. f* + antiLy6G: $p < 0.0001$; G-MDSCs: Ctrl and *A. f*: $p = 0.0003$. *A f* and *A. f* + antiLy6G: $p < 0.0001$) ($n = 6$ biological replicates). (**D**) HE histological analysis of tumors was shown by HE staining (scale bars, 100 μm) (Ctrl and *A. f*: $p = 0.0002$. *A f* and *A. f* + antiLy6G: $p = 0.0045$) ($n = 6$ biological replicates). Data information: Data with error bars are represented as mean ± SEM. Normality was assessed using the Shapiro-Wilk test. Two-group comparisons used the unpaired t-test (normal) or Mann-Whitney U test (non-normal). *$p < 0.05$, **$p < 0.01$, ***$p < 0.001$, ****$p < 0.0001$ as determined by unpaired Student's t-test or Mann-Whitney U test.

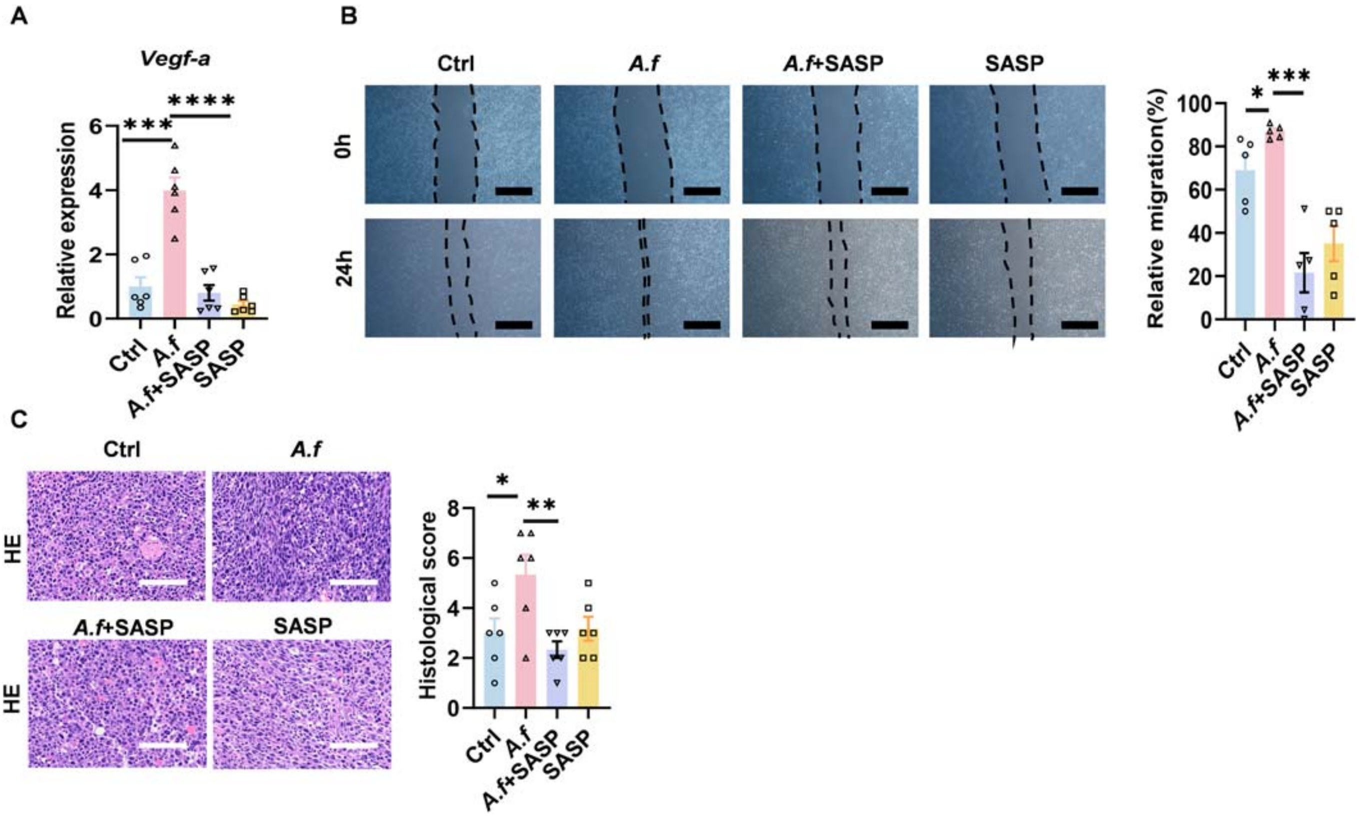

Figure EV5. SLC7A11 mediates *A. fumigatus*-induced pro-Angiogenic function of MDSCs.

The inhibitor of slc7a11, sulfasalazine (SASP) (2 μM), was added to the supernatant of the culture medium of MDSCs. Medium supernatants from MDSCs were collected and used as conditioned medium for HUVEC cells. (**A**) The gene expression of *Vegf-a* in MDSCs was detected by qPCR (Ctrl and *A. f*: $p = 0.0001$; *A. f* and *A. f* + SASP: $p < 0.0001$) ($n = 6$ biological replicates). (**B**) The migratory ability of HUVEC cells were assessed (scale bars, 500 μm) (Ctrl and *A. f*: $p = 0.0135$; *A. f* and *A. f* + SASP: $p = 0.0001$) ($n = 5$ biological replicates). (**C**) SASP (100 mg/kg) was injected intraperitoneally daily in the LLC mouse model. HE histological analysis of tumors was shown by HE staining (scale bars, 100 μm) (Ctrl and *A. f*: $p = 0.0400$; *A. f* and *A. f* + SASP: $p = 0.0062$) ($n = 6$ biological replicates). Data information: Data with error bars are represented as mean ± SEM. *$p < 0.05$, **$p < 0.01$, ***$p < 0.001$, ****$p < 0.0001$ as determined by unpaired Student's t-test.

 © The Author(s)
